# Soluble TREM2 engages cell-surface nucleolin to drive vascular permeability and malignant ascites in ovarian cancer

**DOI:** 10.1038/s44321-026-00452-2

**Published:** 2026-05-26

**Authors:** Qianqian Li, Yubo Zhang, Hui Luo, Yongjian Wu, Jingbo Qin, Lijie Wang, Qianqian Zhang, Ting Yu, Xi Huang, Shuang Guo, Bingfan Xie, Huanhuan He

**Affiliations:** 1 Guangdong Provincial Engineering Research Center of Molecular Imaging, The Fifth Affiliated Hospital of Sun Yat-sen University, 519000 Zhuhai, Guangdong China; 2 Guangdong-Hong Kong-Macao University Joint Laboratory of Interventional Medicine, The Fifth Affiliated Hospital of Sun Yat-sen University, 519000 Zhuhai, Guangdong China; 3 Center for Infection and Immunity, The Fifth Affiliated Hospital of Sun Yat-sen University, 519000 Zhuhai, Guangdong China; 4 College of Bioinformatics Science and Technology, Harbin Medical University, Harbin, 150000 China; 5 Department of Gynecology, The Fifth Affiliated Hospital of Sun Yat-sen University, 519000 Zhuhai, Guangdong China; 6 Faculty of Health Sciences, University of Macau, Taipa, 999078 Macau SAR China; 7 MoE Frontiers Science Center for Precision Oncology, University of Macau, Taipa, 999078 Macau SAR China

**Keywords:** Cancer

## Abstract

Ascites, a hallmark of advanced ovarian cancer, severely impairs patient quality of life and contributes to therapeutic resistance. Current management strategies remain primarily palliative, highlighting the urgent need to elucidate the underlying mechanisms. Although TREM2^+^ macrophages are enriched in malignant ascites, the role of soluble TREM2 (sTREM2) in regulating vascular function and ascites pathogenesis remains poorly understood. Here, we show that sTREM2 levels are elevated in malignant ascites and positively correlate with ascites volume. Using a combination of in vitro, in vivo, and ex vivo models, we demonstrate that sTREM2 promotes vascular permeability and drives ascites formation. Mechanistically, sTREM2 binds to nucleolin (NCL) on endothelial cells and activates the AKT/eNOS signaling pathway, which leads to VE-cadherin phosphorylation and ultimately vascular leakage. sTREM2-induced hyperpermeability was abolished by NCL knockdown or eNOS inhibition. Importantly, administration of a neutralizing antibody against sTREM2 significantly reduced ascites formation and tumor burden in mouse models. Our study identifies the sTREM2-NCL-AKT-eNOS axis as a critical driver of vascular leakage and malignant ascites in ovarian cancer, highlighting it as a promising therapeutic target.

## Synopsis

This study reveals that soluble TREM2 drives vascular hyperpermeability and malignant ascites in ovarian cancer by binding endothelial nucleolin and activating AKT/eNOS signaling, and its neutralization suppresses vascular leakage, ascites formation, and tumor progression.

- sTREM2 levels are significantly elevated in malignant ascites and positively correlate with ascites volume.
- sTREM2 disrupts endothelial barrier integrity in vitro, and promotes vascular leakage and ascites formation in vivo.
- sTREM2 directly binds cell-surface nucleolin on endothelial cells, activating the AKT/eNOS pathway and inducing VE-cadherin phosphorylation.
- Neutralization of sTREM2 with a specific antibody reduces vascular permeability, ascites accumulation, and tumor burden in preclinical models.

## Abstract (web summary)

This study reveals that soluble TREM2 drives vascular hyperpermeability and malignant ascites in ovarian cancer by binding endothelial nucleolin and activating AKT/eNOS signaling, and its neutralization suppresses vascular leakage, ascites formation, and tumor progression.

## Graphical abstract

**Figure:**
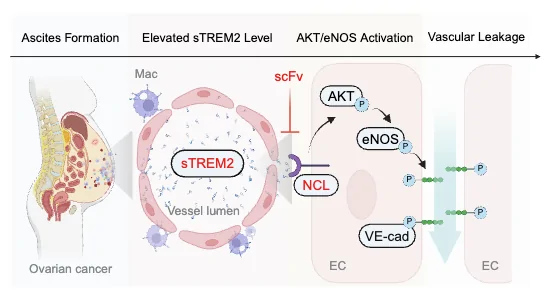

**Figure:**
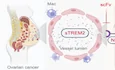

The paper explained **The problem**

Malignant ascites is a frequent and debilitating complication of advanced ovarian cancer, driven in part by increased vascular permeability. Current treatments only relieve symptoms temporarily and do not address the underlying cause. The mechanisms that drive fluid leakage from blood vessels into the abdominal cavity remain poorly understood, limiting the development of targeted therapies.

**Results**

Using patient samples, cell cultures, and mouse models, the authors found that a soluble protein called sTREM2 is highly elevated in malignant ascites and directly promotes vascular leakage. sTREM2 binds to a receptor called nucleolin on the surface of endothelial cells lining blood vessels. This binding activates a signaling pathway (AKT/eNOS) that weakens the connections between endothelial cells, making blood vessels leaky. Blocking sTREM2 with a specific antibody reversed this effect: it restored the integrity of the vessel wall, reduced fluid accumulation, and also slowed tumor growth in preclinical models.

**Impact**

These findings establish sTREM2 as a key driver of malignant ascites and a promising therapeutic target. Targeting sTREM2 could offer a mechanism‑based treatment to prevent or reduce ascites and may complement existing anti-angiogenic therapies. Furthermore, elevated sTREM2 levels in ascites may serve as a biomarker to identify high‑risk patients.

## Introduction

Ovarian cancer (OC) remains one of the most lethal gynecologic malignancies, with therapeutic challenges and poor outcomes intensifying in advanced stages (Malvezzi et al, 2016; Siegel et al, 2025). Malignant ascites, a hallmark of advanced OC, not only severely impairs patient quality of life and complicates surgical interventions but also promotes disease progression by facilitating tumor metastasis and chemoresistance (Ford et al, 2020; Rickard et al, 2021; Zheng et al, 2023). Current management relies on palliative approaches such as paracentesis and diuretics, which fail to address the underlying pathophysiology and carry risks of infection and metabolic disturbances (Berger et al, 2023). These limitations underscore the urgent need to elucidate the molecular mechanisms driving ascites formation for targeted therapeutic development.

The pathogenesis of malignant ascites involves multifactorial interactions, including dysregulated vascular permeability, impaired lymphatic drainage, and tumor-induced angiogenesis (Tamsma et al, 2001; Ford et al, 2020; Berger et al, 2023). Vascular hyperpermeability is particularly critical in this process, as it leads to fluid extravasation into the peritoneal cavity. Both tumor and non-tumor components within malignant ascites, along with various signaling molecules, directly or indirectly regulate vascular permeability (Moughon et al, 2015; Berger et al, 2023). Over the years, our group and others have demonstrated that targeting secreted factors, such as VEGF, IL-6, and IL-10, produced by macrophages (Almeida-Nunes et al, 2022) or inhibiting permeability-inducing macrophage subsets (Zhang et al, 2021; Luo et al, 2025) can reduce ascites production and tumor metastasis in OC. In summary, preserving endothelial barrier function is essential for controlling ascites and mitigating disease progression.

Recent studies have identified triggering receptor expressed on myeloid cells 2 (TREM2) positive macrophages as a functionally significant subset in multiple cancer types (Molgora et al, 2023). In OC, for example, elevated TREM2 expression is associated with advanced disease stage and poor prognosis (Binnewies et al, 2021). Soluble TREM2 (sTREM2), a proteolytically cleaved form detectable in blood, cerebrospinal fluid, and other body fluids (Deczkowska et al, 2020), mediates diverse biological functions. For instance, in optic neuritis, sTREM2 induces optic nerve dysfunction through interactions with heat shock protein 70 (Qin et al, 2024). Additionally, in Alzheimer’s disease, it acts on transglutaminase 2 to improve cognition (Zhang et al, 2023), whereas in breast cancer, it promotes disease progression (Yin et al, 2025). Despite these insights, the role of sTREM2 in OC pathogenesis, particularly in ascites formation, remains elusive.

In this study, we demonstrate that sTREM2 levels are elevated in ascites and reveal its functional role in promoting vascular permeability and driving ascites formation. Furthermore, we propose the dual value of sTREM2 as both a prognostic biomarker and a promising therapeutic target. These findings enhance our understanding of malignant ascites pathogenesis and provide a foundation for developing precise therapeutic strategies against OC and malignant ascites.

## Results

### sTREM2 is significantly elevated in the ascites of ovarian cancer patients

**Figure 1:**
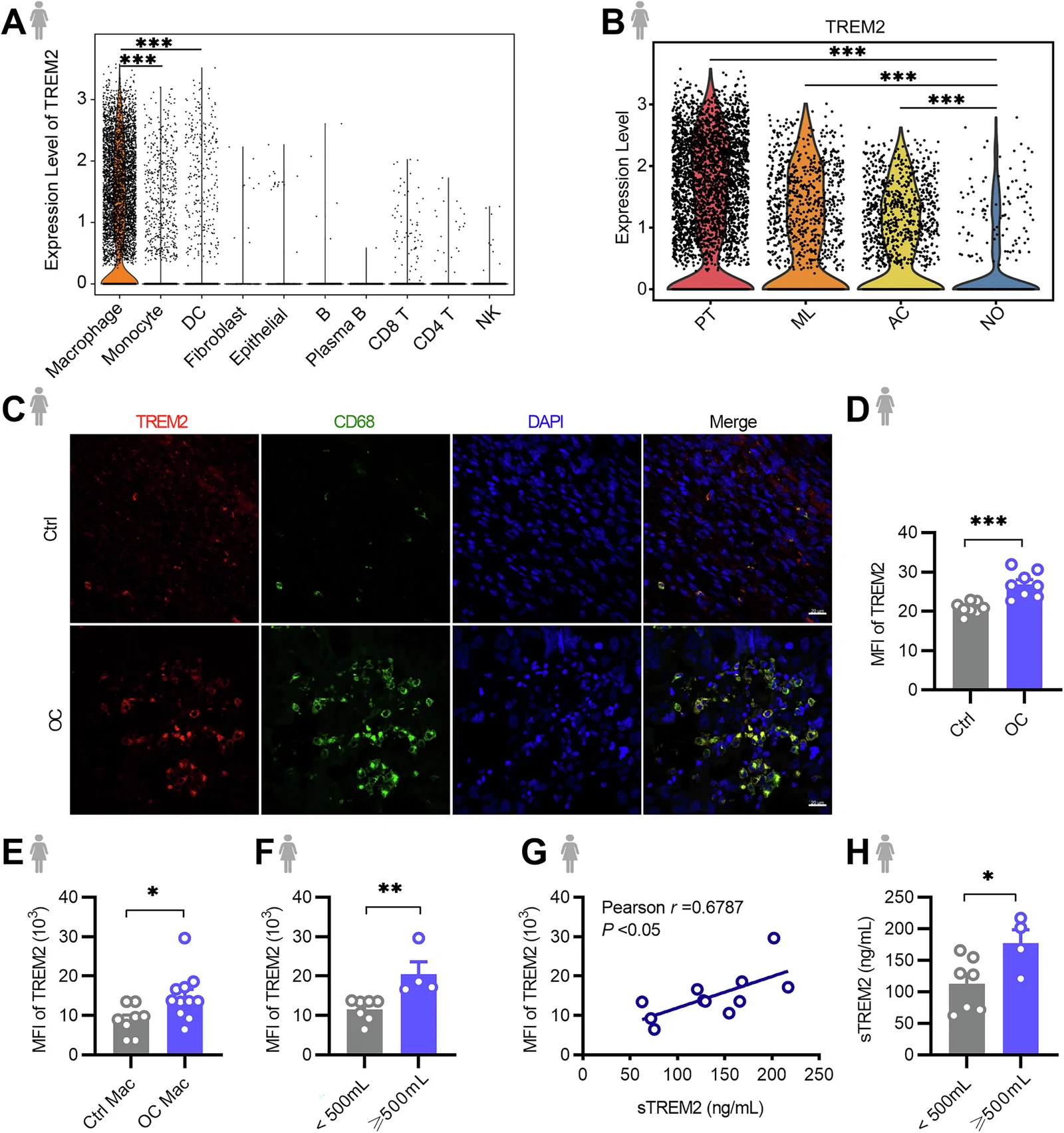
TREM2/sTREM2 levels are elevated in the malignant ascites of ovarian cancer. (**A**) Single-cell RNA-seq analysis of TREM2 expression across immune cell populations (violin plot, dataset: HRA002362, *n* = 16 patient samples). (**B**) Differential expression of TREM2 in tumor-associated macrophages from primary tumors (PT, *n* = 7 patient samples), omental metastatic lesions (ML, *n* = 3 patient samples), and malignant ascites (AC, *n* = 3 patient samples) compared with normal ovarian tissue (NO, *n* = 3 patient samples; violin plot; dataset: HRA002362). (**C**,** D**) Immunofluorescence analysis (**C**) and quantification (**D**) of TREM2 (red) expression in CD68^+^ macrophages (green) within human OC tissues (*n* = 8 patient samples per group). Nuclei are counterstained with DAPI (blue). Scale bar: 20 μm. (**E**) Flow cytometry analysis of TREM2 expression in ascites-derived macrophages from malignant cases (*n* = 8 patient samples) and in peritoneal macrophages from benign cases (*n* = 11 patient samples). (**F**) TREM2 MFI in macrophages stratified by ascites volume (<500 mL, *n* = 7 patient samples; ≥500 mL, *n* = 4 patient samples). (**G**) Positive correlation between macrophage TREM2 expression and sTREM2 levels in ascitic supernatants (*n* = 11 patient samples). (**H**) sTREM2 concentrations stratified by ascites volume (<500 mL, *n* = 7 patient samples; ≥500 mL, *n* = 4 patient samples), detected by ELISA. Ctrl denotes the control group, while OC represents the ovarian cancer group. MFI stands for mean fluorescence intensity. Data were shown as mean ± SEM. *P < 0.05; **P < 0.01; ***P < 0.001. Exact *p* values are provided in Appendix Table S2. Wilcoxon rank-sum test for (**A**, **B**), two-tailed Student’s *t*-test for (**D**–**F**, **H**); Pearson correlation for (**G**). Source data are available online for this figure.

**Figure EV1:**
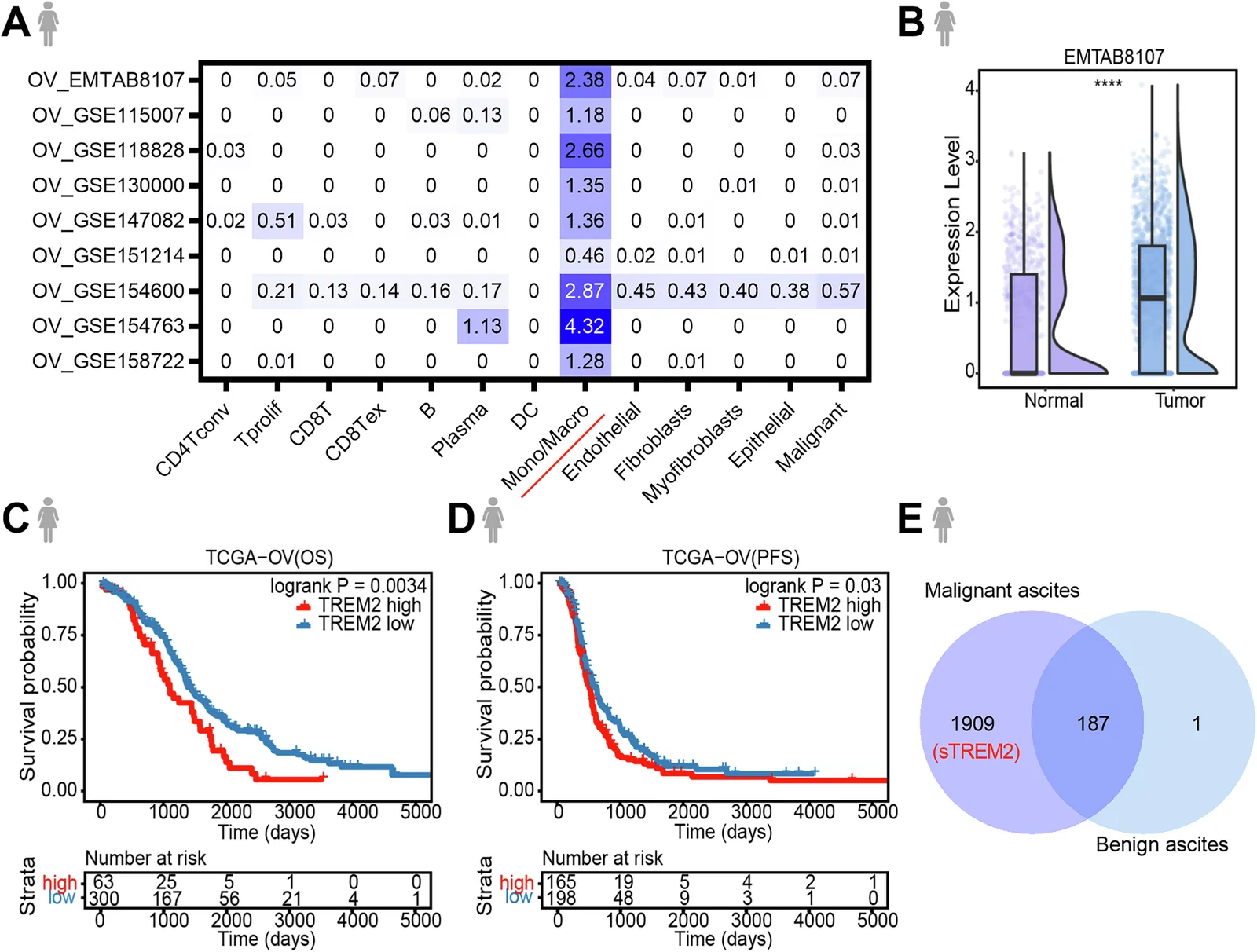
Single-cell and clinical characterization of TREM2 in ovarian cancer. (**A**) Heatmap displaying TREM2 expression patterns across immune cell populations from multiple single-cell RNA sequencing datasets (tumor immune single-cell hub). (**B**) Raincloud plot validating TREM2 expression in macrophages from the EMTAB-8107 dataset. Boxplots show the median (central line), the upper and lower quartiles (box limits, representing the 25th and 75th percentiles), and the whiskers indicating the minimum and maximum values (full range). (**C**,** D**) Kaplan–Meier survival analysis of (**C**) overall survival (OS) and (**D**) progression-free survival (PFS) in TCGA ovarian cancer patients stratified by TREM2 expression levels. (**E**) Venn diagram showing differentially expressed proteins between malignant and benign ascites. Data were shown as mean ± SEM. ***P < 0.001. Exact *p* values are provided in Appendix Table S2. Two-tailed Student’s *t*-test for (**B**); log-rank test for (**C**, **D**). Source data are available online for this figure.

To investigate the role of TREM2 in malignant ascites pathogenesis, we analyzed an in-house single-cell RNA sequencing dataset (HRA002362, Data ref: Luo et al, 2025) and external datasets from public repositories (including accession E-MTAB-8107 and the Tumor Immune Single-cell Hub). Analysis revealed that TREM2 was predominantly expressed by macrophages (Fig. 1A) and was significantly upregulated in primary tumors, metastatic lesions, and malignant ascites relative to normal ovarian tissues (Fig. 1B). These findings were validated in independent external datasets (Fig. EV1A,B). Critically, high TREM2 expression was significantly associated with shorter overall survival and progression-free survival in the Cancer Genome Atlas (TCGA) ovarian cancer cohort (Fig. EV1C,D). Immunofluorescence analysis of ovarian tissues confirmed that TREM2 expression on macrophages was elevated in OC patients (Fig. 1C,D). Flow cytometric analysis further revealed that TREM2 expression on macrophages was significantly higher in malignant ascites than in non-malignant peritoneal fluids (Fig. 1E). Moreover, patients with higher TREM2 levels tended to present with a larger ascites volume (Fig. 1F).

Given that TREM2 has a secreted form, sTREM2, which has been implicated in pathogenesis (Malvezzi et al, 2016; Zheng et al, 2023; Siegel et al, 2025), we quantified sTREM2 levels in ascitic supernatants by ELISA. sTREM2 levels were positively correlated with TREM2 expression (Fig. 1G). Consistent with the TREM2 expression data, sTREM2 levels were also significantly elevated in malignant ascites and were associated with increased ascites volume (Fig. 1H). This clinical finding is consistent with published proteomic data reporting the accumulation of sTREM2 in malignant ascites (Shender et al, 2014; Fig. EV1E). Collectively, these data demonstrate that sTREM2 is upregulated in malignant ascites.

### sTREM2 promotes vascular permeability

**Figure EV2:**
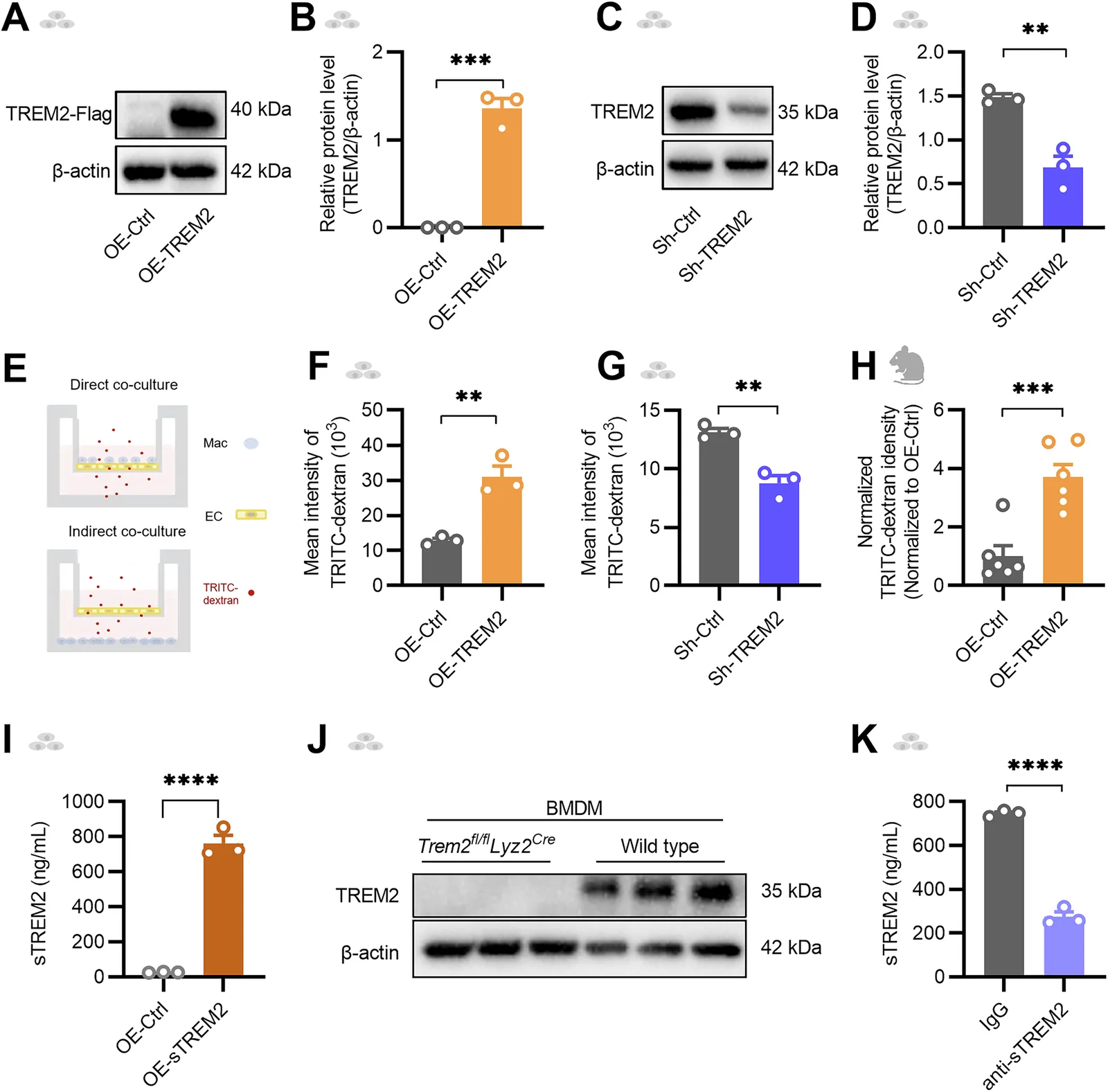
sTREM2 modulates vascular permeability through macrophage-dependent and independent mechanisms. (**A**,** B**) Western blot analysis (**A**) and quantification (**B**) of TREM2 expression in iBMDM cells overexpressing TREM2 (OE-TREM2) versus empty vector control (OE-Ctrl, *n* = 3 biological replicates). β-actin was used as a loading control. (**C**,** D**) Western blot analysis (**C**) and quantification (**D**) of TREM2 expression in iBMDM cells with TREM2 knockdown (Sh-TREM2) versus scramble control (Sh-Ctrl, *n* = 3 biological replicates). β-actin was used as a loading control. (**E**) Schematic diagram of direct and indirect coculture systems. (**F**) TRITC-dextran flux in C166 endothelial cells directly cocultured with OE-sTREM2 or OE-Ctrl iBMDM cells (*n* = 3 biological replicates). (**G**) TRITC-dextran flux in C166 cells cocultured with Sh-TREM2 or Sh-Ctrl iBMDM cells (*n* = 3 biological replicates). (**H**) Peritoneal vascular permeability in mice injected intraperitoneally with 1 × 10^6^ TREM2-overexpressing iBMDM versus control iBMDM cells (*n* = 6 mice). (**I**) sTREM2 levels in the culture supernatants of THP-1 cells, detected by ELISA (*n* = 3 biological replicates). (**J**) Western blot validation of TREM2 knockout in bone marrow-derived macrophages from *Trem2*^*fl/fl*^*Lyz2*^*Cre*^ mice. (**K**) sTREM2 levels in conditioned medium from sTREM2-overexpressing THP-1 cells before and after immunoprecipitation-mediated depletion with an anti-sTREM2 antibody, detected by ELISA (*n* = 3 biological replicates). Data were mean ± SEM. **P < 0.01, ***P < 0.001, ****P < 0.0001. Exact *p* values are provided in Appendix Table S2. Two-tailed Student’s *t*-test for (**B**, **D**, **F**–**I**, **K**). Source data are available online for this figure.

To determine whether TREM2/sTREM2 directly regulates vascular permeability and ascites formation, we established an in vitro coculture system consisting of endothelial cells and macrophages with differentially expressed TREM2 (Fig. EV2A–E). In a direct coculture system, macrophage-specific TREM2 overexpression significantly increased vascular leakage and compromised endothelial barrier integrity (Fig. EV2F), whereas TREM2 knockdown yielded the opposite effect (Fig. EV2G). These in vitro findings were further validated in vivo. Intraperitoneal injection of TREM2-overexpressing macrophages induced significantly more TRITC-dextran leakage than injection of control macrophages (Fig. EV2H), confirming that macrophage TREM2 directly regulates vascular permeability.

**Figure 2:**
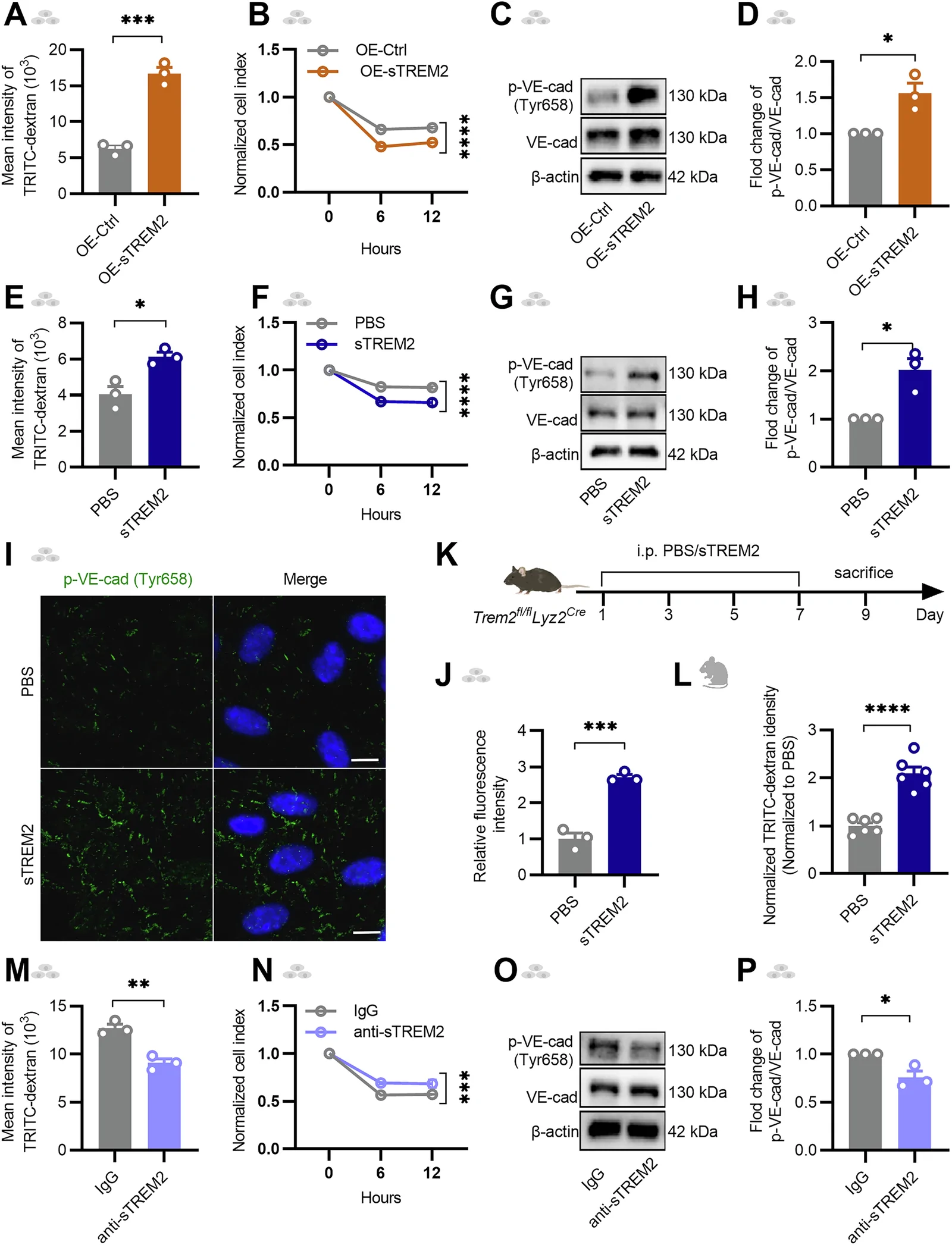
sTREM2 promotes vascular permeability. (**A**) TRITC-dextran flux quantification in HUVECs indirectly cocultured with THP-1 macrophages overexpressing sTREM2 (OE-sTREM2) or empty vector (OE-Ctrl, *n* = 3 biological replicates). (**B**) Cellular permeability of HUVECs exposed to OE-sTREM2 or OE-Ctrl THP-1 macrophages (*n* = 3 biological replicates). (**C**,** D**) Western blot analysis (**C**) and quantification (**D**) of p-VE-cadherin (Tyr658) and total VE-cadherin in HUVECs incubated with conditioned medium from OE-sTREM2 or OE-Ctrl THP-1 macrophages (*n* = 3 biological replicates). β-actin was used as a loading control. (**E**) TRITC-dextran flux in HUVECs treated with 500 ng/mL recombinant sTREM2 versus PBS (*n* = 3 biological replicates). (**F**) Cellular permeability of HUVECs exposed to 500 ng/mL recombinant sTREM2 or PBS (*n* = 3 biological replicates). (**G**,** H**) Western blot analysis (**G**) and quantification (**H**) of p-VE-cadherin (Tyr658) and VE-cadherin in HUVECs treated with 500 ng/mL recombinant sTREM2 versus PBS (*n* = 3 biological replicates). β-actin was used as a loading control. (**I**,** J**) Immunofluorescence analysis (**I**) and quantification (**J**) of p-VE-cadherin (Tyr658, green) in HUVECs treated with 500 ng/mL recombinant sTREM2 versus PBS (*n* = 3 biological replicates). Nuclei are stained with DAPI (blue). Scale bar: 10 μm. (**K**) Schematic diagram of murine intraperitoneal sTREM2 administration protocol. (**L**) Peritoneal vascular permeability in mice injected with sTREM2 (200 μg/dose, every other day, four doses total) versus control (*n* = 6 mice per group). (**M**) TRITC-dextran flux in HUVECs treated with conditioned medium from OE-sTREM2 THP-1 macrophages ± sTREM2 depletion (*n* = 3 biological replicates). (**N**) Cellular permeability assessment of HUVECs exposed to conditioned medium from OE-sTREM2 THP-1 macrophages ± sTREM2 depletion (*n* = 3 biological replicates). (**O**,** P**) Western blot analysis (**O**) and quantification (**P**) of p-VE-cadherin (Tyr658) and VE-cadherin in HUVECs incubated with conditioned medium with or without sTREM2 depletion (*n* = 3 biological replicates). β-actin was used as a loading control. Data are shown as mean ± SEM. *P < 0.05; **P < 0.01; ***P < 0.001; ****P < 0.0001. Exact *p* values are provided in Appendix Table S2. Two-tailed Student’s *t*-test for (**A**, **D**, **E**, **H**, **J**, **L**, **M**, **P**); two-way ANOVA analysis for (**B**, **F**, **N**). Source data are available online for this figure.

To investigate whether sTREM2 directly mediates these effects, we generated macrophages that overexpressed sTREM2 (Fig. EV2I). These macrophages recapitulated the barrier-disruptive phenotype in an indirect coculture system (Fig. 2A–D). Consistently, treatment of endothelial cells with recombinant sTREM2 directly induced vascular leakage (Fig. 2E,F), and promoted VE-cadherin phosphorylation (Fig. 2G–J). Importantly, in vivo administration of sTREM2 to myeloid cell-specific *Trem2* conditional knockout mice (*Trem2*^*fl/fl*^*Lyz2*^*Cre*^) significantly increased peritoneal vascular permeability (Figs. 2K,L and EV2J). Furthermore, endothelial cells exposed to sTREM2-depleted conditioned medium from macrophages exhibited attenuated permeability (Figs. 2M–P and EV2K). Collectively, these results demonstrate that sTREM2 directly compromises endothelial barrier function.

### sTREM2 drives ascites formation in murine ovarian cancer

**Figure 3:**
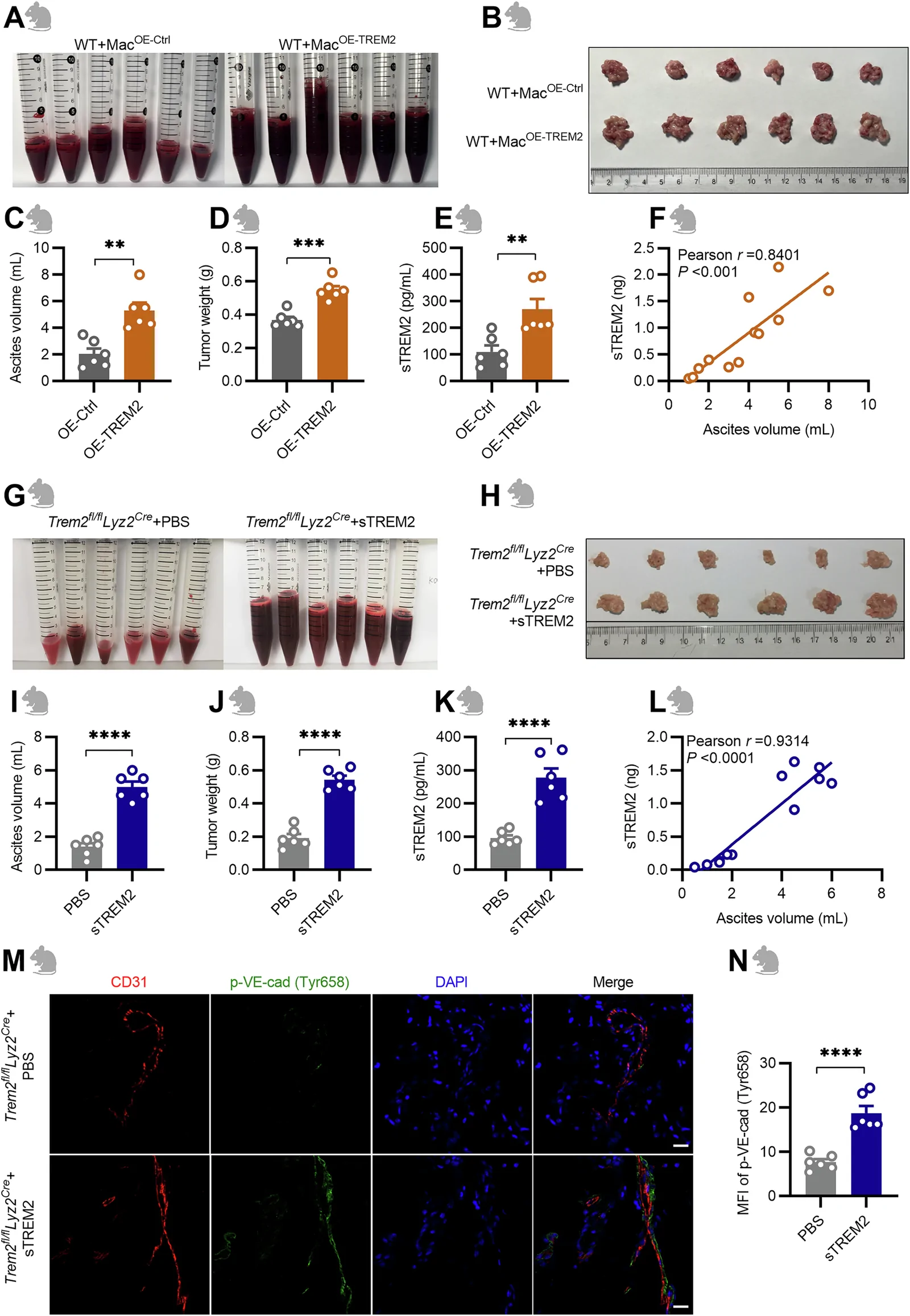
sTREM2 drives malignant ascites formation in ovarian cancer. (**A**–**D**) Representative images and quantification of ascites volume (**A**,** C**) and tumor burden (**B**,** D**) in mice intraperitoneally injected with 1 × 10⁶ OE-TREM2 or OE-Ctrl iBMDM cells together with 1 × 10⁶ ID8 cells (*n* = 6 mice per group). (**E**) sTREM2 levels in ascitic supernatants from (**A**), detected by ELISA (*n* = 6 mice per group). (**F**) Correlation analysis between ascites volume and sTREM2 levels from (**E**, *n* = 12 mice). (**G**–**J**) Representative images and quantification of ascites volume (**G**,** I**) and tumor burden (**H**,** J**) in mice injected with ID8 cells (1 × 10⁶) and recombinant sTREM2 (200 μg/mouse/dose in 100 μL PBS, 4 doses total) or PBS (*n* = 6 mice per group). (**K**) sTREM2 in ascitic supernatants from the mice in (**G**), detected by ELISA (*n* = 6 mice per group). (**L**) Correlation between ascites volume and sTREM2 levels from (**K**, *n* = 12 mice). (**M**,** N**) Immunofluorescence analysis (**M**) and quantification (**N**) of p-VE-cadherin (Tyr658) (green) in mesenteric vessels and tumor vasculature (*n* = 6 mice per group). CD31 (red) marks endothelium; nuclei are counterstained with DAPI (blue). MFI stands for mean fluorescence intensity. Scale bars: 20 μm. Data were shown as mean ± SEM. **P < 0.01; ***P < 0.001; ****P < 0.0001. Exact *p* values are provided in Appendix Table S2. Two-tailed Student’s *t*-test for (**C**–**E**, **I**–**K**, **N**); Pearson correlation for (**F**, **L**). Source data are available online for this figure.

**Figure EV3:**
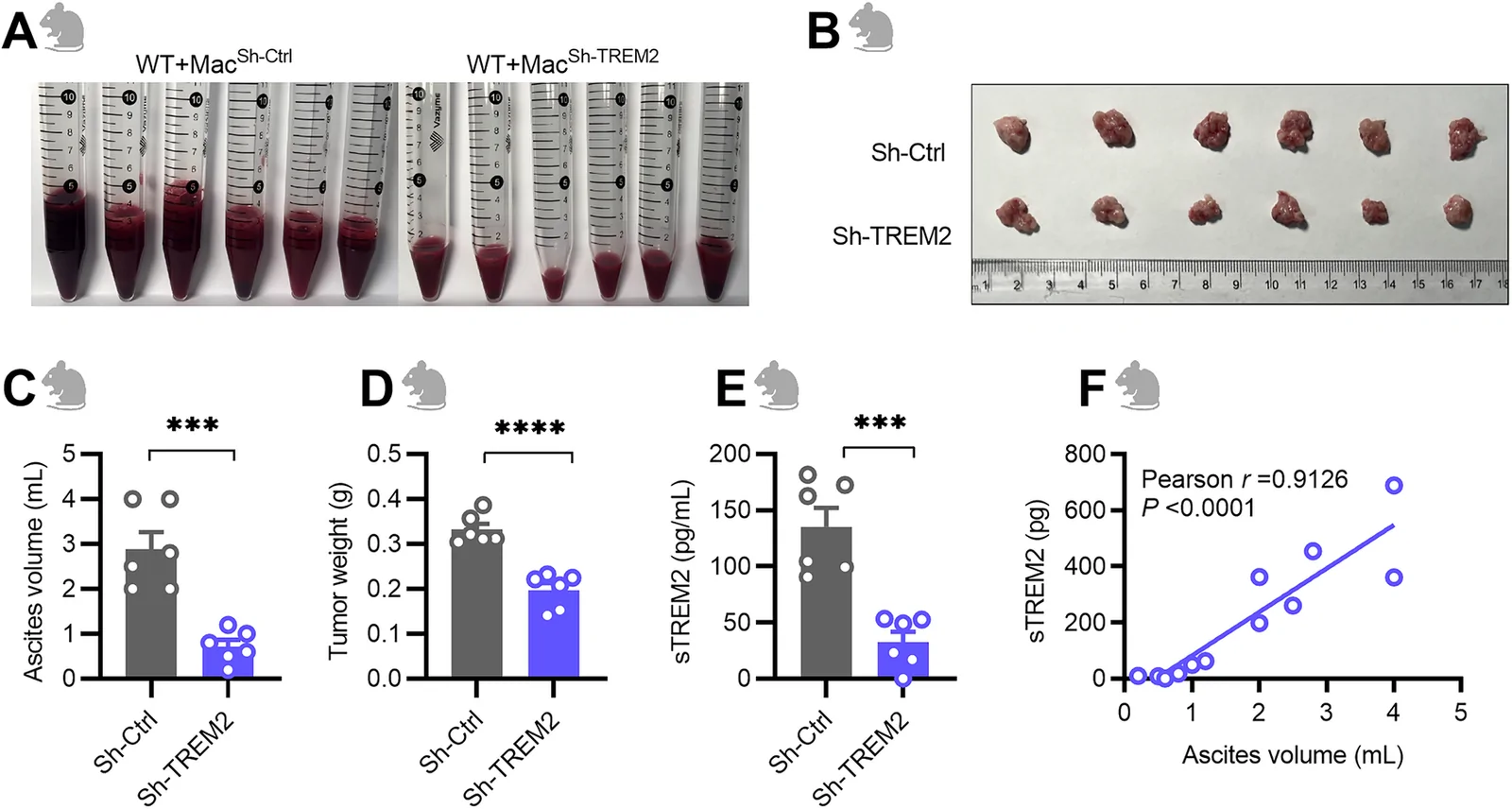
TREM2 knockdown inhibits malignant ascites formation in ovarian cancer. (**A**–**D**) Representative images (**A**,** B**) and quantification (**C**,** D**) of ascites (**A**,** C**) and tumor burden (**B**,** D**) in mice injected intraperitoneally with ID8 cells and Sh-TREM2 iBMDM cells versus Sh-Ctrl iBMDM (*n* = 6 mice per group). (**E**) sTREM2 levels in ascitic supernatants from (**A**), detected by ELISA (*n* = 6 mice per group). (**F**) Correlation analysis between ascites volume and sTREM2 concentration from (**E**, *n* = 12 mice). Data were shown as mean ± SEM. ***P < 0.001; ****P < 0.0001. Exact *p* values are provided in Appendix Table S2. Two-tailed Student’s *t*-test for (**C**–**E**); Pearson correlation for (**F**). Source data are available online for this figure.

To explore the role of TREM2/sTREM2 in ascites formation, we established a murine OC model by intraperitoneally co-injecting ID8 cells and macrophages with modulated TREM2 expression. Notably, TREM2-overexpressing macrophages significantly promoted ascites formation and increased tumor burden relative to control macrophages (Fig. 3A–D). In contrast, TREM2-knockdown macrophages suppressed both ascites formation and tumor growth (Fig. EV3A–D). Correspondingly, ascitic supernatants from mice injected with TREM2-overexpressing macrophages contained higher sTREM2 levels (Fig. 3E), whereas supernatant from mice injected with TREM2-knockdown macrophages showed reduced sTREM2 levels (Fig. EV3E). Furthermore, sTREM2 levels in ascitic supernatants strongly correlated with ascites volume (Figs. 3F and EV3F).

To directly assess the role of sTREM2 in ascites production, ID8 tumor cells were injected into *Trem2*^*fl/fl*^*Lyz2*^*Cre*^ mice, followed by intraperitoneal administration of sTREM2 or PBS. The results showed that sTREM2 induced more ascites and peritoneal tumors compared with the PBS control (Fig. 3G–J). Furthermore, a positive correlation was observed between ascites sTREM2 levels and ascites volume (Fig. 3K, L). Immunofluorescence analysis of mesenteric vessels and tumor vasculature from these mice revealed enhanced phosphorylation of VE-cadherin (Fig. 3M, N), which recapitulated the in vitro findings. In conclusion, these results indicate that sTREM2 promotes vascular hyperpermeability and drives ascites production in OC.

### NCL is a functional receptor for sTREM2 in endothelial cells

**Figure 4:**
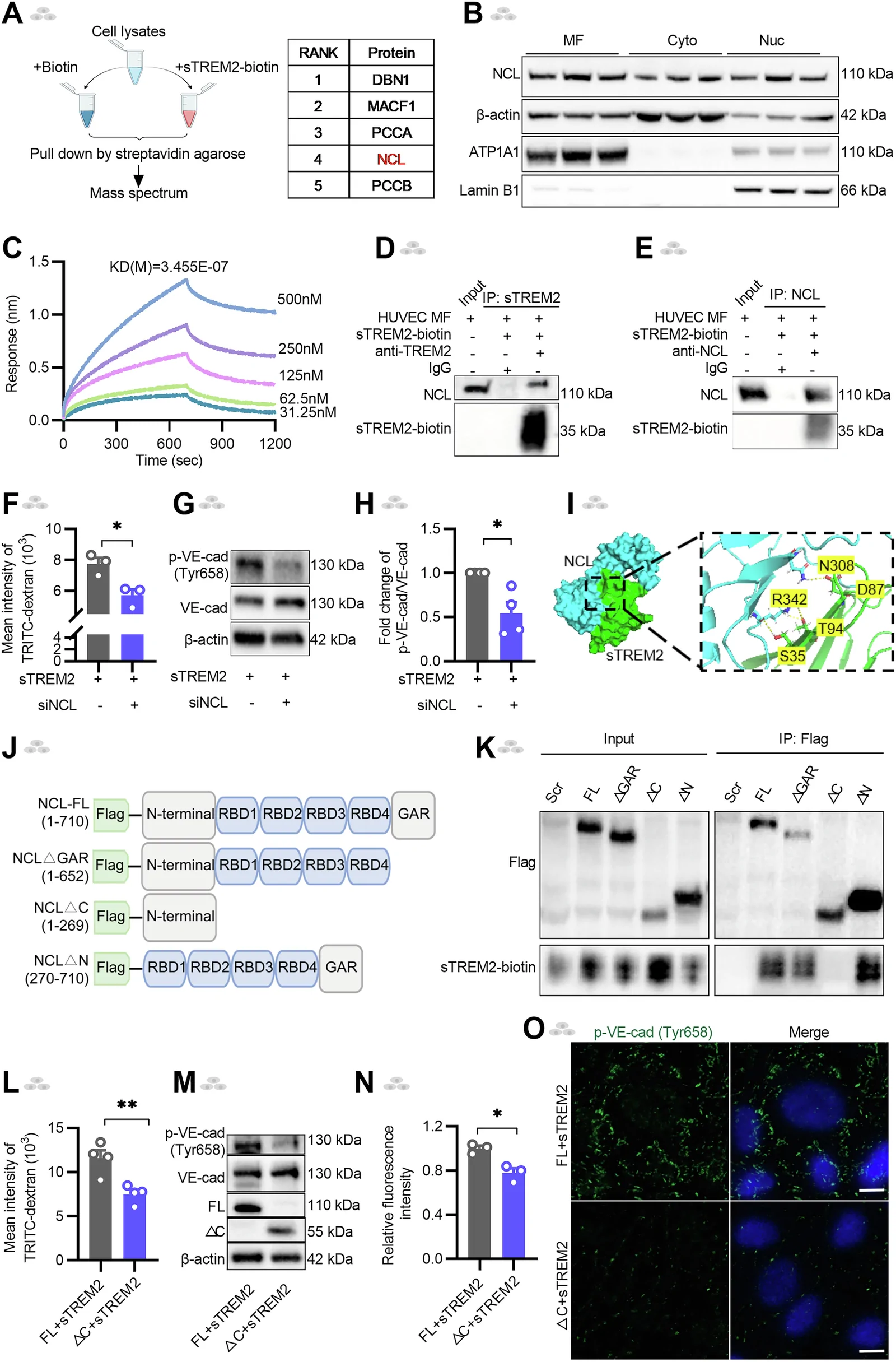
NCL serves as a functional cell-surface receptor for sTREM2 in endothelial cells. (**A**) Schematic workflow of pull-down assay coupled with mass spectrometry analysis, with identified proteins ranked by abundance. (**B**) Western blot showing subcellular distribution of NCL across different cellular fractions. Nuc nucleus, MF plasma membrane, Cyto cytosol. (**C**) Bio-layer Interferometry analysis of binding kinetics between biotinylated sTREM2 (sTREM2-biotin) and recombinant NCL. (**D**,** E**) Co-immunoprecipitation confirming interaction between sTREM2-biotin and endogenous NCL in HUVEC membrane fractions using anti-TREM2 antibody (**D**) or anti-NCL antibody (**E**). (**F**) TRITC-dextran flux in NCL-knockdown HUVECs treated with sTREM2 (500 ng/mL) versus controls (*n* = 3 biological replicates). (**G**,** H**) Western blot analysis (**G**) and quantification (**H**) of p-VE-cadherin (Tyr658) in NCL-knockdown HUVECs treated with sTREM2 (500 ng/mL, *n* = 4 biological replicates). β-actin was used as a loading control. (**I**) Molecular docking model of sTREM2-NCL interaction. (**J**) Domain architecture of Flag-tagged NCL constructs: full-length (NCL-FL1-710), ΔGAR (NCLΔGAR1-652), ΔC-terminal (NCLΔC1-269), and ΔN-terminal (NCLΔN270-710). (**K**) Co-immunoprecipitation analysis of sTREM2-biotin binding to NCL truncation mutants (anti-Flag and anti-biotin blots). (**L**) TRITC-dextran flux in NCL-knockdown HUVECs rescued with NCL truncation mutants and treated with 500 ng/mL sTREM2 (*n* = 4 biological replicates). (**M**) Western blot analysis of p-VE-cadherin (Tyr658) in the same experimental groups as (**L**). β-actin was used as a loading control. (**N**,** O**) Immunofluorescence analysis (**N**) and quantification (**O**) of p-VE-cadherin (Tyr658) (green, pseudo-colored) in the same experimental groups as (**L**, *n* = 3 biological replicates). Nuclei are stained with DAPI (blue). Scale bar: 10 μm. Data were shown as mean ± SEM. *P < 0.05; **P < 0.01. Exact *p* values are provided in Appendix Table S2. Two-tailed Student’s *t*-test for (**F**, **H**, **L**, **N**). Source data are available online for this figure.

Although the functions of sTREM2 in macrophages, neurons and tumor cells have been extensively characterized (Qin et al, 2024; Zhang et al, 2023; Yin et al, 2025; Zhong et al, 2017), its biological effects on endothelial cells remain undefined. To identify the endothelial receptor for sTREM2, we performed pull-down assays followed by mass spectrometry in human umbilical vein endothelial cells (HUVECs). Intriguingly, NCL was detected as the most abundant cell-surface binding partner (Fig. 4A; Appendix Fig. S1A). Despite its primary nucleolar localization, NCL also functions as a cell-surface receptor for tumorigenic and angiogenic ligands (Song et al, 2012; Zhuo et al, 2010). To validate NCL as the functional receptor for sTREM2, we first performed subcellular fractionation assays, which confirmed the presence of NCL on the plasma membrane of HUVECs (Fig. 4B). Next, Bio-Layer Interferometry analysis revealed an affinity between sTREM2 and NCL (Fig. 4C). Co-immunoprecipitation analysis and pull-down assays of membrane proteins further confirmed the interaction between sTREM2 and NCL (Figs. 4D,E; Appendix Fig. S1B). Additionally, genetic knockdown of NCL significantly attenuated sTREM2-mediated endothelial hyperpermeability (Fig. 4F–H; Appendix Fig. S1C,D).

NCL is a multidomain protein consisting of an N-terminal domain, four central RNA-binding domains (RBDs) and a C-terminal glycine-arginine-rich domain (Jia et al, 2017). To identify the specific NCL domain mediating sTREM2 binding, we first performed molecular docking, which predicted the RBDs as the putative binding site for sTREM2 (Fig. 4I). To validate this prediction, we transiently transfected cells with Flag-tagged full-length NCL or various truncation constructs (Fig. 4J; Appendix Fig. S1E) and performed co-immunoprecipitation. Notably, only the C-terminal deletion mutant, which lacks the RBDs, failed to bind sTREM2 (Fig. 4K). Critically, in NCL-knockdown cells, the sTREM2-induced pro-permeability phenotype was observed upon transfection with full-length NCL, but not with the C-terminal deletion mutant (Fig. 4L–O). Taken together, these results indicate that the RBDs of cell-surface NCL are essential for sTREM2 to exert its effects on permeability.

### sTREM2 mediates the AKT/eNOS signaling pathway to regulate vascular permeability

**Figure 5:**
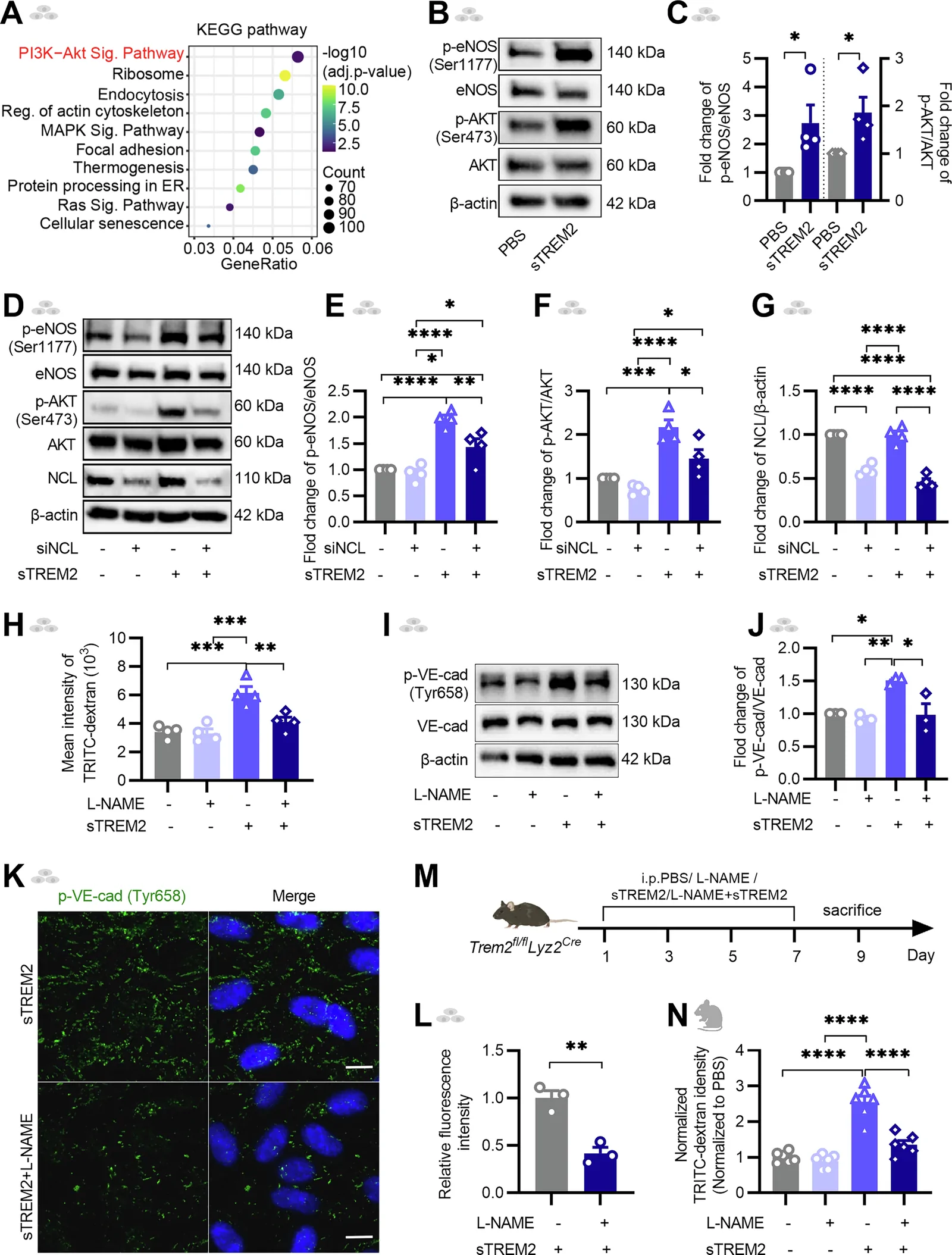
sTREM2 regulates vascular permeability through the AKT/eNOS signaling pathway. (**A**) KEGG pathway analysis of differentially expressed genes in HUVECs treated with 500 ng/mL sTREM2 or control (RNA-seq). (**B**,** C**) Western blot analysis (**B**) and quantification (**C**) of phospho-AKT (p-AKT) and phospho-eNOS (p-eNOS) in HUVECs treated with 500 ng/mL sTREM2 or PBS (*n* = 4 biological replicates). β-actin was used as a loading control. (**D**–**G**) Western blot analysis (**D**) and quantification (**E**–**G**) of p-AKT, p-eNOS and NCL in NCL-knockdown or control HUVECs treated with 500 ng/mL sTREM2 or PBS (*n* = 4 biological replicates). β-actin was used as a loading control. (**H**) TRITC-dextran flux in HUVECs incubated with 1 mM eNOS inhibitor L-NAME for 30 min prior to treatment with 500 ng/mL sTREM2 (*n* = 4 biological replicates). (**I**,** J**) Western blot analysis (**I**) and quantification (**J**) of p-VE-cadherin (Tyr658) in HUVECs incubated with L-NAME prior to treatment with 500 ng/mL sTREM2 (*n* = 3 biological replicates). β-actin was used as a loading control. (**K**,** L**) Immunofluorescence analysis (**K**) and quantification (**L**) of p-VE-cadherin (Tyr658) (green) in HUVECs incubated with L-NAME prior to treatment with 500 ng/mL sTREM2 (*n* = 3 biological replicates). Nuclei are counterstained with DAPI (blue). Scale bar: 10 μm. (**M**) Schematic diagram of L-NAME treatment protocol in vivo. (**N**) Peritoneal vascular permeability in mice treated with intraperitoneal sTREM2 (200 μg/mouse/dose, every other day, four doses total) and L-NAME (625 μg/mouse/dose, every other day, four doses total; *n* = 6 mice per group). Data were shown as mean ± SEM. *P < 0.05; **P < 0.01; ***P < 0.001; ****P < 0.0001. Exact *p* values are provided in Appendix Table S2. Over-representation analysis for (**A**); two-tailed Student’s *t*-test for (**C**, **L**); one-way ANOVA for (**E**–**H**, **J**, **N**). Source data are available online for this figure.

To elucidate the mechanism underlying sTREM2-mediated vascular permeability, we performed RNA sequencing on sTREM2-treated HUVECs. KEGG pathway analysis of the differentially expressed genes revealed significant enrichment of the PI3K/AKT signaling pathway (Fig. 5A). Although interactions between NCL and the PI3K/AKT pathway have been documented in malignant cells (Shin et al, 2018; Liu et al, 2023; Xu et al, 2025), their role in endothelial cells remains poorly characterized. Additionally, endothelial nitric oxide synthase (eNOS), a key downstream effector of PI3K/AKT signaling, is known to regulate vascular permeability by promoting VE-cadherin phosphorylation (Masoumi Moghaddam et al, 2012; Cui et al, 2013; Sjöberg et al, 2023; Li et al, 2024). Based on these findings, we hypothesized that sTREM2 may act through the AKT/eNOS signaling axis.

**Figure EV4:**
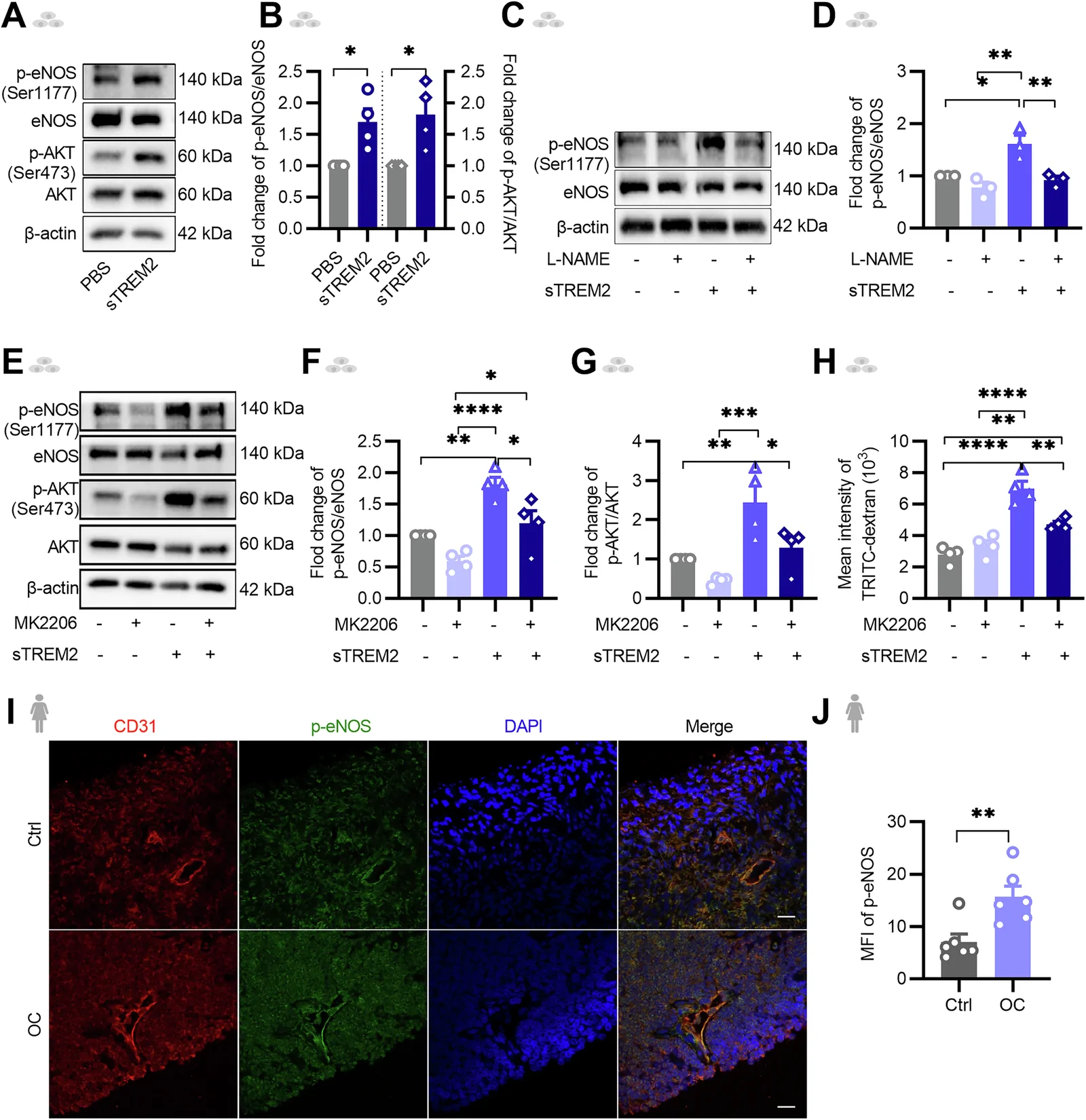
Pharmacological inhibition of AKT/eNOS signaling blocks sTREM2-mediated effects. Pharmacological inhibition of AKT/eNOS signaling blocks sTREM2-mediated effects (**A**,** B**) Western blot analysis (**A**) and quantification (**B**) of p-AKT and p-eNOS in 500 ng/mL sTREM2-treated C166 cells (*n* = 4 biological replicates). β-actin was used as a loading control. (**C**,** D**) Western blot analysis (**C**) and quantification (**D**) of eNOS phosphorylation inhibition by 1 mM L-NAME for 30 min (*n* = 3 biological replicates). β-actin was used as a loading control. (**E**–**G**) Western blot analysis (**E**) and quantification (**F**,** G**) of AKT and eNOS phosphorylation in HUVECs incubated with 1uM MK2206 for 1 h prior to treatment with 500 ng/mL sTREM2 (*n* = 4 biological replicates). β-actin was used as a loading control. (**H**) TRITC-dextran permeability assay in HUVECs treated with sTREM2  ± AKT inhibitor MK2206 (*n* = 4 biological replicates). (**I**,** J**) Immunofluorescence analysis (**I**) and quantification (**J**) of p-eNOS (green) in the vasculature of human OC tissues (*n* = 6 patient samples per group). CD31 (red) marks endothelium; nuclei are counterstained with DAPI (blue). MFI stands for mean fluorescence intensity. Scale bar: 20 μm. Data were shown as mean ± SEM. *P < 0.05; **P < 0.01; ***P < 0.001; ****P < 0.0001. Exact *p* values are provided in Appendix Table S2. Two-tailed Student’s *t*-test for (**B**, **J**); one-way ANOVA for (**D**, **F**–**H**). Source data are available online for this figure.

Strikingly, sTREM2 enhanced the phosphorylation of AKT and eNOS in endothelial cells (Figs. 5B,C and EV4A,B). This activation was abolished by NCL knockdown (Fig. 5D–G). To functionally investigate this mechanism, we treated endothelial cells with the eNOS inhibitor L-NAME, which abolished the sTREM2-mediated endothelial hyperpermeability (Figs. 5H–L and EV4C,D). Similarly, inhibition of AKT phosphorylation with MK2206 produced effects comparable to those of L-NAME (Fig. EV4E–H). Furthermore, in vivo permeability assays showed that intraperitoneal administration of L-NAME effectively suppressed sTREM2-induced vascular leakage (Fig. 5M,N). Critically, we observed a significant increase in p-eNOS signal within endothelial cells of ovarian cancer samples relative to controls, consistent with our in vitro and in vivo observations (Fig. EV4I,J). Collectively, these results support a model in which NCL-dependent AKT/eNOS signaling contributes to sTREM2-mediated vascular permeability.

### Targeting sTREM2 suppresses ascites formation in ovarian cancer

**Figure 6:**
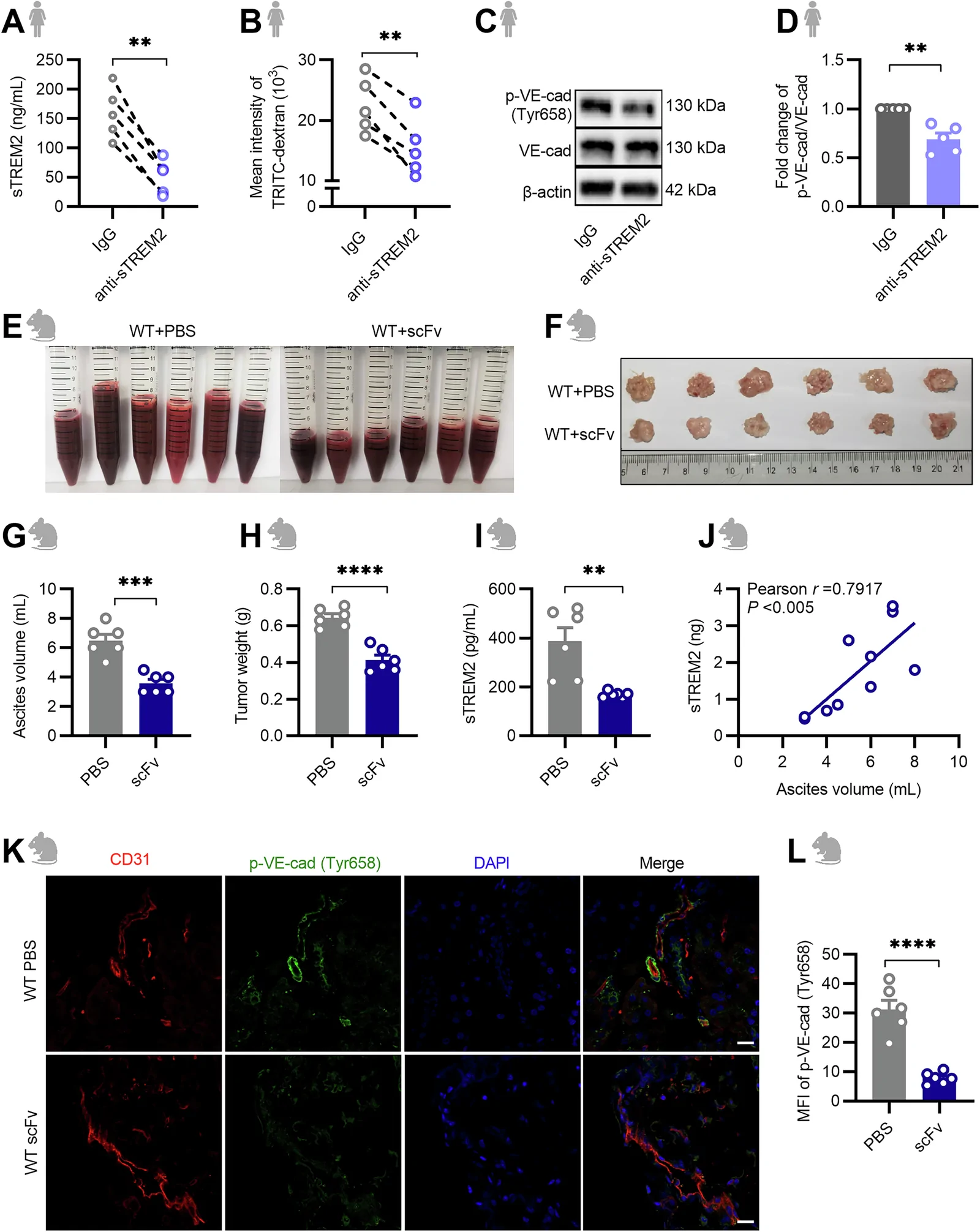
Targeting sTREM2 inhibits ascites formation in ovarian cancer. (**A**) Validation of sTREM2 depletion in ovarian cancer ascites by ELISA (*n* = 5 patient samples). (**B**) TRITC-dextran permeability assay in HUVECs treated with sTREM2-depleted versus control ascites (*n* = 5 patient samples). (**C**,** D**) Western blot analysis (**C**) and quantification (**D**) of p-VE-cadherin (Tyr658) in HUVECs exposed to sTREM2-depleted or control ascites (*n* = 5 patient samples). β-actin was used as a loading control. (**E**–**H**) Representative images (**E**,** F**) and quantification (**G**,** H**) of ascites volume (**E**,** G**) and tumor burden (**F**,** H**) in mice injected intraperitoneally with ID8 cells and anti-sTREM2 scFv (200 μg/mouse/dose in 100 μL PBS, four doses total) or PBS (control; *n* = 6 mice per group). (**I**) sTREM2 levels in ascitic supernatants from (**E**), detected by ELISA (*n* = 6 mice per group). (**J**) Correlation analysis between ascites volume and sTREM2 levels from (**I**, *n* = 12 mice). (**K**,** L**) Immunofluorescence analysis (**K**) and quantification (**L**) of p-VE-cadherin (Tyr658) (green) in mesenteric vessels and tumor vasculature (*n* = 6 mice per group). CD31 (red) marks endothelium; nuclei are counterstained with DAPI (blue). MFI stands for mean fluorescence intensity. Scale bar: 20 μm. Data were shown as mean ± SEM. **P < 0.01; ***P < 0.001; ****P < 0.0001. Exact *p* values are provided in Appendix Table S2. Two-tailed Student’s *t*-test for (**A**, **B**, **D**, **G**–**I**, **L**); Pearson correlation for (J). Source data are available online for this figure.

**Figure EV5:**
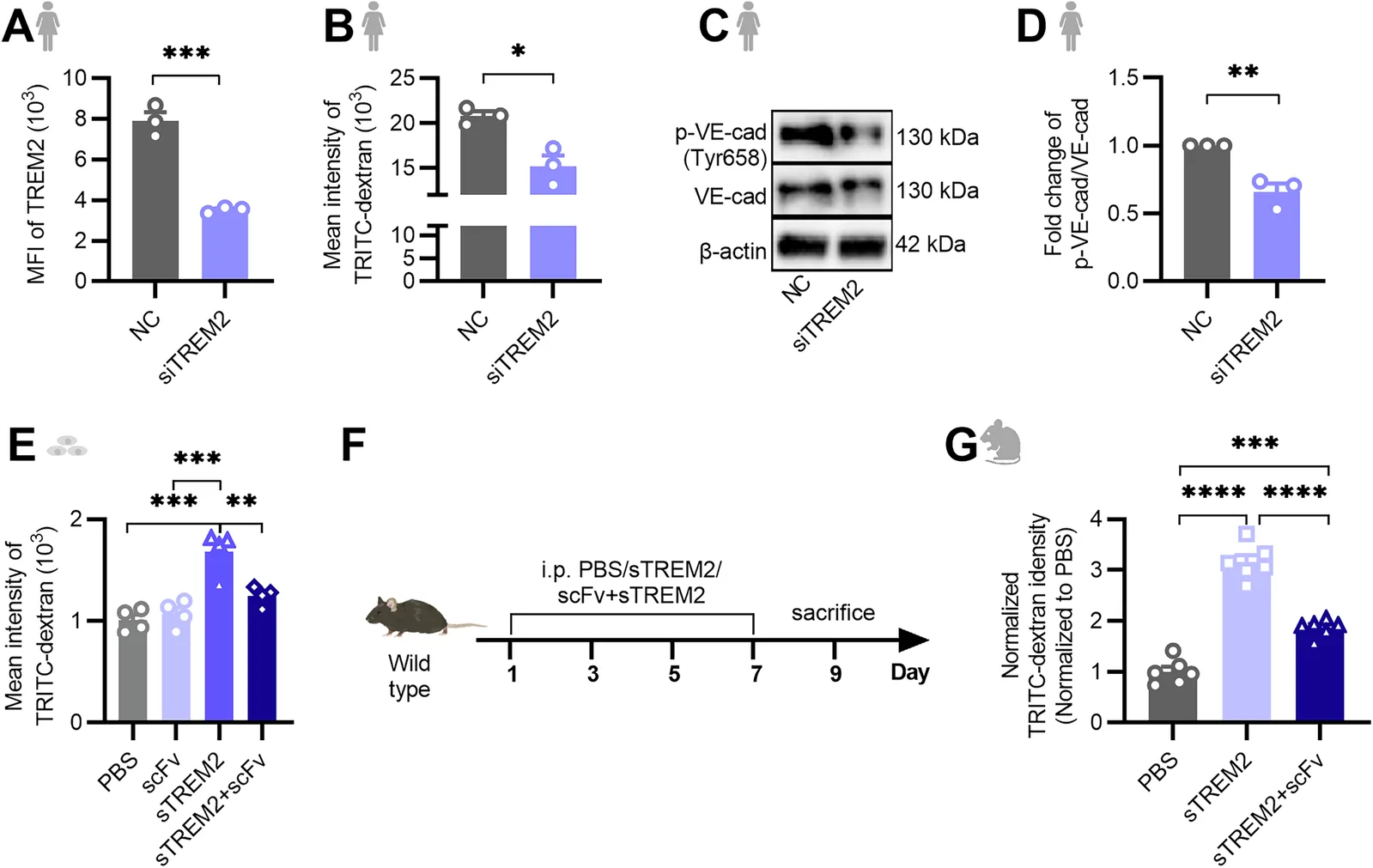
Genetic and pharmacological targeting of TREM2 reduces vascular permeability. Genetic and pharmacological targeting of TREM2 reduces vascular permeability (**A**) Flow cytometry analysis of TREM2 knockdown efficiency in ascites-derived macrophages (siTREM2 vs. negative control [NC]; *n* = 3 biological replicates). (**B**) TRITC-dextran permeability assay in HUVECs cocultured with ascites macrophages treated with siTREM2 or NC (*n* = 3 biological replicates). (**C**,** D**) Western blot analysis (**C**) and quantification (**D**) of p-VE-cadherin (Tyr658) in HUVECs cocultured with ascites macrophages with siTREM2 or NC (*n* = 3 biological replicates). β-actin was used as a loading control. (**E**) TRITC-dextran flux in C166 endothelial cells treated with sTREM2 ± anti-sTREM2 scFv (*n* = 4 biological replicates). (**F**) Schematic diagram of in vivo anti-sTREM2 scFv treatment protocol. (**G**) Peritoneal vascular permeability in mice intraperitoneally treated with sTREM2 (200 μg/dose) ± anti-sTREM2 scFv (200 μg/dose; *n* = 6 mice per group). Data were shown as mean ± SEM. *P < 0.05; **P < 0.01; ***P < 0.001; ****P < 0.0001. Exact *p* values are provided in Appendix Table S2. Two-tailed Student’s *t*-test for (**A**, **B**, **D**); one-way ANOVA for (**E**, **G**). Source data are available online for this figure.

To evaluate whether targeting sTREM2 could mitigate OC ascites formation and disease progression, we first depleted sTREM2 in patient-derived ascitic supernatants (Fig. 6A). Application of the treated supernatant to endothelial cells significantly reduced both TRITC-dextran leakage and VE-cadherin phosphorylation relative to supernatant treated with control IgG (Fig. 6B–D). Concordantly, knockdown of TREM2 in ascites-derived macrophages diminished their capacity to disrupt endothelial barrier function (Fig. EV5A–D). Next, we assessed whether sTREM2 neutralization inhibits vascular leakage in vivo using a previously reported single-chain fragment variable (scFv) antibody that specifically targets murine TREM2 extracellular domain (Chen et al, 2023). Permeability assays confirmed that scFv treatment attenuated sTREM2-induced endothelial leakage (Fig. EV5E). Crucially, in vivo studies revealed that mice receiving scFv treatment exhibited significantly reduced vascular permeability relative to control mice (Fig. EV5F,G).

Finally, in the ID8 murine model, administration of scFv antibody, initiated three weeks after tumor inoculation, significantly reduced both ascites volume and tumor burden relative to control cohorts (Fig. 6E–H). Notably, scFv-treated mice showed decreased levels of ascitic sTREM2 (Fig. 6I) and sTREM2 levels remained positively correlated with ascites volume (Fig. 6J). Furthermore, immunofluorescence analysis of metastatic tissues revealed diminished VE-cadherin phosphorylation in scFv-treated mice (Fig. 6K,L). In conclusion, these results demonstrate that sTREM2 blockade effectively alleviates vascular leakage and malignant ascites formation.

## Discussion

Our study identifies sTREM2 as a critical mediator of vascular hyperpermeability and malignant ascites formation in OC. We demonstrate a direct correlation between ascitic sTREM2 levels and OC severity. Mechanistically, sTREM2 interacts with cell-surface NCL and activates the AKT/eNOS signaling pathway, thereby contributing to endothelial barrier disruption. Importantly, pharmacological targeting of sTREM2 effectively attenuated vascular leakage and ascites production. These findings establish sTREM2 as a central mechanistic driver of ascites pathogenesis, highlighting its potential as a therapeutic target for OC.

TREM2 has been extensively characterized across diverse disease models, exhibiting pronounced context-dependent functionality influenced by disease type, tissue microenvironment, and receptor isoforms (Benitez and Cruchaga 2013; Poliani et al, 2015; Deczkowska et al, 2020; Nakamura and Smyth 2020; Hou et al, 2021; Ming et al, 2024). For instance, in oncological contexts, TREM2 exhibits tumor-suppressive activity in central nervous system malignancies but paradoxical oncogenic activity in peripheral cancers (Zhong et al, 2024). Therefore, the functions of TREM2 cannot be generalized across disease models and require context-specific investigation. Consistent with prior studies of OC (Binnewies et al, 2021), our work confirms the tumor-promoting role of TREM2 in this malignancy. However, in contrast to previous reports that primarily focused on TREM2’s immunosuppressive effects within the myeloid compartment, our study reveals a vascular mechanism whereby sTREM2 fuels disease progression. This dual role—immune suppression via tumor-associated macrophages and barrier disruption via endothelial cells—positions TREM2 as a multifaceted therapeutic target in OC. Future studies should explore whether sTREM2 blockade synergizes with existing anti-angiogenic or immunotherapeutic regimens to improve clinical outcomes.

sTREM2, generated via receptor shedding or alternative splicing, is detectable in plasma and cerebrospinal fluid. Consequently, sTREM2 has been validated as a diagnostic and prognostic biomarker for multiple pathologies, including non-alcoholic fatty liver disease, Alzheimer’s disease, and primary angiitis of the central nervous system (Brown and St George-Hyslop 2021; Filipello et al, 2022; Hendrikx et al, 2022; Guo et al, 2022; Zhao et al, 2022; Zhang et al, 2023). In this study, we detected sTREM2 in ascitic fluid and identified a significant correlation between sTREM2 levels and ascites volume. This finding opens new avenues for OC and ascites research. Mechanistically, how sTREM2 contributes to diverse pathologies is still enigmatic. Current research has primarily focused on its immunomodulatory roles (Zhong et al, 2019; Zhou et al, 2023; Qin et al, 2024; Cui et al, 2025; Gu et al, 2025), while its effects on non-immune components of the microenvironment—particularly the vasculature—remain largely unexplored, representing a major knowledge gap. Addressing this blind spot, we identified that sTREM2 targets endothelial cells through NCL binding, leading to disruption of barrier integrity via mechanisms involving the AKT/eNOS pathway. This discovery establishes the sTREM2-endothelial axis as a novel regulatory mechanism in tumor microenvironments and identifies sTREM2 as a new functional ligand for NCL.

NCL is a multifunctional protein widely expressed in eukaryotic cells. Although predominantly localized to the nucleolus, NCL dynamically shuttles to the nucleoplasm, cytoplasm, and plasma membrane, with its subcellular translocation dictating functional specificity (Jia et al, 2017; Tonello et al, 2022). Notably, membrane-associated NCL in endothelial cells has been previously shown to modulate angiogenesis and tumor lymphangiogenesis by binding to endostatin (Zhuo et al, 2010; Song et al, 2012). Furthermore, endothelial NCL acts as a receptor for secreted ADAMTS5 through its RBDs, thereby regulating apoptosis (Kirman et al, 2022). Critically, our study identifies NCL as the functional receptor for sTREM2 in endothelial cells, positioning NCL as a key regulator of vascular permeability in this context. However, several limitations should be noted. First, direct evidence of sTREM2-dependent NCL activation in patient tissues remains lacking. While p-eNOS staining in ovarian cancer vasculature provides indirect support, the sTREM2–NCL interaction could not be confirmed owing to limited tissue availability and technical constraints. Future studies using fresh ascites or tissues for co-immunoprecipitation or proximity ligation assays will be needed. Second, although we identified the RBD domain of NCL as essential for sTREM2 binding, the precise structural determinants remain to be further characterized. Third, our findings in OC models require validation in other ascites-associated malignancies. Fourth, the multifunctional nature of NCL warrants investigation of potential off-target effects of sTREM2 blockade. Despite these limitations, our work fundamentally advances the understanding of barrier dysfunction by identifying the sTREM2-NCL axis as a novel pathogenic mechanism.

Managing malignant ascites remains a significant challenge in OC. Increased vascular permeability is a key pathophysiological driver, with cellular (e.g., tumor cells and macrophages) and acellular (e.g., VEGF) components promoting fluid generation (Bekes et al, 2016; Ford et al, 2020; Rickard et al, 2021; Monavarian et al, 2022; Konstantinopoulos and Matulonis 2023). Although primary disease treatment and VEGF inhibitors (e.g., bevacizumab) can reduce ascites, recurrence and treatment-related adverse effects are frequently observed (Monk et al, 2013; Monk et al, 2016). Therefore, identifying alternative targets for blocking vascular leakage is imperative. Our prior work has implicated macrophage blockade, particularly of the VLA4^hi^ and PLIN2^hi^ subsets, in ascites pathogenesis (Moughon et al, 2015; Zhang et al, 2021; Luo et al, 2025). However, interventions targeting these cellular pathways have been associated with either significant toxicity or limited efficacy, likely due to the complexity and heterogeneity of the tumor microenvironment. This study identifies a soluble mediator, sTREM2, as a critical driver of malignant ascites. Therapeutic targeting of sTREM2 may offer distinct pharmacological advantages relative to targeting cells or membrane-bound proteins. These advantages include enhanced bioavailability within fluid compartments, reduced potential for cellular toxicity, favorable pharmacokinetic properties, and the ability to monitor circulating sTREM2 levels as both a pharmacodynamic biomarker and an indicator of therapeutic response.

Significantly, administration of an sTREM2-neutralizing scFv antibody attenuated ascites generation in preclinical models, demonstrating therapeutic efficacy. The compact structure of the scFv antibody enhances tissue penetration and reduces immunogenicity. Its transient systemic exposure enables precise dosing control—offering safety advantages over persistent biologics—and facilitates streamlined prokaryotic production and engineering (Tiller and Tessier 2015; Lou and Cao 2022). Critically, its therapeutic value is amplified by functional specificity. Targeting sTREM2 disrupts tumor-associated macrophage-endothelial crosstalk with precision, potentially circumventing the systemic effects of VEGF inhibitors. This novel mechanism offers an alternative strategy beyond anti-VEGF therapies. Initial in vivo studies revealed no detectable toxicity, supporting a favorable preliminary safety profile. While these findings provide robust preclinical proof-of-concept for targeting sTREM2 in OC, comprehensive kinetic studies and evaluation in combination regimens remain essential prior to clinical translation.

Collectively, this study identifies the sTREM2-NCL-AKT/eNOS axis as a mechanistic pathway involved in ascites pathogenesis and proposes a novel therapeutic strategy. Targeting sTREM2 represents a promising approach for the precise treatment of malignant ascites, potentially advancing tumor microenvironment therapies centered on immune-vascular interplay.

## Methods

**Table:** Reagents and tools table

Reagent/resource	Reference or source	Identifier or catalog number
**Experimental models**		
C57BL/6J	GemPharmatech, China	
*Trem2* ^*fl/fl*^ *Lyz2* ^*Cre*^	Ref: Ming et al, 2024	
**Recombinant DNA**		
NCL-FL	Genomeditech, China	Appendix Table S1
NCLΔGAR	Genomeditech, China	Appendix Table S1
NCLΔC	Genomeditech, China	Appendix Table S1
NCLΔN	Genomeditech, China	Appendix Table S1
OE-sTREM2	Genomeditech, China	Appendix Table S1
OE-TREM2	Genechem, China	Appendix Table S1
Sh-TREM2	Igebio, China	Appendix Table S1
**Antibodies**		
anti-TREM2	Abcam, USA	ab223684
Rabbit anti-Goat DyLight 649-conjugated secondary antibody	Abbkine, China	A23630
anti-CD68	CST, USA	76437
Donkey anti-Rabbit DyLight 488-conjugated secondary antibody	Boster, China	BA1146
anti-CD45-BV605	BioLegend, USA	304042
anti-CD68-PE	BioLegend, USA	333808
anti-TREM2-APC	R&D, USA	FAB17291A
anti-phospho-VE-cadherin (Tyr658)	Thermo Fisher, USA	44-1144 G
anti-VE-cadherin	Santa Cruz, USA	(F-8): sc-9989
anti-TREM2	Thermo Fisher, USA	PA5119690
anti-HA	Proteintech, China	51064-2-AP
anti-TREM2	R&D Systems, USA	AF1729
anti-CD31	R&D Systems, USA	AF3628
anti-NCL	CST, USA	14574S
anti-β-actin	CST, USA	4970S
anti-ATP1A1	Thermo Fisher, USA	MA3-928
anti-Lamin B1	Affinity, China	AF5161-50
anti-biotin	CST, USA	7075S
anti-IgG(R)	CST, USA	3900S
anti-IgG(M)	CST, USA	5415S
anti-Flag(R)	Proteintech, China	20543-1-AP
anti-Flag(M)	ABclonal, China	AE005
anti-eNOS	CST, USA	32027 T
anti-phospho-eNOS (Ser1177)	CST, USA	9570S
anti-AKT	CST, USA	4691 s
anti-phospho-AKT (Ser473)	CST, USA	4046 s
CD45-FITC	BioLegend, USA	304006
CD45-BV711	BioLegend, USA	304049
CD14-PE	BioLegend, USA	325606
**Oligonucleotides and other sequence-based reagents**		
siTREM2	Ribobio, China	Appendix Table S1
siNCL	Ribobio, China	Appendix Table S1
**Chemicals, Enzymes and other reagents**		
human sTREM2-His	Sino Biological, China	11084-H08H
Mouse sTREM2-His	Sino Biological, China	50149-M08H
Biotinylated human sTREM2-His	Sino Biological, China	11084-H49H-B
Human NCL recombinant protein	OriGene, USA	TP319082
Anti-sTREM2 scFv-His	Kairui Biotech, China Ref: Chen et al, 2023	WO2020123664A
Mouse sTREM2-Flag	Tsingke, China	UniProtKB Q99NH8,
L-NAME	Selleck, USA	S2877-100mg
MK2206	Proteintech, China	CM00917-1MG
Clodronate Liposomes	Yeasen, China	40337ES10
Protein A/G agarose beads	Santa Cruz Biotechnology, USA	sc-2003
TRITC-dextran	Sigma-Aldrich, USA	T1162
Pierce™ Streptavidin Agarose Beads	Thermo Fisher Scientific, USA	20347
Blasticidin	MCE, USA	HY-103401
Puromycin	BioFroxx, Germany	1299MG025
Red Blood Cell Lysis Buffer	Beyotime, China	C3702
eBioscience Fixation/Permeabilization Diluent	Invitrogen, USA	00-5223-56
Lipofectamine 3000 transfection reagent	Thermo Fisher Scientific, USA	L3000-015
Lipofectamine LTX with Plus Reagent	Thermo Fisher Scientific, USA	15338100
Lipofectamine RNAiMAX	Thermo Fisher Scientific, USA	13778030
LIVE/DEAD Fixable 808/876	Thermo Fisher Scientific, USA	L34982
**Software**		
ImageJ (version 1.51j8)	https://imagej.net/ij/	
GraphPad Prism (version 8.2.1)	https://www.graphpad.com/features	
OriginPro (version 2022 and 2025)	https://www.originlab.com/	
R (version 3.6.0)	https://www.r-project.org/	
CytExpert (version 2.3)	https://www.beckman.hk/flow-cytometry/research-flow-cytometers/cytoflex/software	
**Other**		
Human TREM2 ELISA kits	Multi Sciences, China	EK1226-96
Human TREM2 ELISA kits	Abcam, USA	ab224881
Mouse TREM2 ELISA kits	Jonlnbio, China	JL20435-96T
Plasma Membrane/Protein Isolation and Cell Fractionation Kit	Invent, USA	SM-005

### Sex as a biological variable

As OC occurs exclusively in females, this study utilized samples and models from female subjects only.

### Bioinformatics analysis

We validated TREM2 expression patterns using bulk RNA-seq data from the TCGA ovarian cancer cohort and single-cell RNA-seq datasets (accessions: HRA002362 and E-MTAB-8107). From the HRA002362 dataset, we isolated cells from primary tumors, metastatic lesions, malignant ascites, and non-malignant ovarian tissues. Differential gene expression analysis across these sampling sites was performed using the FindMarkers function in Seurat with a Wilcoxon rank-sum test (adjusted *p* value <0.05, fold change >1.5). TREM2 expression levels across distinct cell types were visualized using the VlnPlot function in Seurat. Similarly, we assessed differential TREM2 expression between tumor and normal samples in the E-MTAB-8107 dataset. Furthermore, we evaluated the association between TREM2 expression and patient prognosis using the TCGA ovarian cancer dataset. Optimal cut-points for TREM2 expression were determined using the surv_cutpoint function (survminer package), and Kaplan–Meier survival curves were subsequently generated with the ggsurvplot function. Statistical significance was assessed using the log-rank test (*p* value <0.05).

### Human specimen collection

The collection of all samples (including tissues and peritoneal fluid) complied with ethical guidelines, and written informed consent was obtained from patients (Approval numbers: 2022-K319-1 and 2025-011). Tissue and peritoneal fluid samples were collected from specimens obtained during routine surgical procedures. No additional surgical intervention or procedural modification was performed for research purposes. All samples were anonymized prior to analysis.

### Inclusion and exclusion criteria for tissue donors

Ovarian tissue samples were obtained from female patients aged ≥18 years. The ovarian cancer group comprised patients with a histopathologically confirmed diagnosis of ovarian cancer from whom sufficient tumor tissue (>100 mg) was collected intraoperatively. The control group consisted of female patients aged ≥18 years who underwent gynecologic surgery for non-ovarian malignant indications, such as cervical cancer, endometrial cancer, severe endometriosis, benign ovarian cysts, or prophylactic surgery in high-risk individuals. Control ovarian tissues were obtained during surgery and were confirmed to be free of malignant or premalignant lesions by postoperative histopathological examination when available, or were considered macroscopically normal by the operating surgeons. Patients were excluded if they had received neoadjuvant chemotherapy or radiotherapy within 3 months prior to surgery, were pregnant or lactating, or had incomplete clinical data.

### Inclusion and exclusion criteria for peritoneal fluid donors

Peritoneal fluid samples were obtained from female patients aged ≥18 years. The ovarian cancer group comprised patients with histopathologically confirmed ovarian cancer accompanied by ascites, from whom malignant ascites (>5 mL) was collected. The control group consisted of patients undergoing surgery for benign conditions, such as uterine fibroids, in whom intraoperative peritoneal lavage with normal saline was routinely performed, and lavage fluid (>5 mL) was collected. Patients were excluded if they had received neoadjuvant chemotherapy or radiotherapy within 3 months prior to surgery; if they had other primary malignant tumors or ascites suspected to be caused by non-ovarian malignancies (ovarian cancer group); if they had intraoperative or postoperative evidence of ovarian precancerous lesions or malignant lesions or other primary peritoneal malignancies (control group); if they were pregnant or lactating; or if they had incomplete clinical data.

### Cell lines and cell culture

HUVECs were obtained from ScienCell (USA); murine endothelial C166 cells were purchased from ATCC; THP-1 human monocyte cell line was generously provided by Professor Dong-Ming Kuang (Sun Yat-sen University); and ID8 mouse ovarian epithelial cancer cell line was purchased from ZQXZBIO (China). Immortalized bone marrow-derived macrophages (iBMDM) were purchased from Fuheng (China) and were on a syngeneic C57BL/6 background. All cell lines were maintained at 37 °C in a humidified 5% CO₂ atmosphere. HUVECs were cultured in Endothelial Cell Medium (ScienCell, USA) supplemented with 5% fetal bovine serum, 1% endothelial cell growth supplement, and 1% penicillin/streptomycin (100 U/mL penicillin and 100 μg/mL streptomycin). THP-1 cells were cultured in RPMI 1640 medium (Gibco, USA) containing 10% fetal bovine serum (Procell, China) and 1% penicillin/streptomycin (Beyotime, China). Prior to experiments, THP-1 cells were treated with 0.1 μg/mL PMA (Solarbio, China) for 24 h to induce macrophage differentiation. ID8, C166, and RAW264.7 cells were maintained in DMEM (Gibco, USA) supplemented with 10% fetal bovine serum and 1% penicillin/streptomycin. iBMDM cells were cultured in specifically formulated medium (Fuheng, China). All cell lines were authenticated by short tandem repeat (STR) profiling and were routinely tested for mycoplasma contamination.

### Transfection and establishment of stable cell lines

(1) Stable TREM2-knockdown and TREM2-overexpressing iBMDM cells were generated via lentiviral transduction. Cells were transduced with lentiviral vectors carrying the respective constructs (Ige Bio and Genechem, China) at a multiplicity of infection (MOI) of 25 in the presence of 8 μg/mL polybrene. Starting 48 h post-transduction, stable transformants were selected with 5 μg/mL puromycin for two weeks. Transduction efficiency was monitored by fluorescence microscopy, and successful TREM2 knockdown or overexpression was confirmed by Western blot analysis. (2) To generate sTREM2-overexpressing macrophages, THP-1 were transduced with a lentiviral vector encoding human sTREM2 (Genomeditech, China) at an MOI of 50 in the presence of 8 μg/mL polybrene. Starting 48 h post-transduction, cells were selected with 5 μg/mL blasticidin for two weeks. Transduction efficiency was monitored by fluorescence microscopy, and sTREM2 protein levels were confirmed by ELISA.

### Permeability assay

Endothelial barrier integrity was assessed using a Transwell-based permeability assay. A confluent endothelial monolayer was established by seeding HUVECs or murine C166 cells (1 × 10⁵ cells/insert) into the upper chambers of 24-well Transwell plates (0.4 μm pore size; Corning, USA). After monolayer formation, macrophages (5 × 10^4^ cells/well) or experimental treatments were applied to the endothelial monolayer for 12 h. Cells were then washed twice with PBS (100 μL per wash), followed by the addition of 500 μL complete medium to the lower chamber and 200 μL TRITC-dextran (2 mg/mL, 70 kDa; Sigma-Aldrich, USA) to the upper chamber. After 2 h of incubation at 37 °C, fluid from the lower chamber was collected, and fluorescence intensity was measured using a PerkinElmer EnVision plate reader (USA).

### Real-time monitoring of cell interaction

The xCELLigence RTCA DP system (ACEA Biosciences, USA) was used to monitor cellular interactions in real time. Endothelial cells were seeded into E-plates at a density of 2 × 10⁴ cells per well and allowed to form a confluent monolayer. Macrophages (1 × 10⁴ cells per well) or indicated treatments were then added, and coculture was continued with continuous monitoring of Cell Index (CI) values for 12 h. The CI is a parameter measured by the RTCA system that reflects changes in the impedance of an adherent cell layer. A decrease in CI reflects disruption of cell–cell junctions and corresponds to increased endothelial permeability. The normalized CI was calculated by normalizing each time point to the CI value immediately after the addition of macrophages or treatments.

### Flow cytometry

Ascites samples were filtered through a 70-μm strainer to remove cellular aggregates, followed by lysis of red blood cells using red blood cell lysis buffer. After centrifugation, the cell pellet was washed with PBS and stained for flow cytometric analysis with the following reagents: LIVE/DEAD Fixable Near-IR viability dye (808/876 nm; Thermo Fisher, USA, cat# L34982), anti-human CD45-BV605 (BioLegend, USA, cat# 304042), anti-human CD68-PE (BioLegend, USA, cat# 333808), and anti-human TREM2-APC (R&D Systems, USA, cat# FAB17291A).

### Fluorescence-activated cell sorting

To isolate macrophages from ascitic fluid, human ascites samples were first centrifuged, and erythrocytes were subsequently lysed. Cells were then stained with the following fluorescently-conjugated antibodies: anti-human CD45-FITC (BioLegend, USA, cat# 304006), anti-human CD45-BV711 (BioLegend, USA, cat# 304049), and anti-human CD14-PE (BioLegend, USA, cat# 325606). CD14^+^ macrophages were subsequently isolated by fluorescence-activated cell sorting using a BD FACSAria Fusion system (BD Biosciences, USA).

### Enzyme-linked immunosorbent assay

The concentration of sTREM2 was quantified using commercial ELISA kits under standardized conditions. For human samples, sTREM2 levels were measured in both ascitic supernatants and culture supernatants of ascites-derived macrophages using human-specific ELISA kits from Abcam (USA) and Multi Sciences (China). Murine sTREM2 levels in ascitic fluid from ovarian cancer mouse models were quantified using a mouse-specific ELISA kit from Jonlnbio (China). All procedures were strictly followed by the manufacturer’s protocols. Briefly, standard curves were established using the provided calibrators, followed by sample preparation and optical density measurement. The sTREM2 concentrations were interpolated from the standard curves.

### Western blot analysis

Total cellular proteins were extracted using Western & Immunoprecipitation lysis buffer (Beyotime, China). Membrane, cytoplasmic, and nuclear fractions were isolated using a Protein Extraction and Cell Fractionation Kit (Invent, USA). Protein concentrations were quantified using a bicinchoninic acid assay kit (Beyotime, China). Equal amounts of protein lysates were separated by SDS-PAGE (7.5% or 10% gels) and transferred onto polyvinylidene fluoride membranes (Merck Millipore, Germany). After blocking with 5% non-fat milk or bovine serum albumin, membranes were incubated with primary antibodies overnight at 4 °C, followed by incubation with the corresponding horseradish peroxidase-conjugated secondary antibodies (Proteintech, China) at room temperature. Protein bands were visualized using enhanced chemiluminescence substrate and detected with a ChemiDoc XRS imaging system (Bio-Rad, USA). Appropriate loading controls were used for each fraction. Western blot band intensities were quantified using ImageJ software (version 1.51j8; National Institutes of Health, USA).

### Immunofluorescence staining

(1) HUVECs were cultured on confocal dishes and treated with sTREM2 or other indicated conditions. Cells were fixed with 4% paraformaldehyde, permeabilized with 0.1% Triton X-100 (Solarbio, China) in PBS, and blocked with 1% bovine serum albumin (Solarbio, China) in PBS for 1 h at room temperature. After incubation with an anti-p-VE-cadherin (Tyr658; Thermo Fisher, USA, cat# 44-1144 G) antibody overnight at 4 °C, samples were washed and incubated with either DyLight 488-conjugated goat anti-rabbit (Abbkine, cat# A23220, China) or DyLight 649-conjugated goat anti-rabbit (Abbkine, cat# A23620, China) secondary antibodies for 1 h at room temperature. (2) Human ovarian tissue samples were fixed in 4% paraformaldehyde, embedded in paraffin, sectioned, deparaffinized, and subjected to antigen retrieval. Sections were blocked and permeabilized with PBS containing 5% BSA and 0.3% Triton X-100 for 1 h, then incubated overnight at 4 °C with the following primary antibodies: anti-CD68 (Cell Signaling Technology, USA, cat# 76437) and anti-TREM2 (Abcam, UK, cat# ab223684). Murine tissue samples were embedded in optimal cutting temperature compound (Sakura Finetek, USA) immediately after collection. Cryosections were prepared and processed following optimal cutting temperature compound removal using the same immunohistochemical protocol as for human tissues. Primary antibodies used were anti-CD31 (R&D Systems, USA, cat# AF3628) and anti-p-VE-cadherin (Tyr658; Thermo Fisher, USA, cat# 44-1144 G). All sections were then incubated with appropriate secondary antibodies: donkey anti-rabbit IgG DyLight 488 (Boster, China, cat# BA1146) and rabbit anti-goat IgG DyLight 649 (Abbkine, China, cat# A23630). Nuclei were counterstained with DAPI (Solarbio, China) for 10 min at room temperature. Images were acquired using a confocal microscope (Zeiss, Germany). Immunofluorescence signal intensity was quantified using ImageJ software.

### Streptavidin pull-down and mass spectrometry

Cells were washed three times with PBS (5 min each) and then lysed to prepare whole-cell lysates or subcellular fractions, followed by incubation with biotinylated recombinant human TREM2 protein (Sino Biological, China; cat# 11084-H49H-B) for 6 h at 4 °C. The biotinylated sTREM2 and its interacting proteins were then pulled down from the lysates or membrane fractions using Pierce™ Streptavidin Agarose Beads (Thermo Fisher Scientific, USA; cat# 20347). The bound protein complexes were eluted, separated by SDS-PAGE, and analyzed by liquid chromatography-tandem mass spectrometry (APTBIO, China).

### Co-immunoprecipitation analysis

Co-immunoprecipitation assays were conducted to investigate protein–protein interactions. Cells were lysed on ice for 30 min using Western & Immunoprecipitation Lysis Buffer (Beyotime, China) supplemented with protease and phosphatase inhibitor cocktails (Beyotime, China). Lysates were then centrifuged at 12,000×*g* for 20 min at 4 °C. The supernatants were incubated overnight at 4 °C with the indicated primary antibodies: anti-nucleolin (NCL; CST, USA; cat# 14574S), anti-biotin (CST, USA; cat# 7075S), anti-Flag (Proteintech, China, cat# 20543-1-AP; ABclonal, China, cat# AE005), or normal rabbit/mouse IgG control antibodies (CST, USA; cat# 3900S, 5415S). Protein A/G agarose beads (Santa Cruz Biotechnology, USA) were added to the lysate-antibody mixtures and incubated for 2 h at 4 °C with gentle rotation. The beads were washed three times with ice-cold PBS, and bound proteins were eluted by boiling in 2× SDS loading buffer at 95 °C for 5 min. The eluted proteins were then separated by SDS-PAGE and analyzed by Western blotting.

### Bio-layer interferometry

Bio-layer interferometry assays were performed using an Octet R8 system (Sartorius, Germany). All assays were conducted at 25 °C in PBS supplemented with 0.02% (v/v) Tween-20 (pH 7.4). Biotinylated sTREM2 was freshly prepared and immobilized onto streptavidin biosensors, and a series of concentrations of NCL were analyzed to assess binding kinetics, generating real-time binding curves.

### Molecular docking

To elucidate the potential interaction between sTREM2 (PDB: 1FJ7) and NCL (PDB: 9CB5), we performed protein–protein interaction simulations using the Prime module of the Schrödinger suite. The computational modeling revealed a favorable binding interface between sTREM2 and NCL, suggesting a stable interaction. Specifically, Ser35 and Thr94 of sTREM2 were predicted to form hydrogen bonds with Arg342 of NCL, while Asp87 of sTREM2 was predicted to form a hydrogen bond with Asn308 of NCL. These hydrogen-bonding interactions may contribute to the stabilization of the sTREM2-NCL complex, providing structural insights into their potential functional association.

### Depletion of sTREM2 in vitro

To deplete the target protein, conditioned medium was incubated with specific antibodies—including anti-TREM2 (Thermo Fisher Scientific, USA; cat# PA5119690) or anti-HA (Proteintech, China; cat# 51064-2-AP)—at a concentration of 10 μg/mL for 2 h at 37 °C or 12 h at 4 °C. Subsequently, 100 μL of Protein A/G agarose beads were added, and the mixture was incubated for 6 h at 4 °C with gentle rotation. The immunocomplexes were pelleted by centrifugation at 400×*g* for 5 min, and the supernatants were collected and analyzed by ELISA.

### Recombinant proteins and scFv antibody acquisition

All recombinant proteins and scFv antibodies were commercially sourced, including: human sTREM2-His (Sino Biological, China; cat# 11084-H08H), mouse sTREM2-His (Sino Biological; cat# 50149-M08H), and biotinylated human sTREM2-His (Sino Biological; cat# 11084-H49H-B); human NCL recombinant protein (OriGene, USA; cat# TP319082); anti-sTREM2 scFv-His (Kairui Biotech, China; patent WO2020123664A1); and mouse sTREM2-Flag (Tsingke, China; UniProtKB Q99NH8, residues 19-171). The scFv antibody is structured with a G_4_S linker connecting the anti-TREM2_VH_ and anti-TREM2_VL_, and the antibody sequence is based on patent WO2020123664A (Chen et al, 2023).

### siRNA and plasmid transfection

Gene silencing and overexpression were achieved using Lipofectamine-based transfection systems following the manufacturer’s protocols. For siRNA-mediated knockdown, Lipofectamine RNAiMAX (Thermo Fisher Scientific, USA) was employed to transfect cells with siRNAs. For plasmid transfection, Lipofectamine LTX transfection reagent and Lipofectamine 3000 transfection reagent (Thermo Fisher Scientific, USA) were used. Briefly, cells were seeded in six-well plates and transfected for 8 h with complexes formed between siRNA and Lipofectamine RNAiMAX or between plasmid DNA and Lipofectamine 3000. The transfected cells were then cultured in complete medium for 24 or 48 h prior to functional assays. The sequences of siRNAs and the constructs of plasmids are provided in Appendix Table S1.

### Animals

All animals were housed under specific pathogen-free conditions at the Guangdong Provincial Engineering Research Center of Molecular Imaging. Female C57BL/6 WT and *Trem2*^*fl/fl*^*Lyz2*^*Cre*^ mice on a C57BL/6 background were used in this study (Approval numbers: 00353 00585 and 00702). The *Trem2*^*fl/fl*^*Lyz2*^*Cre*^ mice were generously provided by Professor Xi Huang (Sun Yat-sen University). Specifically, this model was generated by crossing mice carrying a floxed *Trem2* allele (*Trem2*^*fl/fl*^) with *Lyz2*^*Cre*^ transgenic mice, in which Cre recombinase expression is driven by the lysozyme M promoter to achieve specific deletion in myeloid cells, including macrophages (Ming et al, 2024).

### Permeability assay in vivo models

(1) To evaluate the effect of sTREM2, female *Trem2*^*fl/fl*^*Lyz2*^*Cre*^ mice (6–8 weeks old) were randomly assigned to experimental groups and administered intraperitoneal injections (100 μL) of either PBS (control) or recombinant sTREM2 (200 μg/mouse/dose) every other day for four doses. On day 9, mice were intravenously injected with 100 μL TRITC-dextran (10 mg/mL, 70 kDa, Sigma-Aldrich, USA) via the tail vein. After 30 min of circulation, peritoneal lavage was performed using 2.5 mL ice-cold PBS. The lavage fluid was centrifuged, and the fluorescence intensity of the supernatant was measured. (2) To evaluate macrophage-specific TREM2 function, WT female C57BL/6 mice (6–8 weeks old) were randomly assigned to experimental groups and injected intraperitoneally with clodronate liposomes (200 μL/mouse/dose; Yeasen, China) on days 1 and 3 for macrophage depletion. On day 5, 1 × 10⁶ TREM2-overexpressing or control macrophages (in 100 μL PBS) were transferred intraperitoneally. On day 8, TRITC-dextran was injected intravenously, followed by peritoneal lavage 30 min later as described above. (3) For L-NAME treatment, *Trem2*^*fl/fl*^*Lyz2*^*Cre*^ mice were randomly assigned and divided into four groups receiving: PBS, L-NAME (625 μg/mouse/dose; Selleck, USA), sTREM2 (200 μg/mouse/dose), or a combination of sTREM2 (200 μg/mouse/dose) and L-NAME (625 μg/mouse/dose; Selleck, USA). sTREM2 was administered every other day for four doses, and L-NAME was intraperitoneally injected 30 min prior to each sTREM2 administration to inhibit nitric oxide production. Vascular permeability was assessed on day 9 using the method described above. (4) For scFv Treatment, *Trem2*^*fl/fl*^*Lyz2*^*Cre*^ mice were randomly assigned and divided into three groups: PBS, sTREM2 (200 μg/mouse/dose), or a combination of sTREM2 and scFv (200 μg/mouse/dose) every other day for four doses. Vascular permeability was evaluated on day 9. Investigators were blinded to group assignment throughout experiments and data analyses.

### Establishment of OC mouse models

(1) To assess sTREM2 function, female *Trem2*^*fl/fl*^*Lyz2*^*Cre*^ mice (6–8 weeks old) were randomly assigned to experimental groups and intraperitoneally inoculated with 1 × 10⁶ ID8 cells on day 1. Subsequently, they were treated weekly via intraperitoneal injection with either recombinant sTREM2 (200 μg/mouse/dose in 100 μL PBS) or PBS on days 1, 8, 15, and 22. (2) To assess macrophage-specific TREM2 function, age-matched WT C57BL/6 females were co-injected intraperitoneally with 1 × 10⁶ ID8 cells and 1 × 10⁶ iBMDM cells (TREM2-overexpressing, knockdown, or control) on day 1. (3) To evaluate the therapeutic potential of sTREM2-targeting scFv, WT mice bearing established ID8 tumors (inoculated with 1 × 10⁶ cells on day 1) were randomly assigned to experimental groups and intraperitoneally injected weekly with scFv (200 μg/mouse/dose in 100 μL PBS) or PBS starting on day 21. At the experimental endpoint, the animals were euthanized; mesenteric tissues, ascites, and tumor tissues were collected. Ascites cells and ascitic supernatants were collected by centrifugation. Ascites volume, tumor burden, and ascitic sTREM2 were assessed. Investigators were blinded to group assignment throughout experiments and data analyses.

### Statistics

All quantitative data are expressed as mean ± SEM from at least three independent biological replicates. Statistical analyses were performed using GraphPad Prism (version 8.2.1). Comparisons between two groups were performed using two-tailed Student’s *t*-tests, while comparisons among more than two groups were conducted using one-way ANOVA. To evaluate the effects of two independent variables, two-way ANOVA was employed. Correlation analyses were conducted using Pearson’s correlation coefficient. A *p* value of less than 0.05 (*p* < 0.05) was considered statistically significant. Immunofluorescence and Western blot images were quantified using ImageJ software (version 1.51j8; National Institutes of Health, USA).

### Study approval

The study was approved by the Animal Ethics Committee of the Fifth Affiliated Hospital of Sun Yat-Sen University (Approval numbers: 00353, 00585, and 00702). All animal experiments were performed in accordance with the guidelines of the Animal Care and Use Committee of Sun Yat-Sen University. All human sample collections were performed in compliance with protocols (Approval Nos.: 2022-K319-1 and 2025-011) approved by the Ethics Committee of the Fifth Affiliated Hospital, Sun Yat-sen University.

## Supplementary information

Appendix

Peer Review File

Source data Fig. 1

Source data Fig. 2

Source data Fig. 3

Source data Fig. 4

Source data Fig. 5

Source data Fig. 6

Figure EV1 Source Data

Figure EV2 Source Data

Figure EV3 Source Data

Figure EV4 Source Data

Figure EV5 Source Data

Expanded View Figures

## Data Availability

All data used in our study are publicly available. The raw RNA-seq data have been deposited in the Sequence Read Archive (SRA) under the accession number PRJNA1332085 and are accessible via the URL: https://www.ncbi.nlm.nih.gov/sra/?term=PRJNA1332085. The source data of this paper are collected in the following database record: biostudies:S-SCDT-10_1038-S44321-026-00452-2.
