# Supplementary material for: Soluble TREM2 engages cell-surface nucleolin to drive vascular permeability and malignant ascites in ovarian cancer
Source: EMBO Mol Med. 2026 May 26;18(7):2667–90. doi: 10.1038/s44321-026-00452-2 (PMC13365401; doi:10.1038/s44321-026-00452-2)
Supplement: Supplementary file 1 — Appendix [file 44321_2026_452_MOESM1_ESM.pdf]

Appendix

Appendix Fig. S1. Validation of NCL as an sTREM2 receptor in endothelial cells. 2

Appendix Table S1. Sequences for All siRNAs and Plasmid Constructs 3

Appendix Table S2. Exact p-values 4

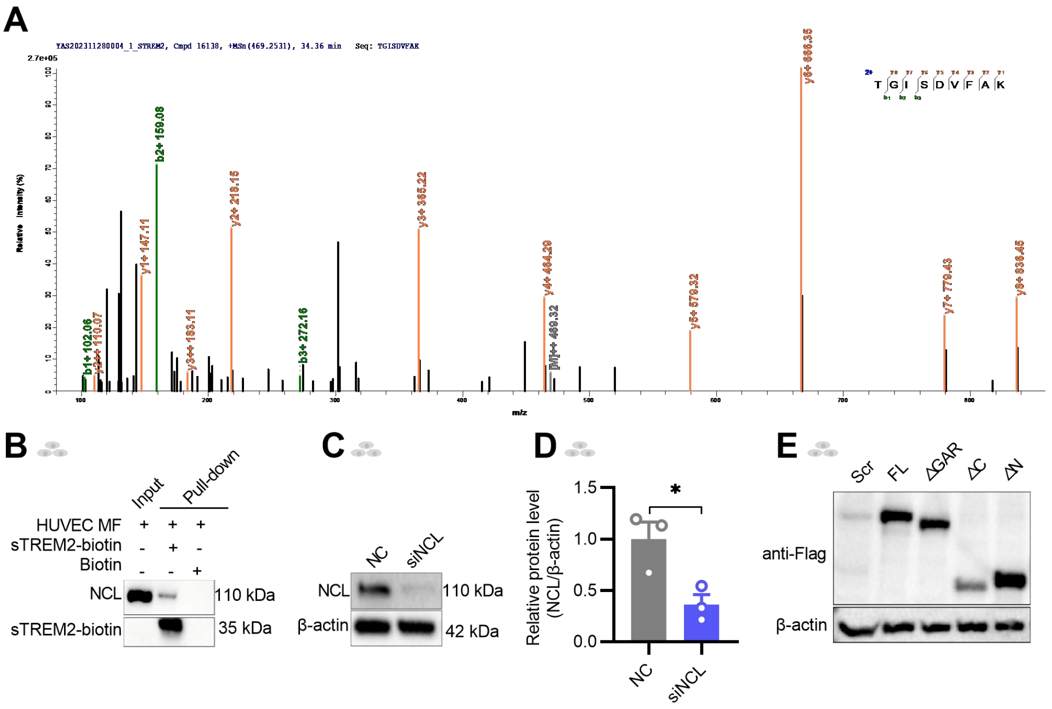

Appendix Fig. S1. Validation of NCL as an sTREM2 receptor in endothelial cells.

(A) Mass spectrometry chromatogram showing NCL peptide peaks identified from sTREM2 pull-down assays. (B) Streptavidin-agarose pull-down assay confirming interaction between sTREM2-biotin and endogenous NCL in HUVEC membrane fractions. (C, D) Western blot analysis (C) and quantification (D) of NCL knockdown efficiency in HUVECs (n = 3 biological replicates). β-actin was used as a loading control. (E) Western blot analysis of Flag-tagged NCL truncation mutants expressed in HEK293T cells (anti-Flag antibody; β-actin loading control). Data are shown as mean ± SEM. *$P < 0.05$. Exact p-values are provided in Appendix Table S2. Two-tailed Student's t-test for D.

Appendix Table S1. Sequences for All siRNAs and Plasmid Constructs

Plasmid or siRNA	Company	Sequence
NCL-FL	Genomeditech,China	PGMLV-CMV-3×Flag-H_NCL(FL)(p.1to710)-EF1-ZsGreen1-T2A-Puromycin
NCLΔGAR	Genomeditech,China	PGMLV-CMV-3×Flag-H_NCL(ΔGAR)(p.1to652)-EF1-ZsGreen1-T2A-Puromycin
NCLΔC	Genomeditech,China	PGMLV-CMV-3×Flag-H_NCL(ΔC)(p.1to269)-EF1-ZsGreen1-T2A-Puromycin
NCLΔN	Genomeditech,China	PGMLV-CMV-3×Flag-H_NCL(ΔN)(p.270to710)-EF1-ZsGreen1-T2A-Puromycin
OE-sTREM2	Genomeditech,China	PGMLV-CMV-H_TREM2(p.1to157)-HA-EF1-mScarlet-T2A-Blasticidin
OE-TREM2	Genechem,China	Ubc-TREM2-3×Flag-CBh-gcGFP-IRES-Puromycin
Sh-TREM2	Igebio,China	pLKO.1-U6-TREM2-sh2-EF1a-copGFP-T2A-Puromycin
siTREM2	Ribobio,China	CCATTACGCTGCGGAATCT
siNCL	Ribobio,China	CGTTCGGGCAAGGATAGTT

Appendix Table S2. Exact p-values

Figure	panel	comparision	p-values
1	A	Macrophage vs. Monocyte	6.4403e-219
		Macrophage vs. DC	1.3924e-145
	B	PT vs. NO	1.5910e-45
		ML vs. NO	3.7509e-24
		AC vs. NO	8.6580e-20
	D	OC vs. Ctrl	0.0003
	E	OC Mac vs. Ctrl Mac	0.0245
	F	≥ 500mL vs. < 500mL	0.0081
	G	Pearson correlation analysis	0.0217
	H	≥ 500mL vs. < 500mL	0.0377
2	A	OE-sTREM2 vs. OE-Ctrl	0.0004
	B	OE-sTREM2 vs. OE-Ctrl	2.6570e-12
	D	OE-sTREM2 vs. OE-Ctrl	0.0170
	E	sTREM2 vs. PBS	0.0148
	F	sTREM2 vs. PBS	1.8629e-6
	H	sTREM2 vs. PBS	0.0129
	J	sTREM2 vs. PBS	0.0007
	L	sTREM2 vs. PBS	2.6334e-5
	M	anti-sTREM2 vs. IgG	0.0035
	N	anti-sTREM2 vs. IgG	0.0004
	P	anti-sTREM2 vs. IgG	0.0219
3	C	OE-sTREM2 vs. OE-Ctrl	0.0011
	D	OE-sTREM2 vs. OE-Ctrl	0.0001
	E	OE-sTREM2 vs. OE-Ctrl	0.0057
	F	Pearson correlation analysis	0.0006
	I	sTREM2 vs. PBS	2.9914e-6
	J	sTREM2 vs. PBS	1.2512e-6
	K	sTREM2 vs. PBS	9.4354e-5
	L	Pearson correlation analysis	1.0645e-5
	N	sTREM2 vs. PBS	9.1907e-5
4	F	siNCL+sTREM2 vs. sTREM2	0.0211
	H	siNCL+sTREM2 vs. sTREM2	0.0112
	L	△C+sTREM2 vs. FL+sTREM2	0.0074
	N	△C+sTREM2 vs. FL+sTREM2	0.0121
5	C	sTREM2 vs. PBS (Left)	0.0350
		sTREM2 vs. PBS (Right)	0.0400
	E	NC+PBS vs. siNCL+PBS	0.9540
		NC+PBS vs. NC+sTREM2	6.0202e-5
		NC+PBS vs. siNCL+sTREM2	0.0321
		siNCL+PBS vs. NC+sTREM2	3.0218e-5

		siNCL+PBS vs. siNCL+sTREM2	0.0130
		NC+sTREM2 vs. siNCL+sTREM2	0.0095
	F	NC+PBS vs. siNCL+PBS	0.5487
		NC+PBS vs. NC+sTREM2	0.0003
		NC+PBS vs. siNCL+sTREM2	0.1359
		siNCL+PBS vs. NC+sTREM2	4.0242e-5
		siNCL+PBS vs. siNCL+sTREM2	0.0133
		NC+sTREM2 vs. siNCL+sTREM2	0.0135
	G	NC+PBS vs. siNCL+PBS	1.4649e-5
		NC+PBS vs. NC+sTREM2	0.9994
		NC+PBS vs. siNCL+sTREM2	7.4947e-7
		siNCL+PBS vs. NC+sTREM2	1.6884e-5
		siNCL+PBS vs. siNCL+sTREM2	0.0849
		NC+sTREM2 vs. siNCL+sTREM2	8.4029e-7
	H	PBS vs. L-NAME	0.9964
		PBS vs. sTREM2	0.0005
		PBS vs. L-NAME+sTREM2	0.4995
		L-NAME vs. sTREM2	0.0004
		L-NAME vs. L-NAME+sTREM2	0.3875
		sTREM2 vs. L-NAME+sTREM2	0.0058
	J	PBS vs. L-NAME	0.9190
		PBS vs. sTREM2	0.0158
		PBS vs. L-NAME+sTREM2	0.9987
		L-NAME vs. sTREM2	0.0069
		L-NAME vs. L-NAME+sTREM2	0.9606
		sTREM2 vs. L-NAME+sTREM2	0.0130
	L	L-NAME+sTREM2 vs. sTREM2	0.0043
	N	PBS vs. L-NAME	0.9139
		PBS vs. sTREM2	9.1371e-8
		PBS vs. L-NAME+sTREM2	0.2013
		L-NAME vs. sTREM2	2.9254e-8
		L-NAME vs. L-NAME+sTREM2	0.0601
		sTREM2 vs. L-NAME+sTREM2	4.7370e-6
6	A	anti-sTREM2 vs. anti-IgG	0.0012
	B	anti-sTREM2 vs. anti-IgG	0.0036
	D	anti-sTREM2 vs. anti-IgG	0.0012
	G	scFv vs. PBS	0.0002
	H	scFv vs. PBS	4.0225e-5
	I	scFv vs. PBS	0.0033
	J	Pearson correlation analysis	0.0021
	L	scFv vs. PBS	2.9934e-5
EV1	B	Normal vs. Tumor	1.4909e-18
EV2	B	OE-TREM2 vs. OE-Ctrl	0.0003

	D	Sh-TREM2 vs. Sh-Ctrl	0.0045
	F	OE-TREM2 vs. OE-Ctrl	0.0045
	G	Sh-TREM2 vs. Sh-Ctrl	0.0043
	H	OE-TREM2 vs. OE-Ctrl	0.0007
	I	OE-sTREM2 vs. OE-Ctrl	9.0505e-5
	K	anti-sTREM2 vs. IgG	3.9439e-5
EV3	C	Sh-TREM2 vs. Sh-Ctrl	0.0003
	D	Sh-TREM2 vs. Sh-Ctrl	7.0976e-5
	E	Sh-TREM2 vs. Sh-Ctrl	0.0003
	F	Pearson correlation analysis	3.4645e-5
Appendix Figure S1	D	SiNCL vs. NC	0.0286
EV4	B	sTREM2 vs.PBS	0.0157
		sTREM2 vs.PBS	0.0172
	D	PBS vs. L-NAME	0.4685
		PBS vs. sTREM2	0.0138
		PBS vs. L-NAME+sTREM2	0.9545
		L-NAME vs. sTREM2	0.0021
		L-NAME vs. L-NAME+sTREM2	0.7469
		sTREM2 vs. L-NAME+sTREM2	0.0071
	F	PBS vs. MK2206	0.1394
		PBS vs. sTREM2	0.0025
		PBS vs. MK2206+sTREM2	0.6891
		MK2206 vs. sTREM2	6.6962e-5
		MK2206 vs. MK2206+sTREM2	0.0209
		sTREM2 vs. MK2206+sTREM2	0.0167
	G	PBS vs. MK2206	0.4561
		PBS vs. sTREM2	0.0079
		PBS vs. MK2206+sTREM2	0.8454
		MK2206 vs. sTREM2	0.0006
		MK2206 vs. MK2206+sTREM2	0.1437
		sTREM2 vs. MK2206+sTREM2	0.0330
	H	PBS vs. MK2206	0.6128
		PBS vs. sTREM2	5.3742e-6
		PBS vs. MK2206+sTREM2	0.0059
		MK2206 vs. sTREM2	2.4654e-5
		MK2206 vs. MK2206+sTREM2	0.0514
		sTREM2 vs. MK2206+sTREM2	0.0019
	J	OC vs. Ctrl	0.0075
EV5	A	si-TREM2 vs. NC	0.0006
	B	si-TREM2 vs. NC	0.0124
	D	si-TREM2 vs. NC	0.0060
	E	PBS vs. scFv	0.9332

		PBS vs. sTREM2	0.0001
		PBS vs. sTREM2+scFv	0.1487
		scFv vs. sTREM2	0.0003
		scFv vs. sTREM2+scFv	0.3530
		sTREM2 vs. sTREM2+scFv	0.0055
	G	PBS vs. sTREM2	1.2362e-9
		PBS vs. sTREM2+scFv	1.1778e-4
		sTREM2 vs. sTREM2+scFv	1.1980e-6
