# Supplementary figures and images for: Soluble TREM2 engages cell-surface nucleolin to drive vascular permeability and malignant ascites in ovarian cancer (part 1 of 2)
Source: EMBO Mol Med. 2026 May 26;18(7):2667–90. doi: 10.1038/s44321-026-00452-2 (PMC13365401; doi:10.1038/s44321-026-00452-2)

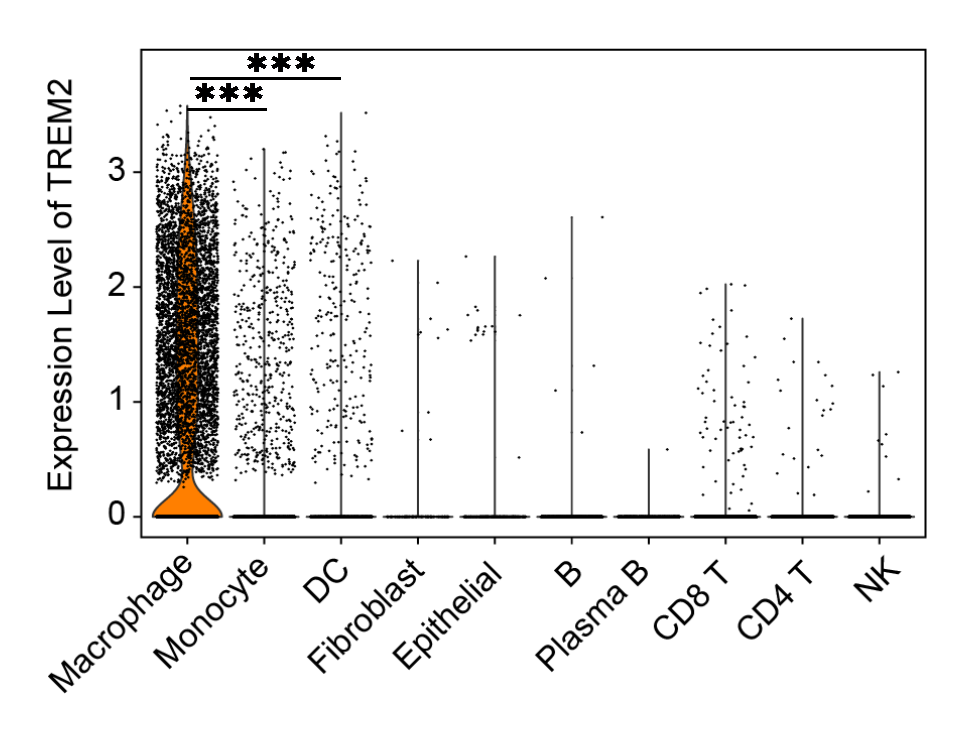

Supplement: Supplementary file 3 — Source data Fig. 1 [file 44321_2026_452_MOESM3_ESM.zip › Figure 1/1A/Figure 1A.tif]

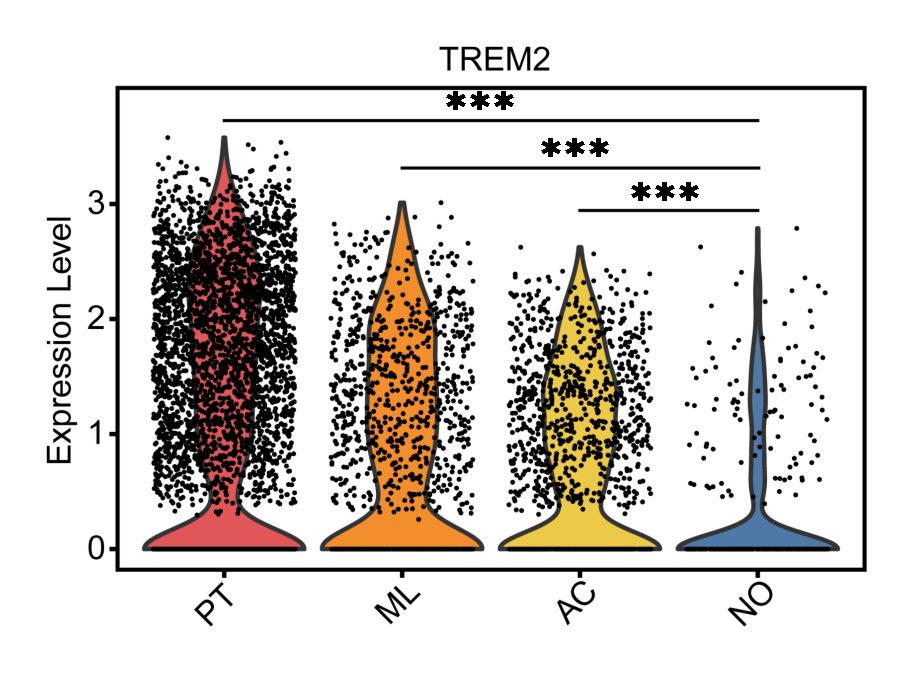

Supplement: Supplementary file 3 — Source data Fig. 1 [file 44321_2026_452_MOESM3_ESM.zip › Figure 1/1B/Figure 1B.tif]

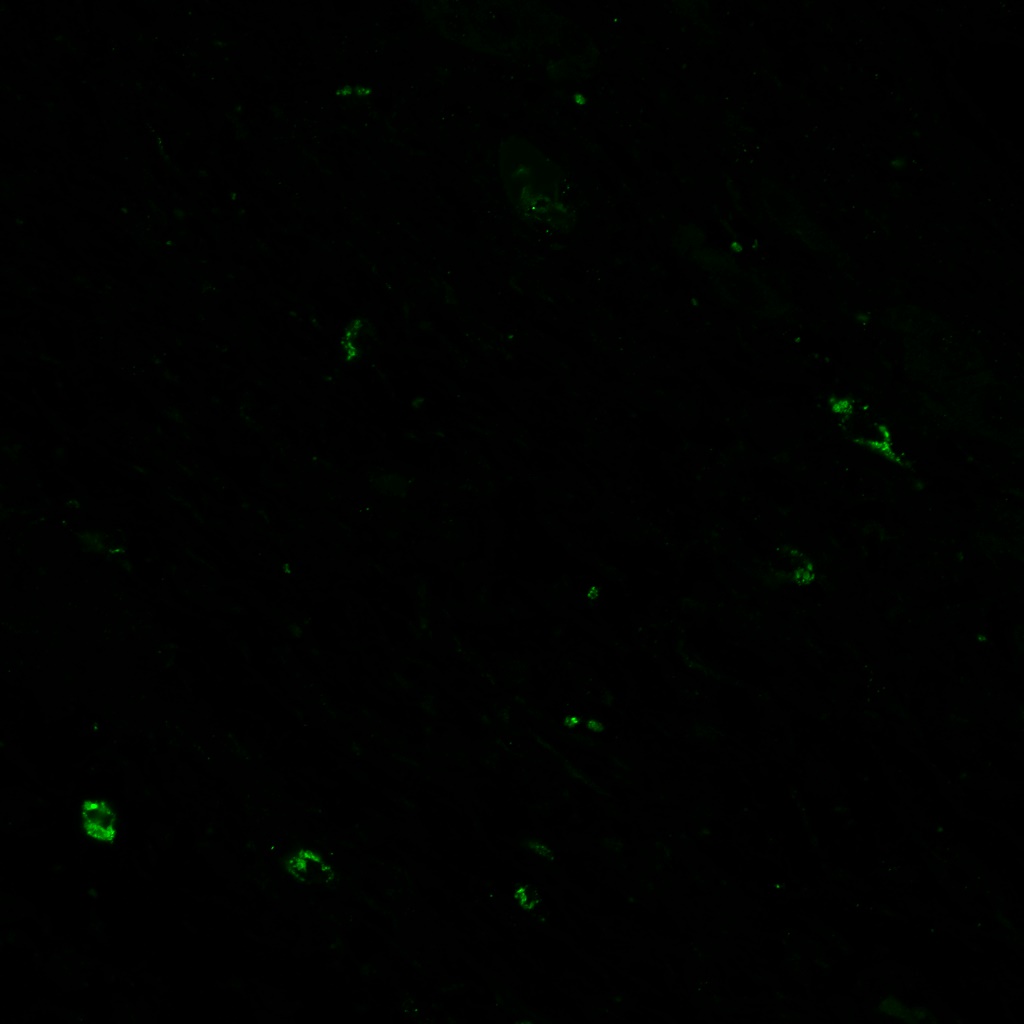

Supplement: Supplementary file 3 — Source data Fig. 1 [file 44321_2026_452_MOESM3_ESM.zip › Figure 1/1C-D/IF Ctrl CD68.jpg]

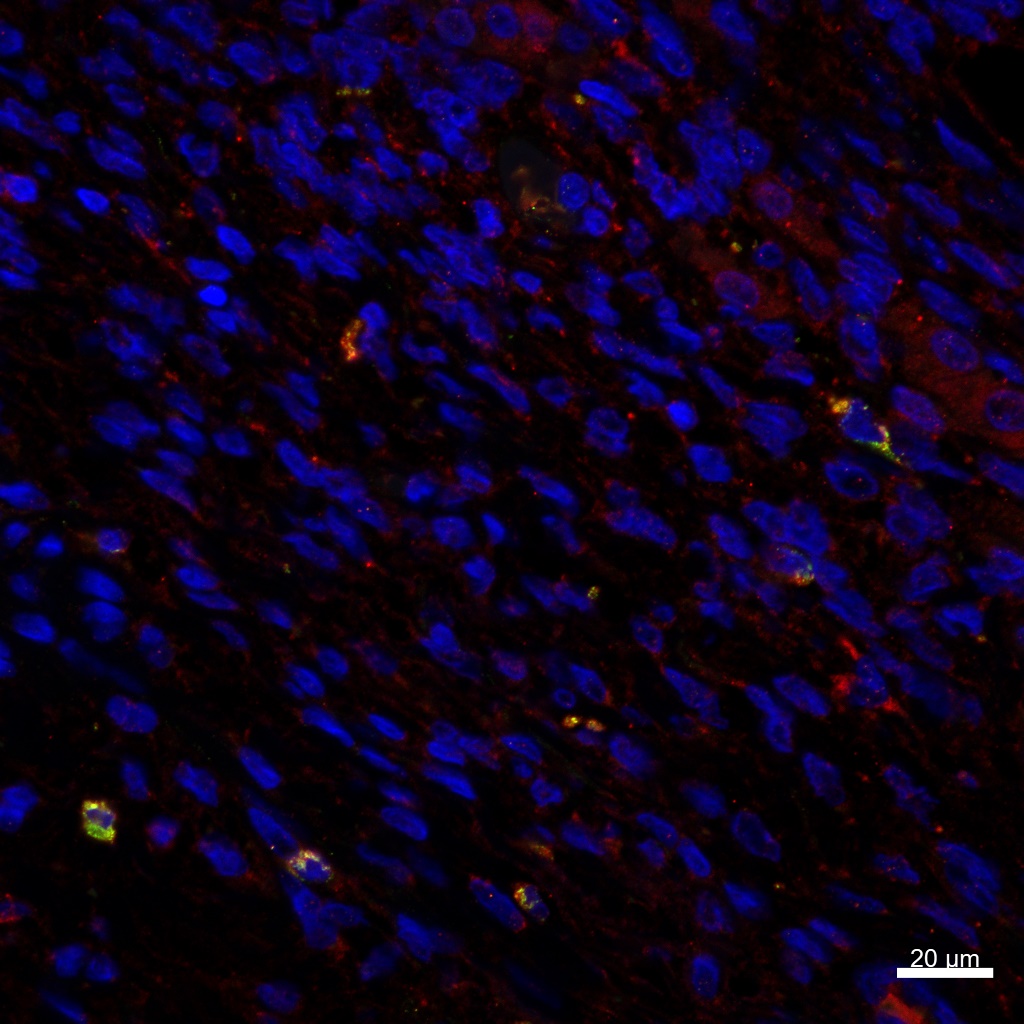

Supplement: Supplementary file 3 — Source data Fig. 1 [file 44321_2026_452_MOESM3_ESM.zip › Figure 1/1C-D/IF Ctrl Merge.jpg]

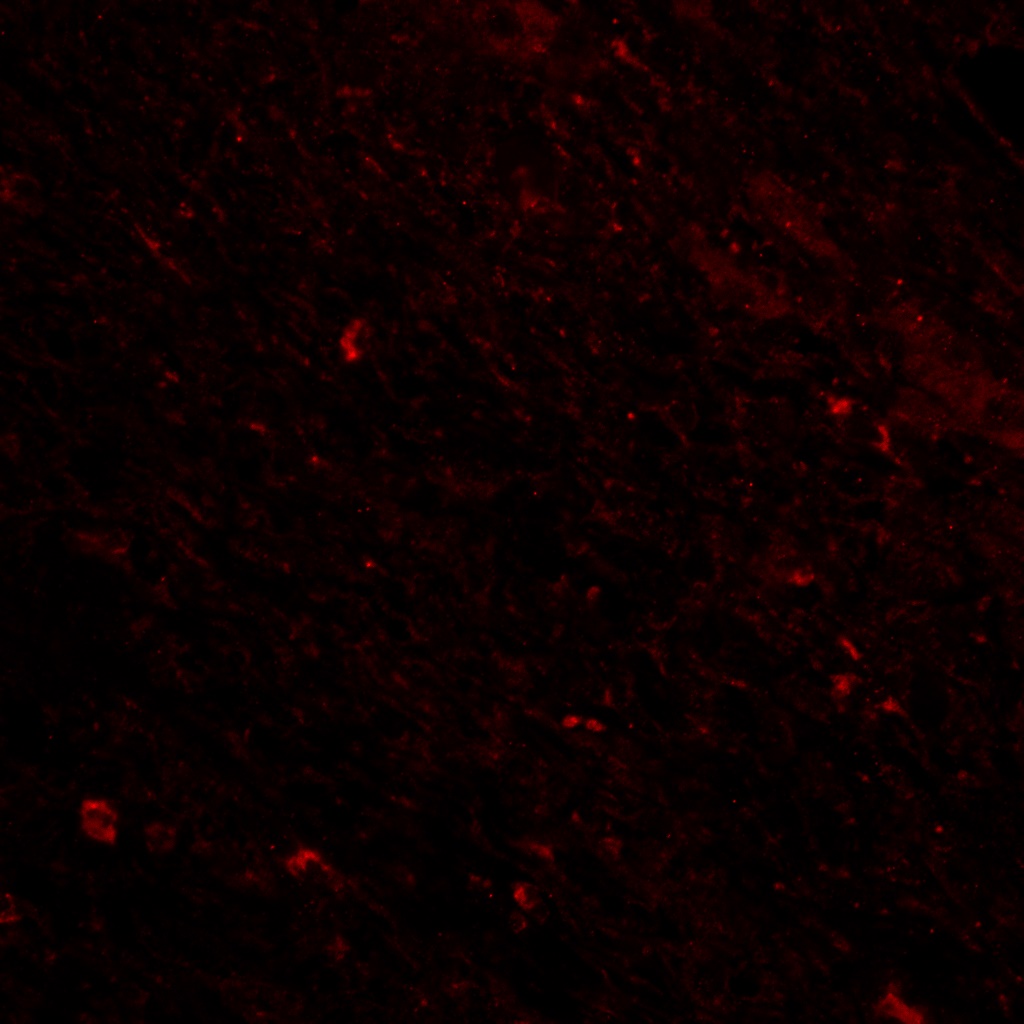

Supplement: Supplementary file 3 — Source data Fig. 1 [file 44321_2026_452_MOESM3_ESM.zip › Figure 1/1C-D/IF Ctrl TREM2.jpg]

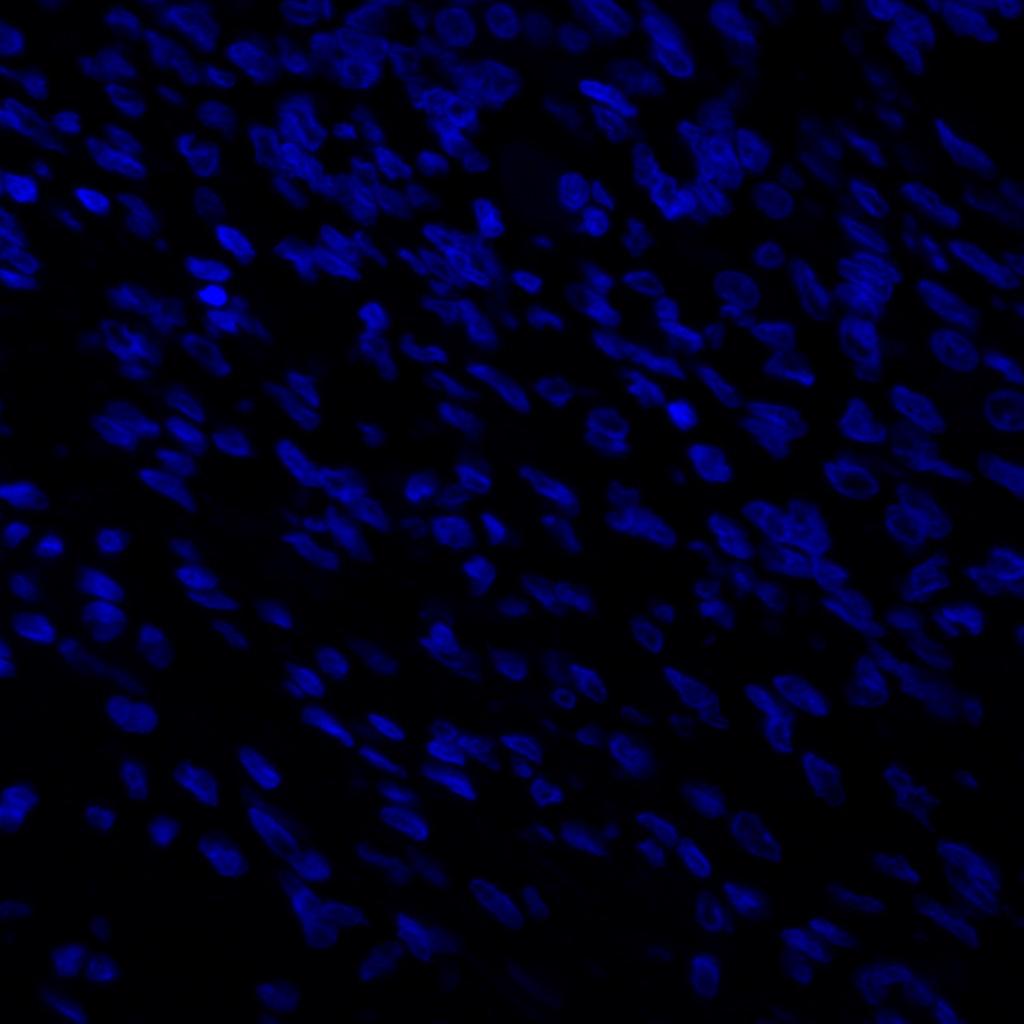

Supplement: Supplementary file 3 — Source data Fig. 1 [file 44321_2026_452_MOESM3_ESM.zip › Figure 1/1C-D/IF Ctrl DAPI.jpg]

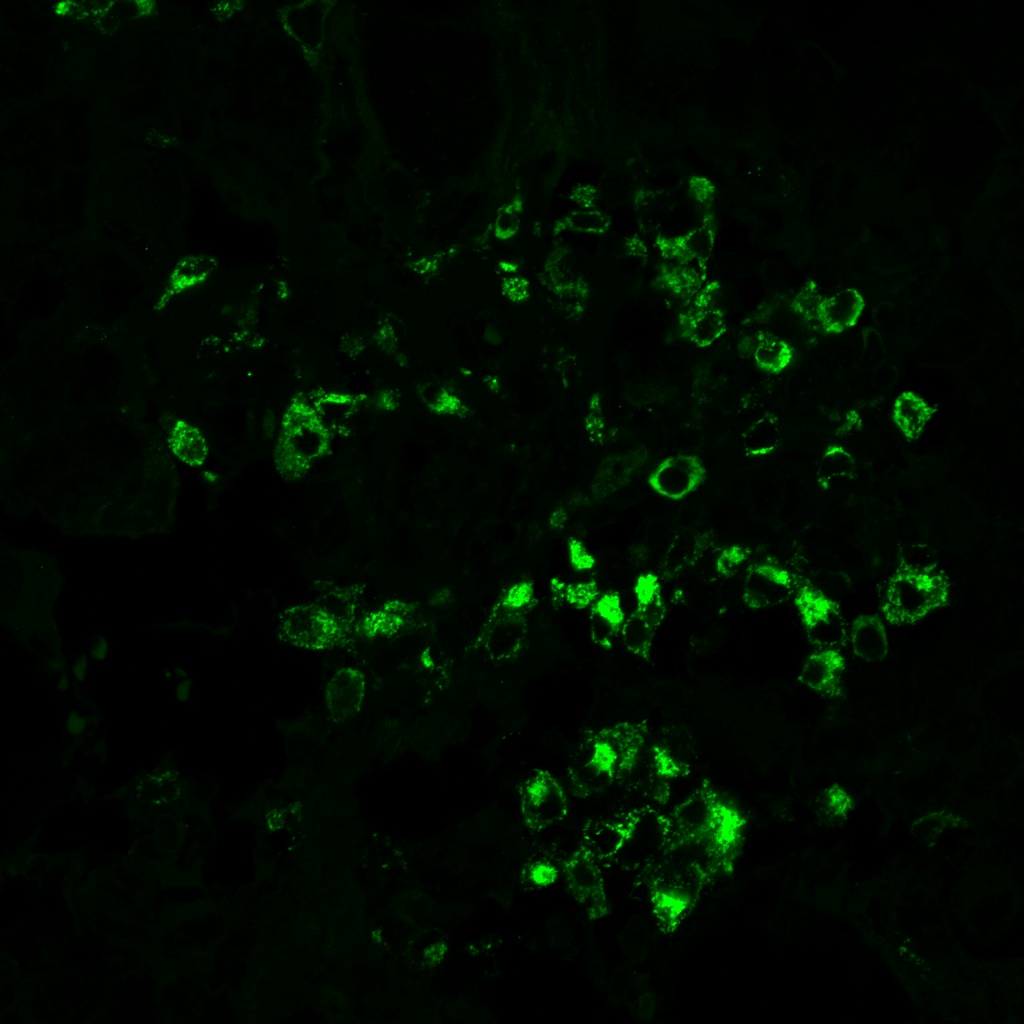

Supplement: Supplementary file 3 — Source data Fig. 1 [file 44321_2026_452_MOESM3_ESM.zip › Figure 1/1C-D/IF OC CD68.jpg]

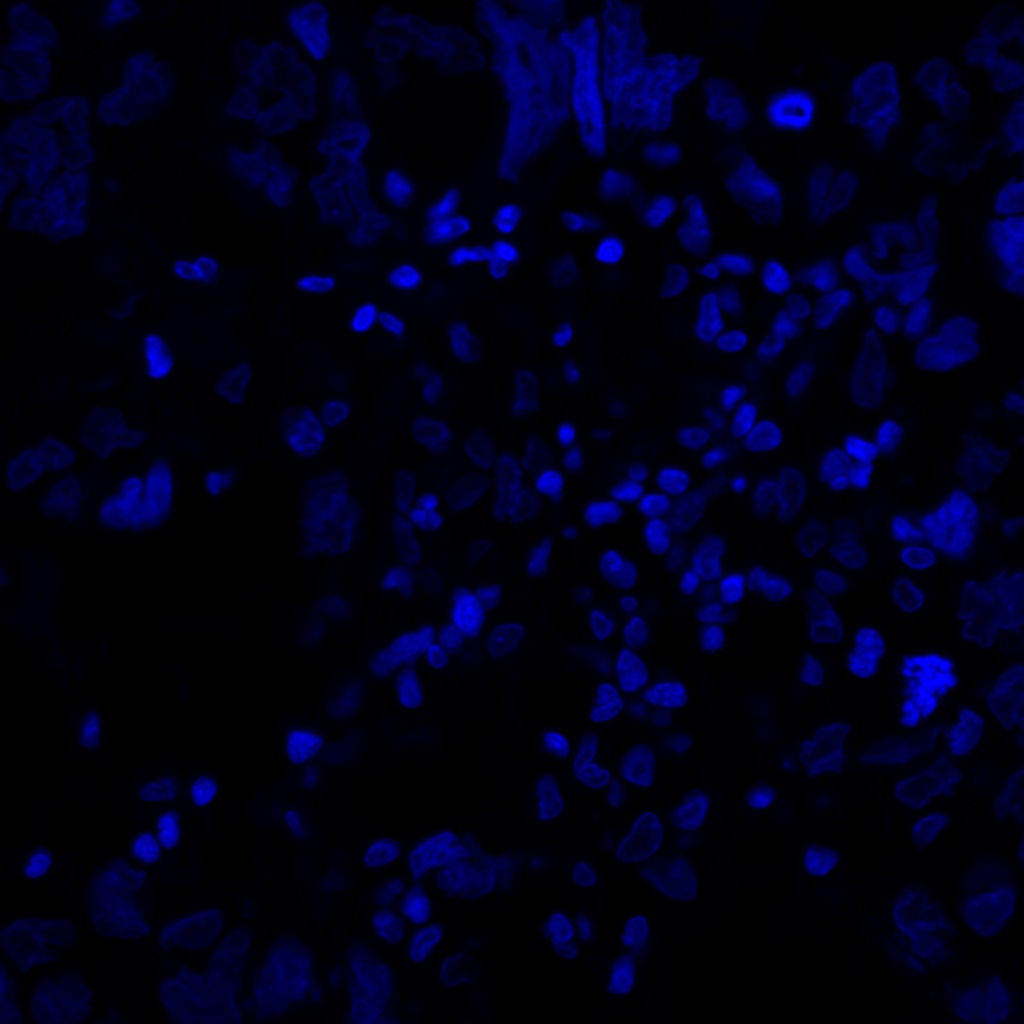

Supplement: Supplementary file 3 — Source data Fig. 1 [file 44321_2026_452_MOESM3_ESM.zip › Figure 1/1C-D/IF OC DAPI.jpg]

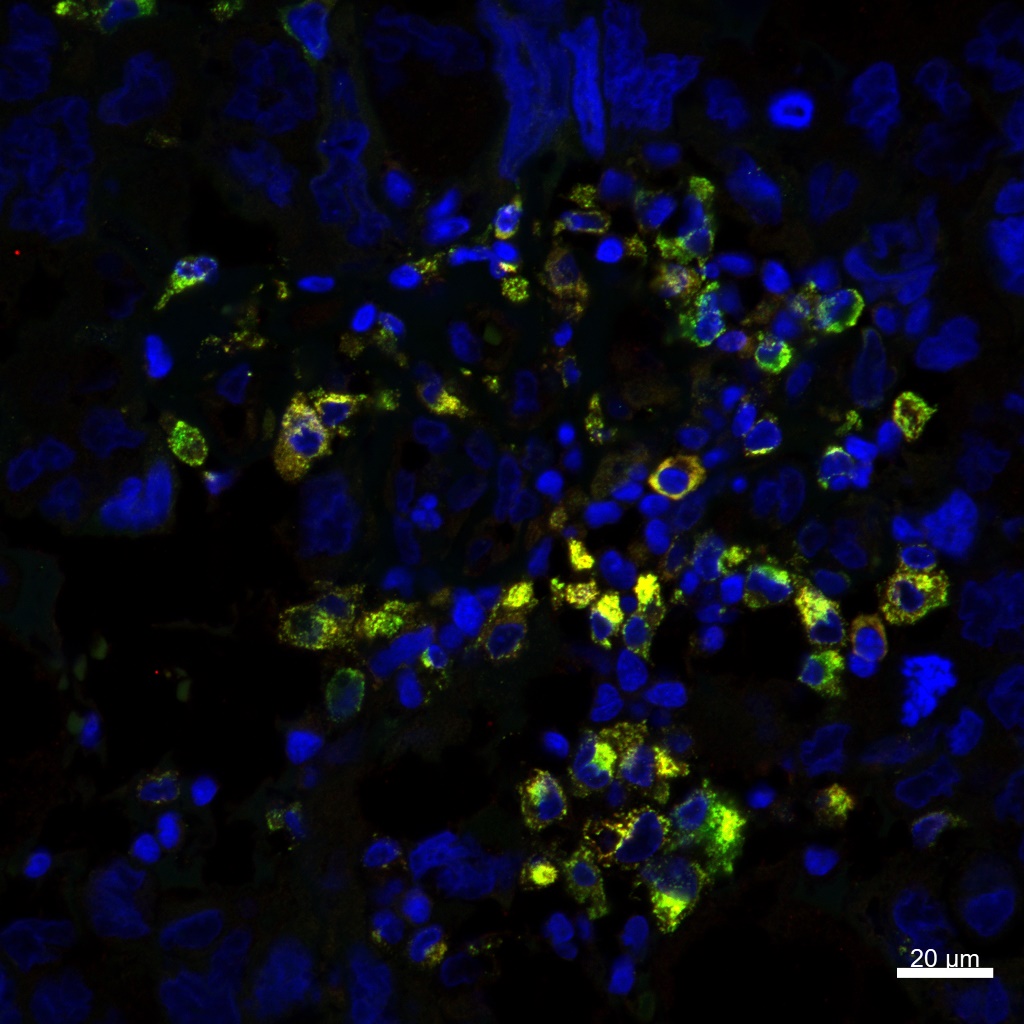

Supplement: Supplementary file 3 — Source data Fig. 1 [file 44321_2026_452_MOESM3_ESM.zip › Figure 1/1C-D/IF OC Merge.jpg]

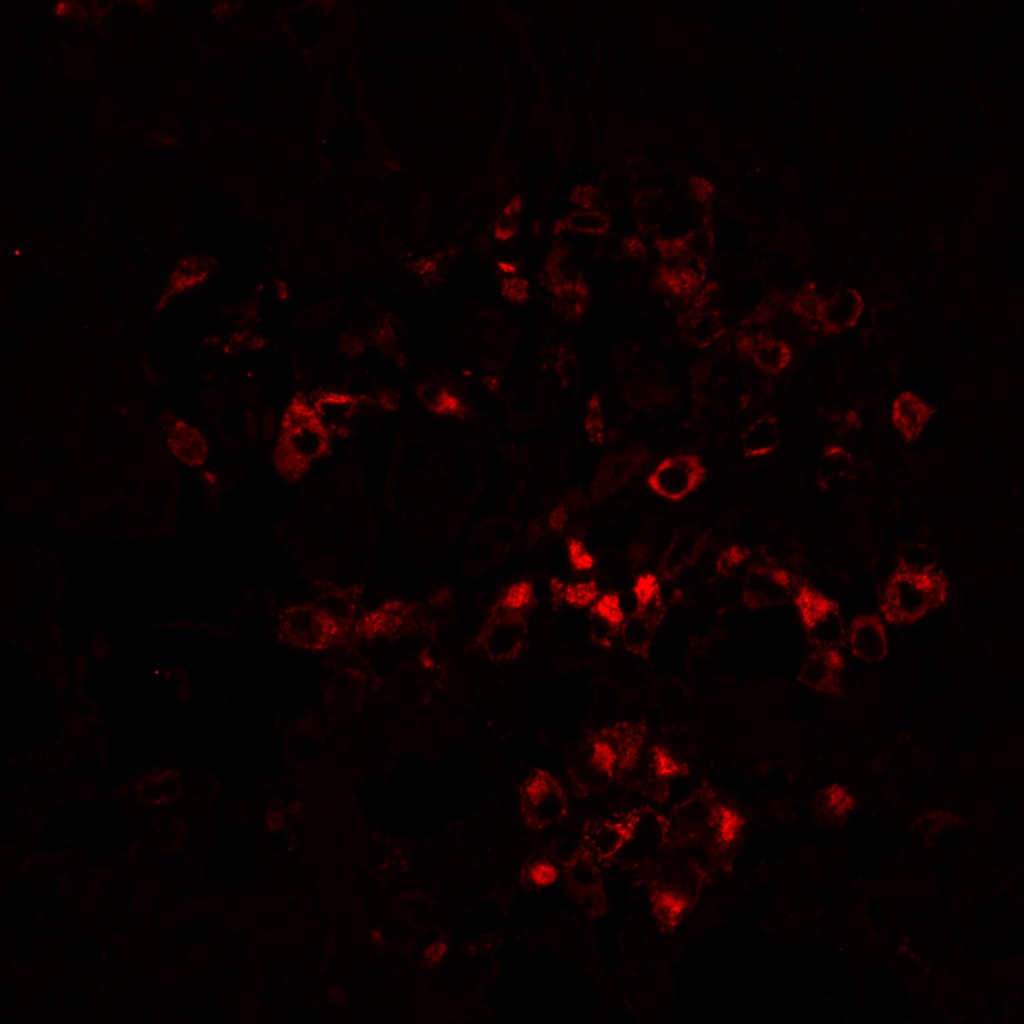

Supplement: Supplementary file 3 — Source data Fig. 1 [file 44321_2026_452_MOESM3_ESM.zip › Figure 1/1C-D/IF OC TREM2.jpg]

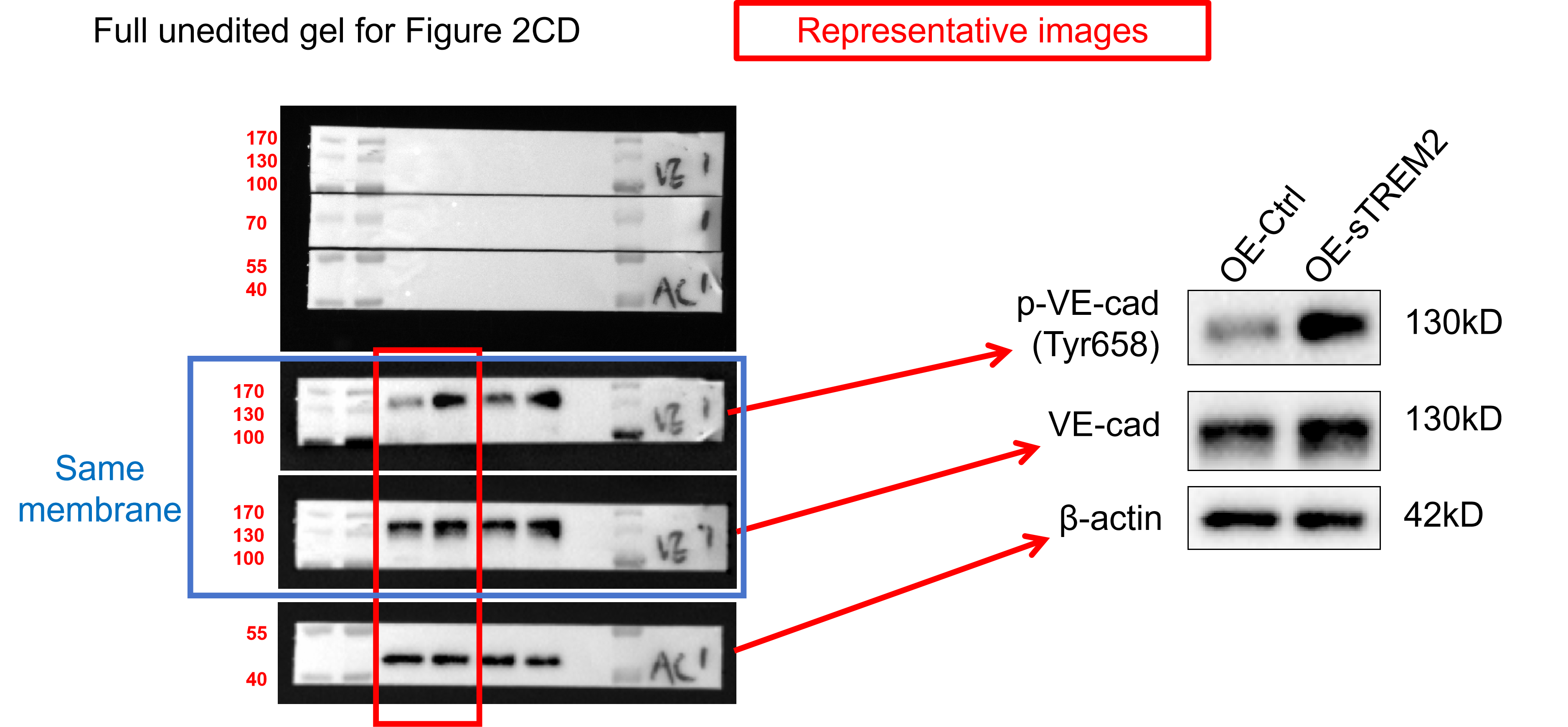

Supplement: Supplementary file 4 — Source data Fig. 2 [file 44321_2026_452_MOESM4_ESM.zip › Figure 2/2C-D/Instructions for cropping Western blot images 1.tif]

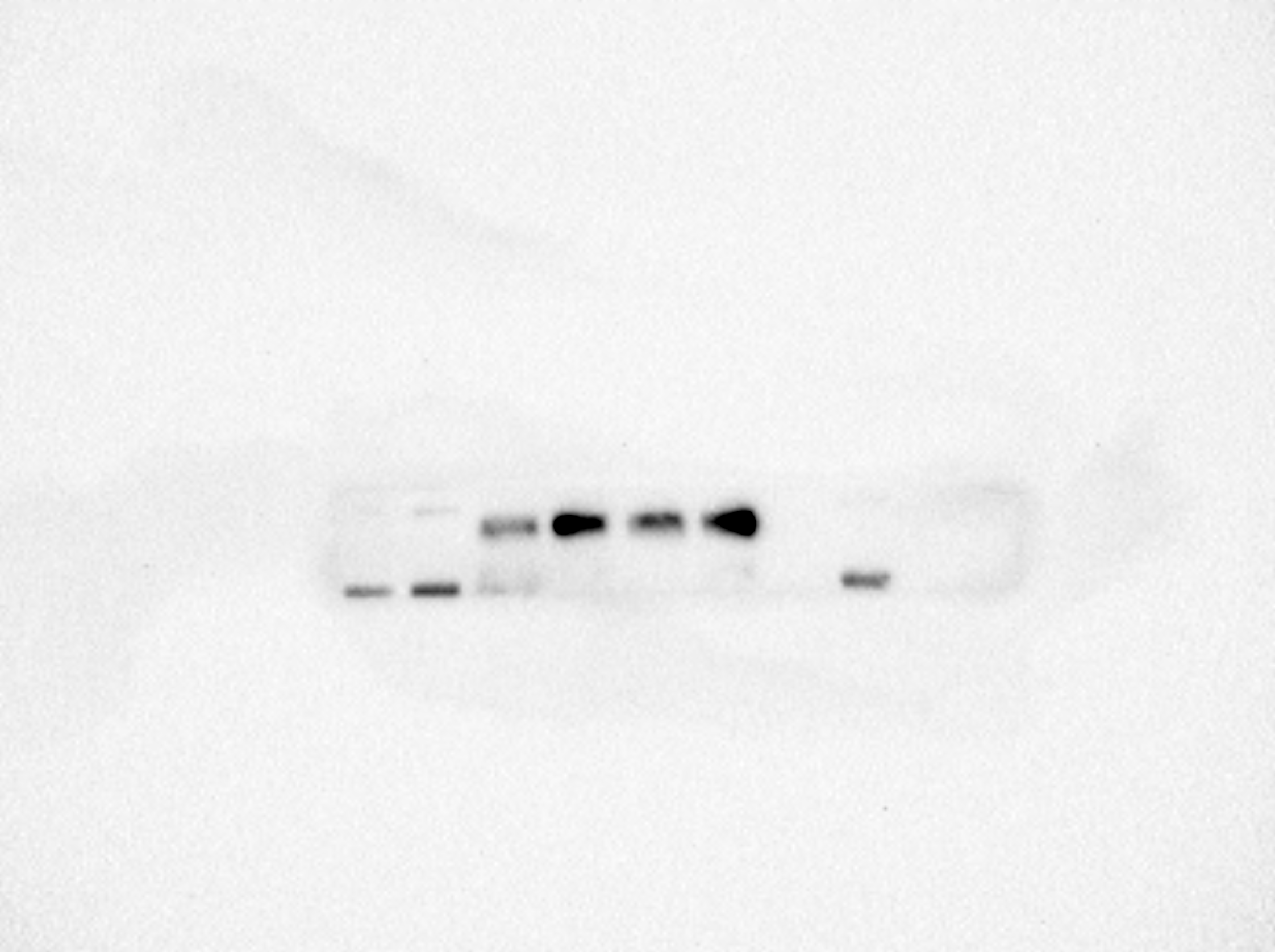

Supplement: Supplementary file 4 — Source data Fig. 2 [file 44321_2026_452_MOESM4_ESM.zip › Figure 2/2C-D/WB_ Uncropped blots_ p-VEcad.tif]

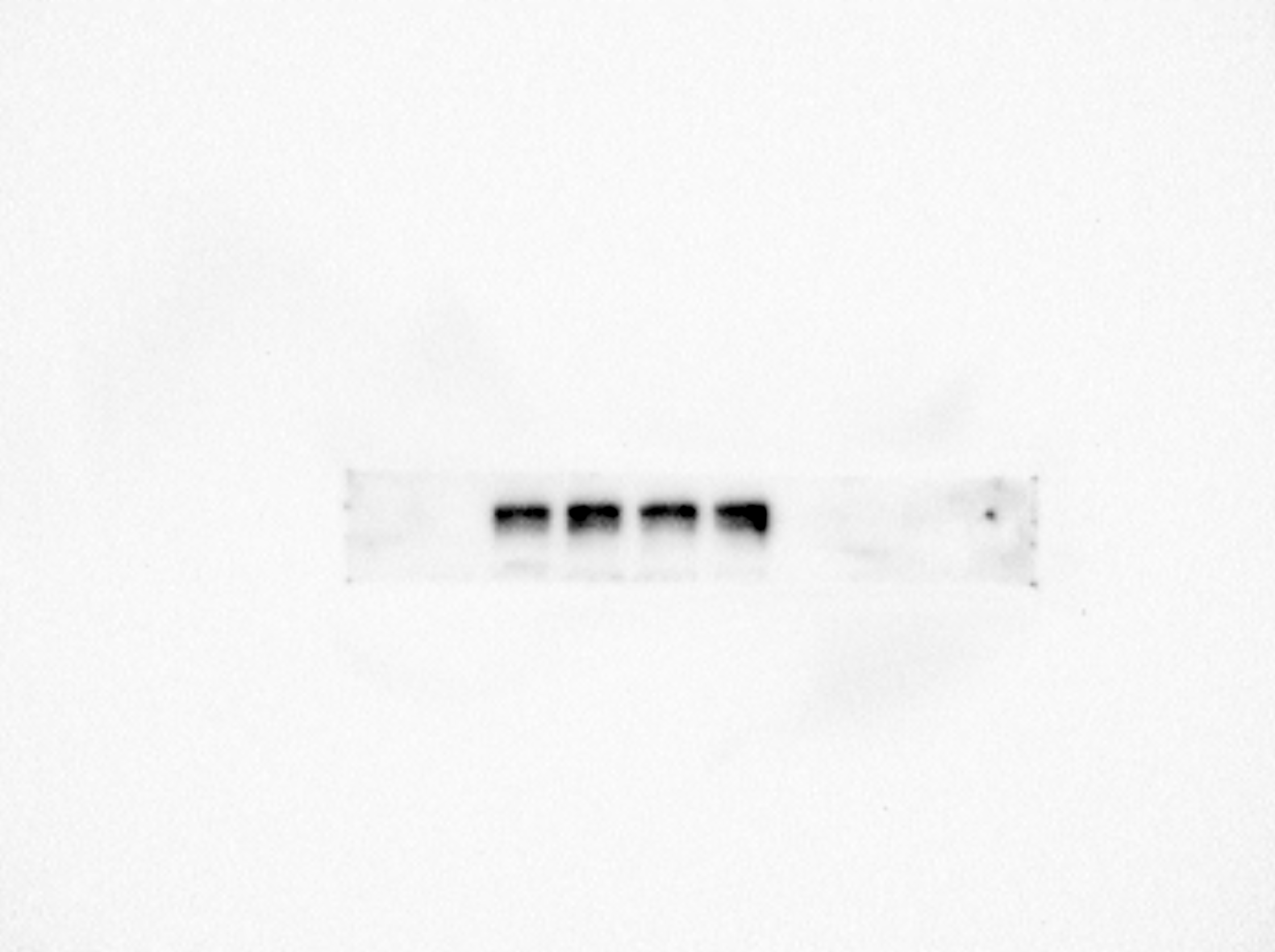

Supplement: Supplementary file 4 — Source data Fig. 2 [file 44321_2026_452_MOESM4_ESM.zip › Figure 2/2C-D/WB_ Uncropped blots_ VEcad.tif]

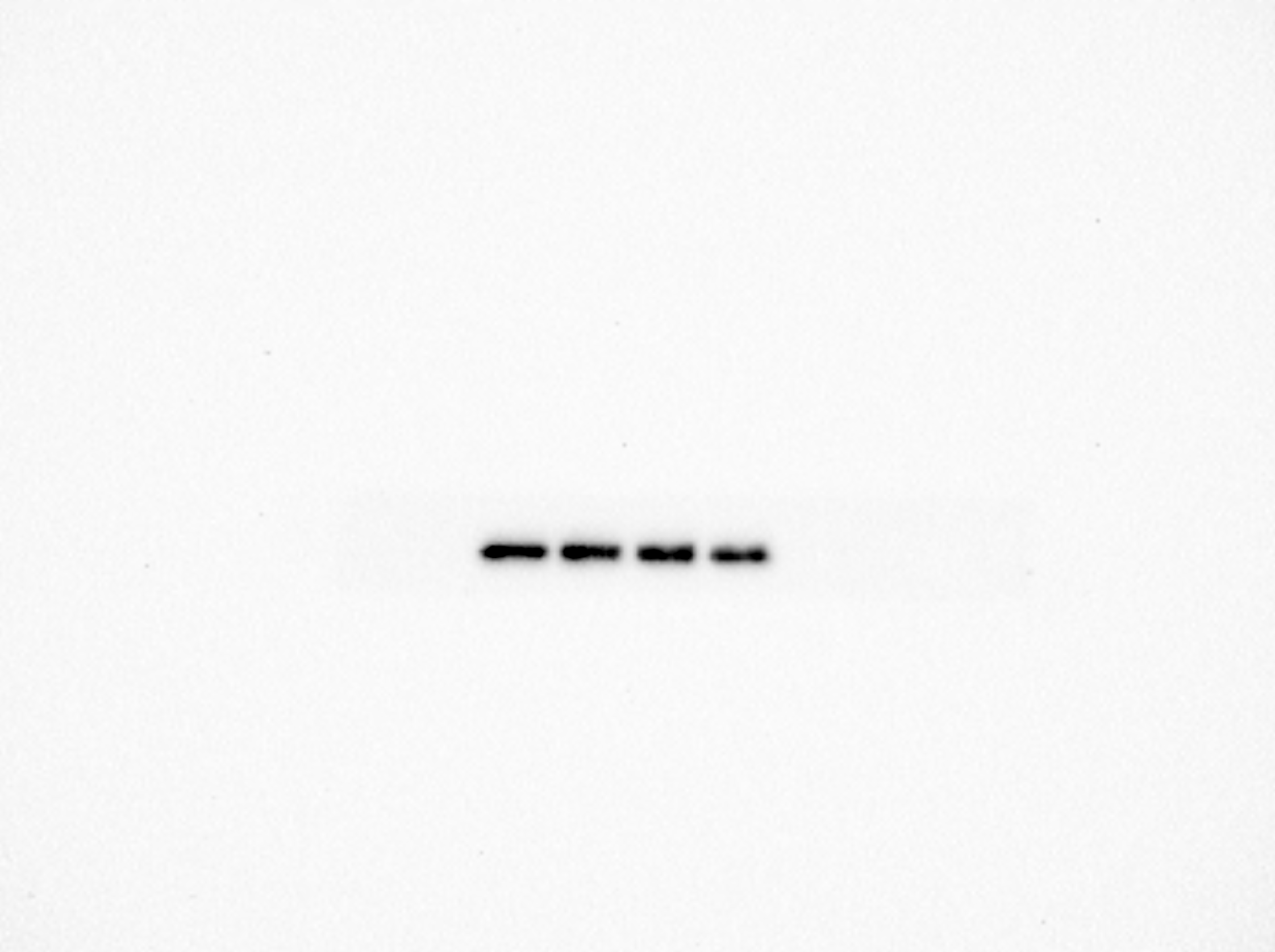

Supplement: Supplementary file 4 — Source data Fig. 2 [file 44321_2026_452_MOESM4_ESM.zip › Figure 2/2C-D/WB_ Uncropped blots_ β-actin.tif]

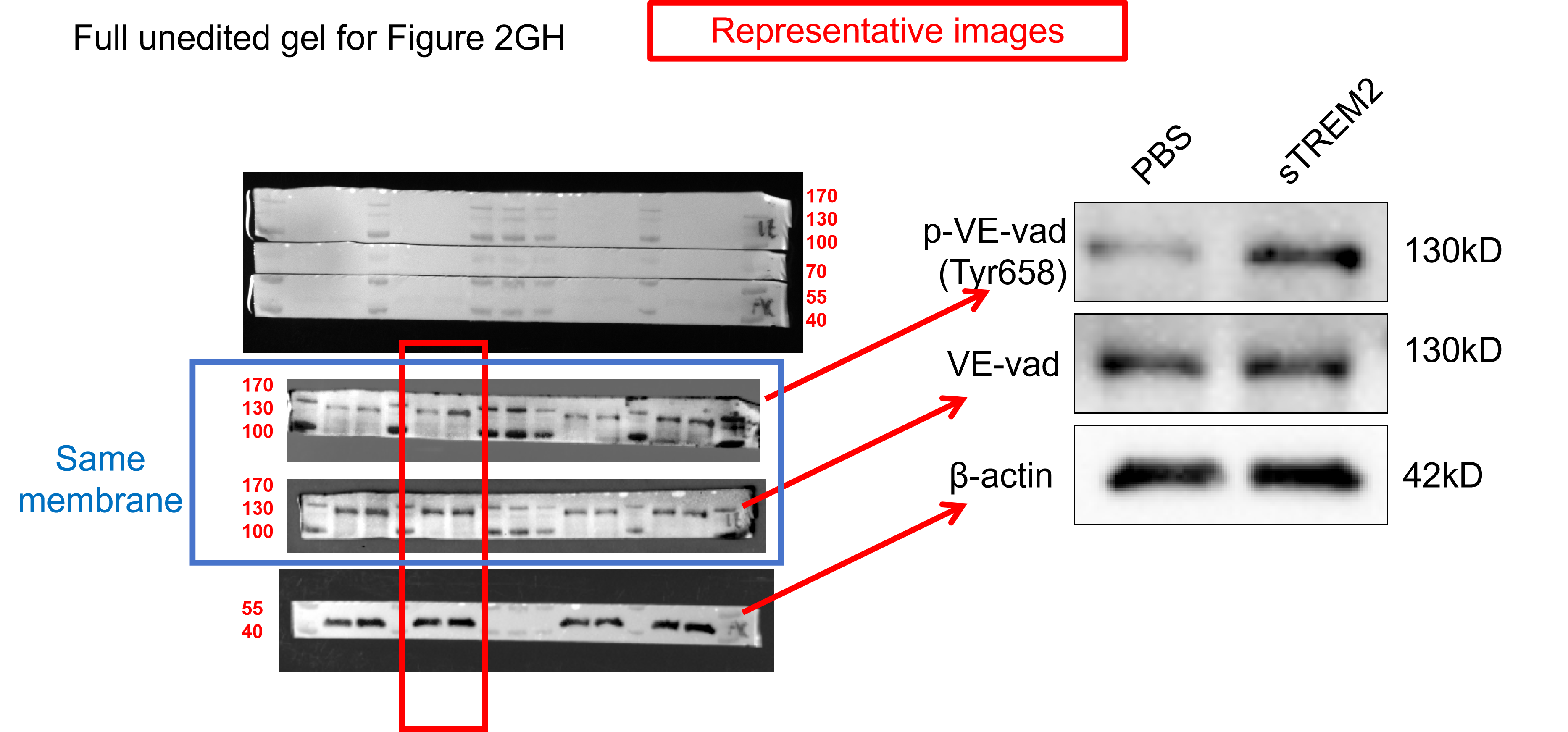

Supplement: Supplementary file 4 — Source data Fig. 2 [file 44321_2026_452_MOESM4_ESM.zip › Figure 2/2G-H/Instructions for cropping Western blot images 1.tif]

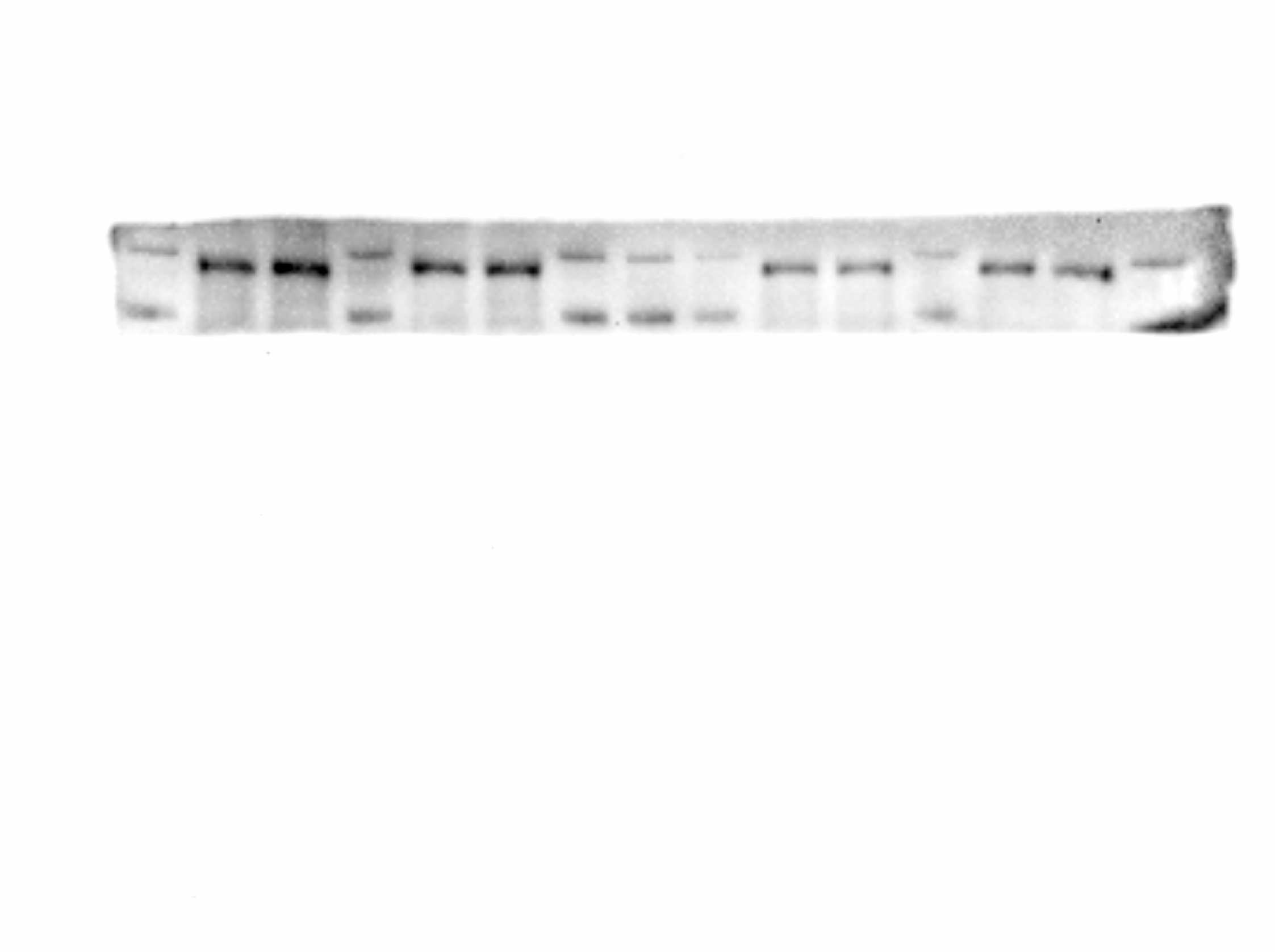

Supplement: Supplementary file 4 — Source data Fig. 2 [file 44321_2026_452_MOESM4_ESM.zip › Figure 2/2G-H/WB_ Uncropped blots_ VEcad.tif]

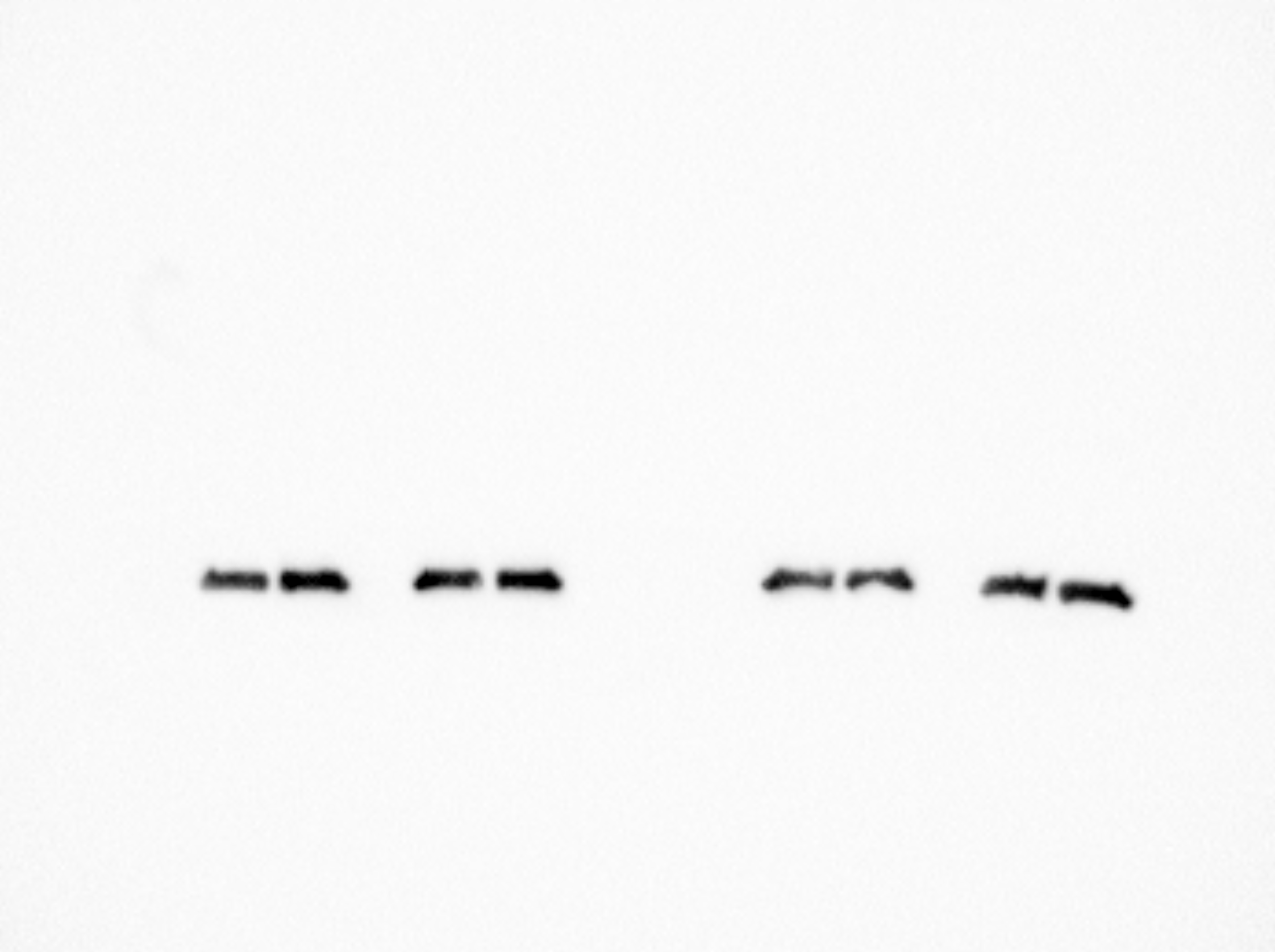

Supplement: Supplementary file 4 — Source data Fig. 2 [file 44321_2026_452_MOESM4_ESM.zip › Figure 2/2G-H/WB_ Uncropped blots_ β-actin.tif]

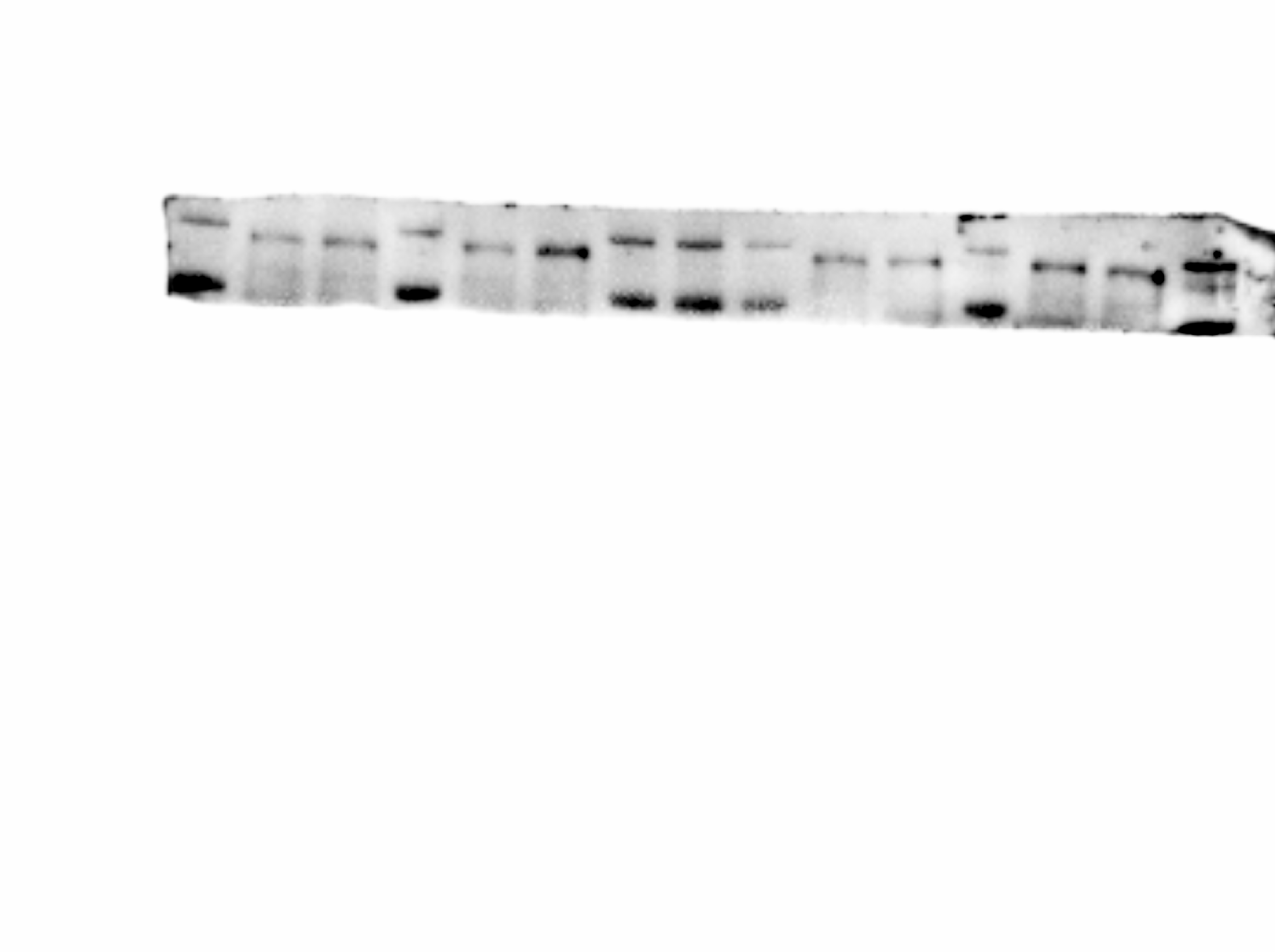

Supplement: Supplementary file 4 — Source data Fig. 2 [file 44321_2026_452_MOESM4_ESM.zip › Figure 2/2G-H/WB_ Uncropped blots_p-VEcad.tif]

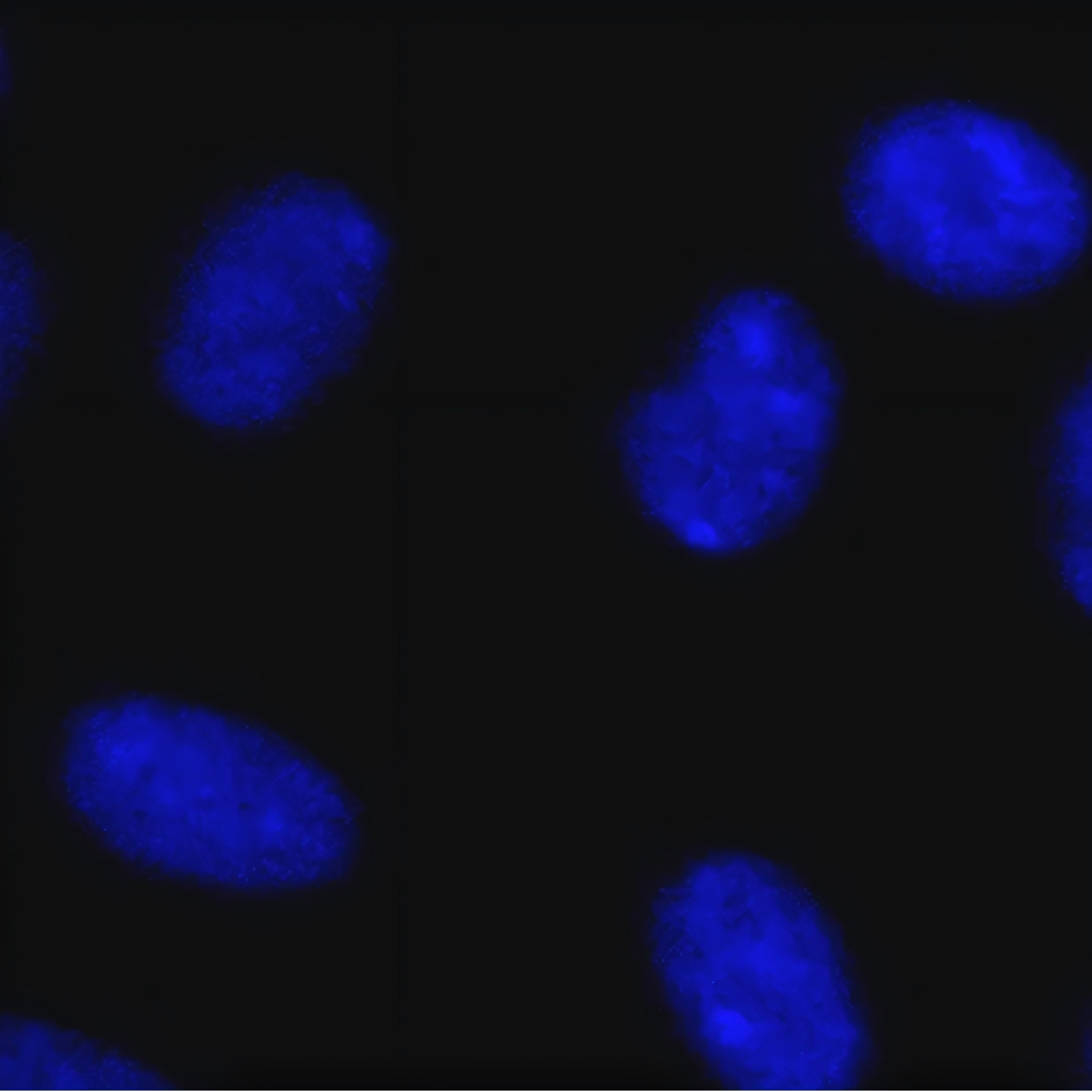

Supplement: Supplementary file 4 — Source data Fig. 2 [file 44321_2026_452_MOESM4_ESM.zip › Figure 2/2I-J/PBS DAPI.tif]

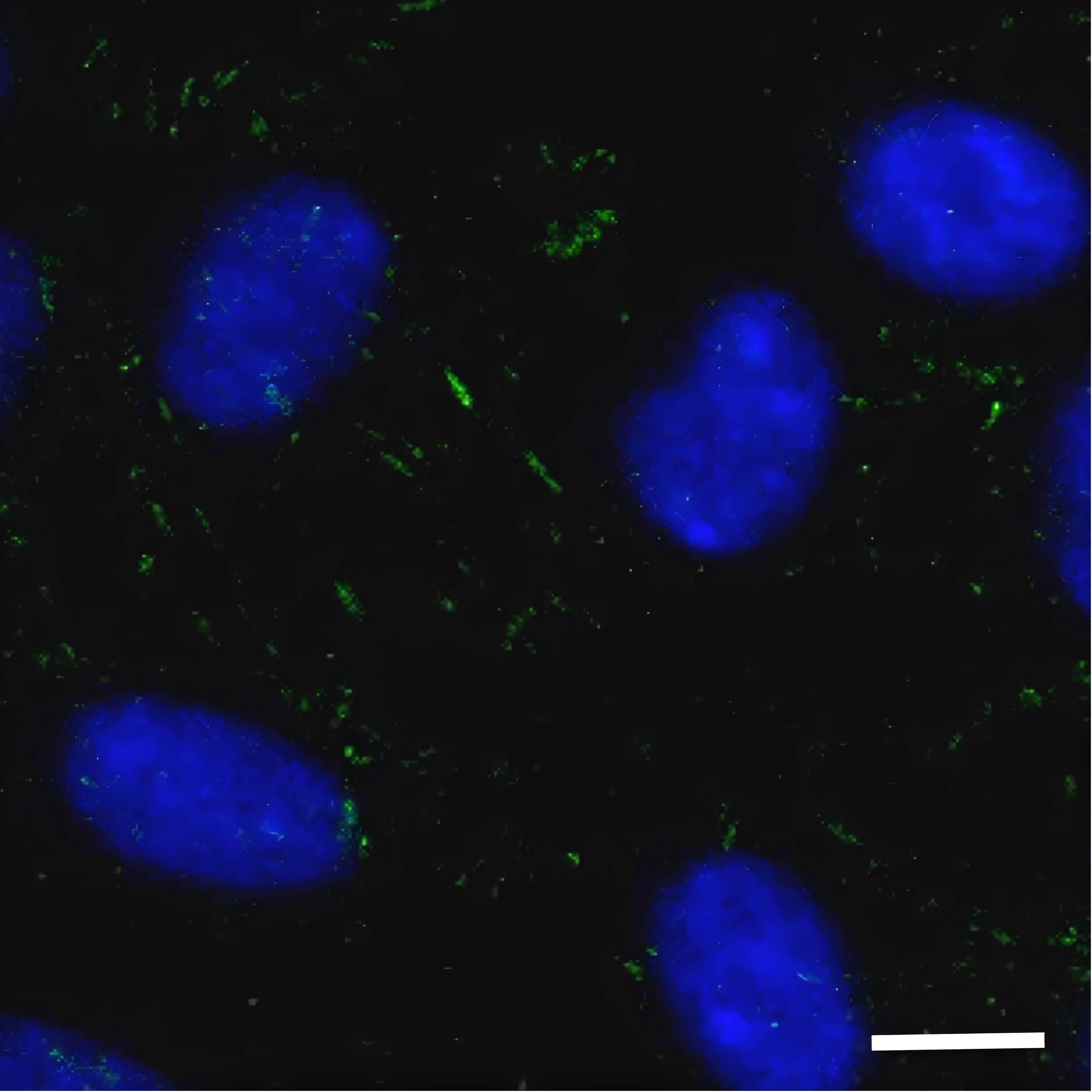

Supplement: Supplementary file 4 — Source data Fig. 2 [file 44321_2026_452_MOESM4_ESM.zip › Figure 2/2I-J/PBS Merge.tif]

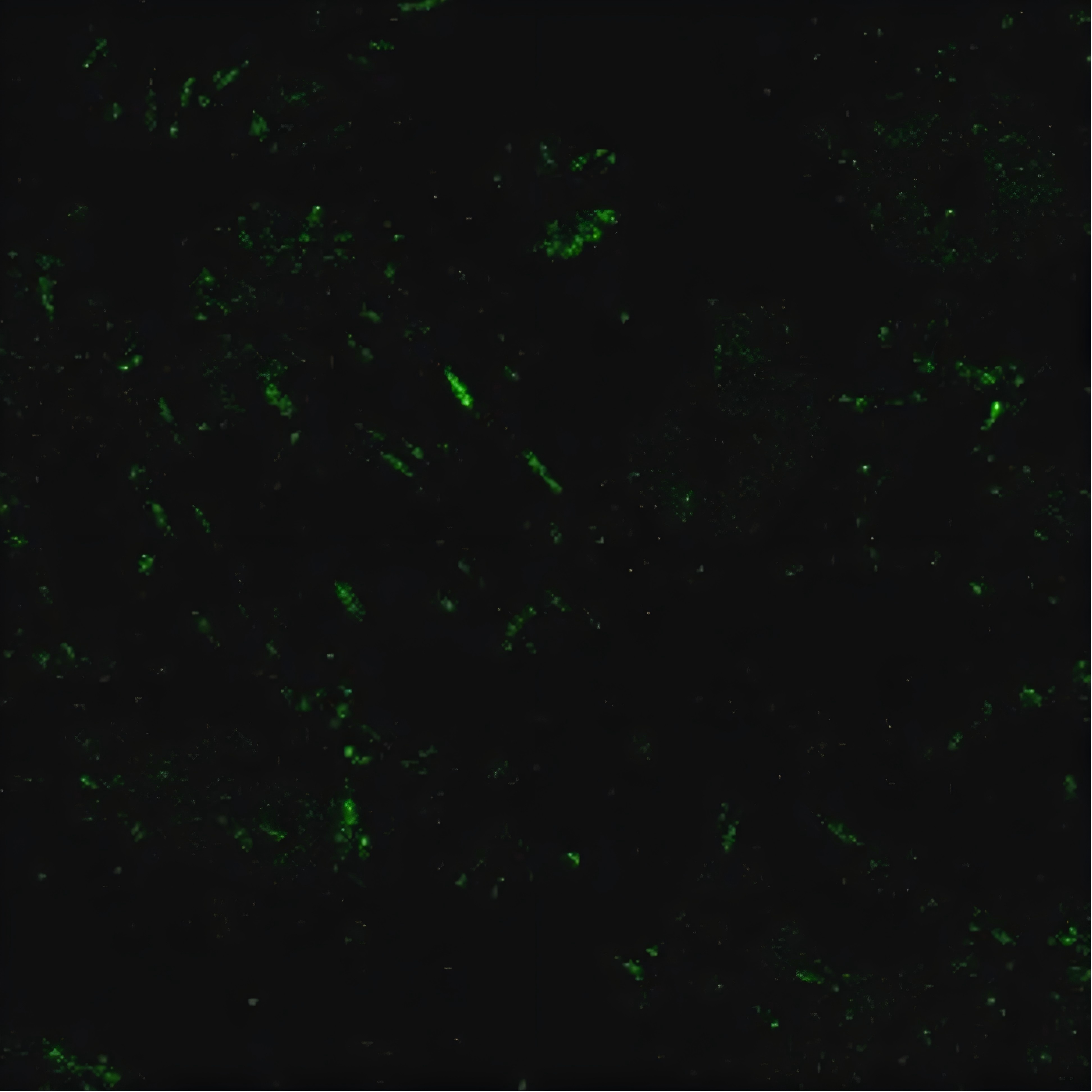

Supplement: Supplementary file 4 — Source data Fig. 2 [file 44321_2026_452_MOESM4_ESM.zip › Figure 2/2I-J/PBS p-VEcad.tif]

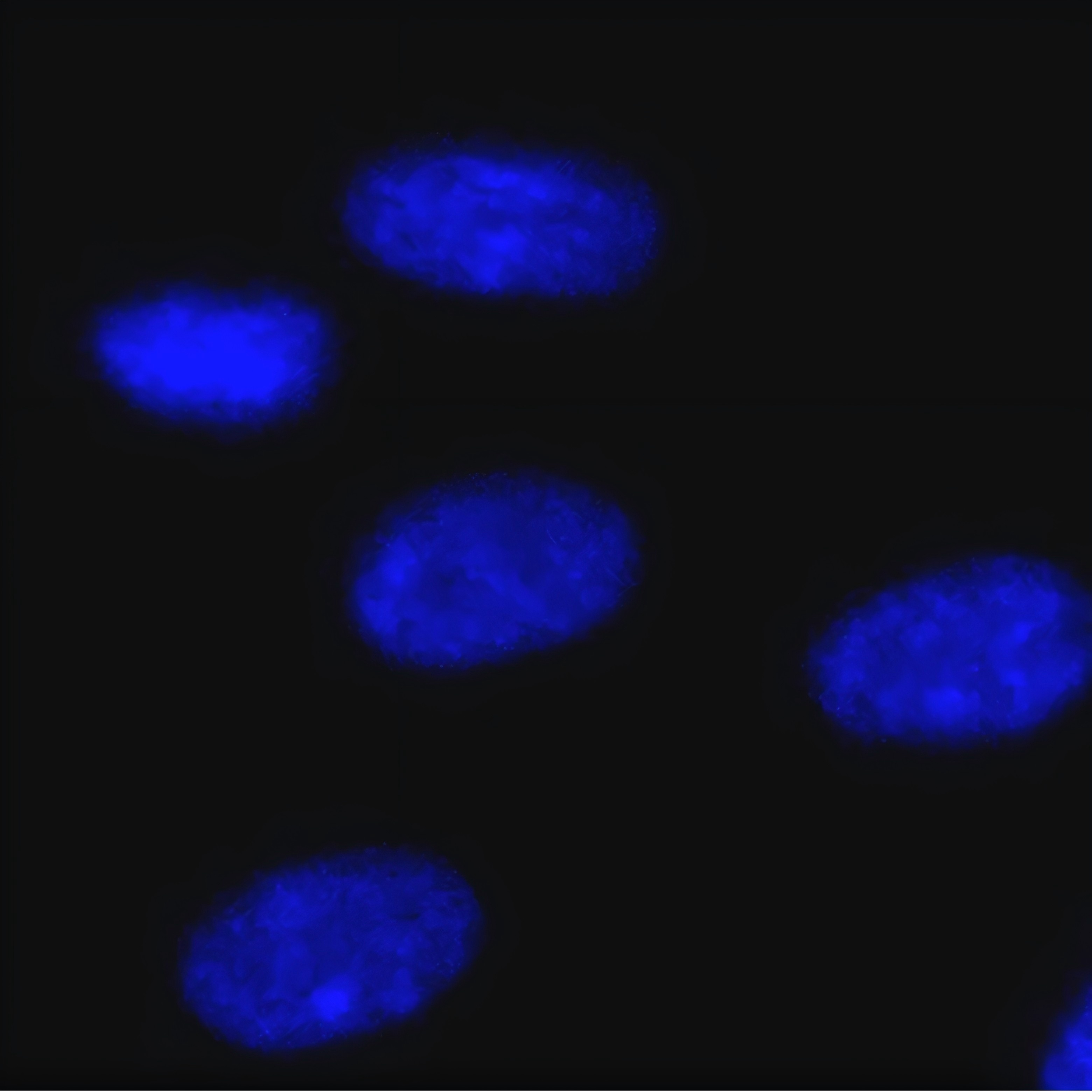

Supplement: Supplementary file 4 — Source data Fig. 2 [file 44321_2026_452_MOESM4_ESM.zip › Figure 2/2I-J/sTREM2 DAPI.tif]

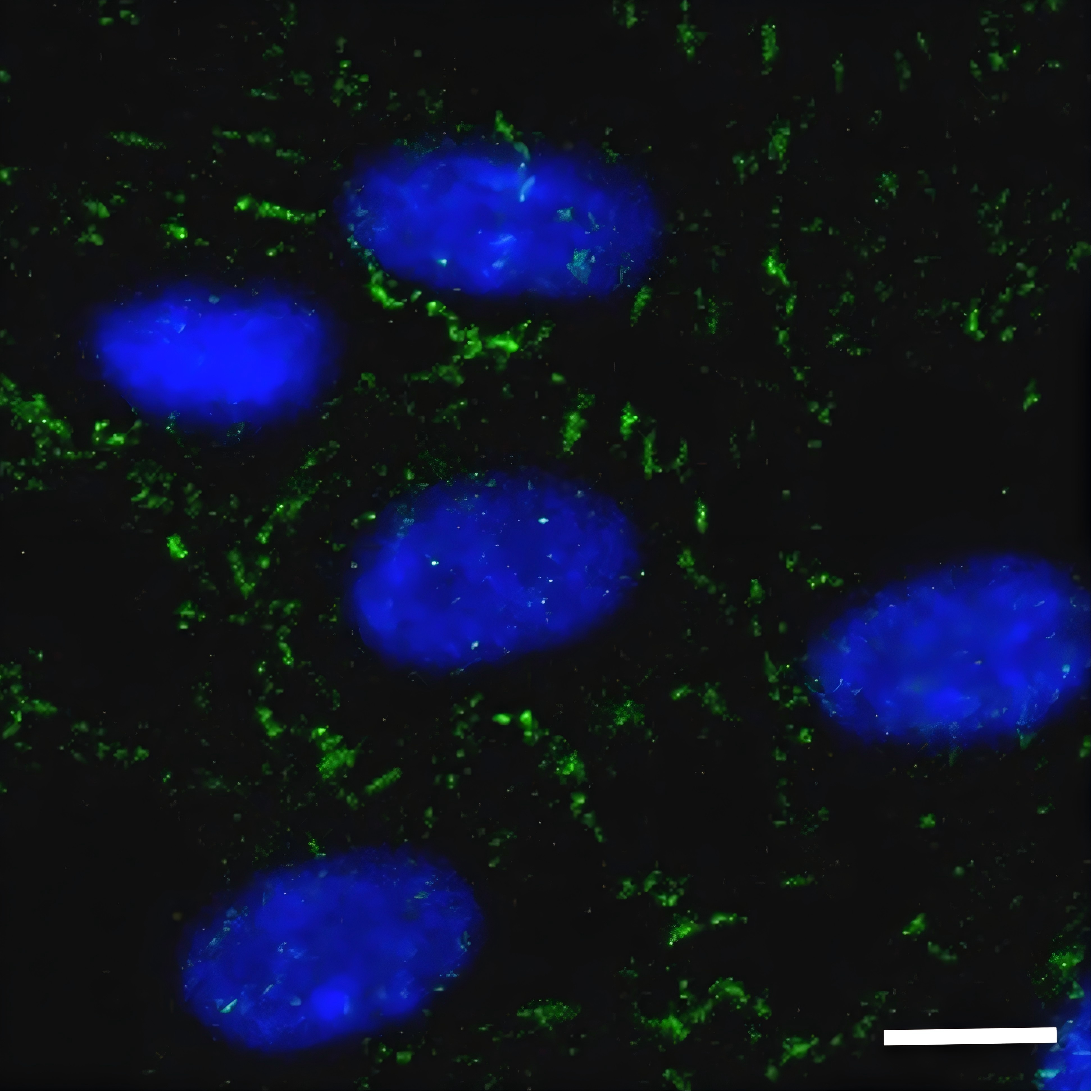

Supplement: Supplementary file 4 — Source data Fig. 2 [file 44321_2026_452_MOESM4_ESM.zip › Figure 2/2I-J/sTREM2 Merge.tif]

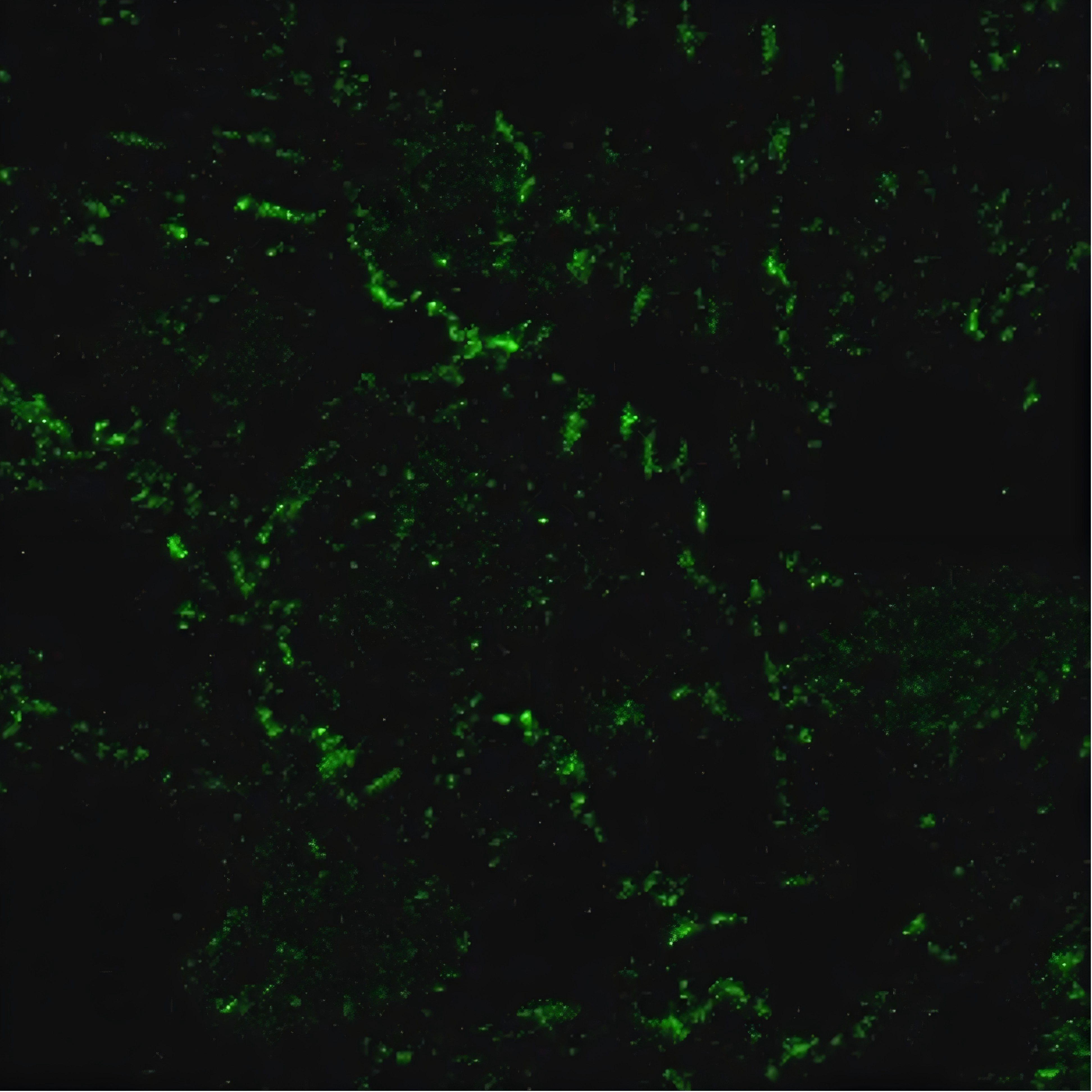

Supplement: Supplementary file 4 — Source data Fig. 2 [file 44321_2026_452_MOESM4_ESM.zip › Figure 2/2I-J/sTREM2 p-VEcad.tif]

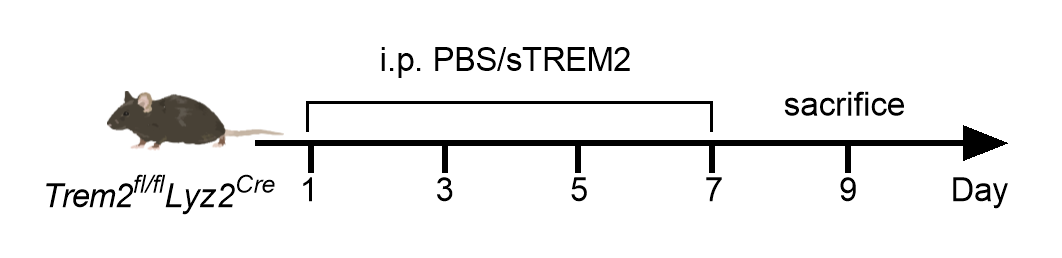

Supplement: Supplementary file 4 — Source data Fig. 2 [file 44321_2026_452_MOESM4_ESM.zip › Figure 2/2K-L/Fig2K.tif]

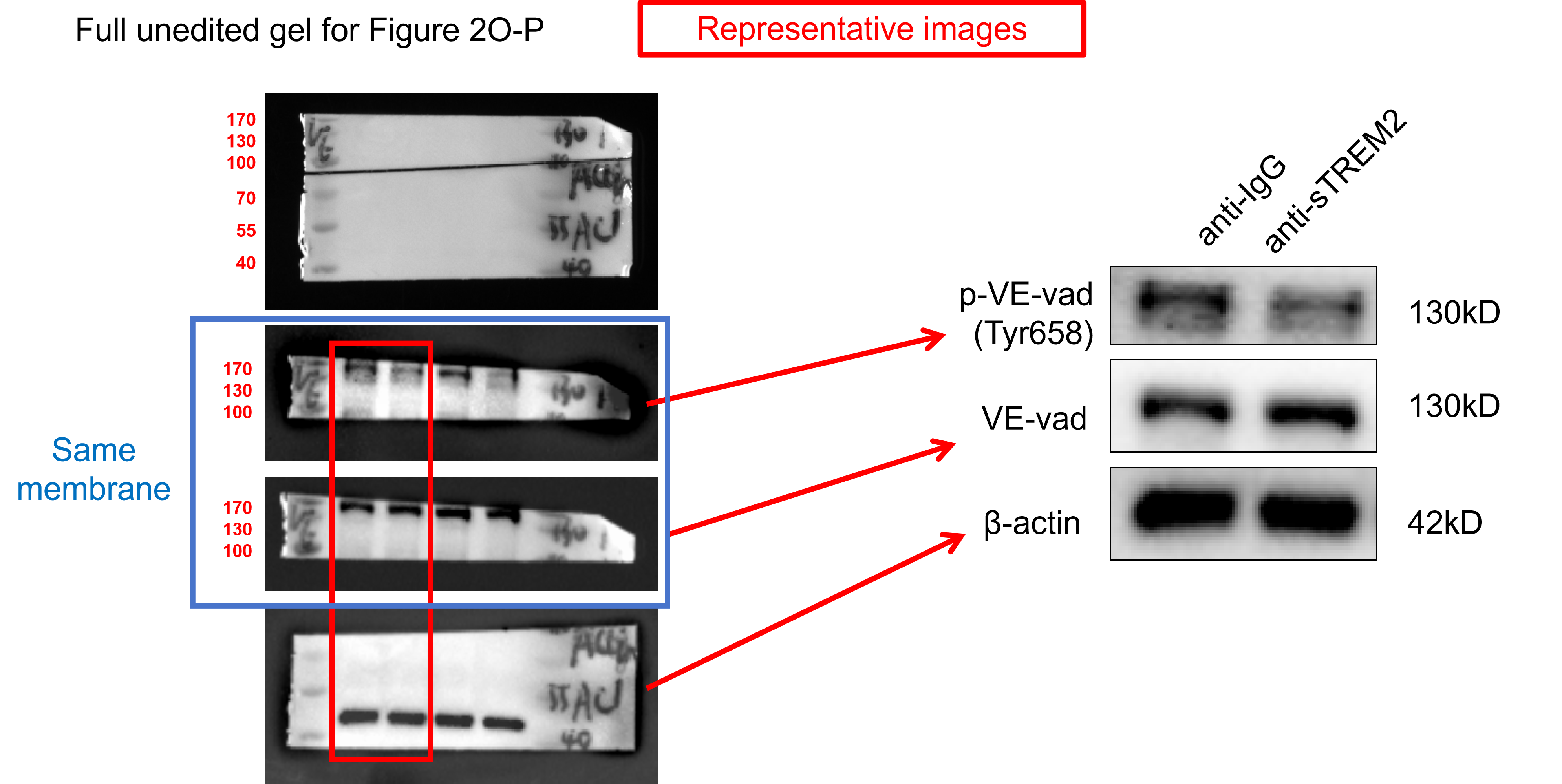

Supplement: Supplementary file 4 — Source data Fig. 2 [file 44321_2026_452_MOESM4_ESM.zip › Figure 2/2O-P/Instructions for cropping Western blot images 1.tif]

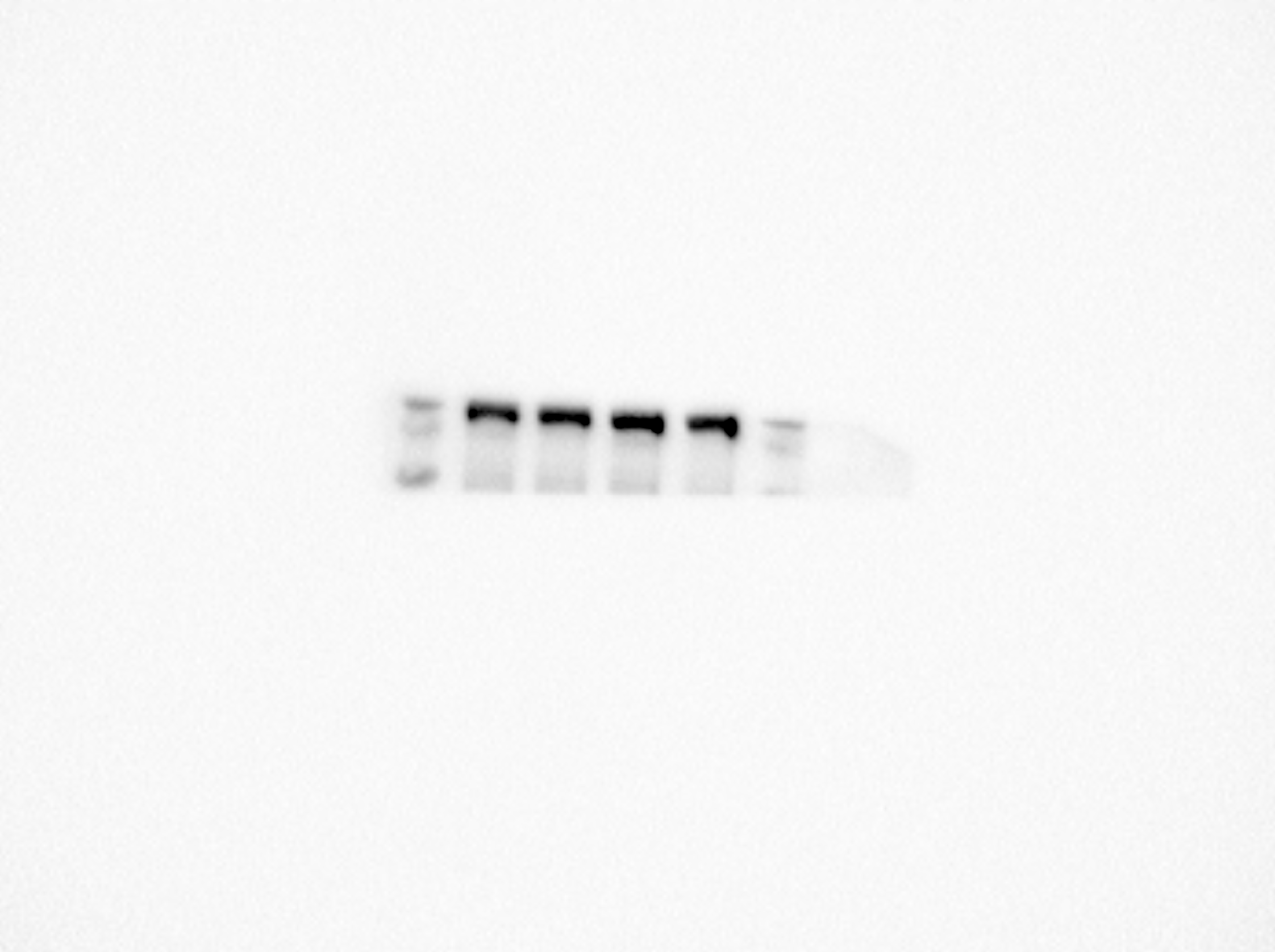

Supplement: Supplementary file 4 — Source data Fig. 2 [file 44321_2026_452_MOESM4_ESM.zip › Figure 2/2O-P/WB_ Uncropped blots_ VEcad.tif]

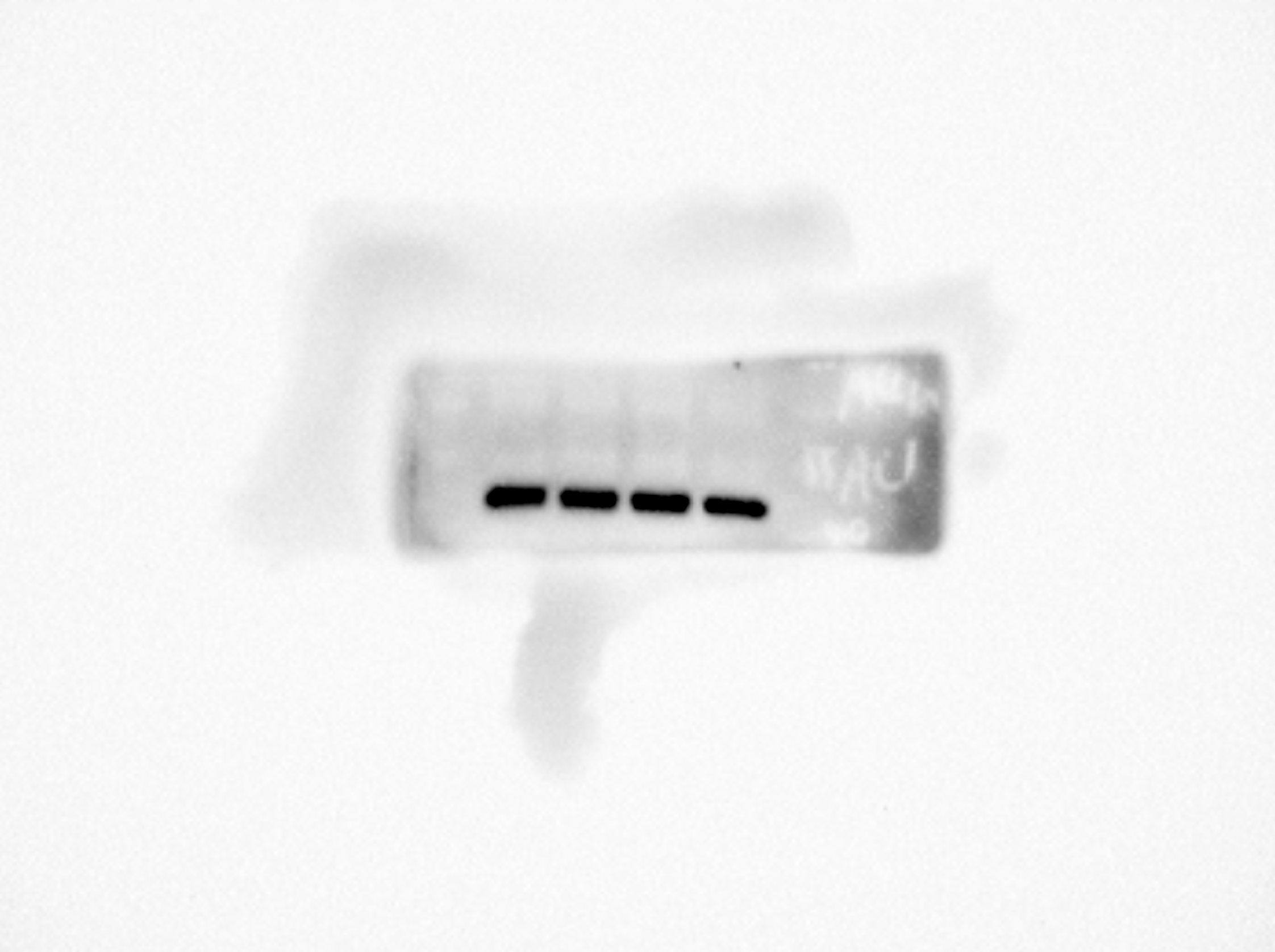

Supplement: Supplementary file 4 — Source data Fig. 2 [file 44321_2026_452_MOESM4_ESM.zip › Figure 2/2O-P/WB_ Uncropped blots_ β-actin.tif]

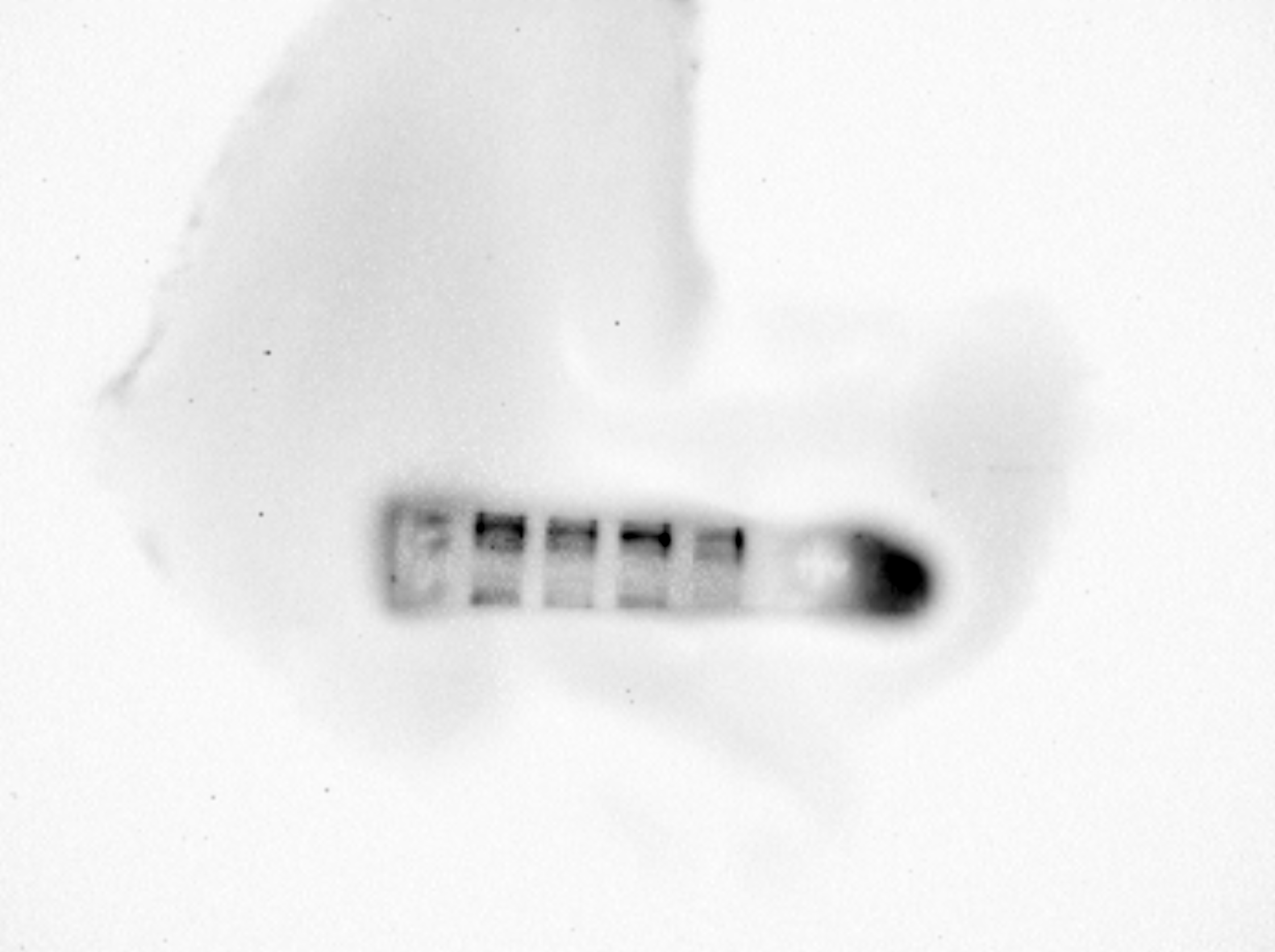

Supplement: Supplementary file 4 — Source data Fig. 2 [file 44321_2026_452_MOESM4_ESM.zip › Figure 2/2O-P/WB_ Uncropped blots_p-VEcad.tif]

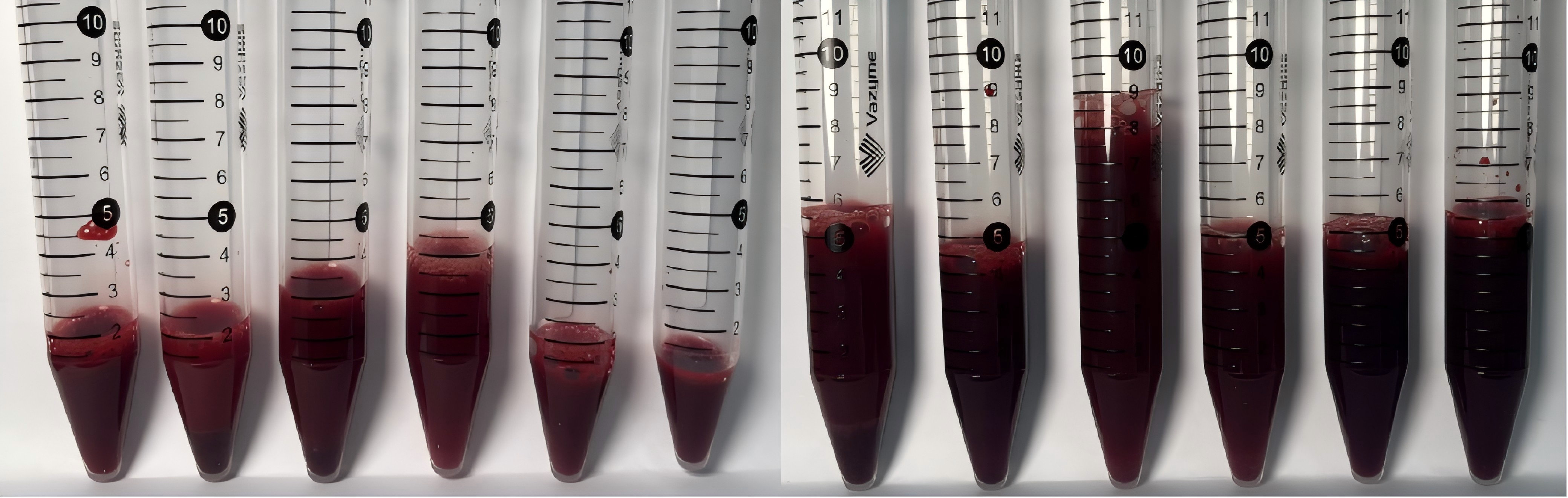

Supplement: Supplementary file 5 — Source data Fig. 3 [file 44321_2026_452_MOESM5_ESM.zip › Figure 3/3A-D/Figure 3A ascites volume.tif]

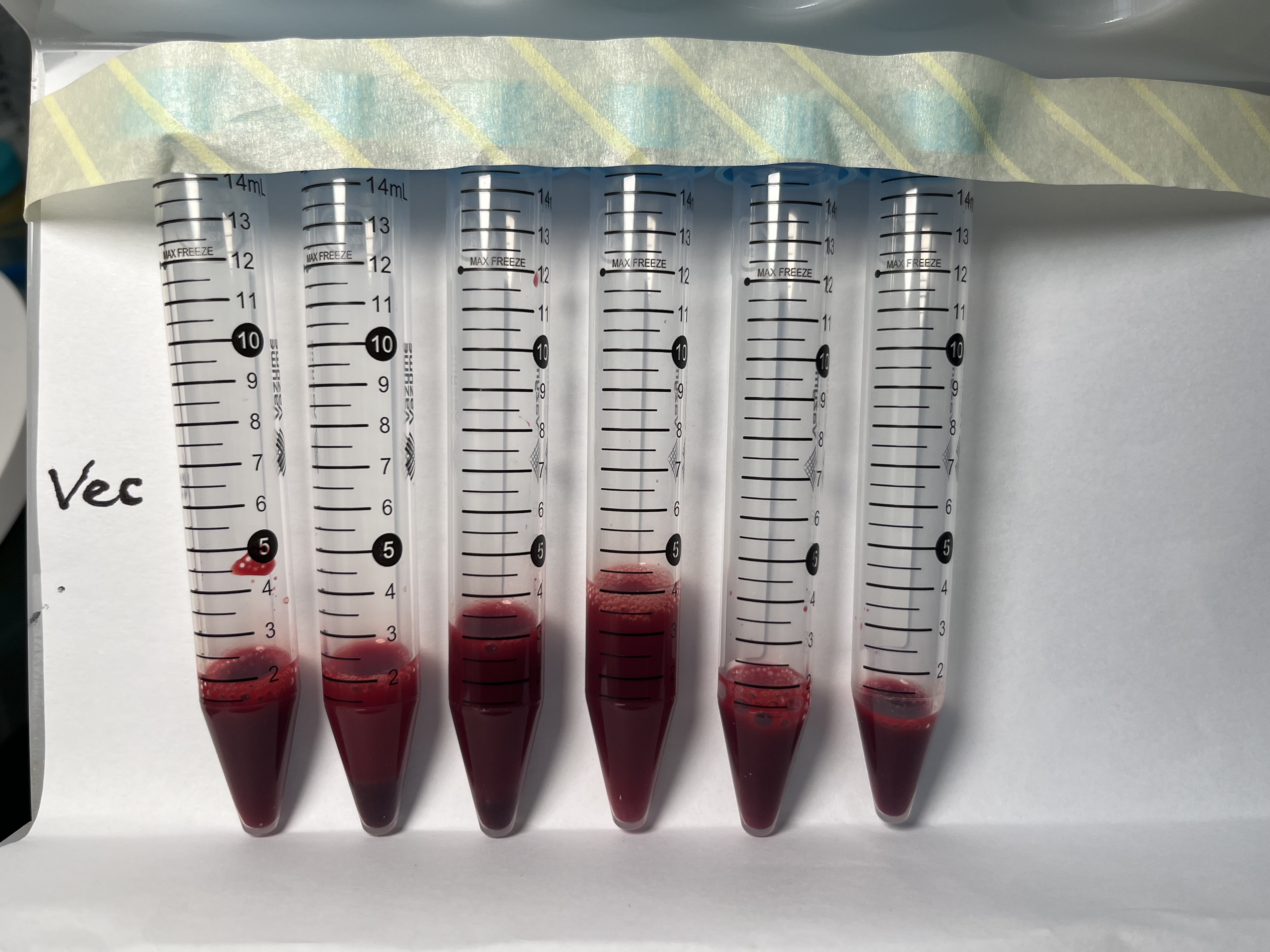

Supplement: Supplementary file 5 — Source data Fig. 3 [file 44321_2026_452_MOESM5_ESM.zip › Figure 3/3A-D/Figure 3A(left) OE-Ctrl.jpg]

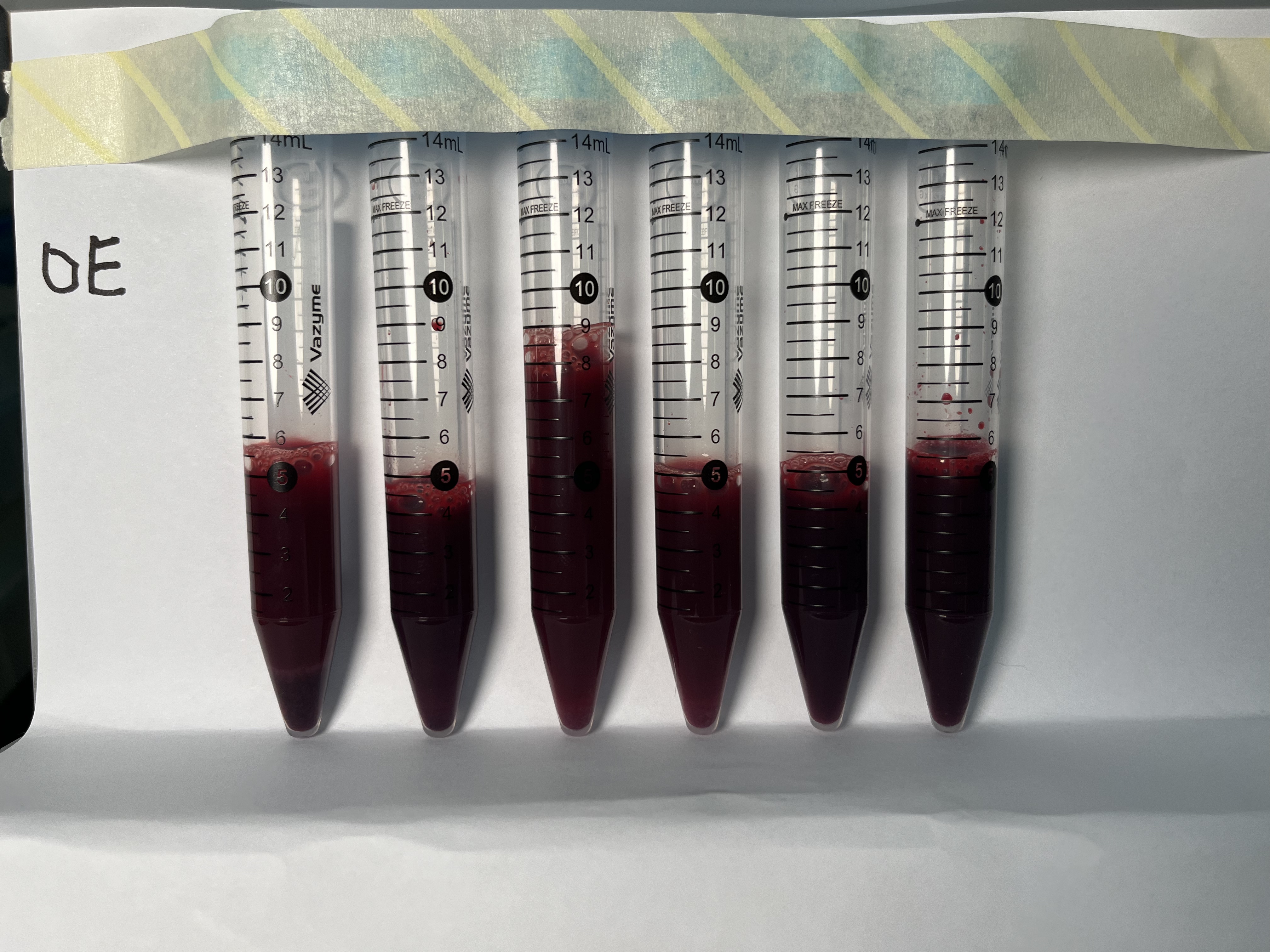

Supplement: Supplementary file 5 — Source data Fig. 3 [file 44321_2026_452_MOESM5_ESM.zip › Figure 3/3A-D/Figure 3A(right) OE-TREM2.jpg]

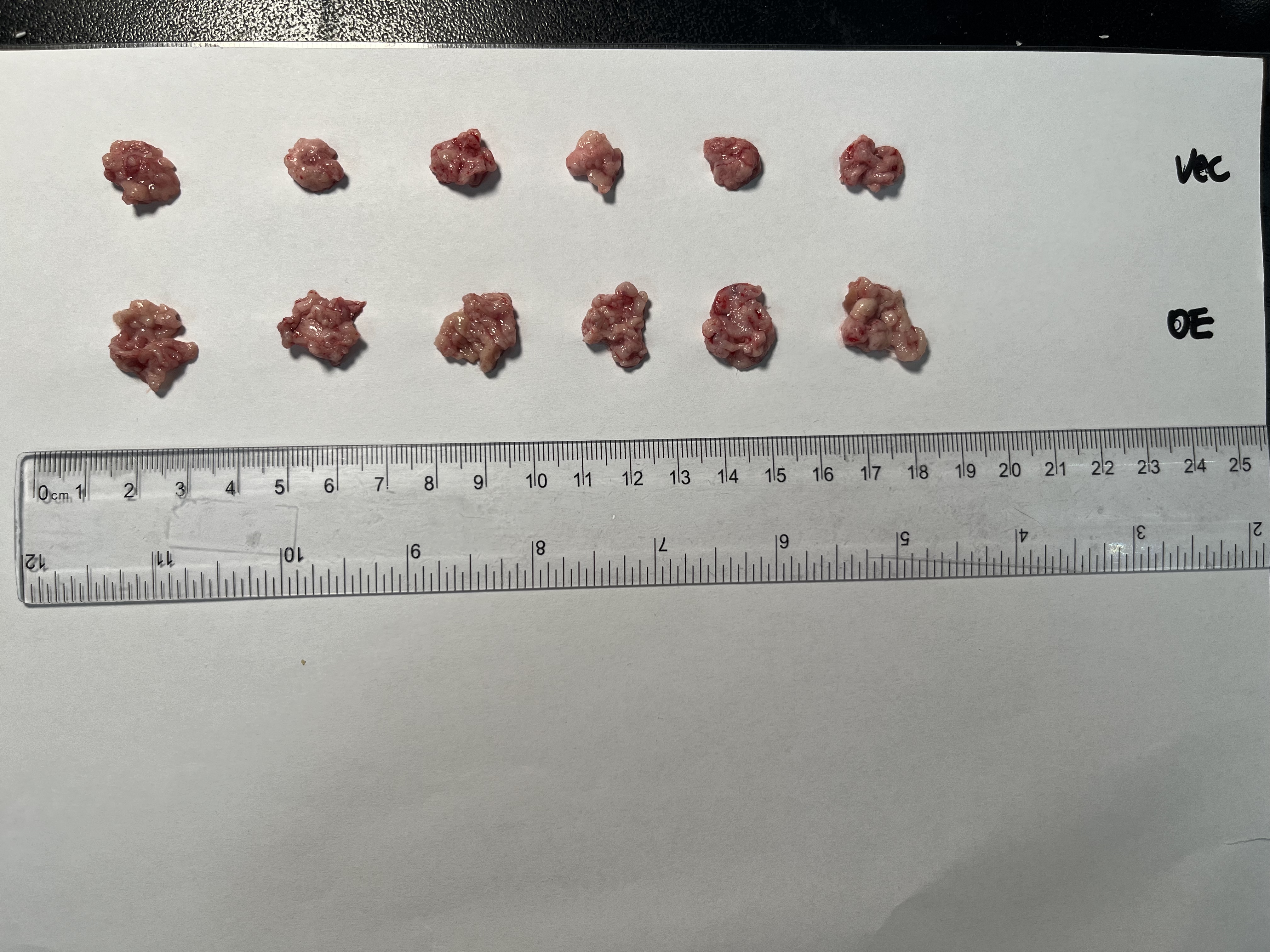

Supplement: Supplementary file 5 — Source data Fig. 3 [file 44321_2026_452_MOESM5_ESM.zip › Figure 3/3A-D/Figure 3B tumor burden (RAW DATA).jpg]

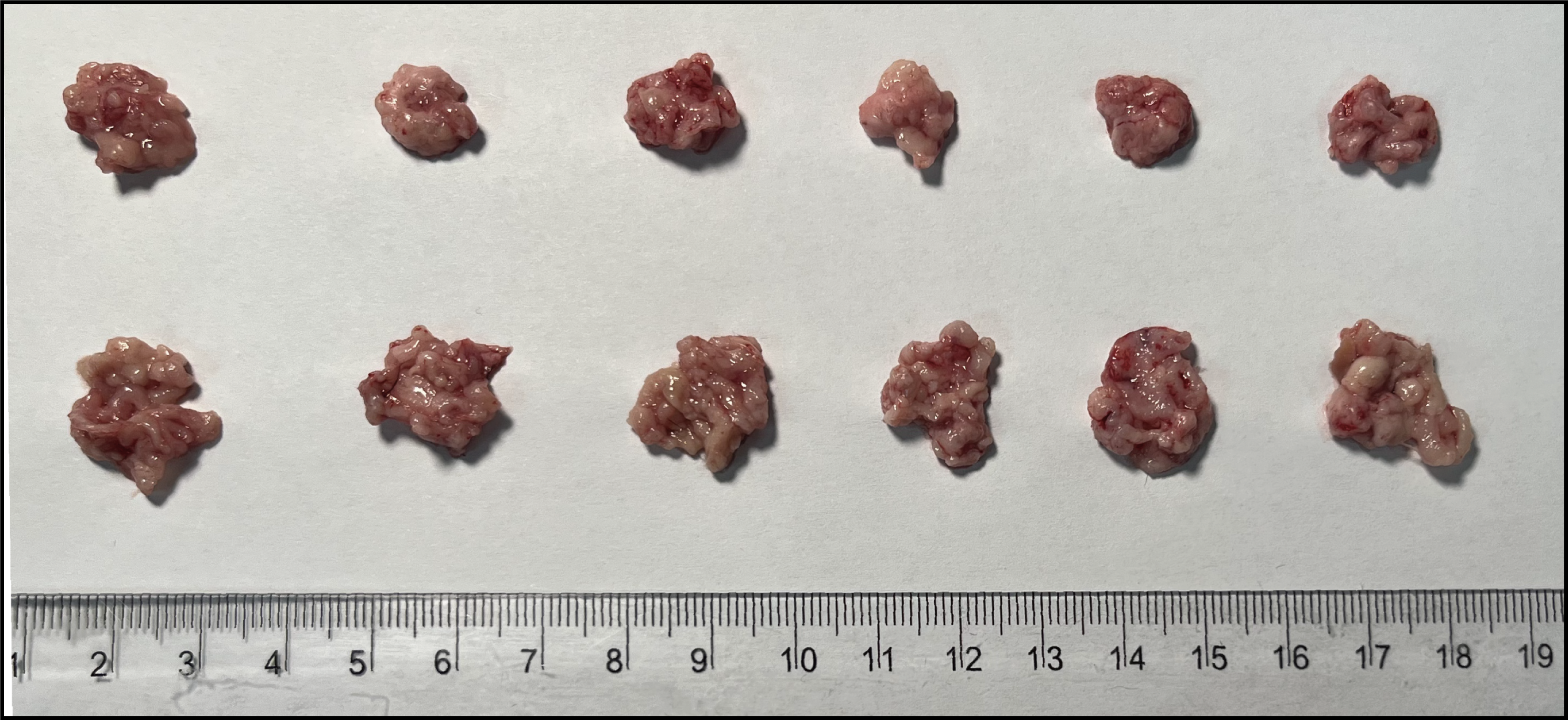

Supplement: Supplementary file 5 — Source data Fig. 3 [file 44321_2026_452_MOESM5_ESM.zip › Figure 3/3A-D/Figure 3B tumor burden.tif]

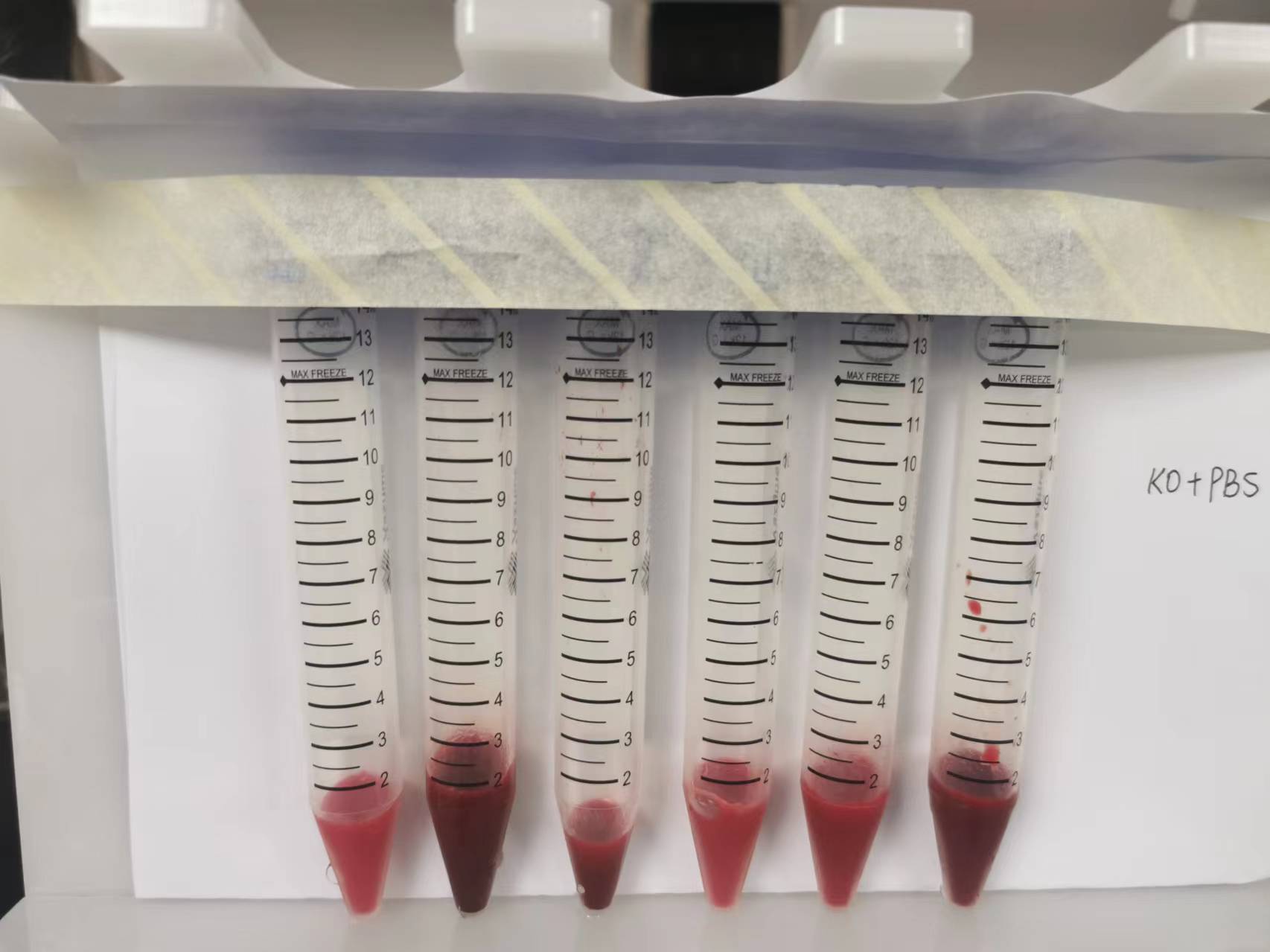

Supplement: Supplementary file 5 — Source data Fig. 3 [file 44321_2026_452_MOESM5_ESM.zip › Figure 3/3G-J/Figure 3G (left).jpg]

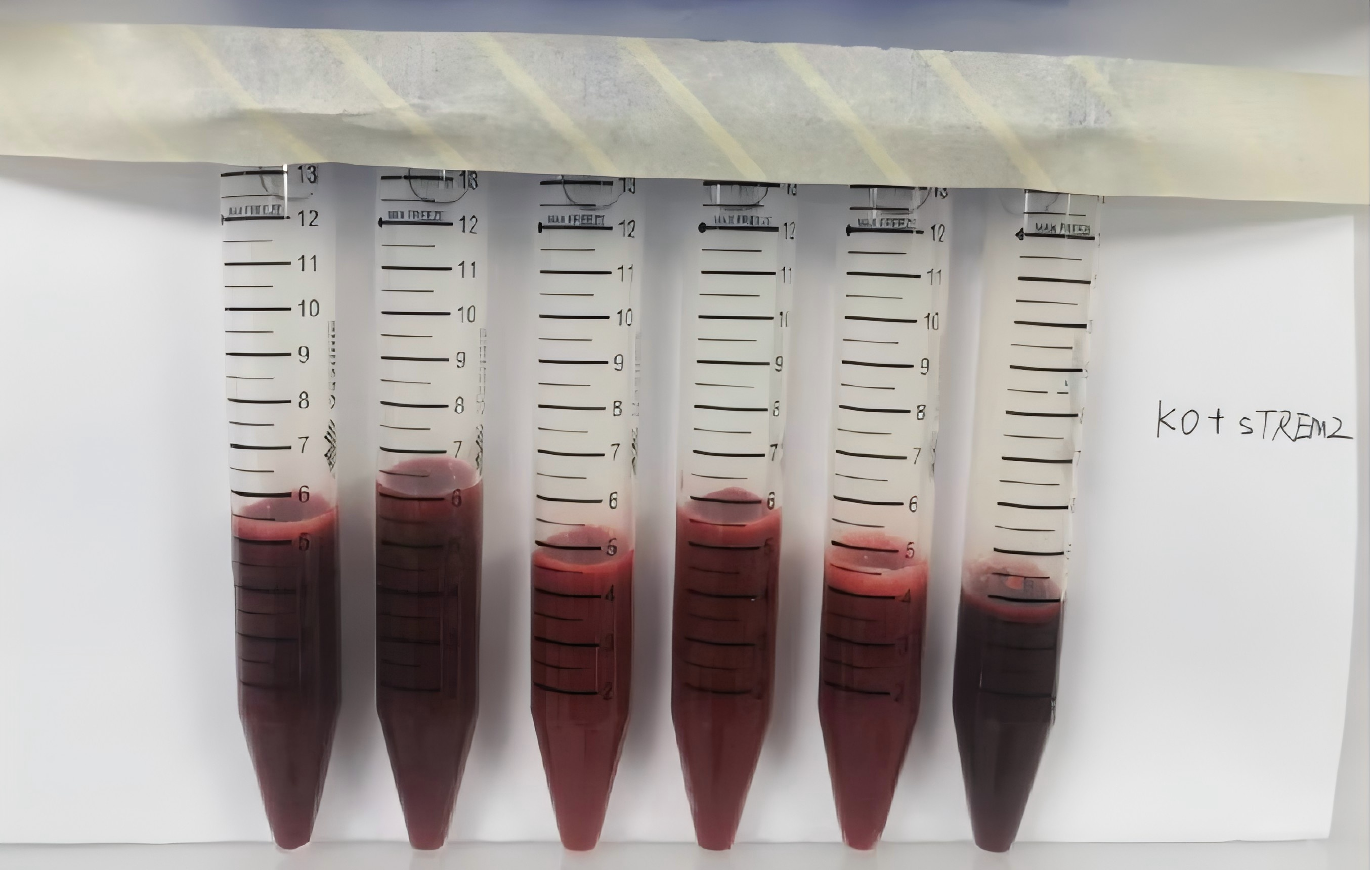

Supplement: Supplementary file 5 — Source data Fig. 3 [file 44321_2026_452_MOESM5_ESM.zip › Figure 3/3G-J/Figure 3G (right).png]

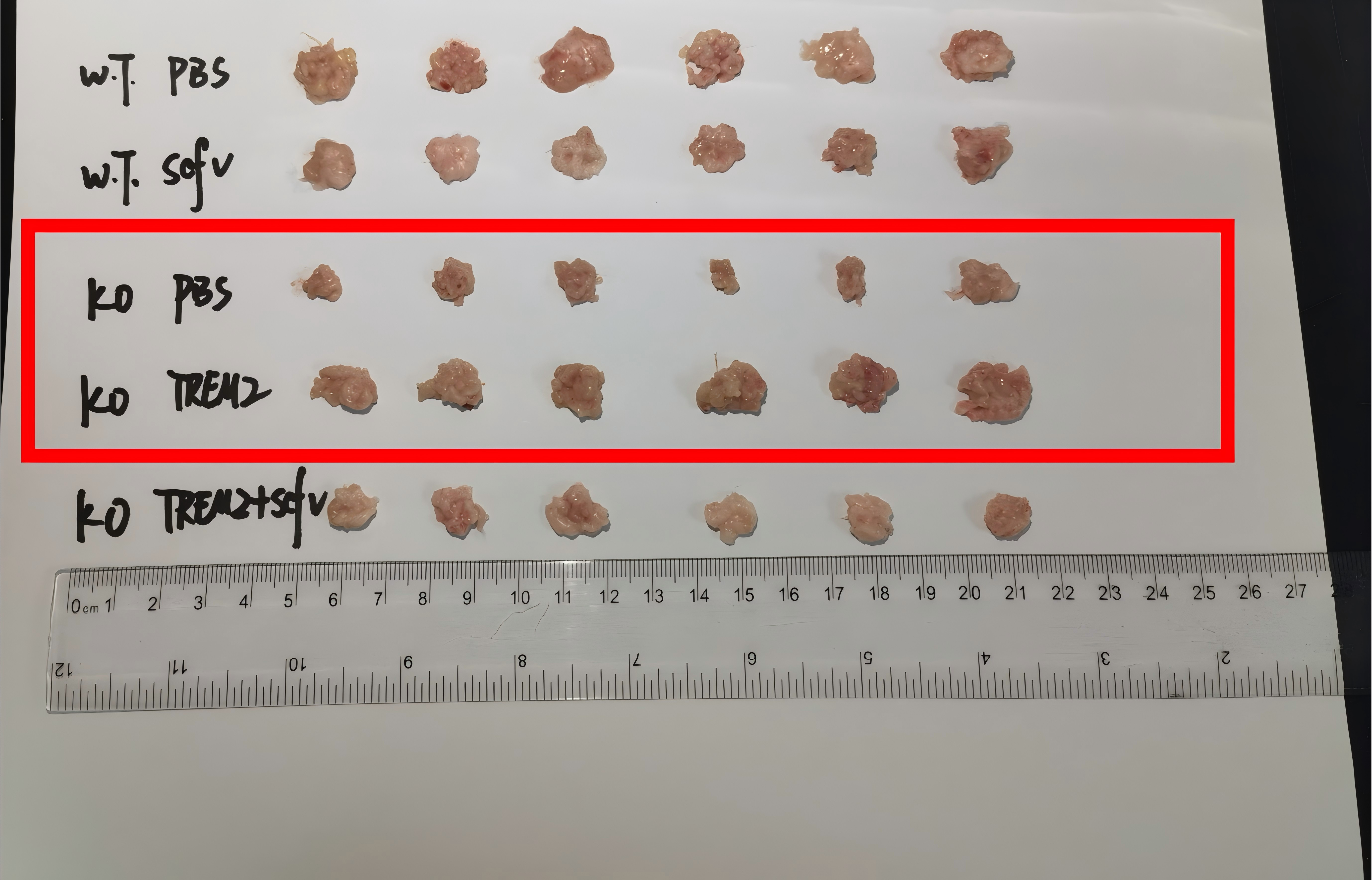

Supplement: Supplementary file 5 — Source data Fig. 3 [file 44321_2026_452_MOESM5_ESM.zip › Figure 3/3G-J/Figure 3H (RAW DATA).png]

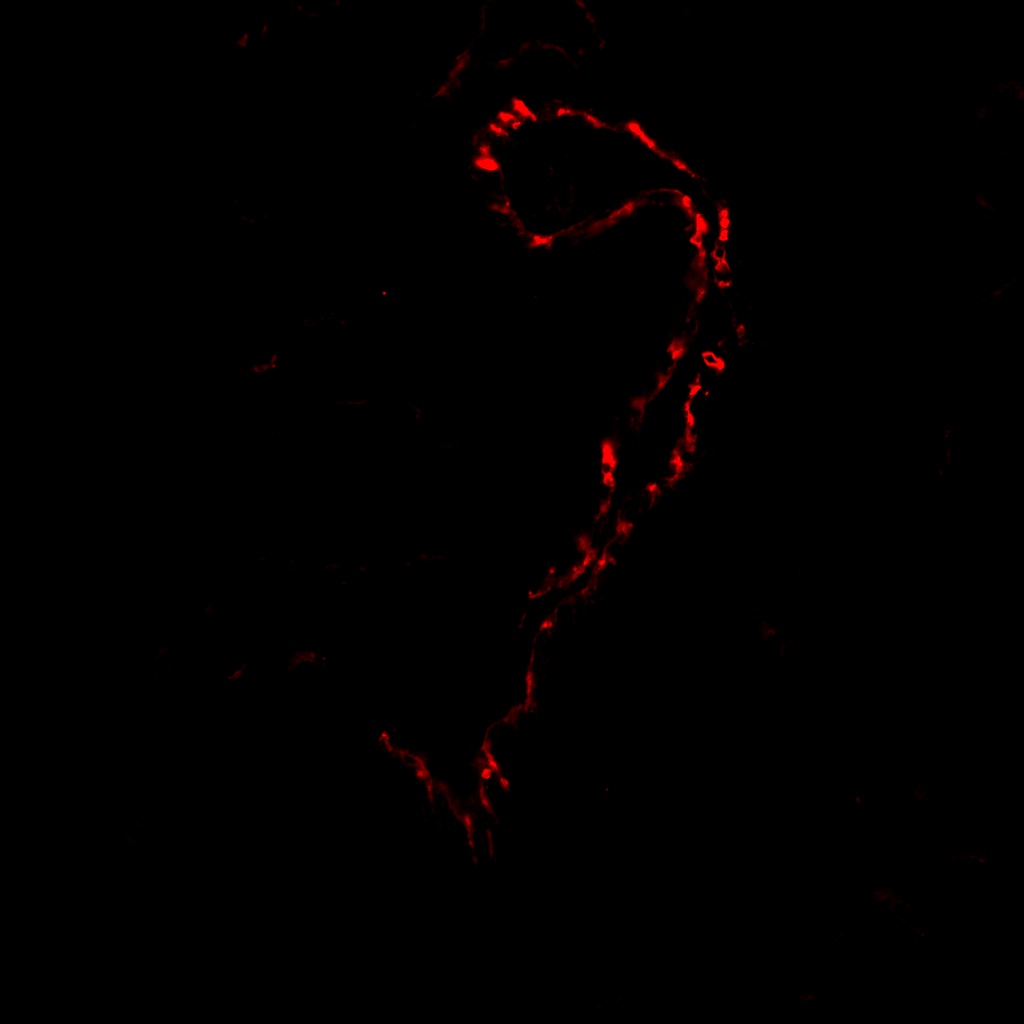

Supplement: Supplementary file 5 — Source data Fig. 3 [file 44321_2026_452_MOESM5_ESM.zip › Figure 3/3M-N/IF PBS CD31.jpg]

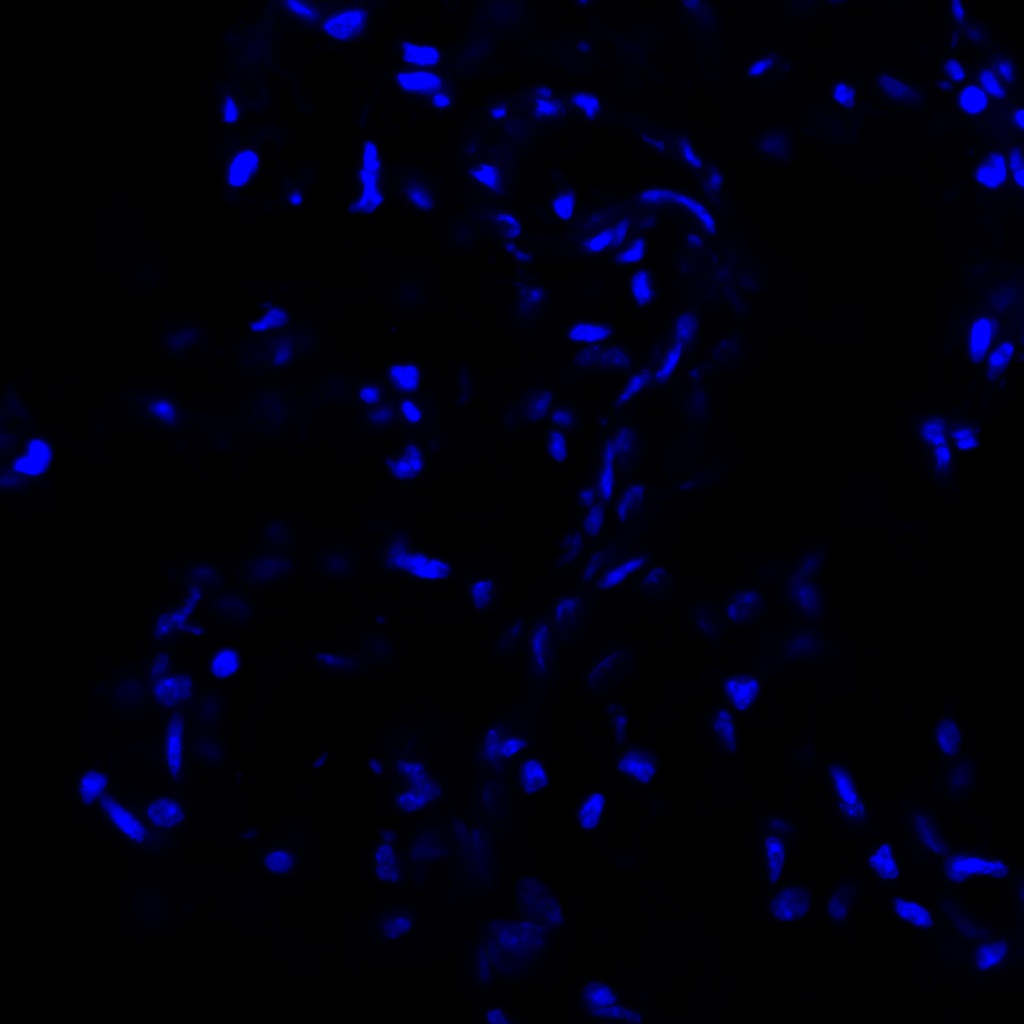

Supplement: Supplementary file 5 — Source data Fig. 3 [file 44321_2026_452_MOESM5_ESM.zip › Figure 3/3M-N/IF PBS DAPI.jpg]

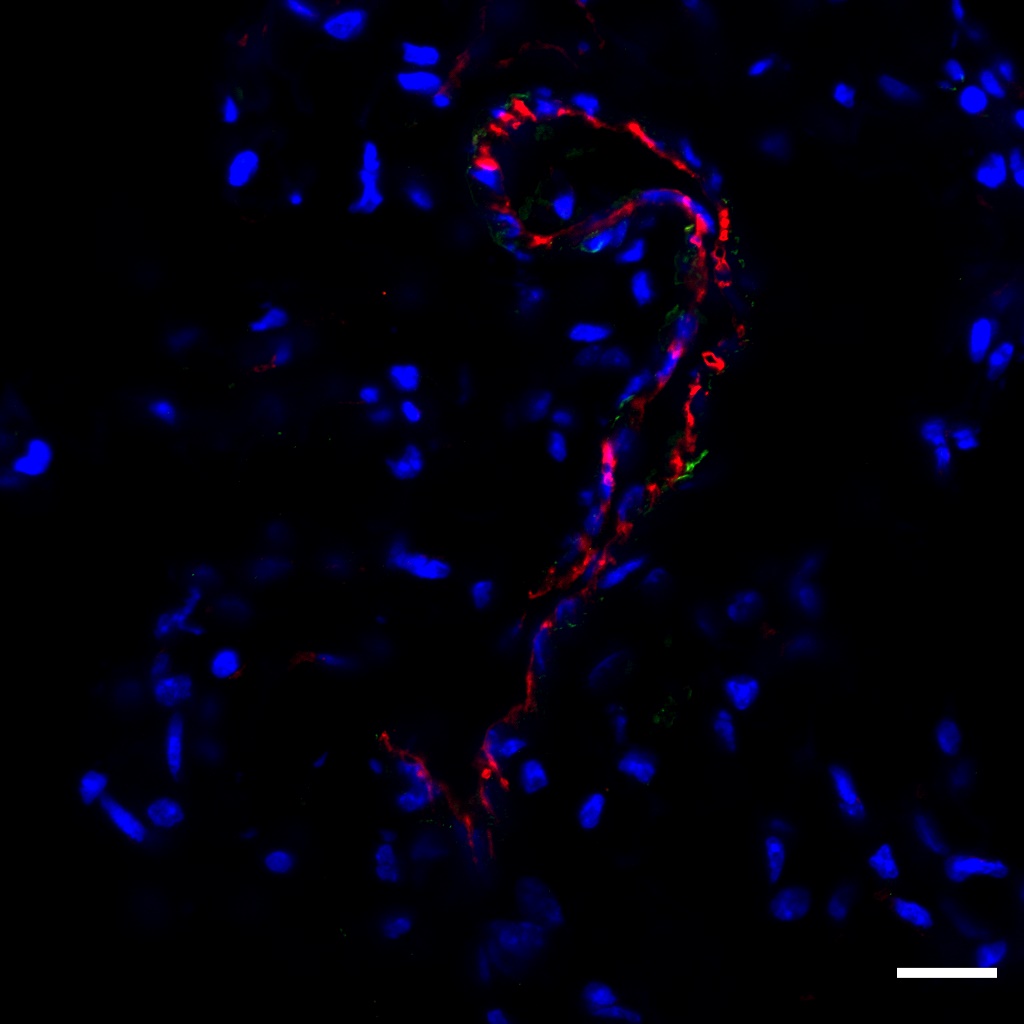

Supplement: Supplementary file 5 — Source data Fig. 3 [file 44321_2026_452_MOESM5_ESM.zip › Figure 3/3M-N/IF PBS merge.jpg]

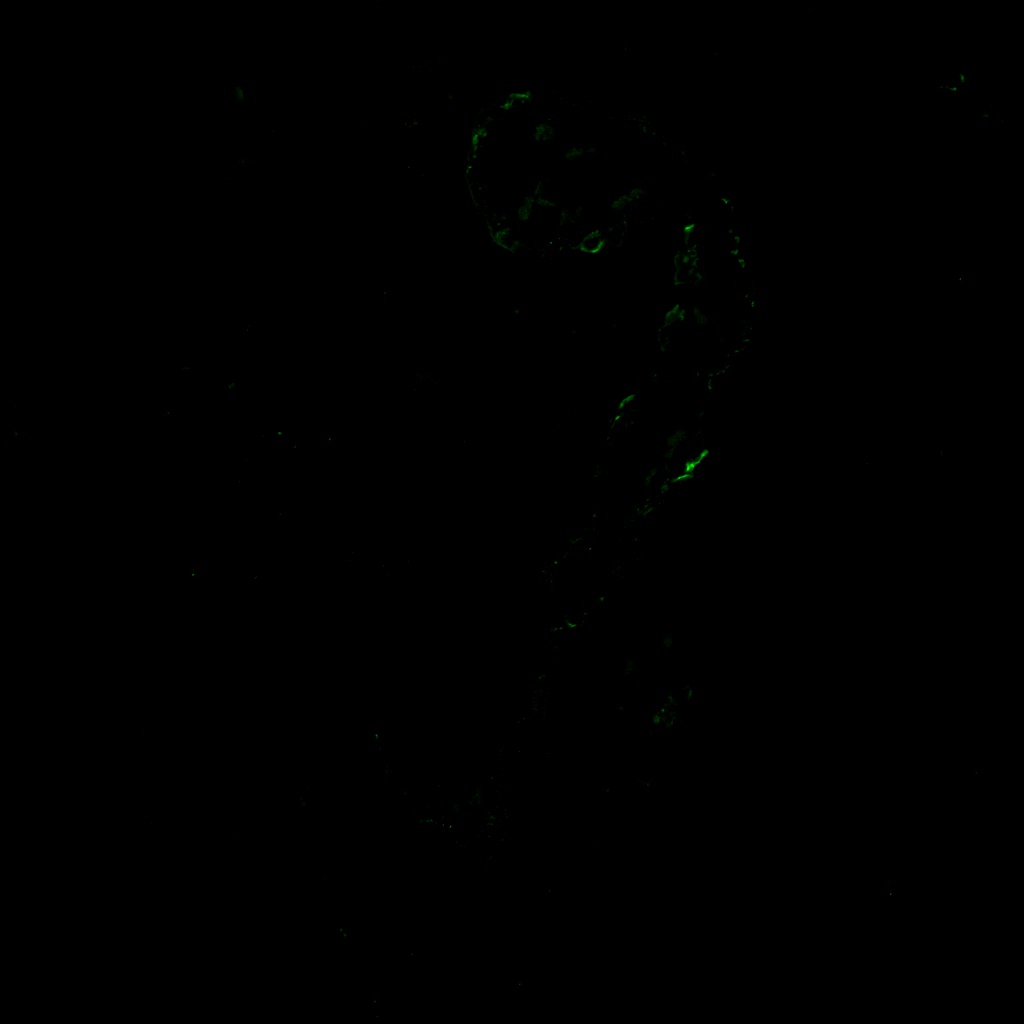

Supplement: Supplementary file 5 — Source data Fig. 3 [file 44321_2026_452_MOESM5_ESM.zip › Figure 3/3M-N/IF PBS p-VE-cad.jpg]

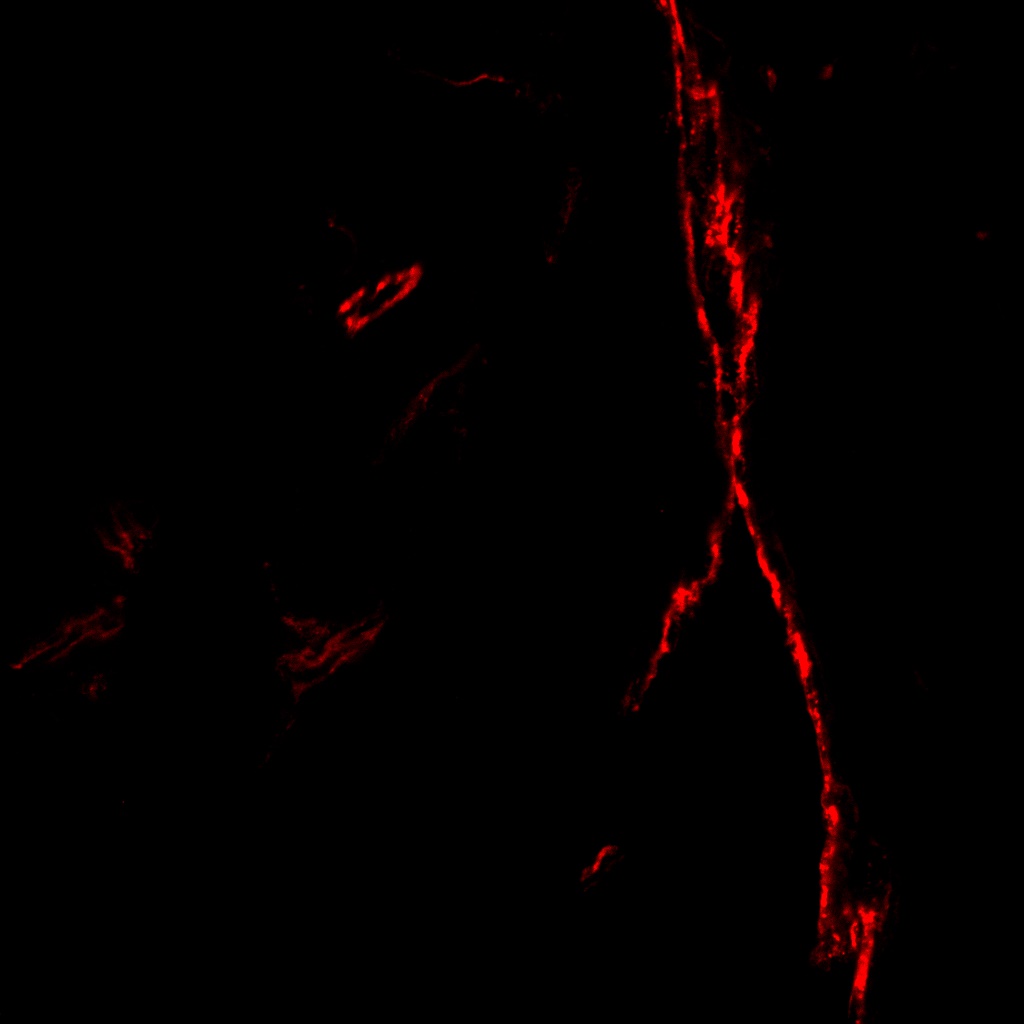

Supplement: Supplementary file 5 — Source data Fig. 3 [file 44321_2026_452_MOESM5_ESM.zip › Figure 3/3M-N/IF sTREM2 CD31.jpg]

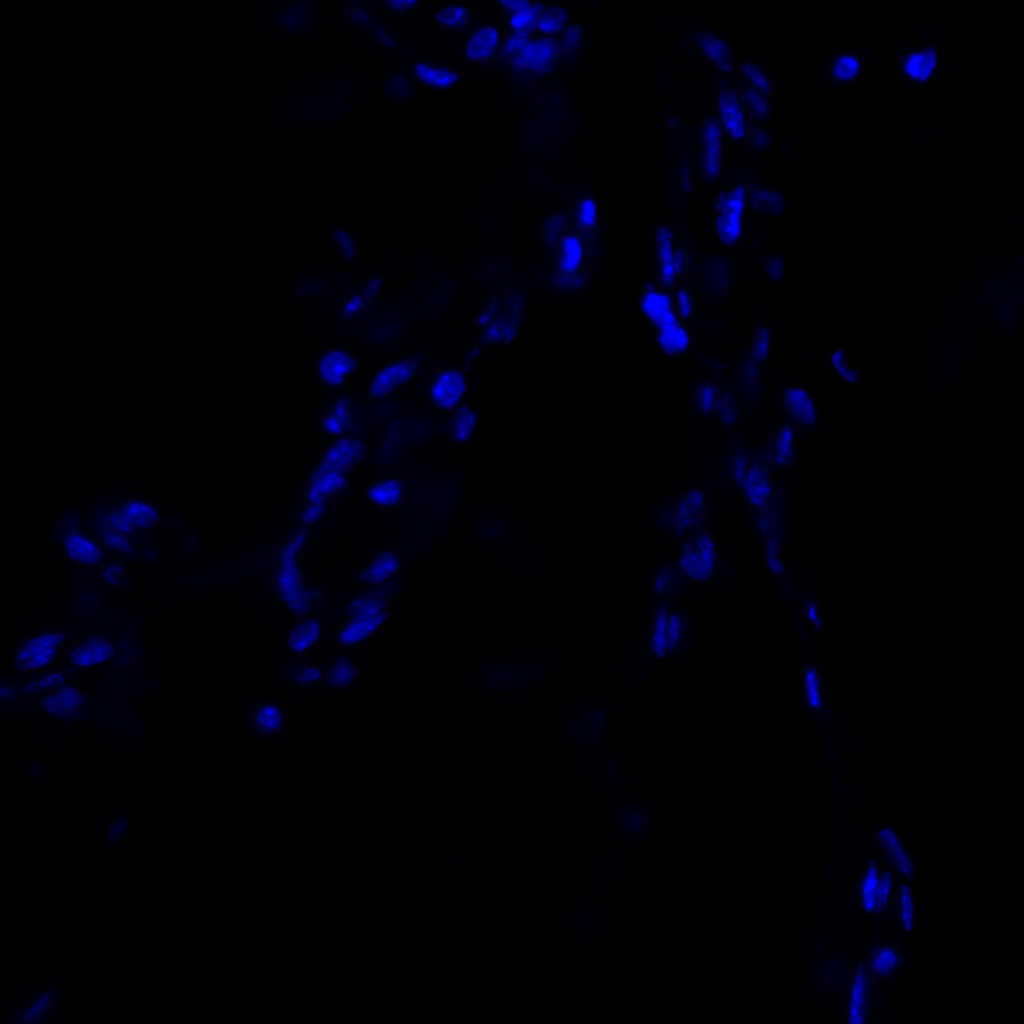

Supplement: Supplementary file 5 — Source data Fig. 3 [file 44321_2026_452_MOESM5_ESM.zip › Figure 3/3M-N/IF sTREM2 DAPI.jpg]

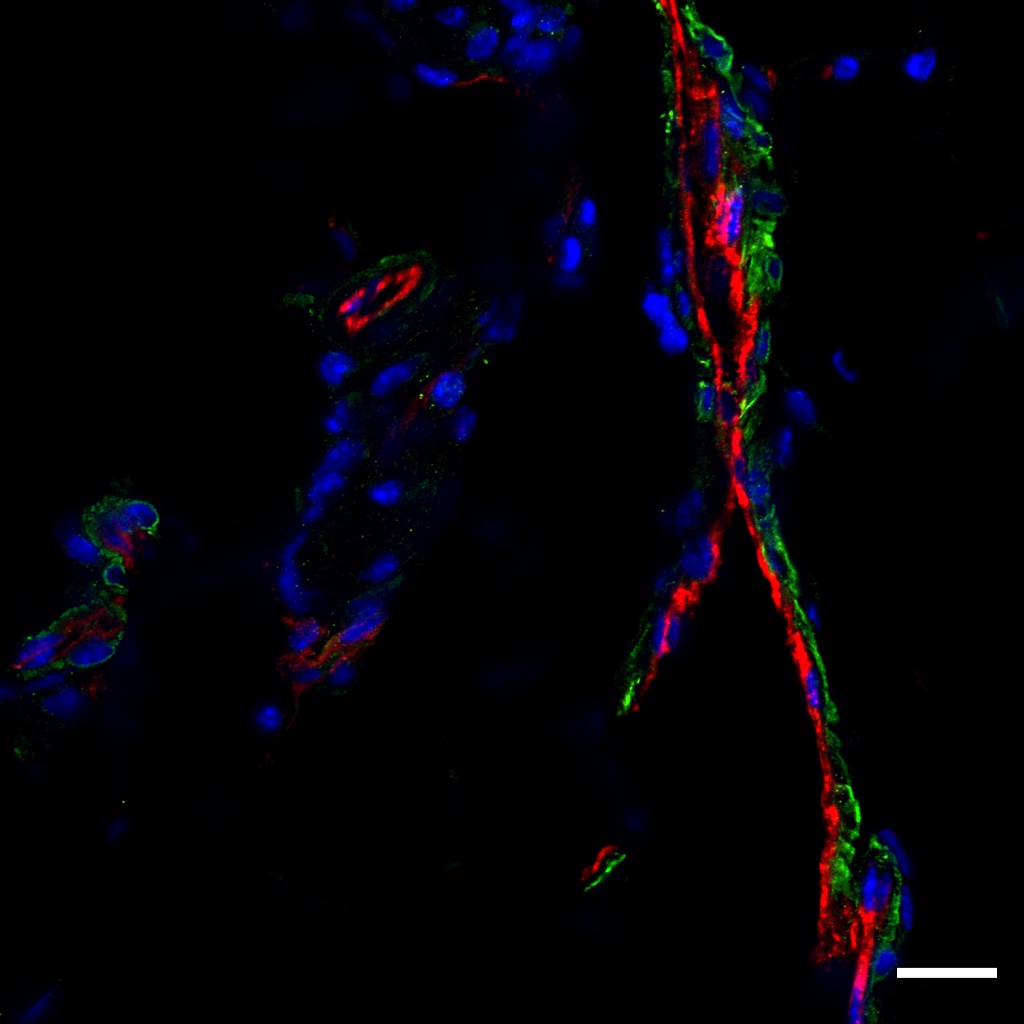

Supplement: Supplementary file 5 — Source data Fig. 3 [file 44321_2026_452_MOESM5_ESM.zip › Figure 3/3M-N/IF sTREM2 merge.jpg]

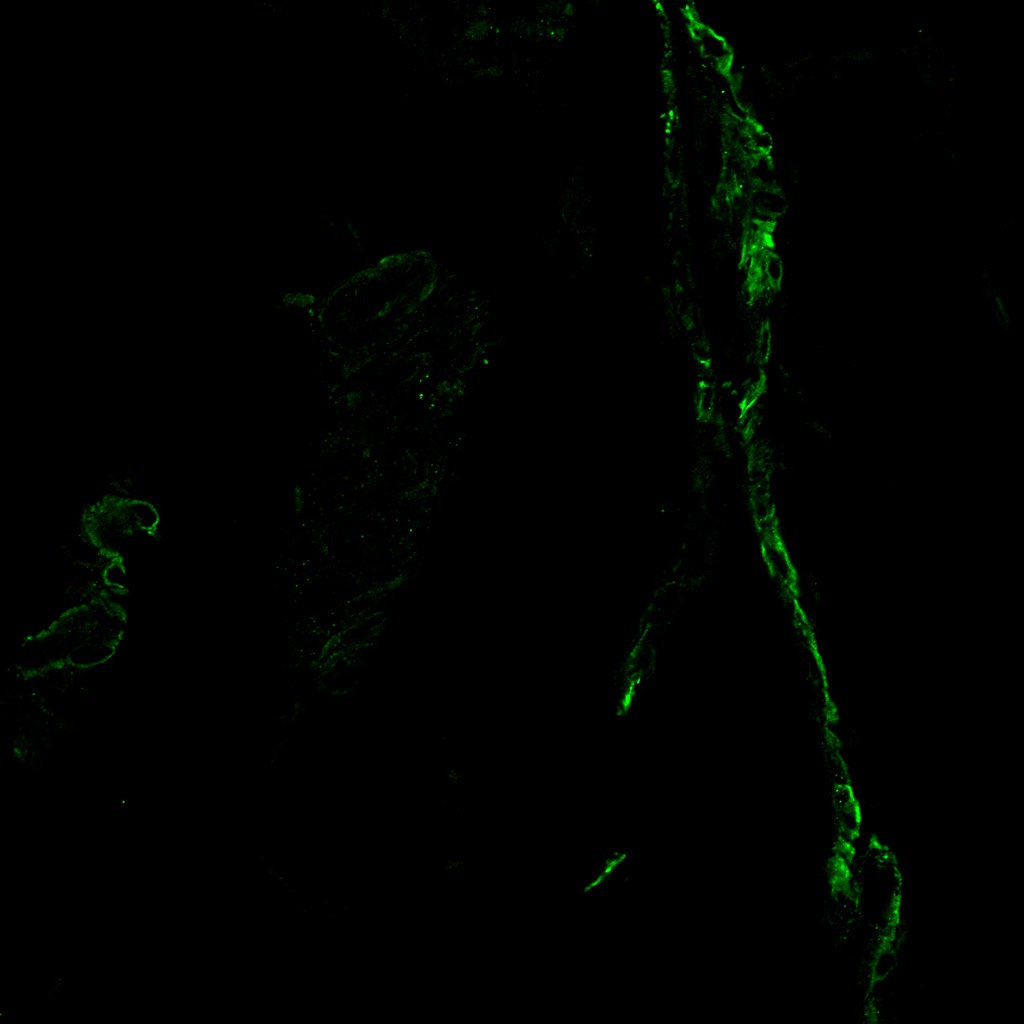

Supplement: Supplementary file 5 — Source data Fig. 3 [file 44321_2026_452_MOESM5_ESM.zip › Figure 3/3M-N/IF sTREM2 p-VE-cad.jpg]

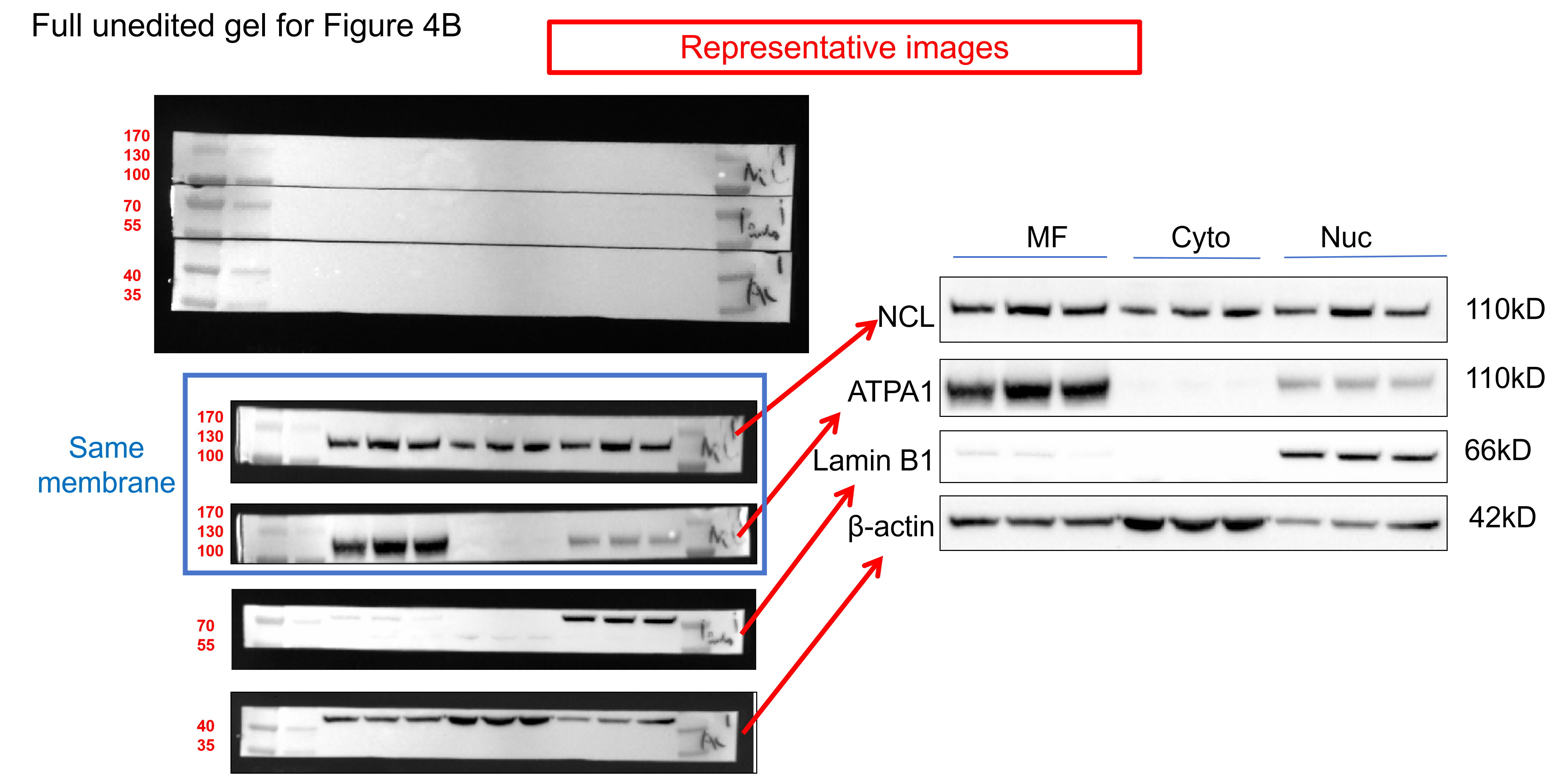

Supplement: Supplementary file 6 — Source data Fig. 4 [file 44321_2026_452_MOESM6_ESM.zip › Figure 4/4B/Instructions for cropping Western blot images 1.tif]

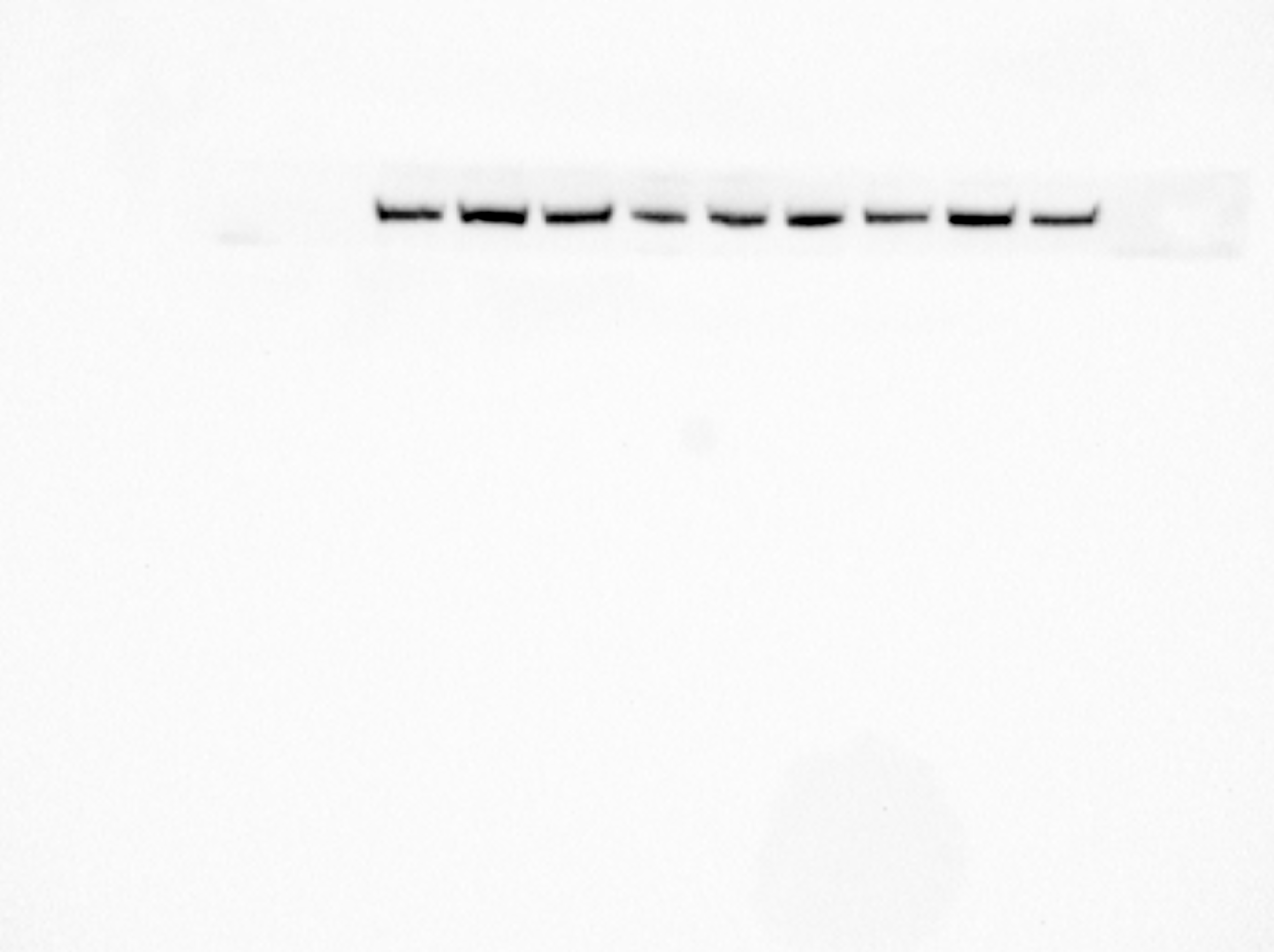

Supplement: Supplementary file 6 — Source data Fig. 4 [file 44321_2026_452_MOESM6_ESM.zip › Figure 4/4B/WB_ Uncropped blots_ NCL.tif]

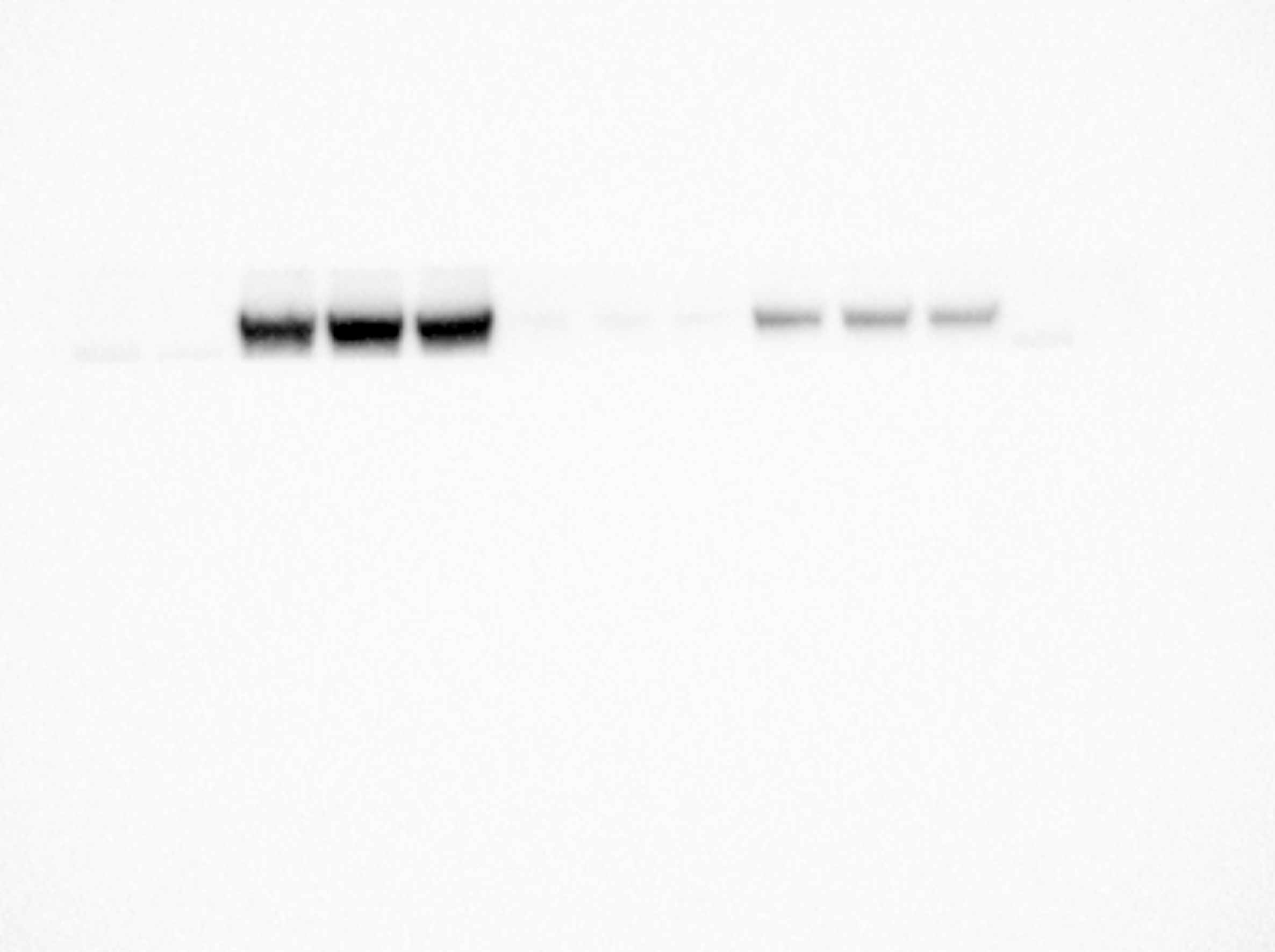

Supplement: Supplementary file 6 — Source data Fig. 4 [file 44321_2026_452_MOESM6_ESM.zip › Figure 4/4B/WB_ Uncropped blots_ ATP1A.tif]

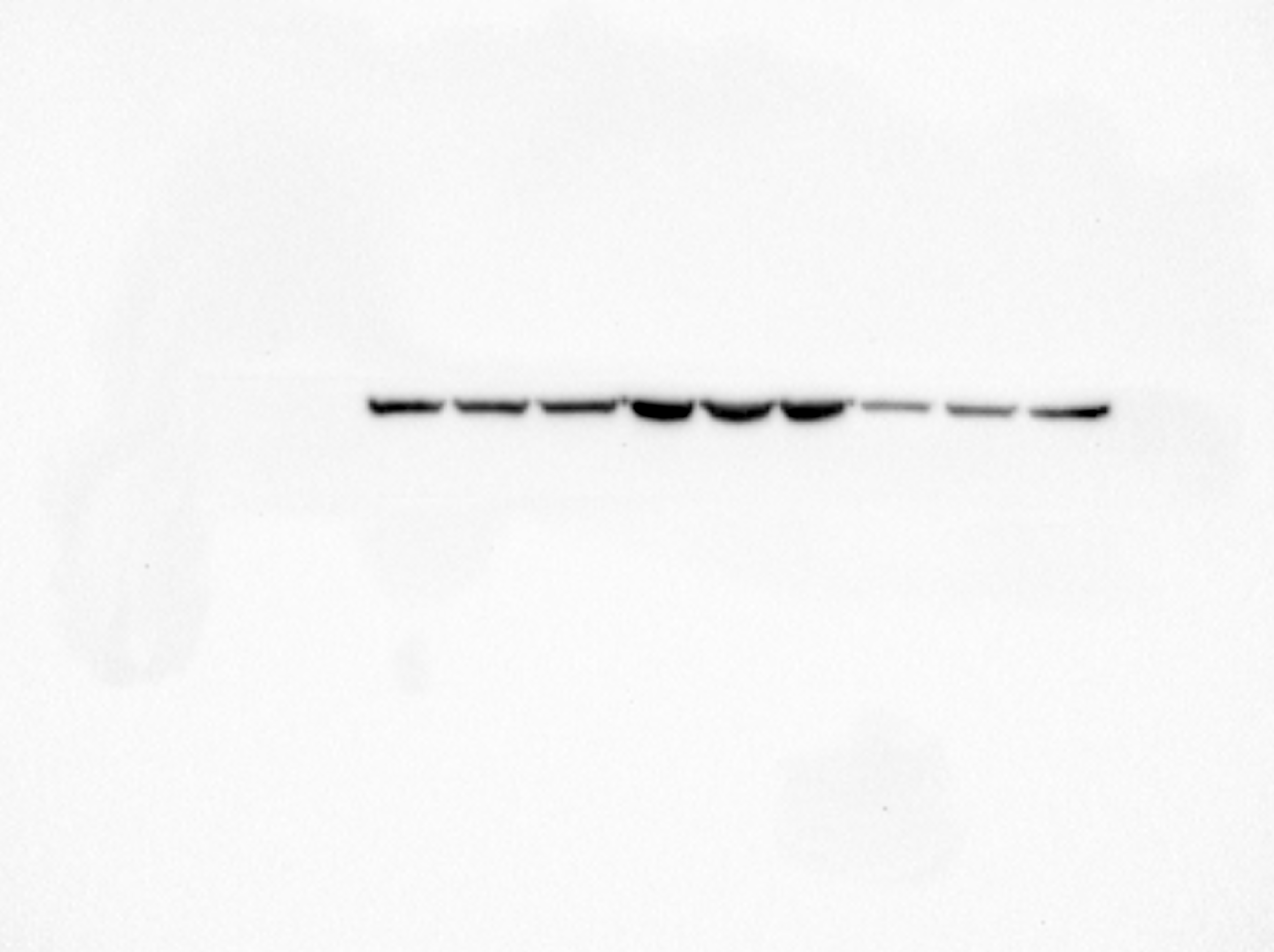

Supplement: Supplementary file 6 — Source data Fig. 4 [file 44321_2026_452_MOESM6_ESM.zip › Figure 4/4B/WB_ Uncropped blots_ β-actin.tif]

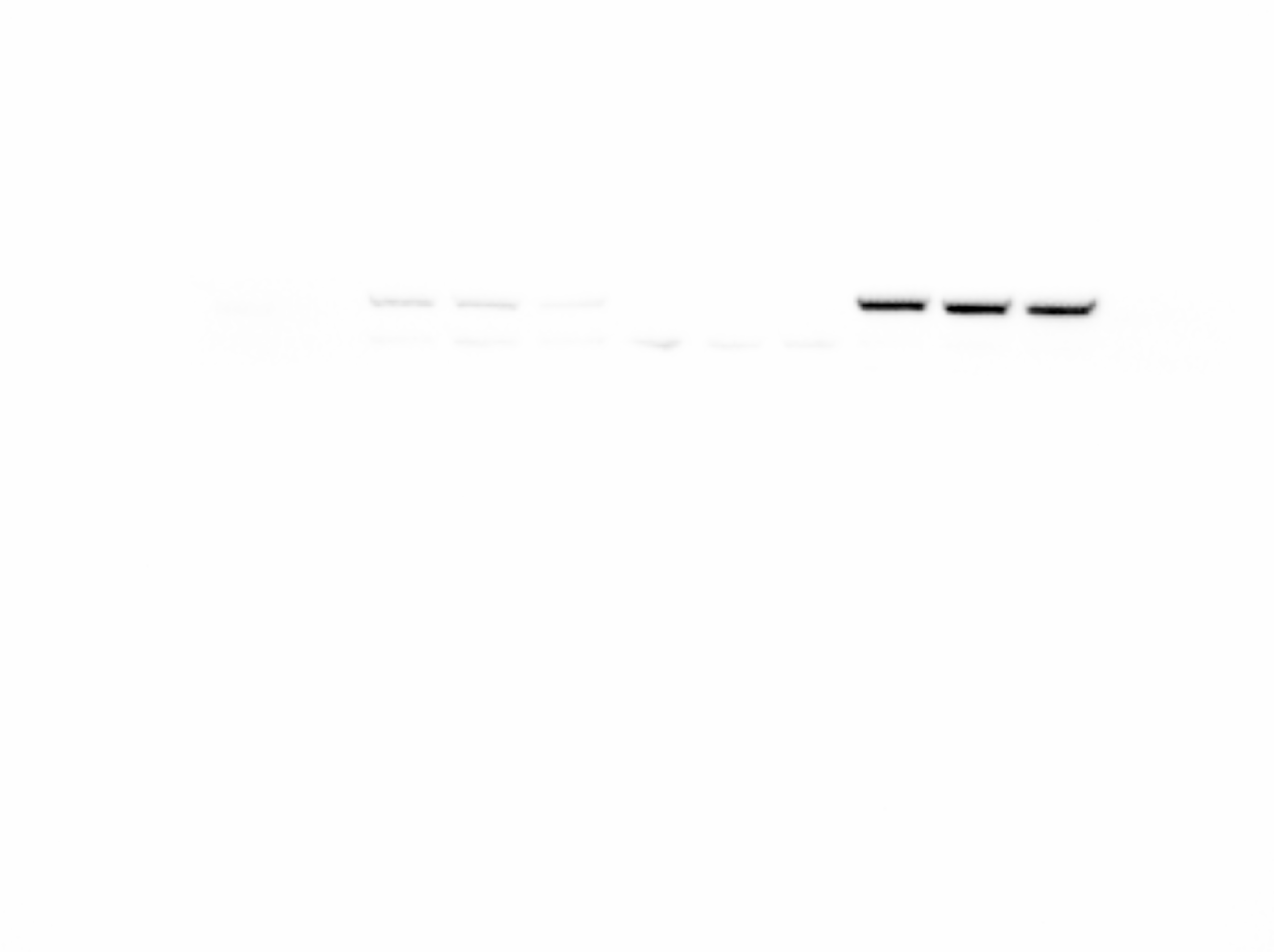

Supplement: Supplementary file 6 — Source data Fig. 4 [file 44321_2026_452_MOESM6_ESM.zip › Figure 4/4B/WB_ Uncropped blot_ Laminb1.tif]

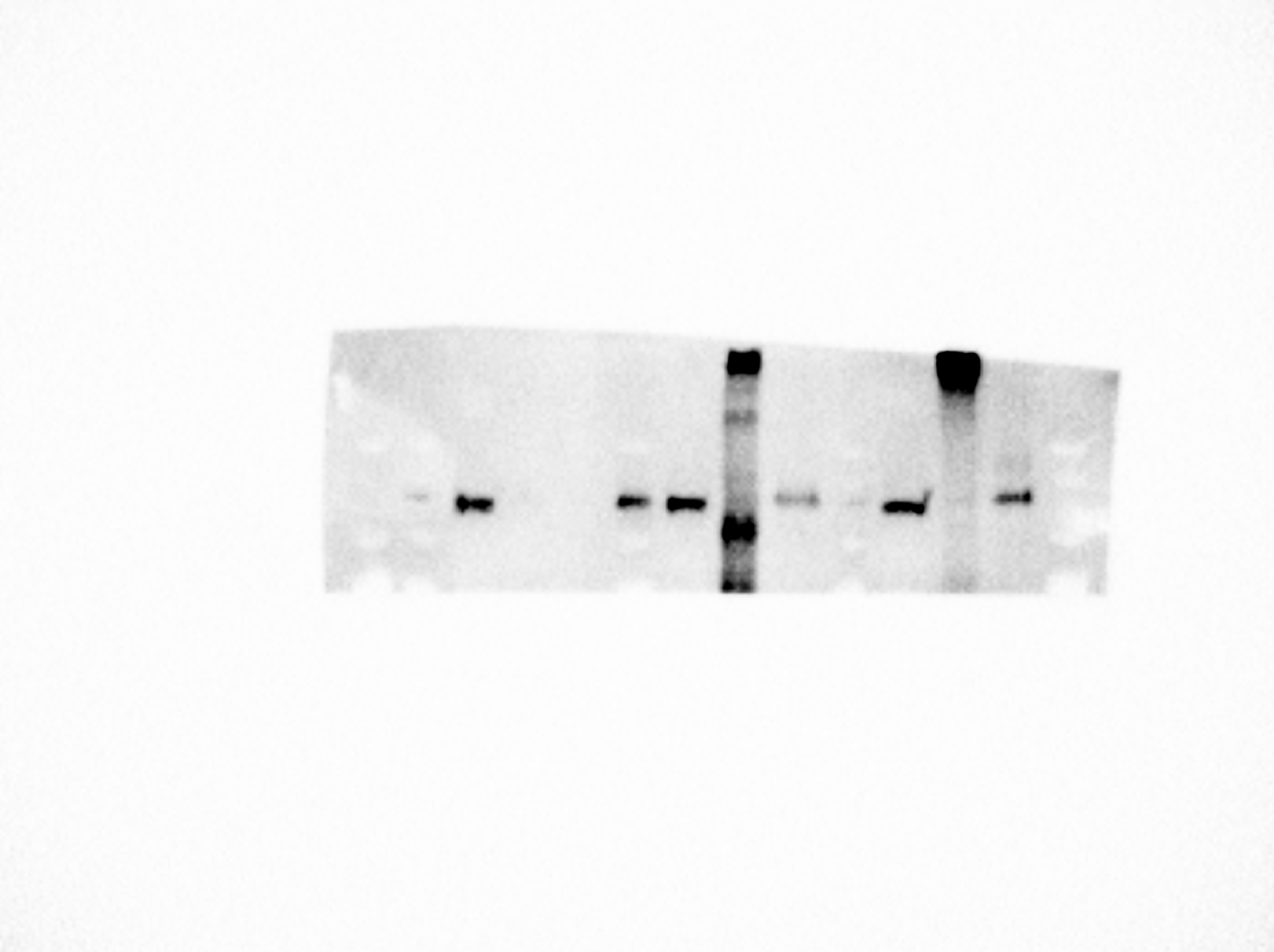

Supplement: Supplementary file 6 — Source data Fig. 4 [file 44321_2026_452_MOESM6_ESM.zip › Figure 4/4D/WB_ Uncropped blots_ NCL.tif]

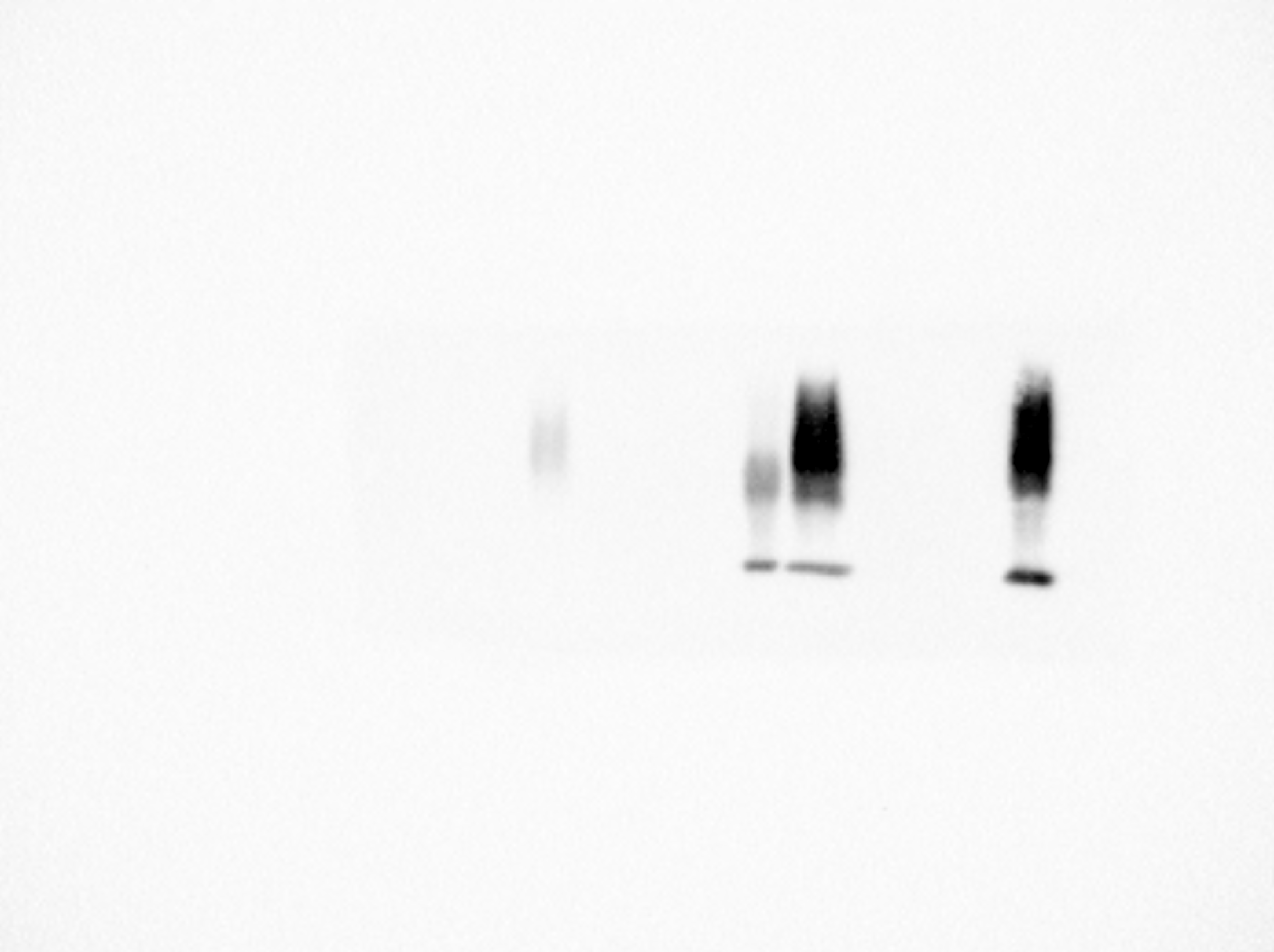

Supplement: Supplementary file 6 — Source data Fig. 4 [file 44321_2026_452_MOESM6_ESM.zip › Figure 4/4D/WB_ Uncropped blots_ sTREM2.tif]

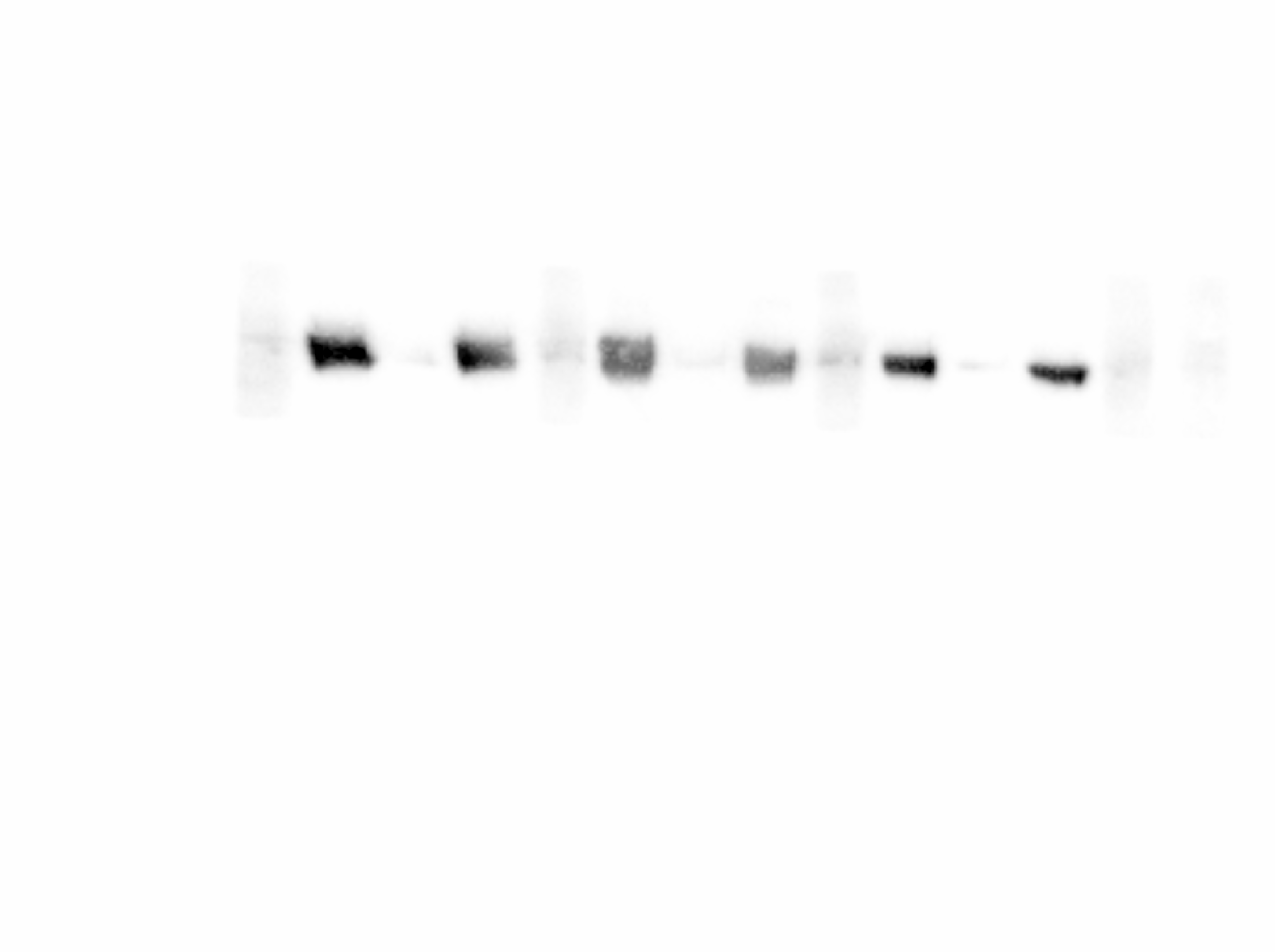

Supplement: Supplementary file 6 — Source data Fig. 4 [file 44321_2026_452_MOESM6_ESM.zip › Figure 4/4E/WB_ Uncropped blots_ NCL.tif]

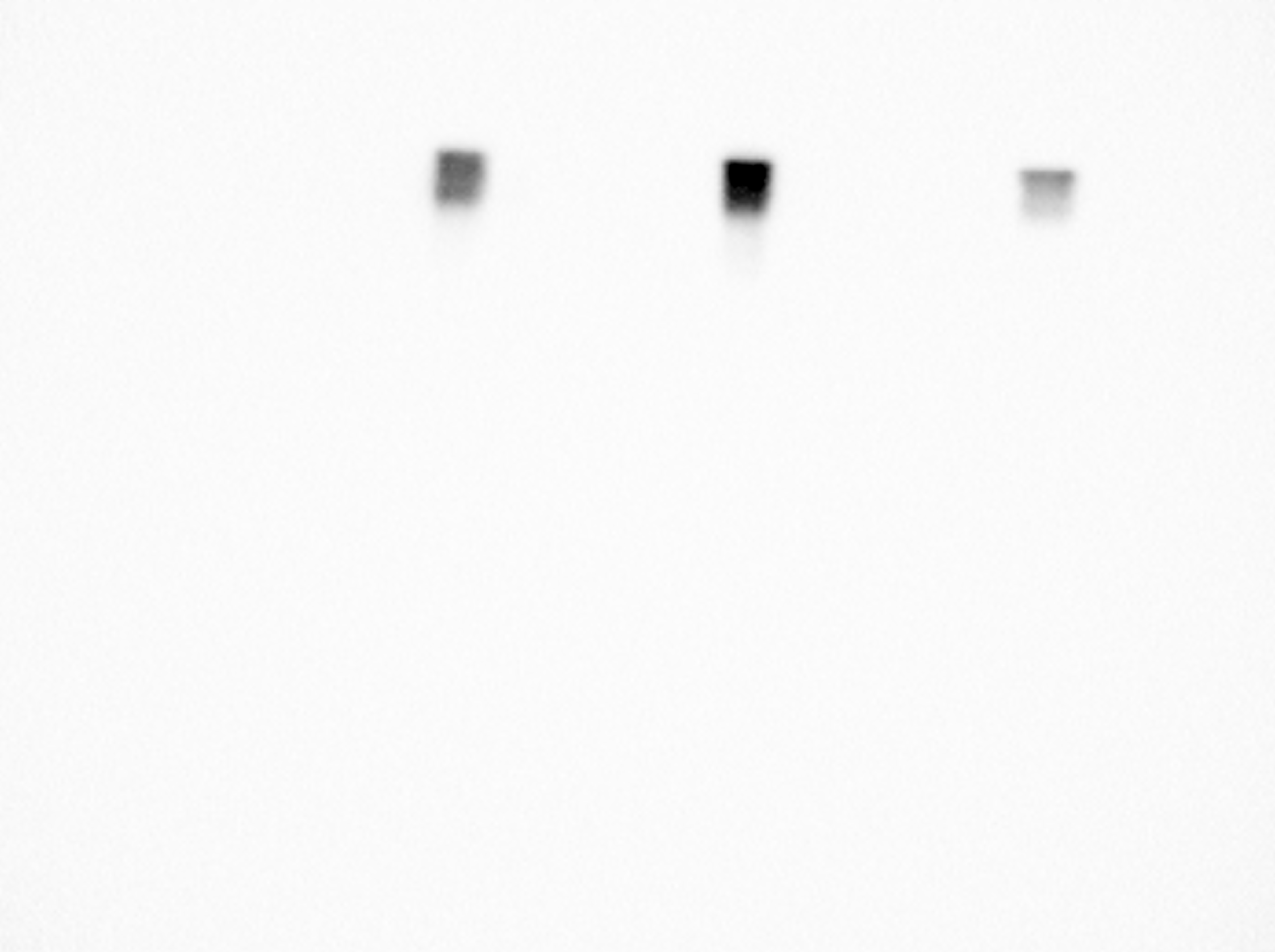

Supplement: Supplementary file 6 — Source data Fig. 4 [file 44321_2026_452_MOESM6_ESM.zip › Figure 4/4E/WB_ Uncropped blots_ sTREM2.tif]

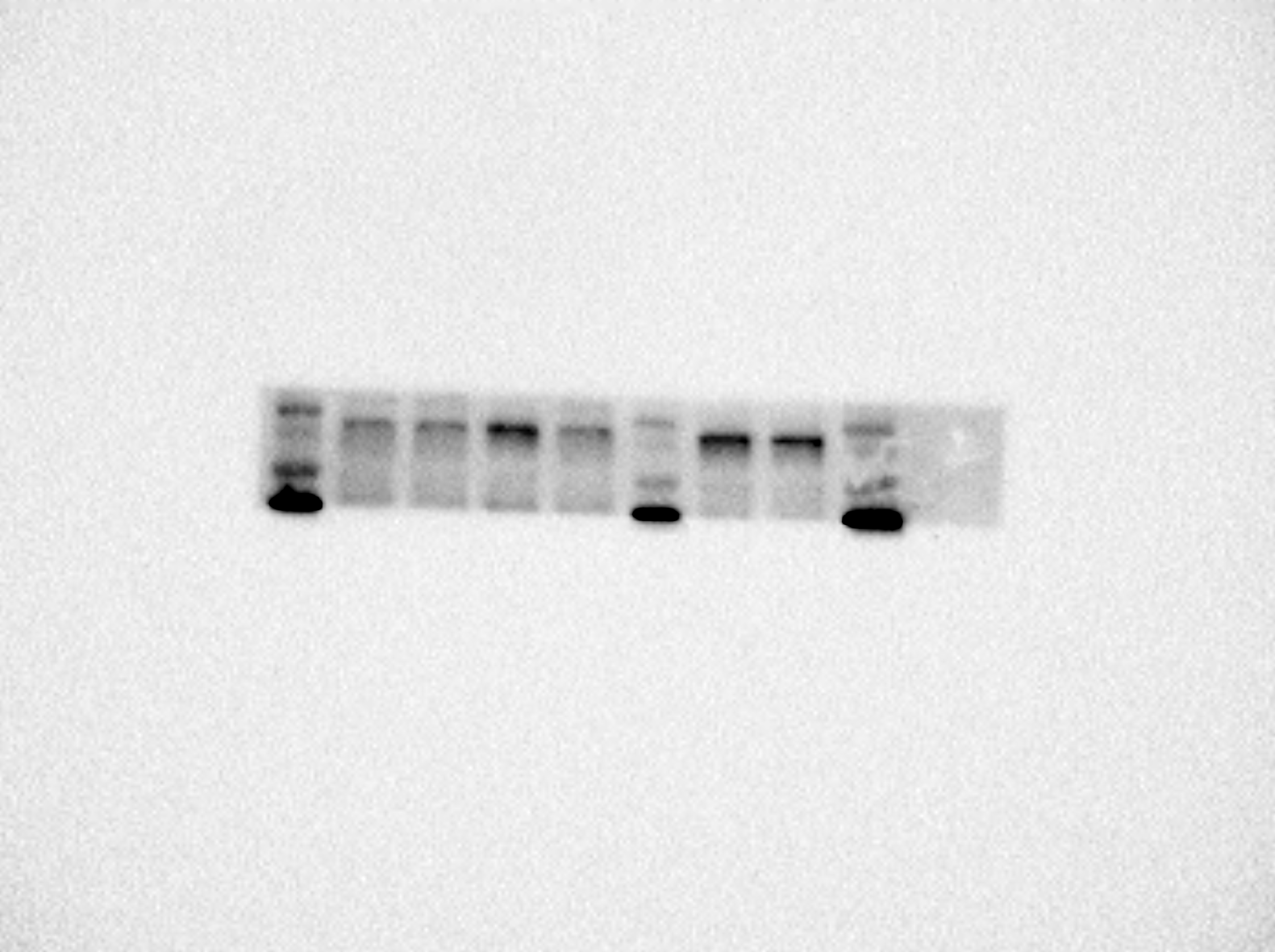

Supplement: Supplementary file 6 — Source data Fig. 4 [file 44321_2026_452_MOESM6_ESM.zip › Figure 4/4G-H/WB_ Uncropped blots_pVEcad.tif]

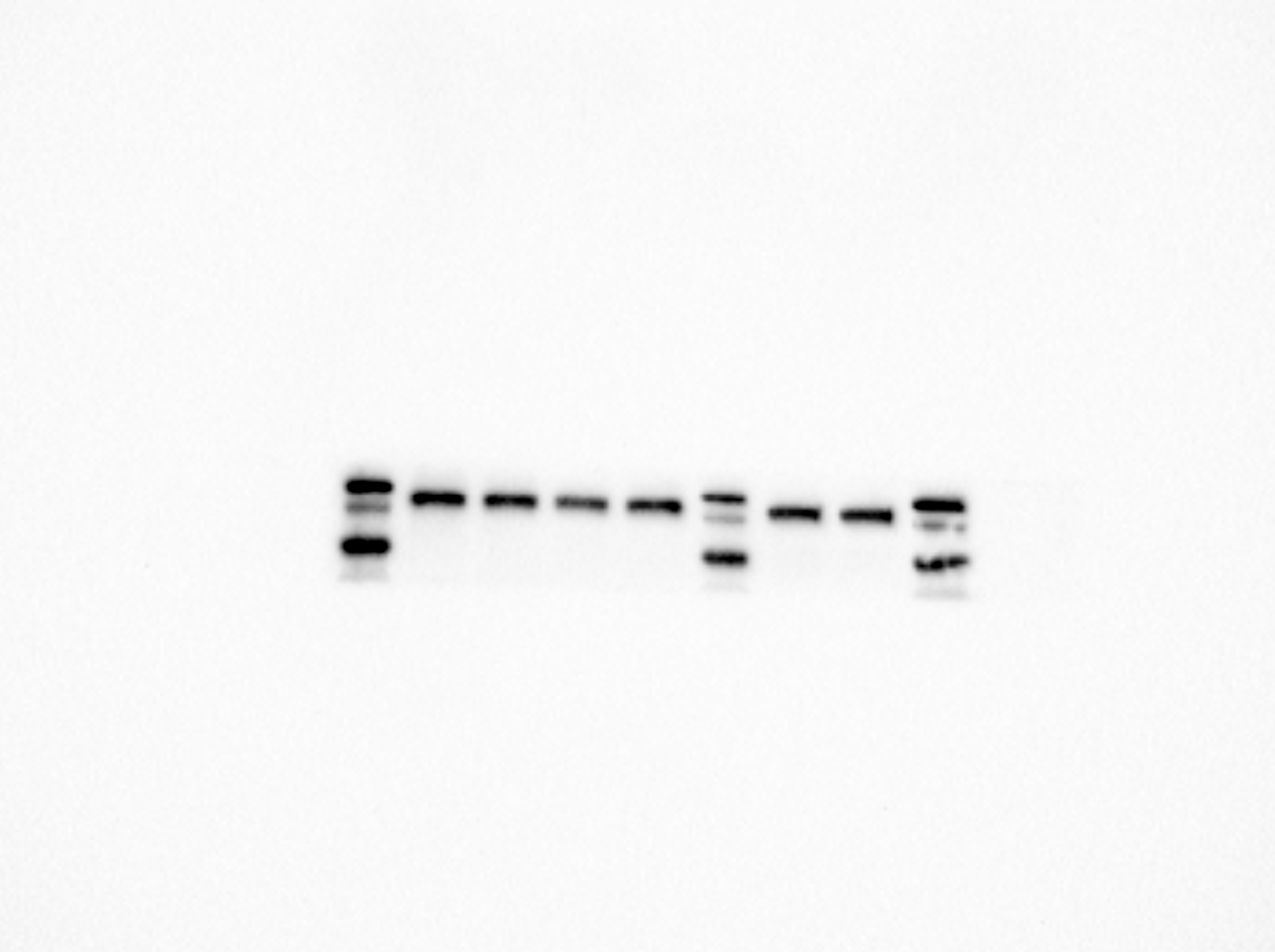

Supplement: Supplementary file 6 — Source data Fig. 4 [file 44321_2026_452_MOESM6_ESM.zip › Figure 4/4G-H/WB_ Uncropped blots_VEcad.tif]

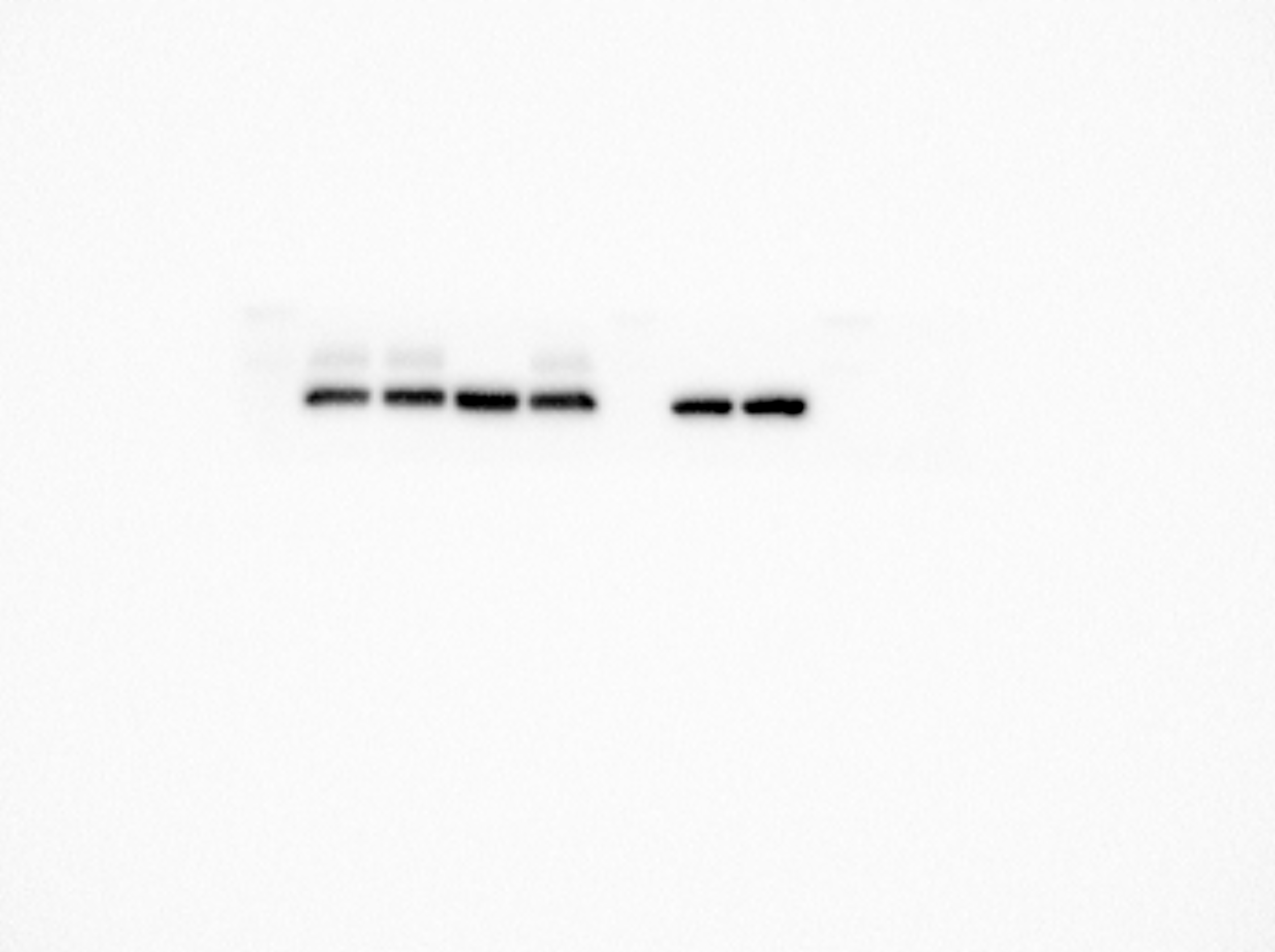

Supplement: Supplementary file 6 — Source data Fig. 4 [file 44321_2026_452_MOESM6_ESM.zip › Figure 4/4G-H/WB_ Uncropped blots_β-actin.tif]

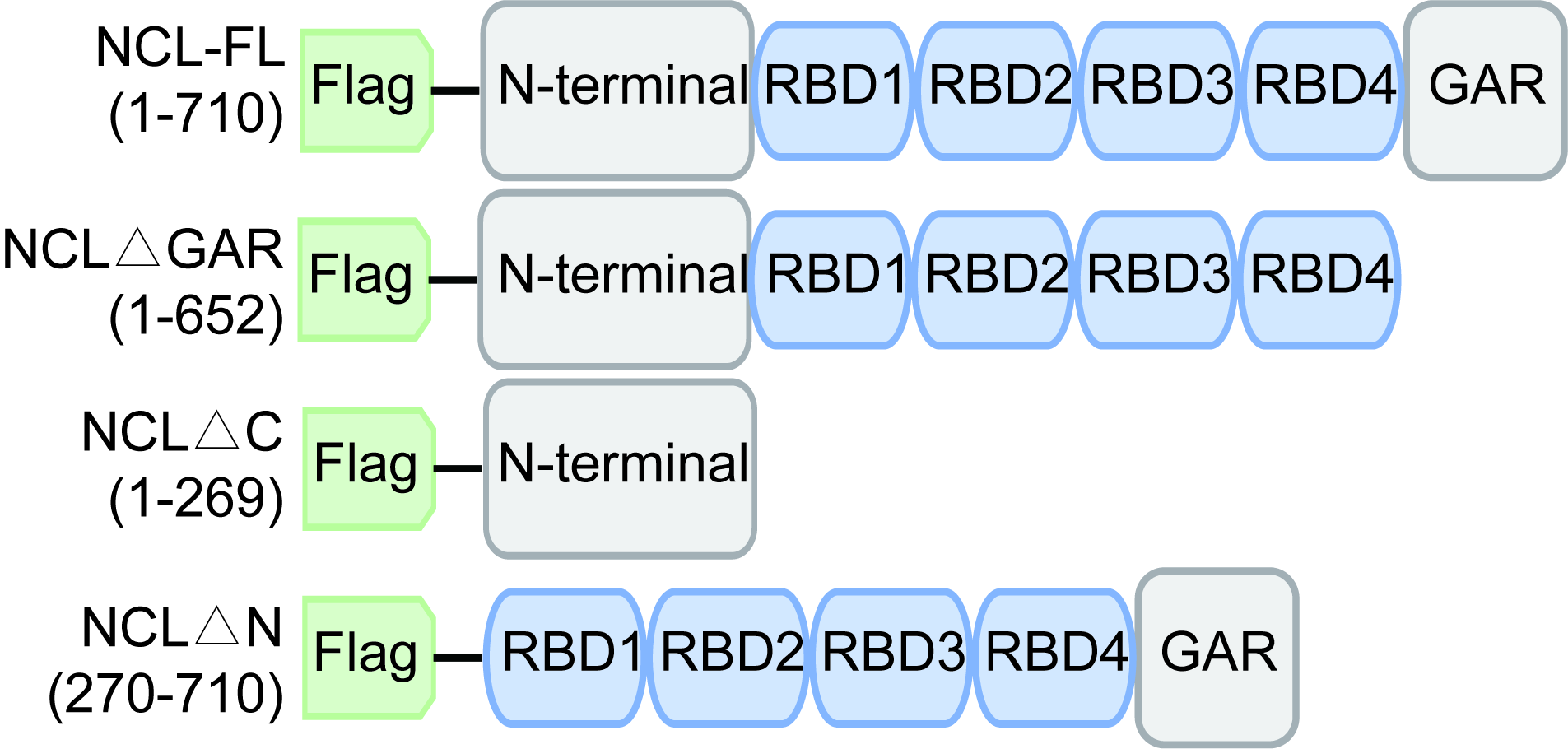

Supplement: Supplementary file 6 — Source data Fig. 4 [file 44321_2026_452_MOESM6_ESM.zip › Figure 4/4J/Figure 4J.tif]

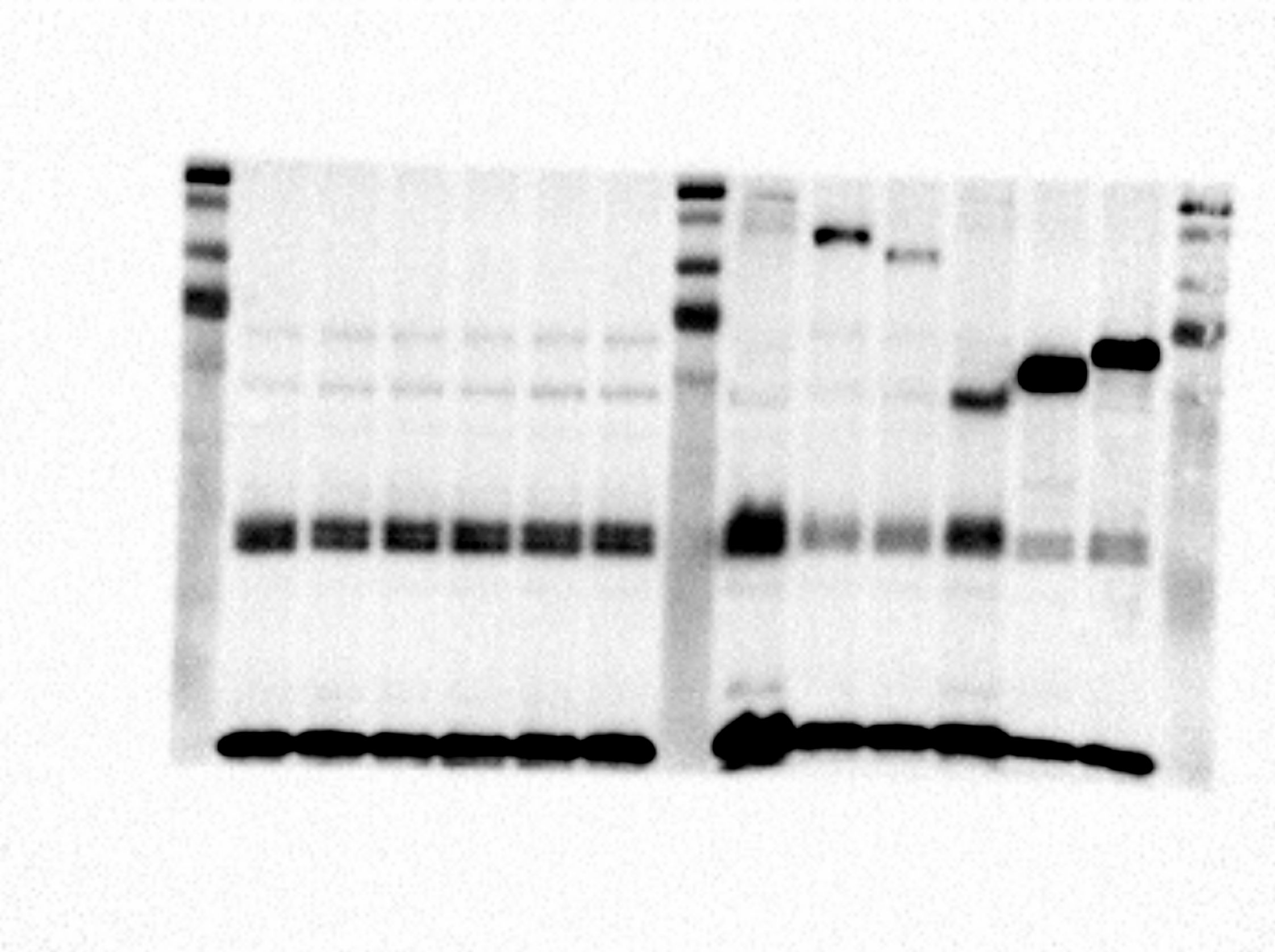

Supplement: Supplementary file 6 — Source data Fig. 4 [file 44321_2026_452_MOESM6_ESM.zip › Figure 4/4K/WB_ Uncropped blots_ IP_Flag.tif]

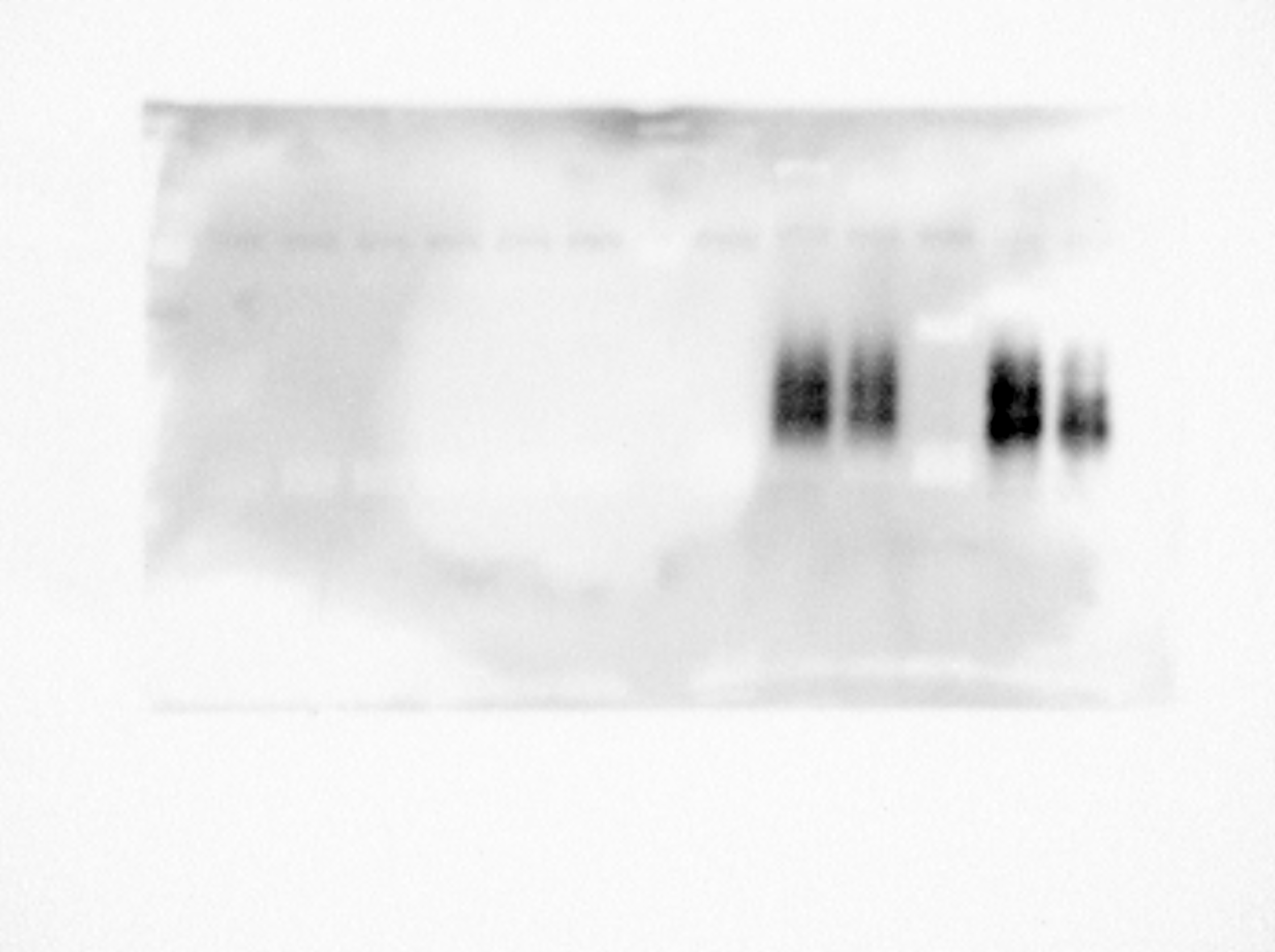

Supplement: Supplementary file 6 — Source data Fig. 4 [file 44321_2026_452_MOESM6_ESM.zip › Figure 4/4K/WB_ Uncropped blots_ IP_sTREM2.tif]

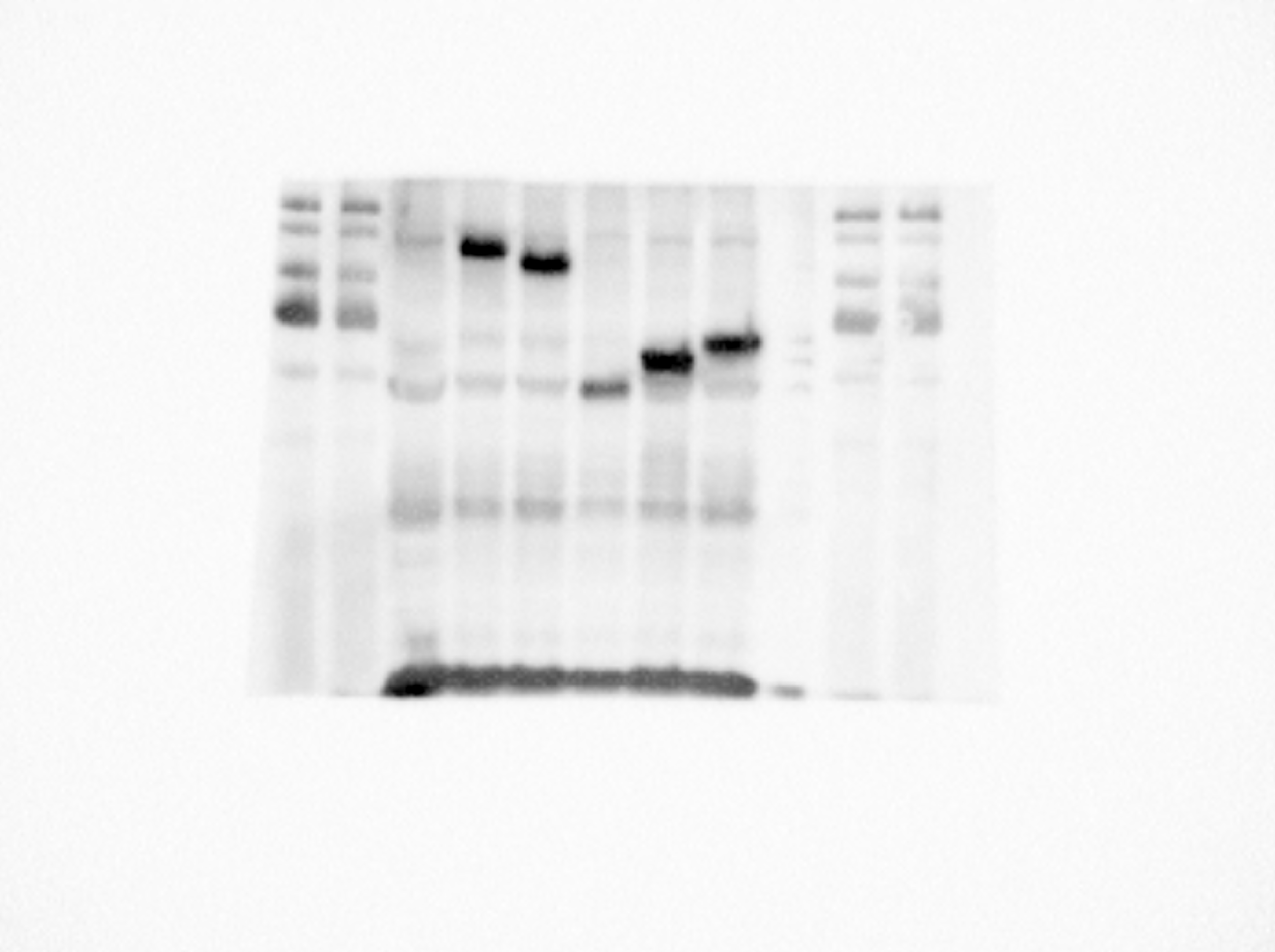

Supplement: Supplementary file 6 — Source data Fig. 4 [file 44321_2026_452_MOESM6_ESM.zip › Figure 4/4K/WB_ Uncropped blots_input_Flag.tif]

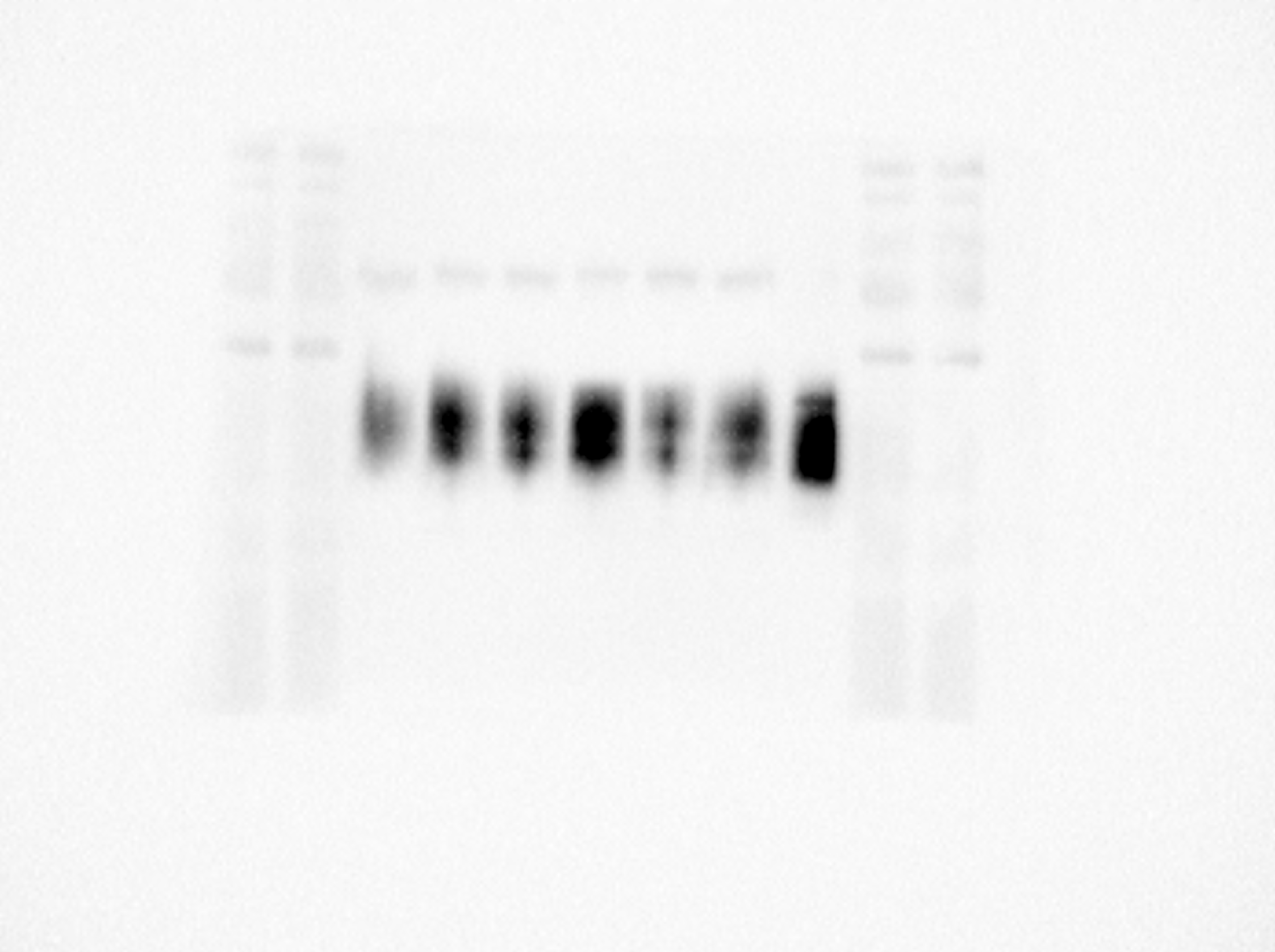

Supplement: Supplementary file 6 — Source data Fig. 4 [file 44321_2026_452_MOESM6_ESM.zip › Figure 4/4K/WB_ Uncropped blots_input_sTREM2.tif]

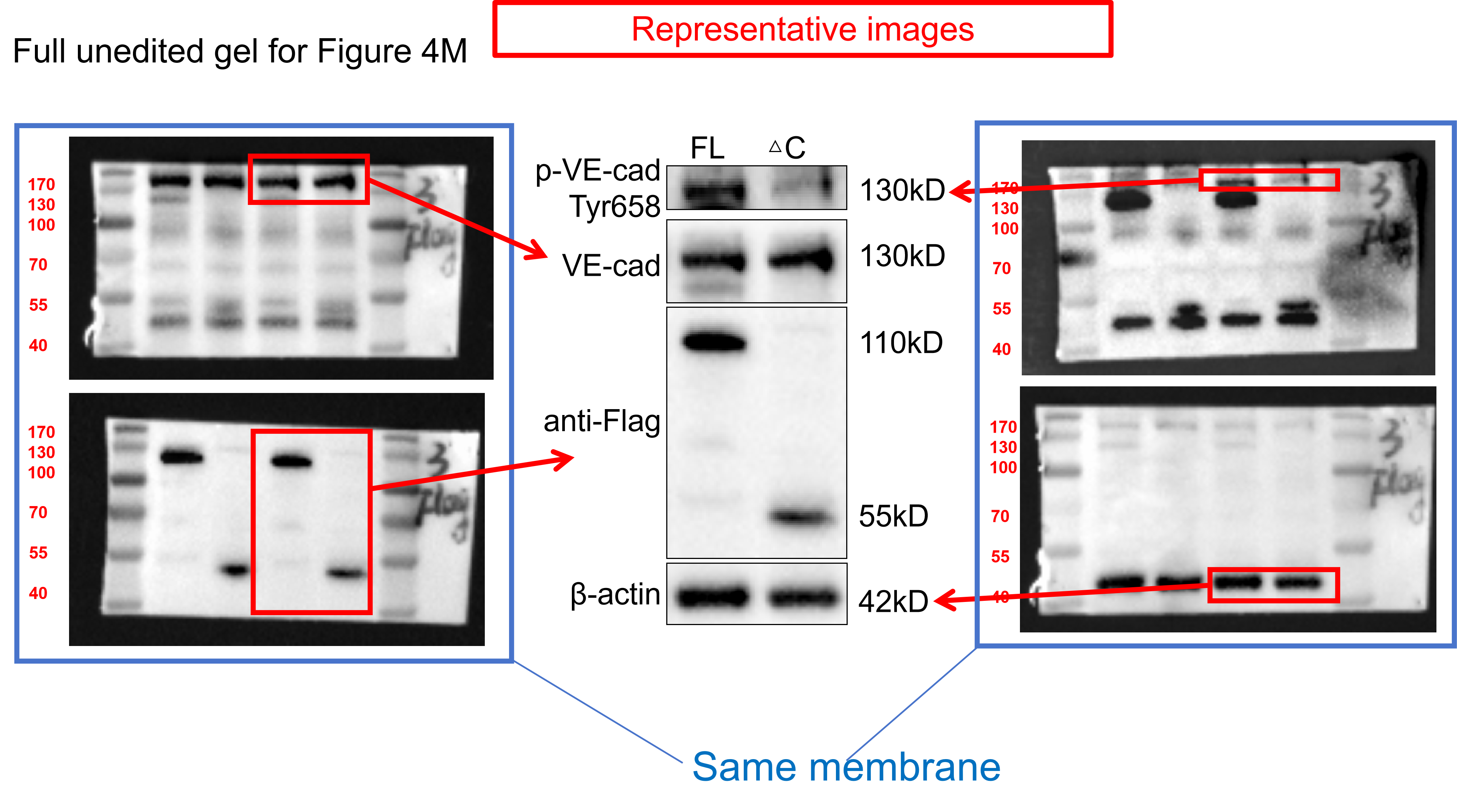

Supplement: Supplementary file 6 — Source data Fig. 4 [file 44321_2026_452_MOESM6_ESM.zip › Figure 4/4M/Instructions for cropping Western blot images 1.tif]

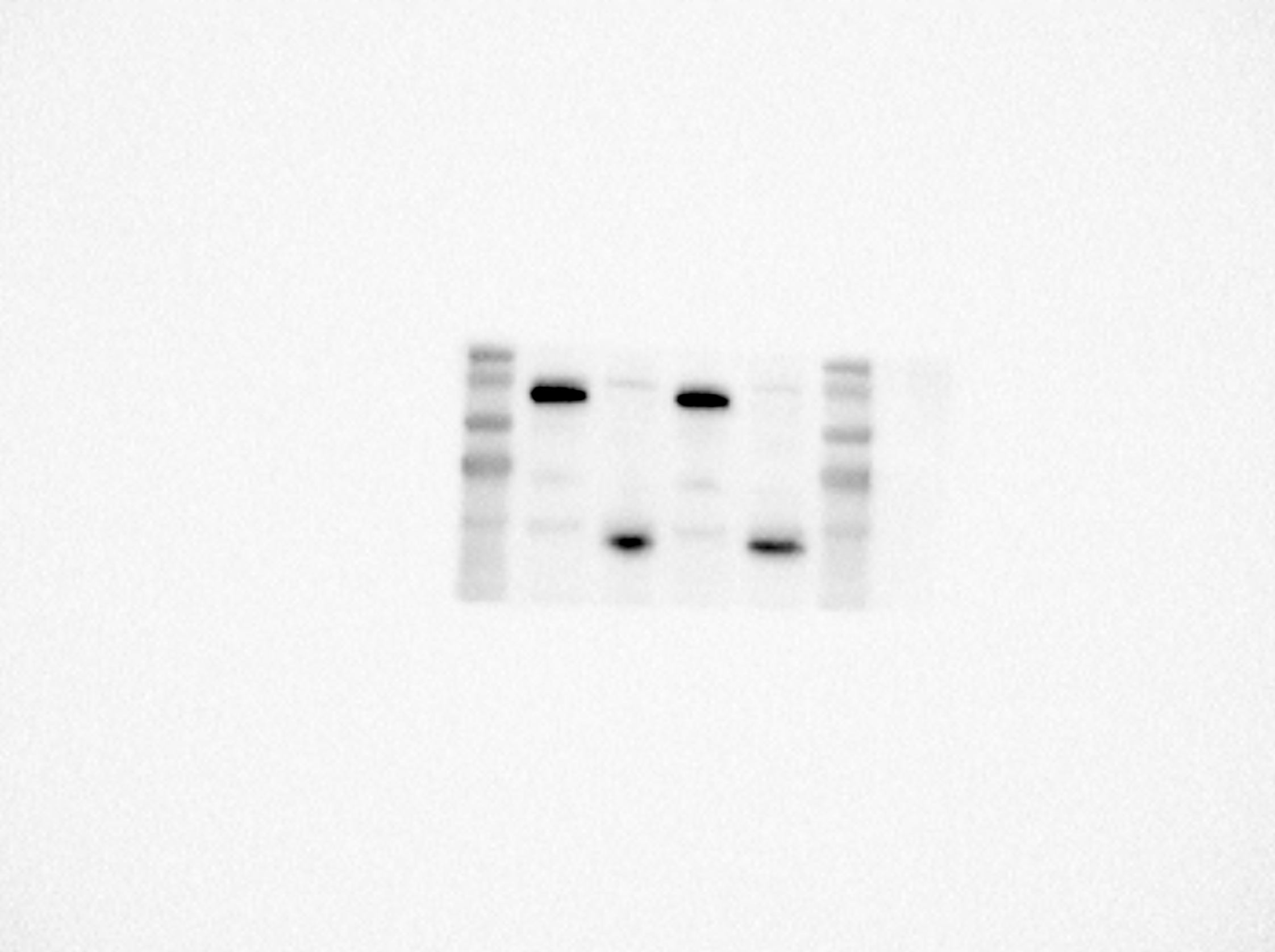

Supplement: Supplementary file 6 — Source data Fig. 4 [file 44321_2026_452_MOESM6_ESM.zip › Figure 4/4M/WB_ Uncropped blots_ Flag.tif]

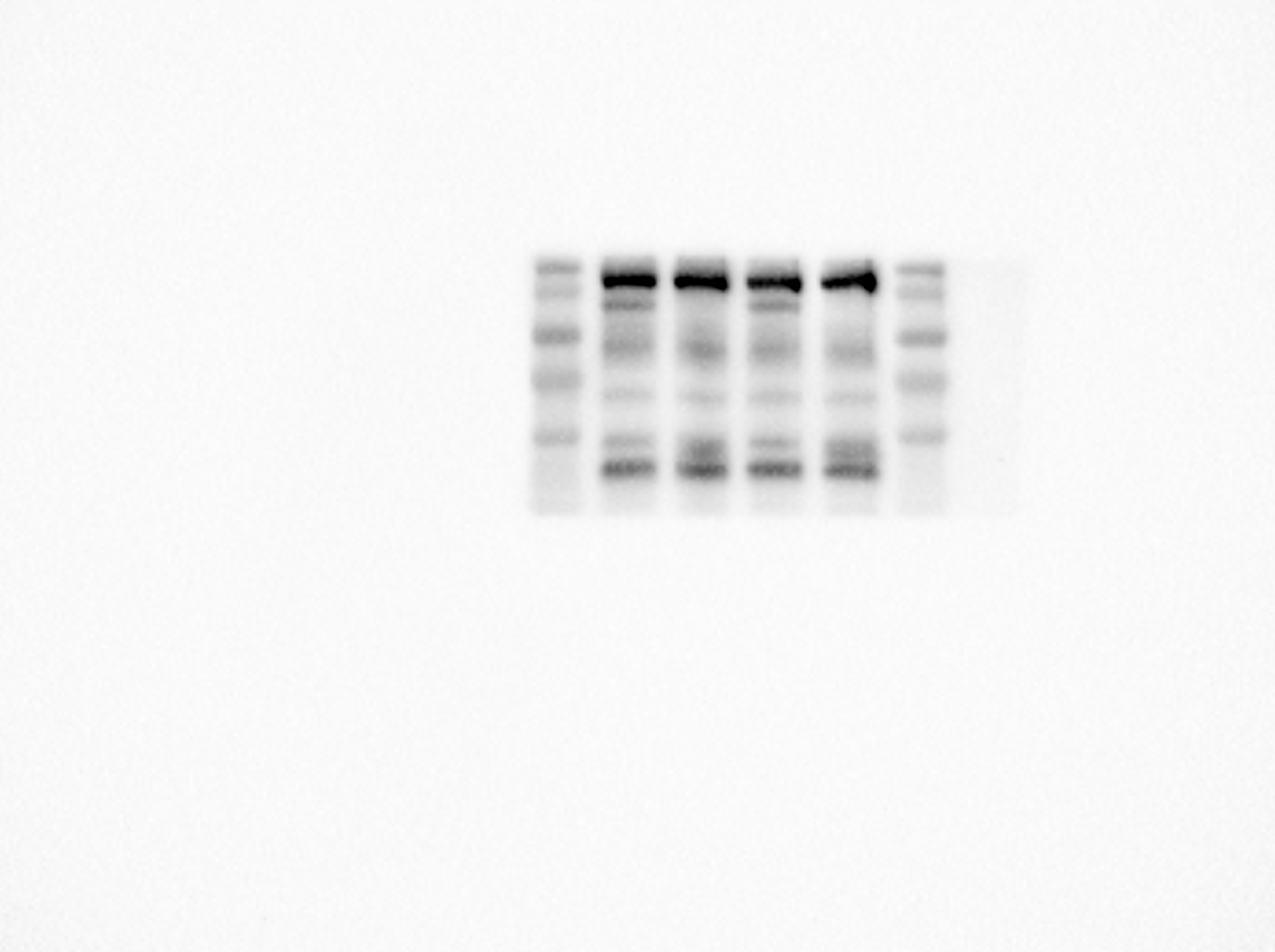

Supplement: Supplementary file 6 — Source data Fig. 4 [file 44321_2026_452_MOESM6_ESM.zip › Figure 4/4M/WB_ Uncropped blots_ VEcad.tif]

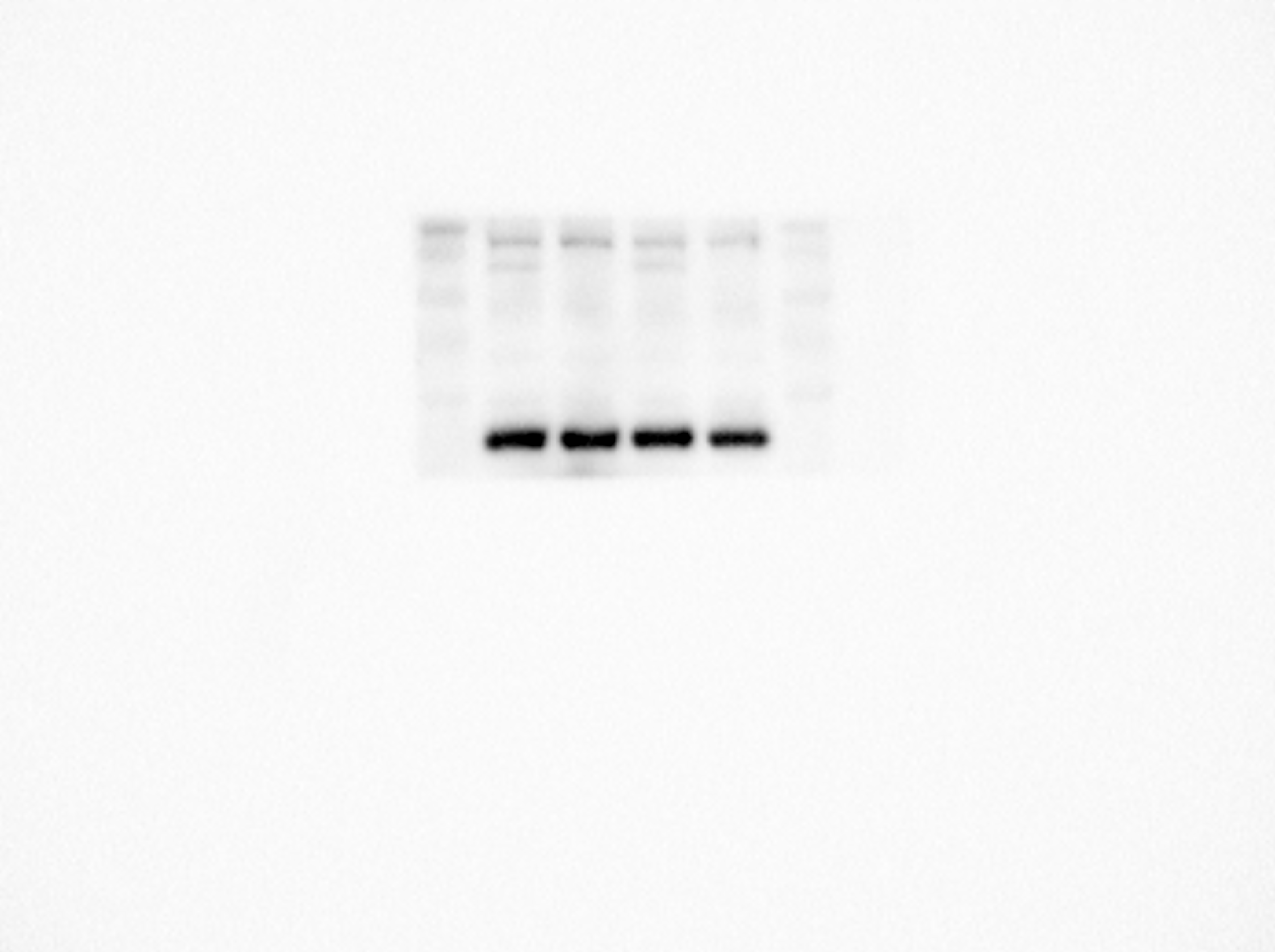

Supplement: Supplementary file 6 — Source data Fig. 4 [file 44321_2026_452_MOESM6_ESM.zip › Figure 4/4M/WB_ Uncropped blots_ β-actin.tif]

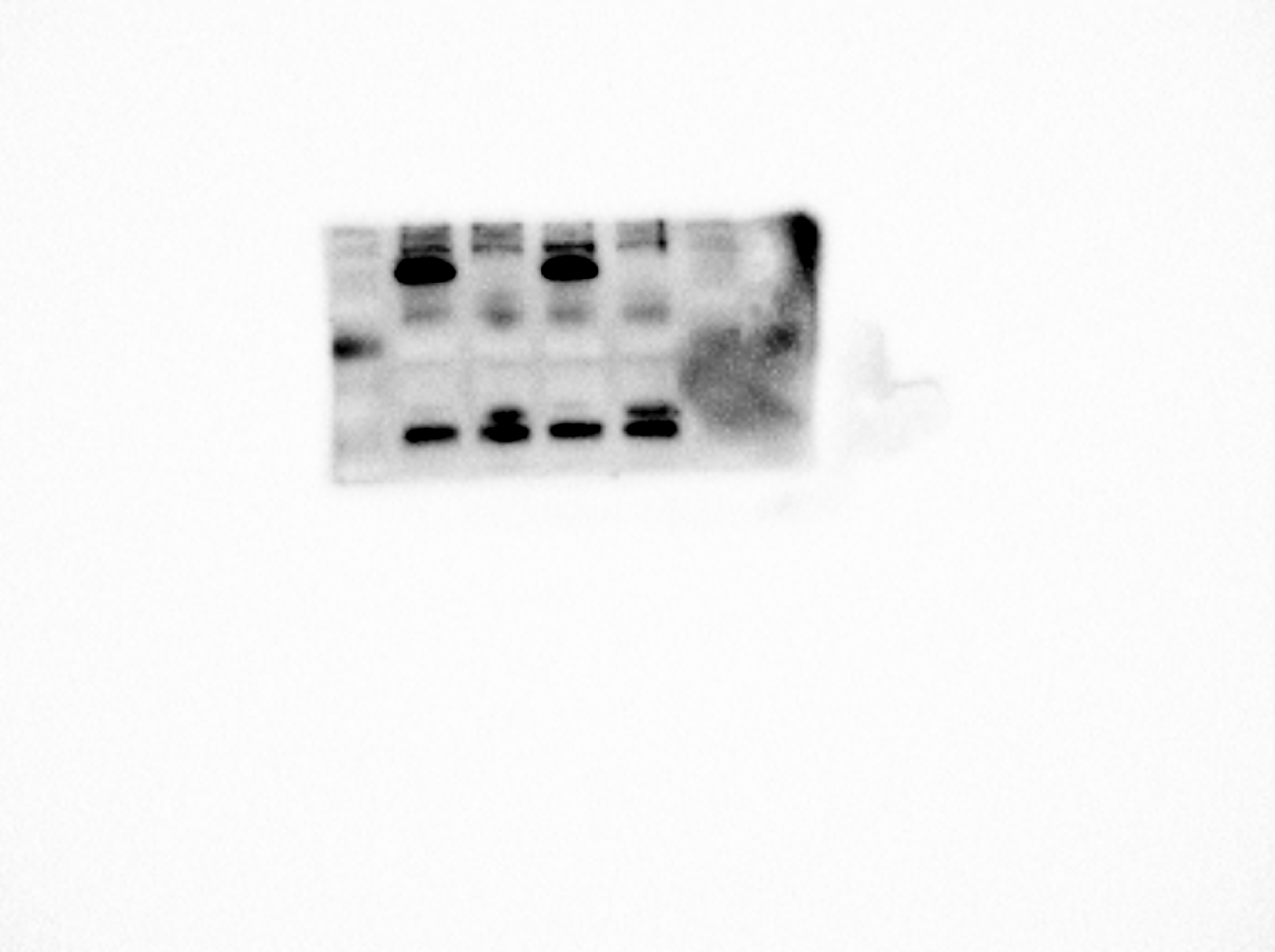

Supplement: Supplementary file 6 — Source data Fig. 4 [file 44321_2026_452_MOESM6_ESM.zip › Figure 4/4M/WB_ Uncropped blots_pVEcad.tif]

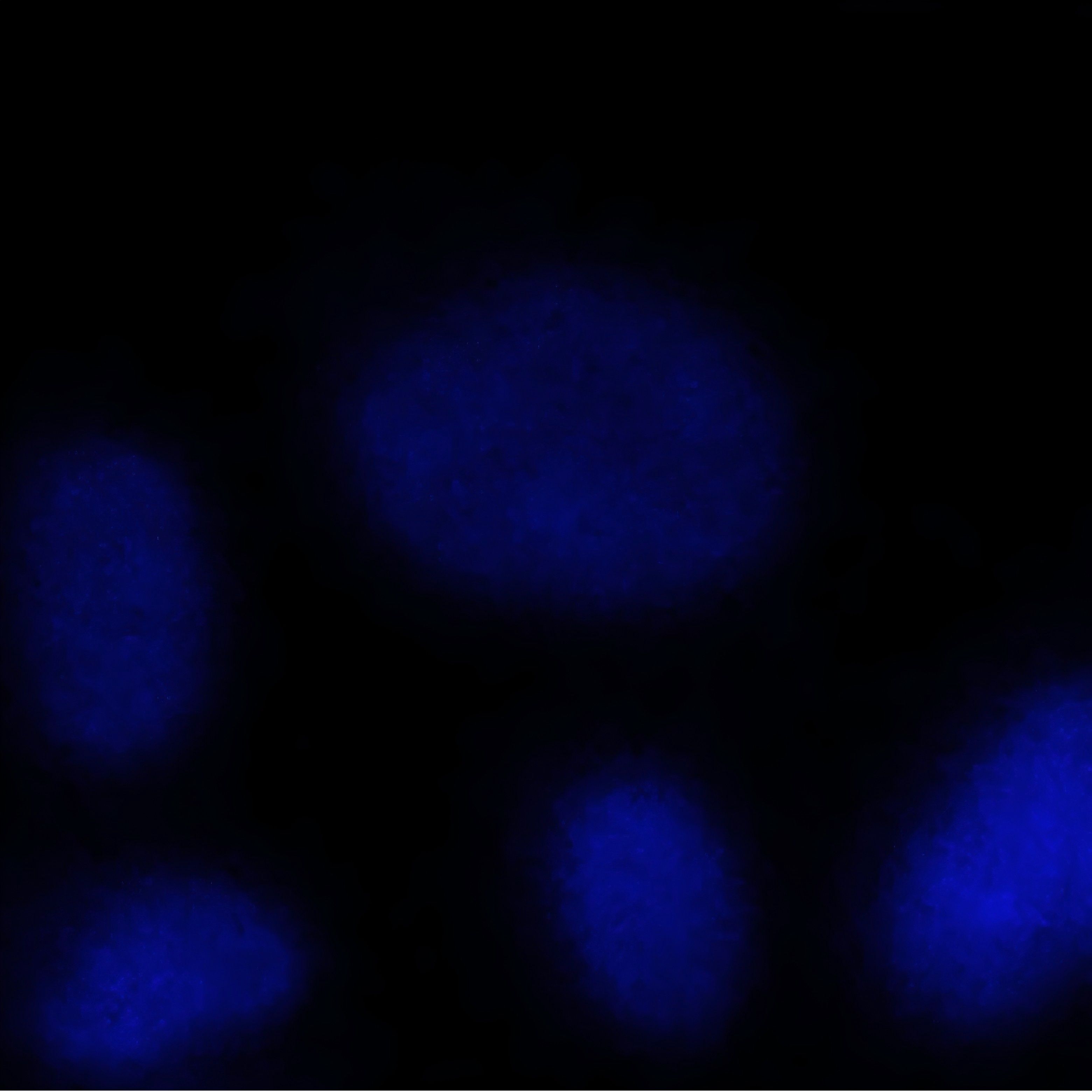

Supplement: Supplementary file 6 — Source data Fig. 4 [file 44321_2026_452_MOESM6_ESM.zip › Figure 4/4N-O/FL+sTREM2 DAPI.tif]

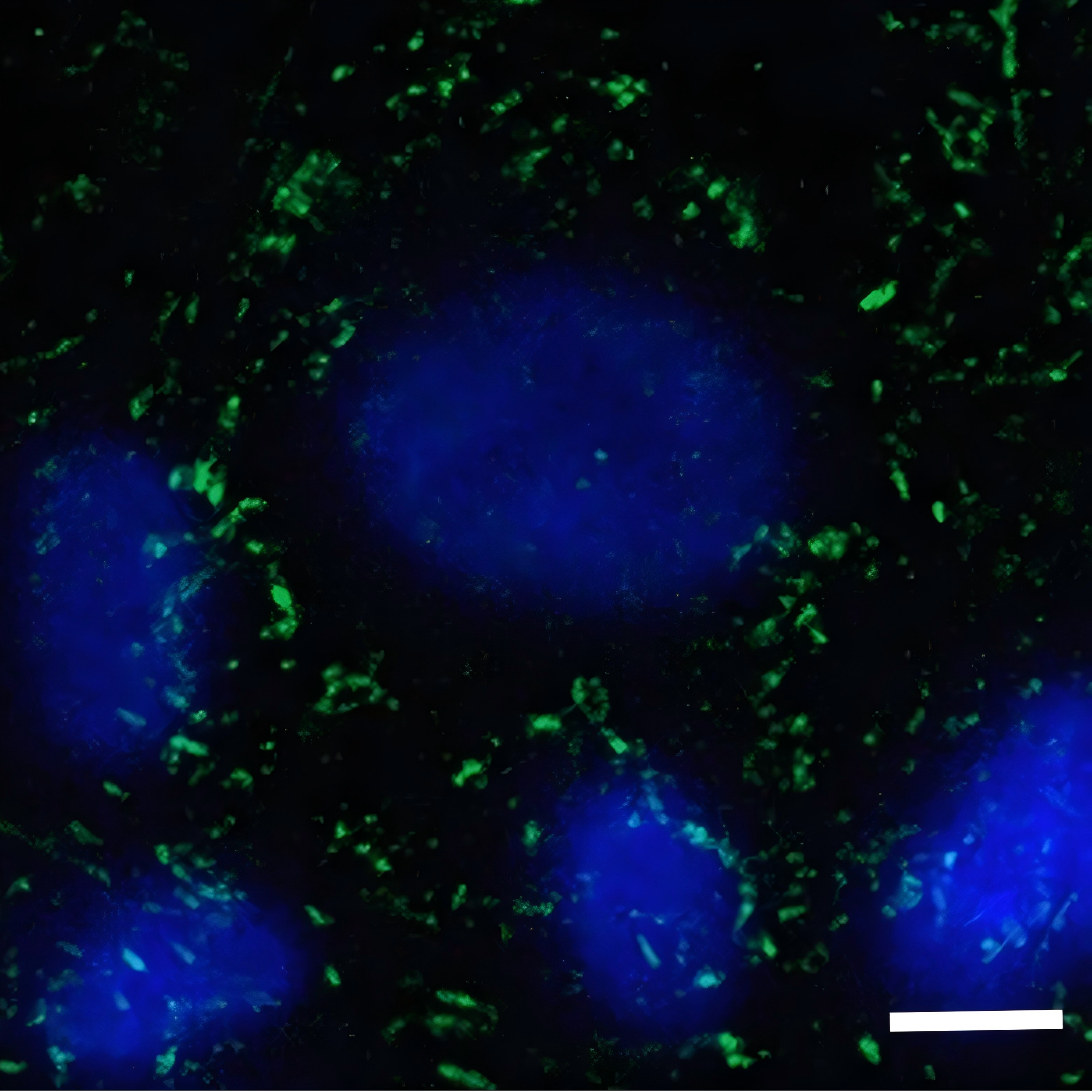

Supplement: Supplementary file 6 — Source data Fig. 4 [file 44321_2026_452_MOESM6_ESM.zip › Figure 4/4N-O/FL+sTREM2 merge.tif]

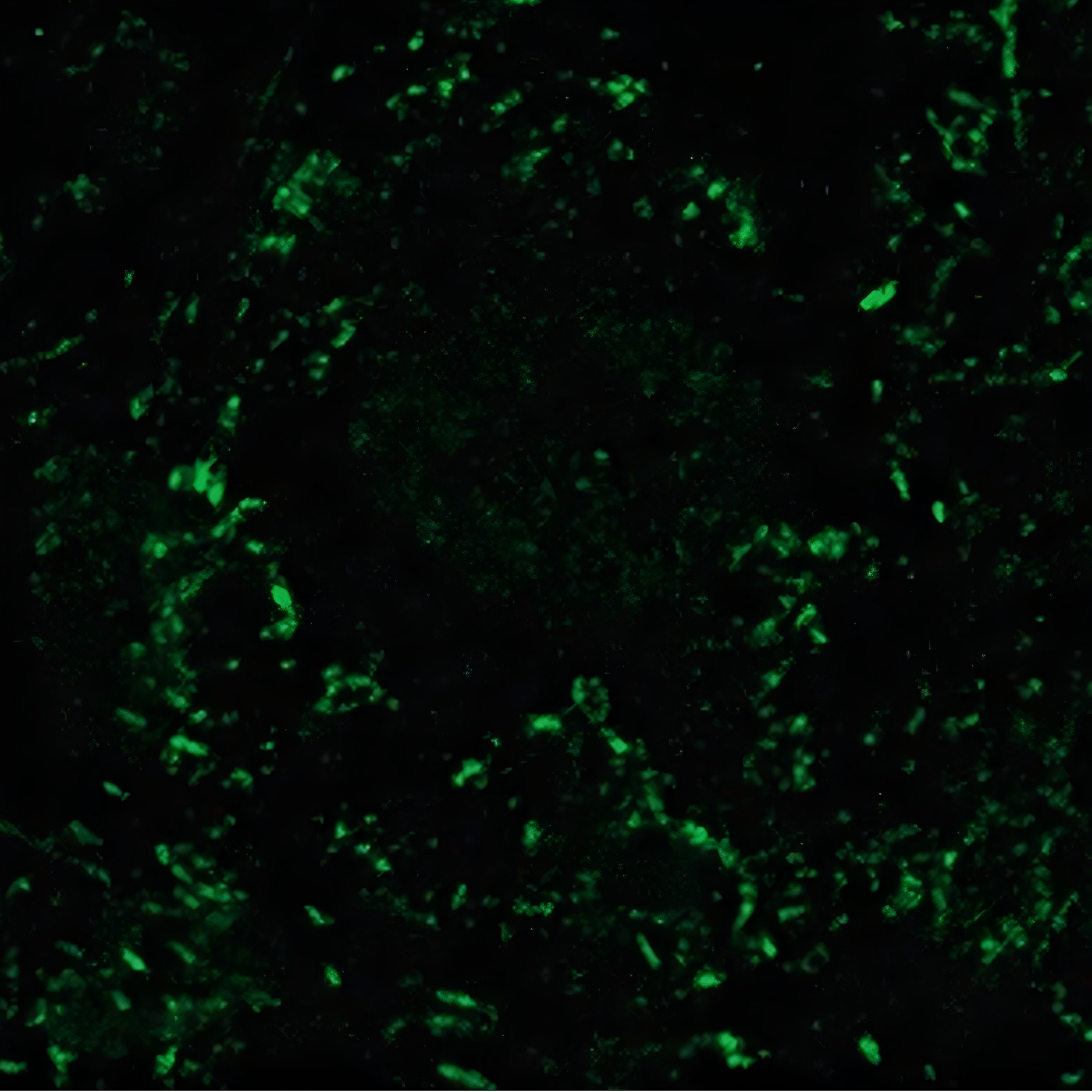

Supplement: Supplementary file 6 — Source data Fig. 4 [file 44321_2026_452_MOESM6_ESM.zip › Figure 4/4N-O/FL+sTREM2 p-VEcad.tif]

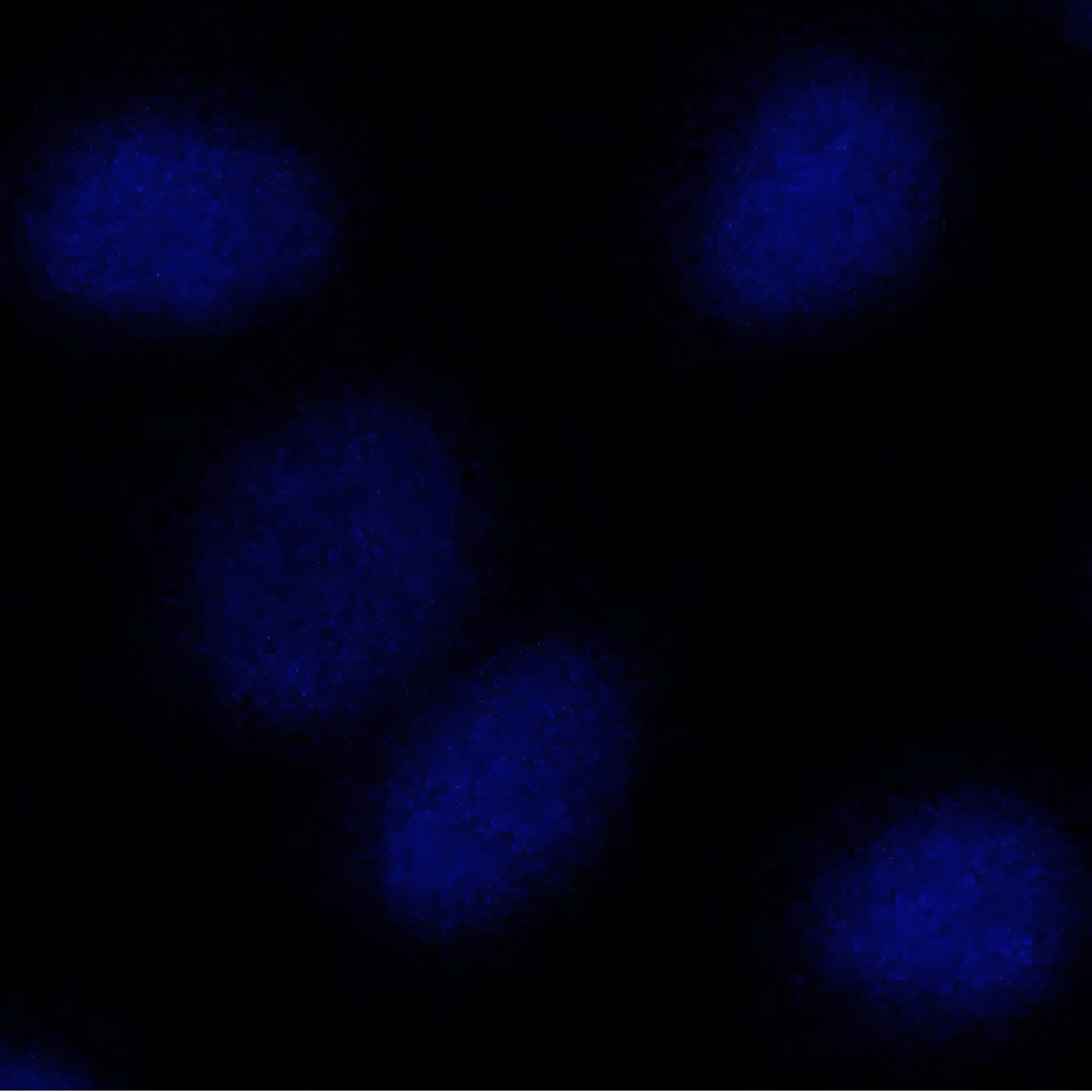

Supplement: Supplementary file 6 — Source data Fig. 4 [file 44321_2026_452_MOESM6_ESM.zip › Figure 4/4N-O/△C+sTREM2 DAPI.tif]

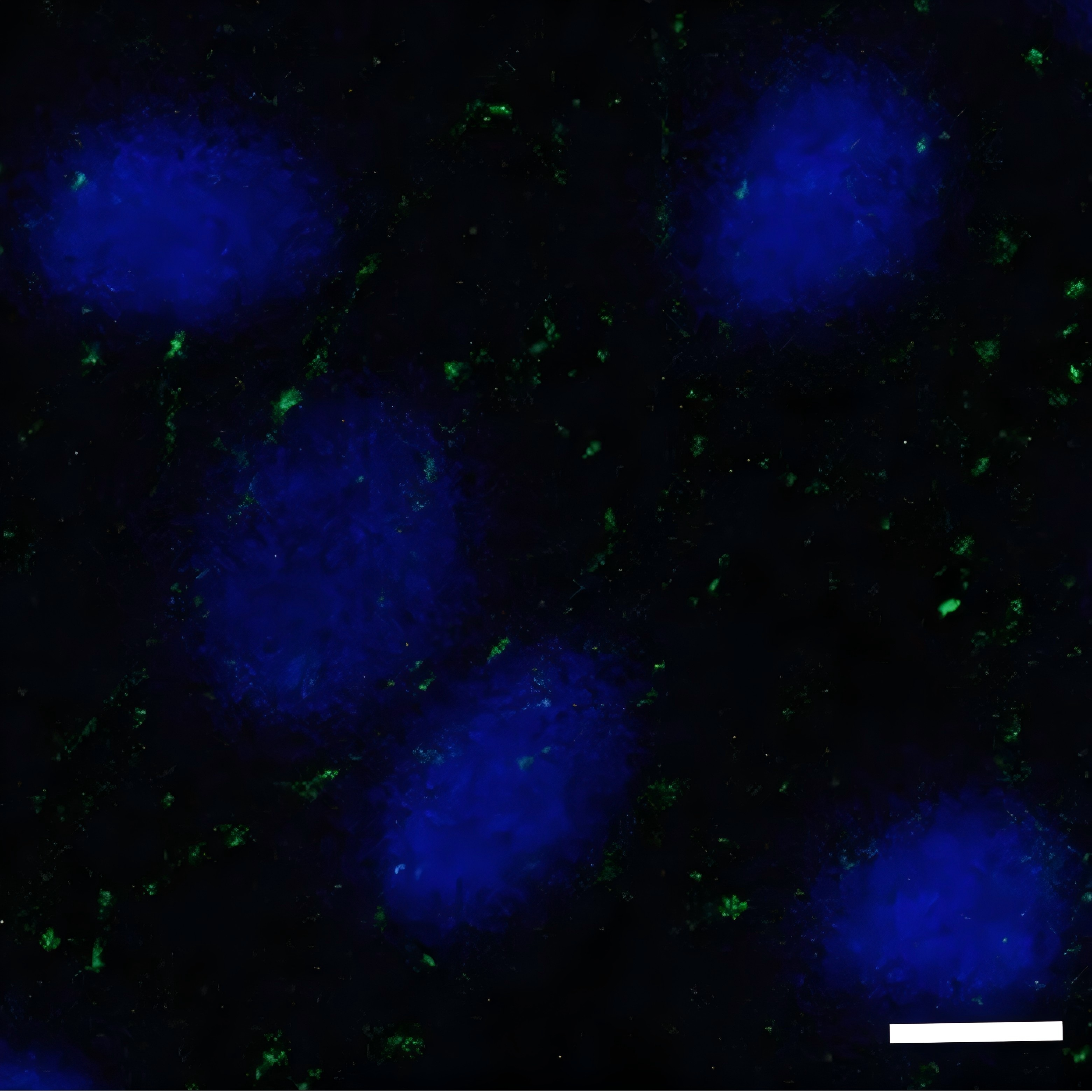

Supplement: Supplementary file 6 — Source data Fig. 4 [file 44321_2026_452_MOESM6_ESM.zip › Figure 4/4N-O/△C+sTREM2 merge.tif]

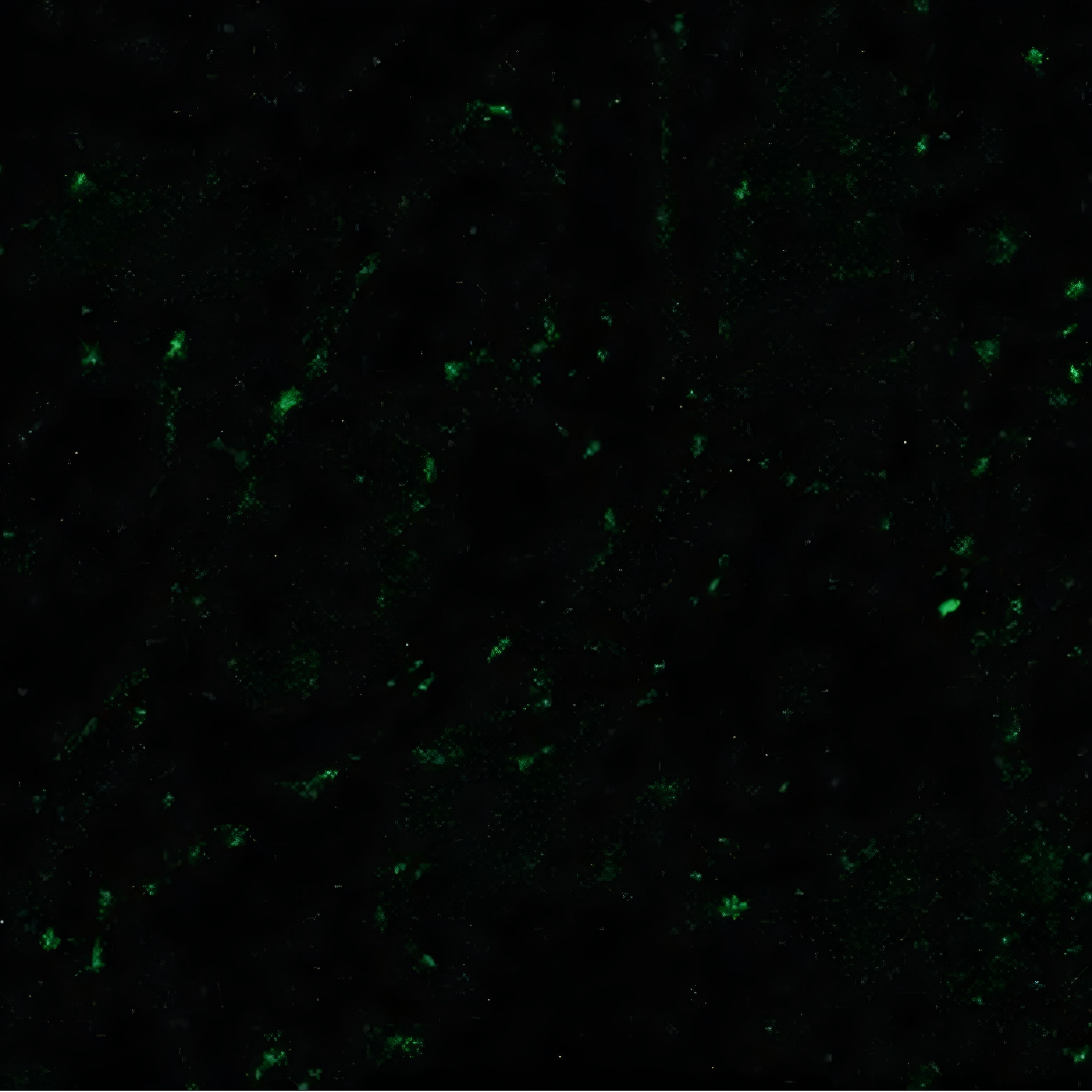

Supplement: Supplementary file 6 — Source data Fig. 4 [file 44321_2026_452_MOESM6_ESM.zip › Figure 4/4N-O/△C+sTREM2 p-VEcad.tif]

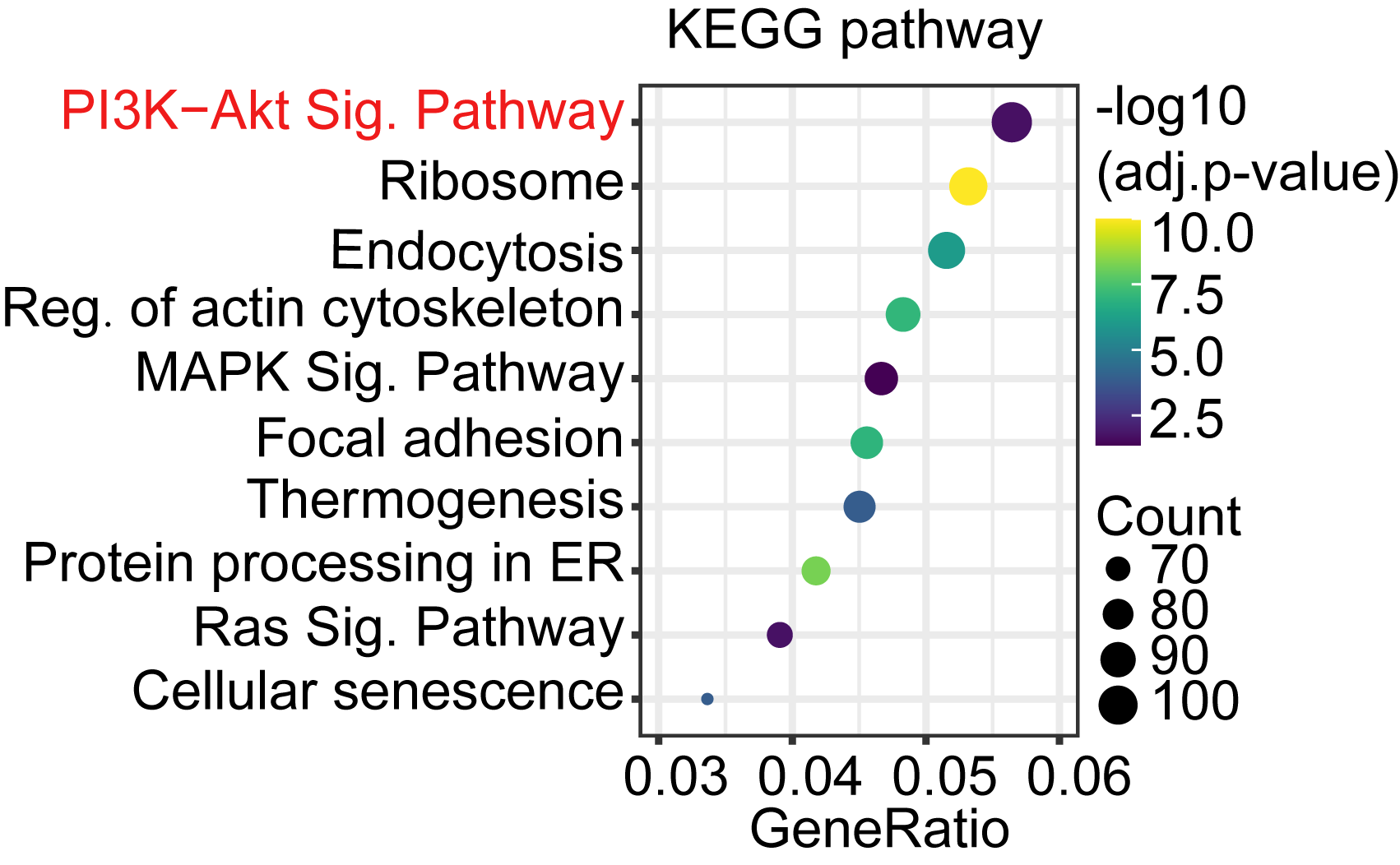

Supplement: Supplementary file 7 — Source data Fig. 5 [file 44321_2026_452_MOESM7_ESM.zip › Figure 5/5A/Figure 5A RNAseq_HUVEC_KEGGtop10.tif]

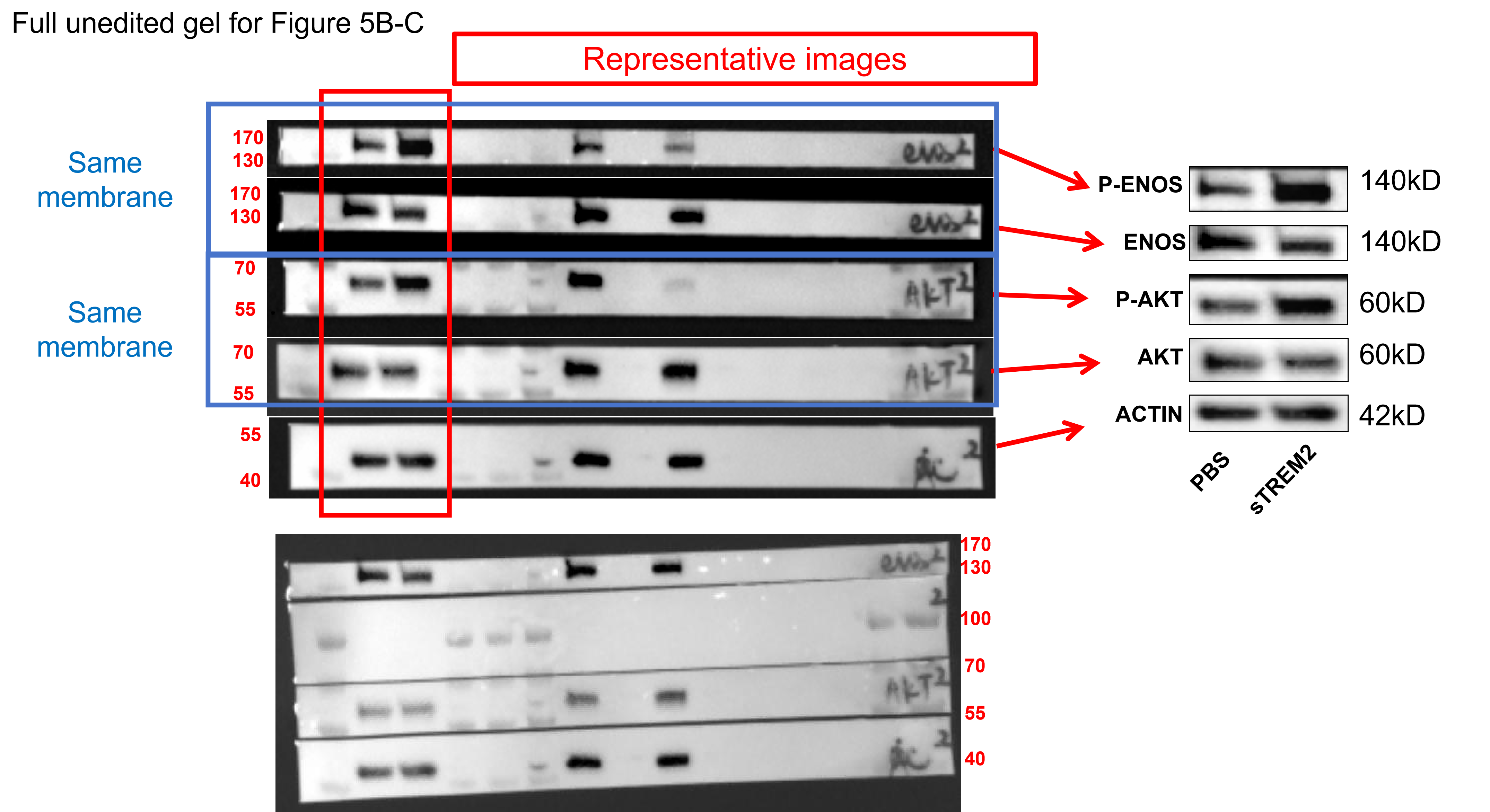

Supplement: Supplementary file 7 — Source data Fig. 5 [file 44321_2026_452_MOESM7_ESM.zip › Figure 5/5B-C/Instructions for cropping Western blot images 1.tif]

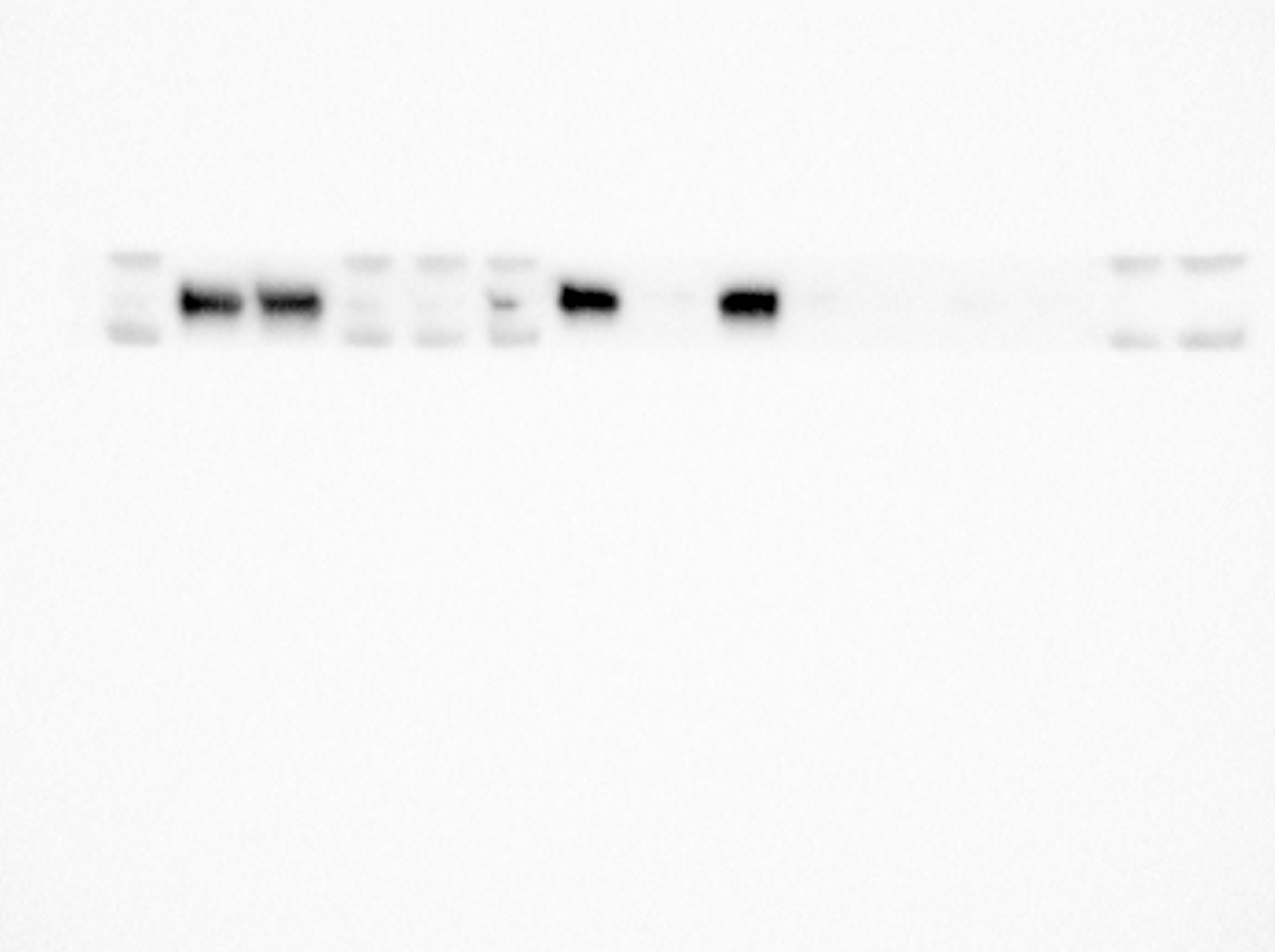

Supplement: Supplementary file 7 — Source data Fig. 5 [file 44321_2026_452_MOESM7_ESM.zip › Figure 5/5B-C/WB_ Uncropped blots_ AKT.tif]

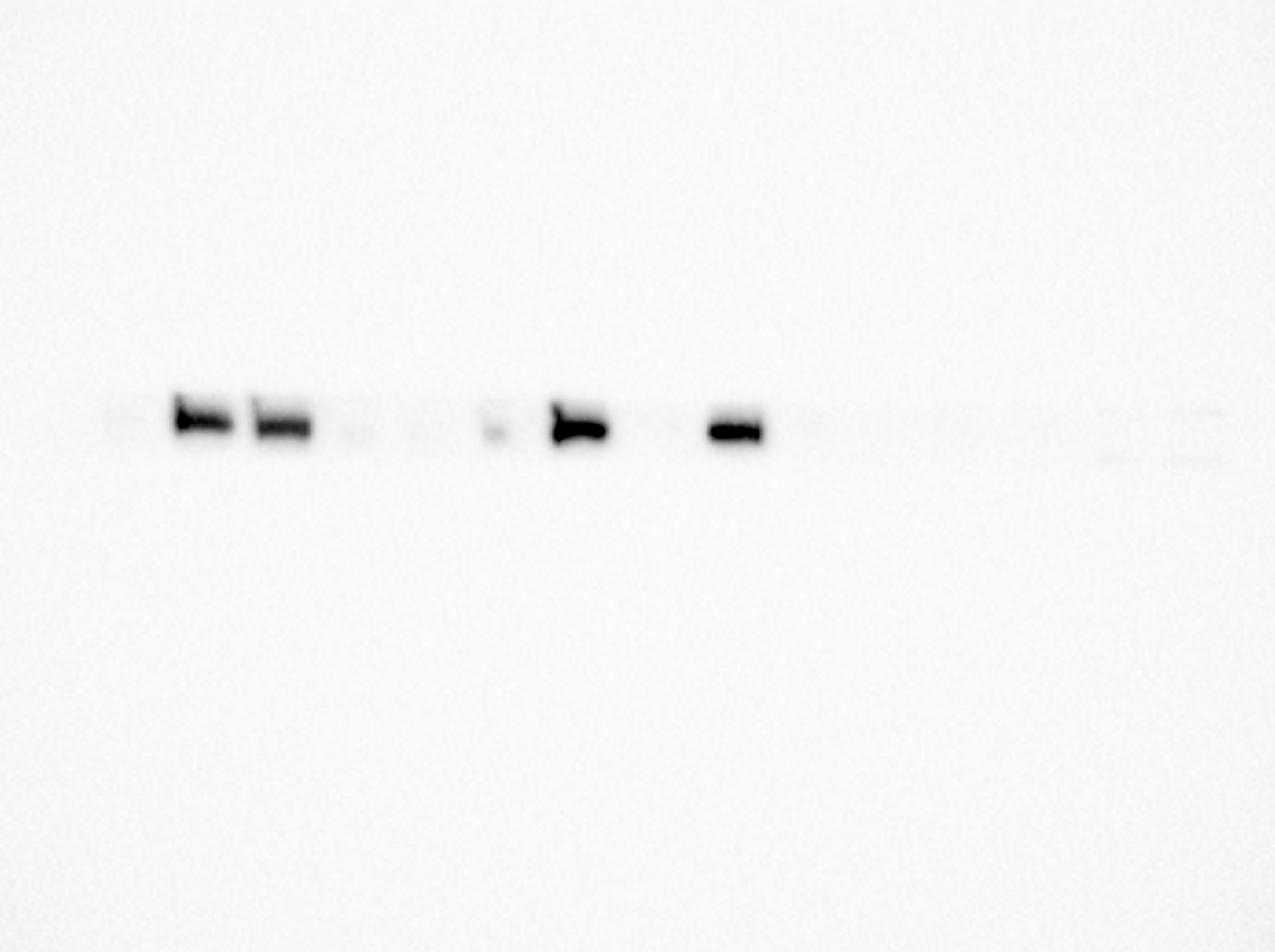

Supplement: Supplementary file 7 — Source data Fig. 5 [file 44321_2026_452_MOESM7_ESM.zip › Figure 5/5B-C/WB_ Uncropped blots_ eNOS.tif]

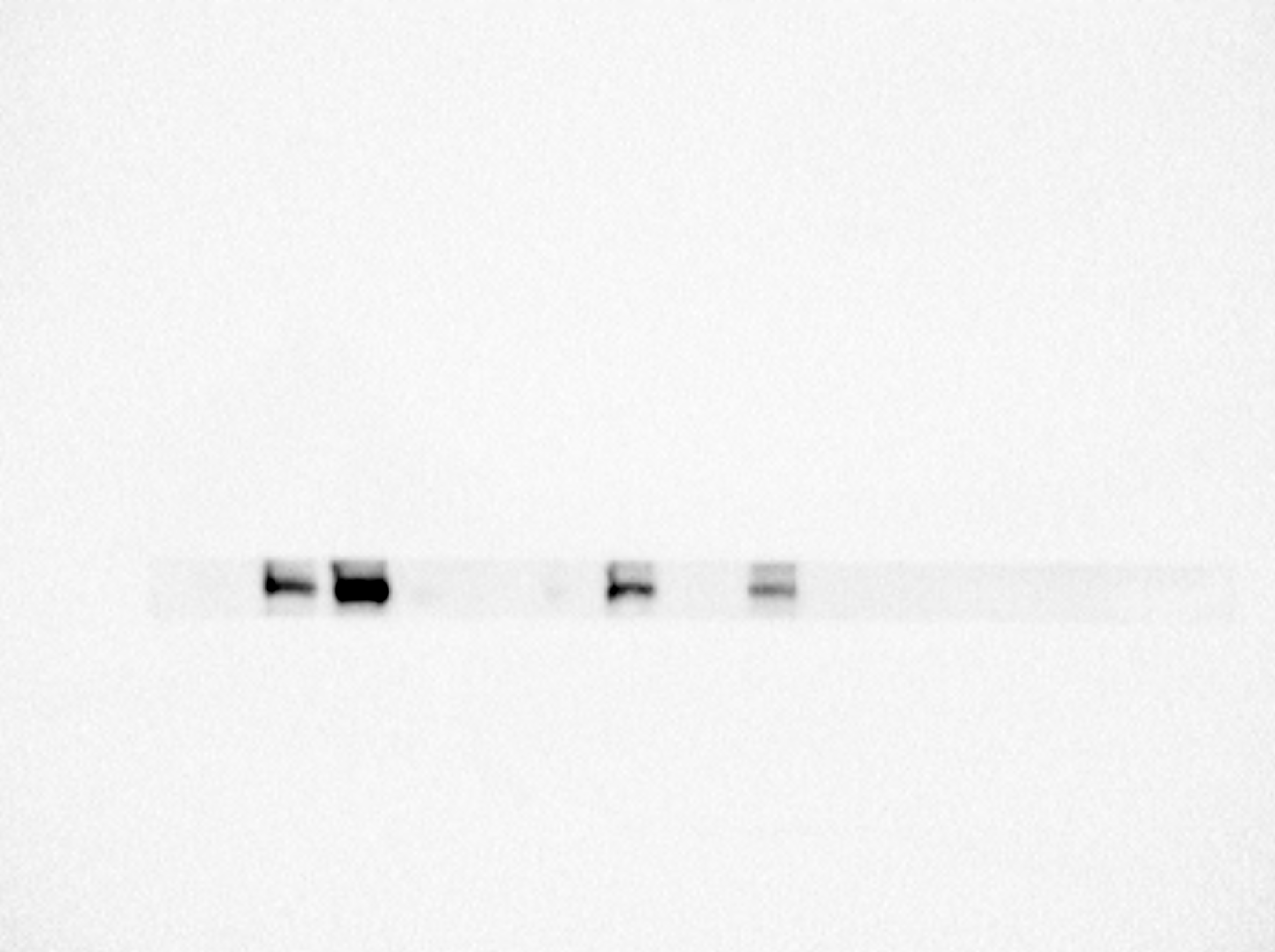

Supplement: Supplementary file 7 — Source data Fig. 5 [file 44321_2026_452_MOESM7_ESM.zip › Figure 5/5B-C/WB_ Uncropped blots_ p-eNOS.tif]

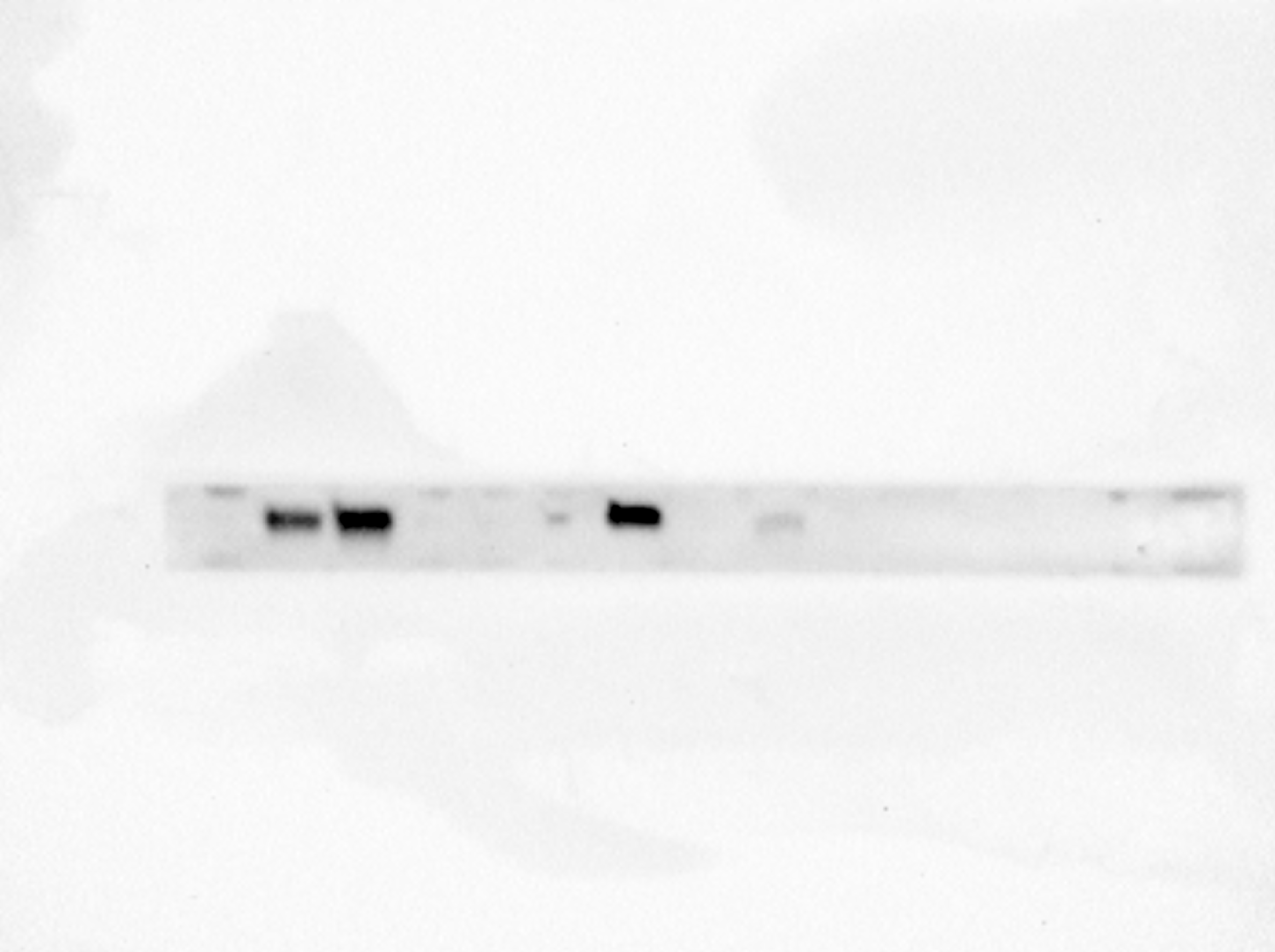

Supplement: Supplementary file 7 — Source data Fig. 5 [file 44321_2026_452_MOESM7_ESM.zip › Figure 5/5B-C/WB_ Uncropped blots_ pAKT.tif]

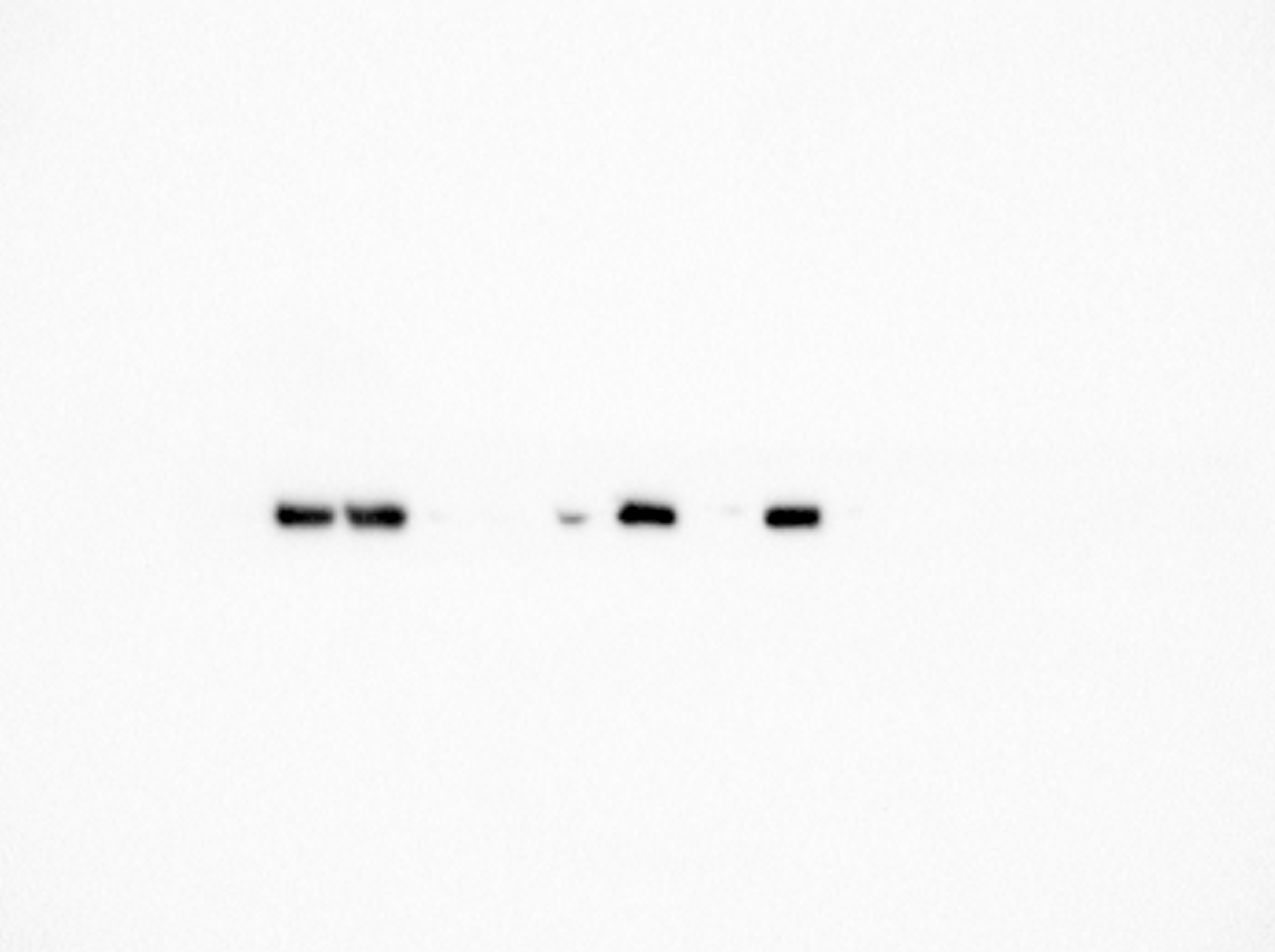

Supplement: Supplementary file 7 — Source data Fig. 5 [file 44321_2026_452_MOESM7_ESM.zip › Figure 5/5B-C/WB_ Uncropped blots_ β-actin.tif]

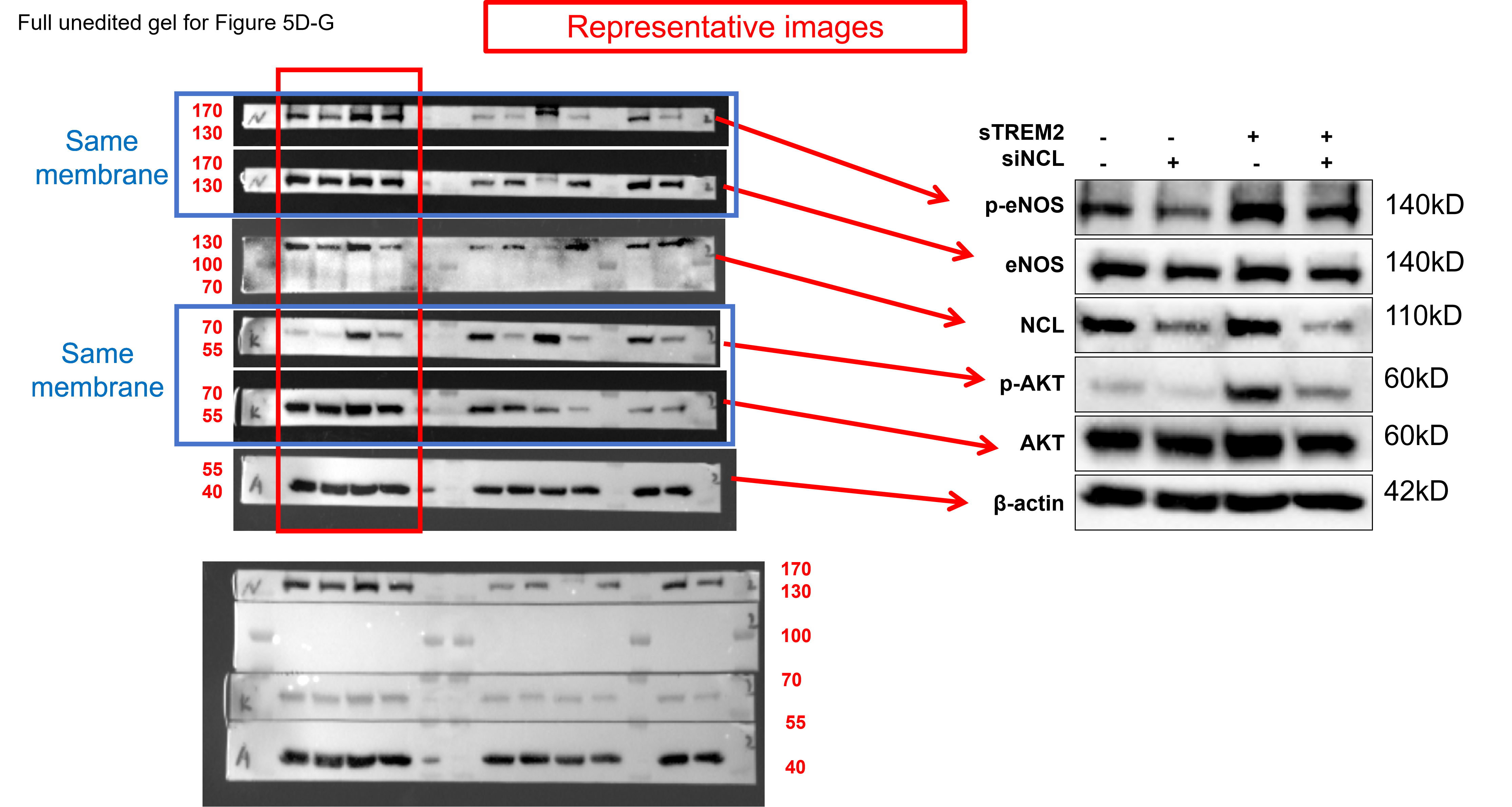

Supplement: Supplementary file 7 — Source data Fig. 5 [file 44321_2026_452_MOESM7_ESM.zip › Figure 5/5D-G/Instructions for cropping Western blot images 1.tif]

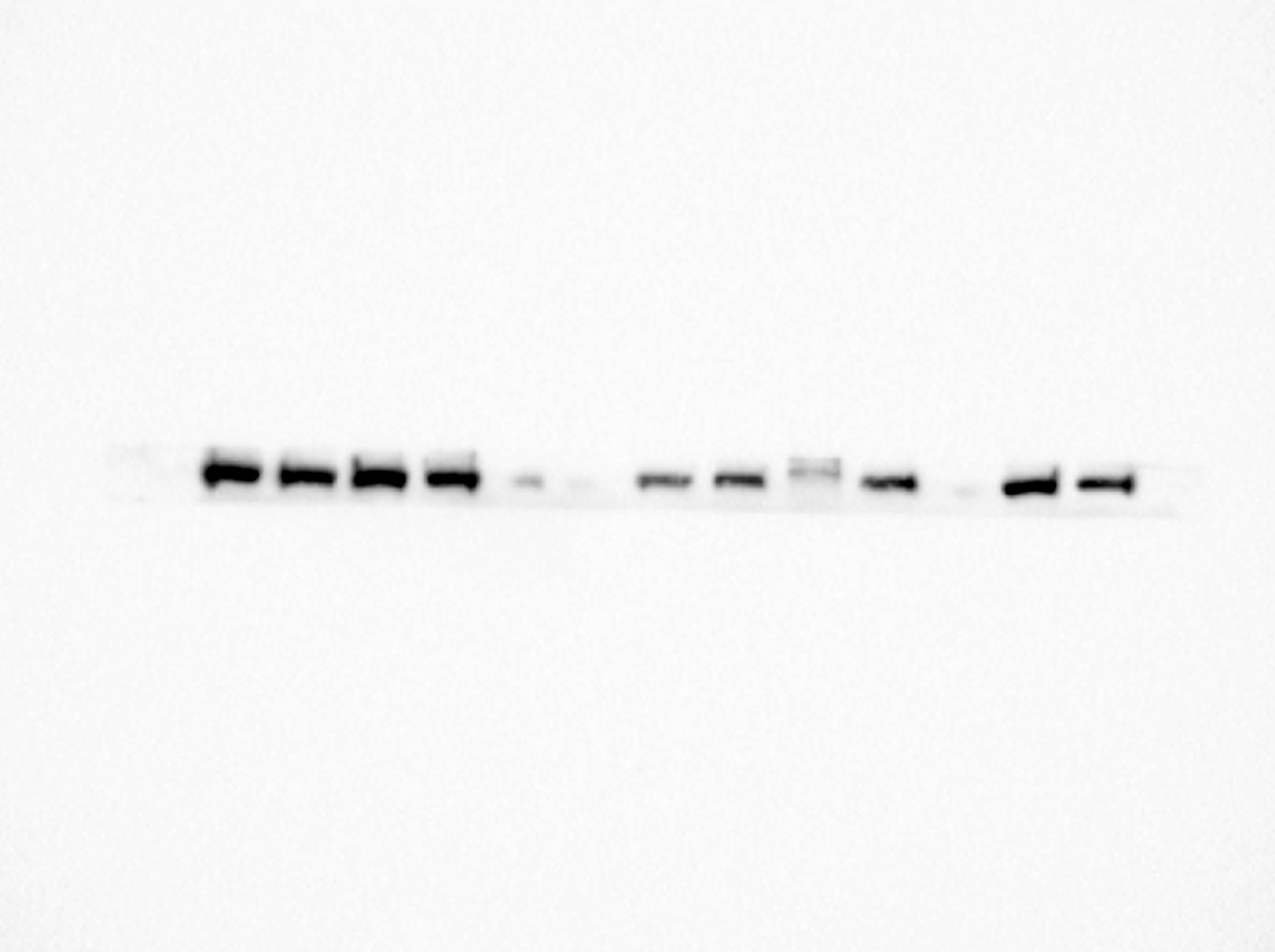

Supplement: Supplementary file 7 — Source data Fig. 5 [file 44321_2026_452_MOESM7_ESM.zip › Figure 5/5D-G/WB_ Uncropped blots_ eNOS.tif]

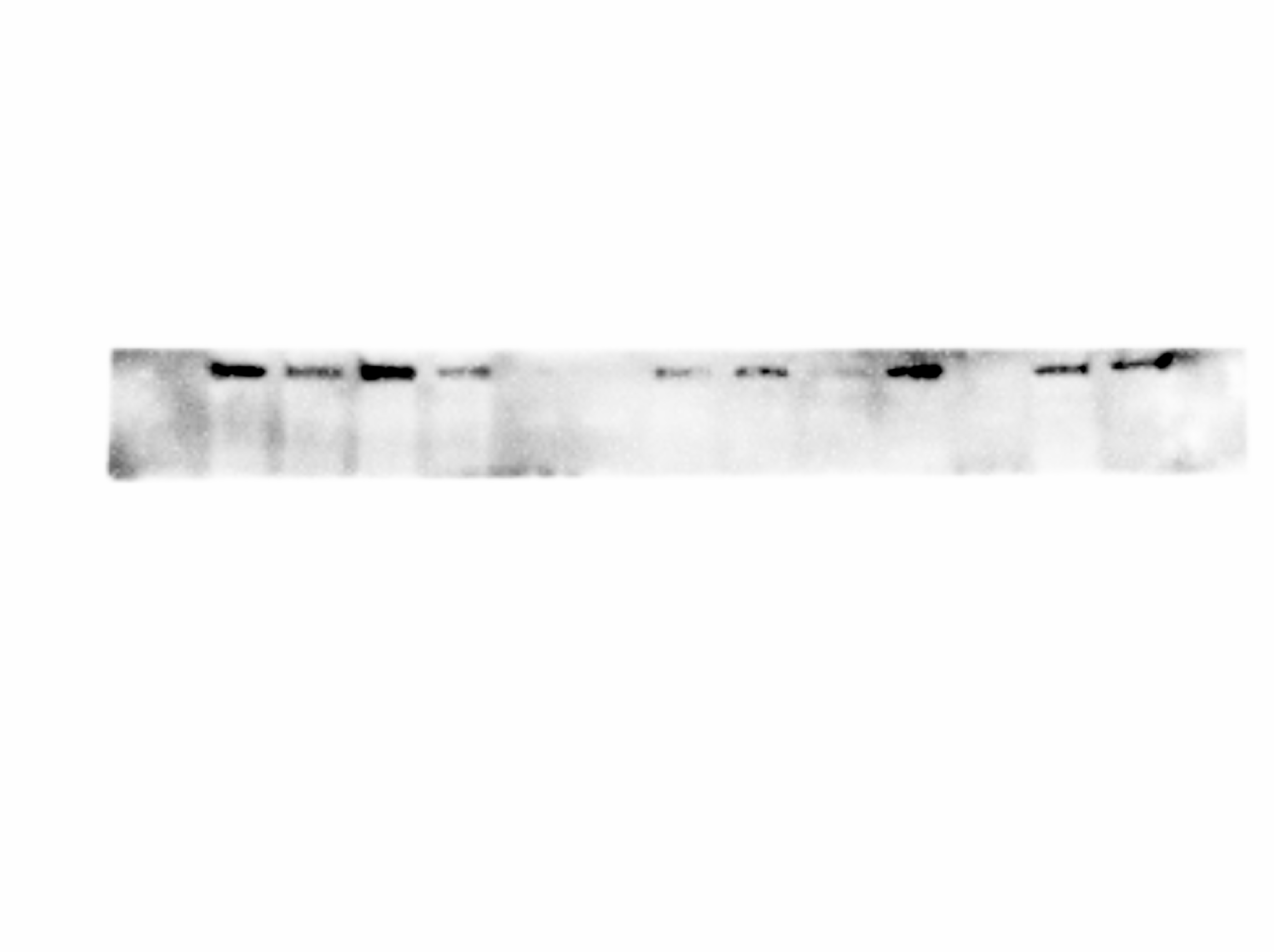

Supplement: Supplementary file 7 — Source data Fig. 5 [file 44321_2026_452_MOESM7_ESM.zip › Figure 5/5D-G/WB_ Uncropped blots_ NCL.tif]

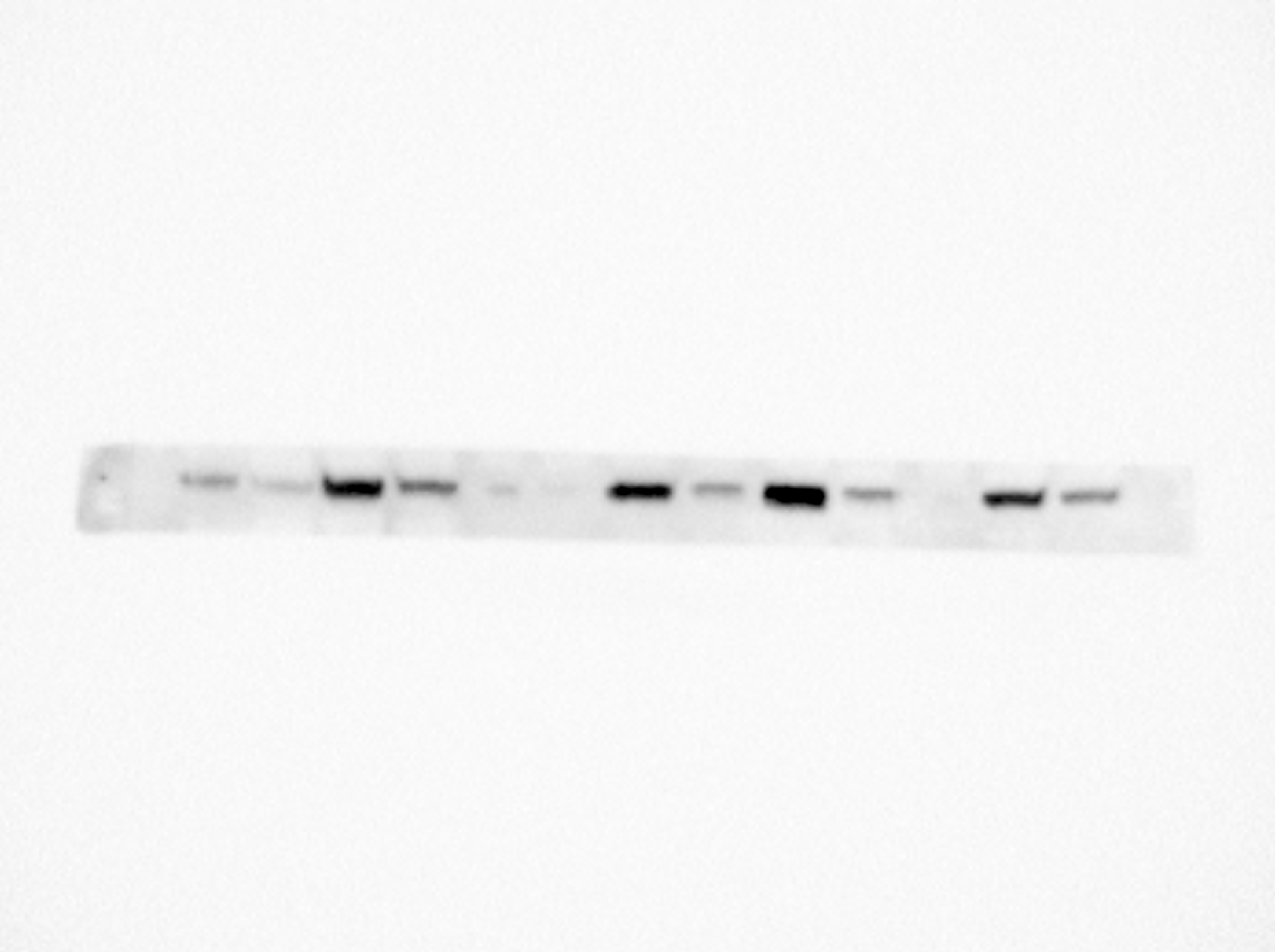

Supplement: Supplementary file 7 — Source data Fig. 5 [file 44321_2026_452_MOESM7_ESM.zip › Figure 5/5D-G/WB_ Uncropped blots_ p-AKT.tif]

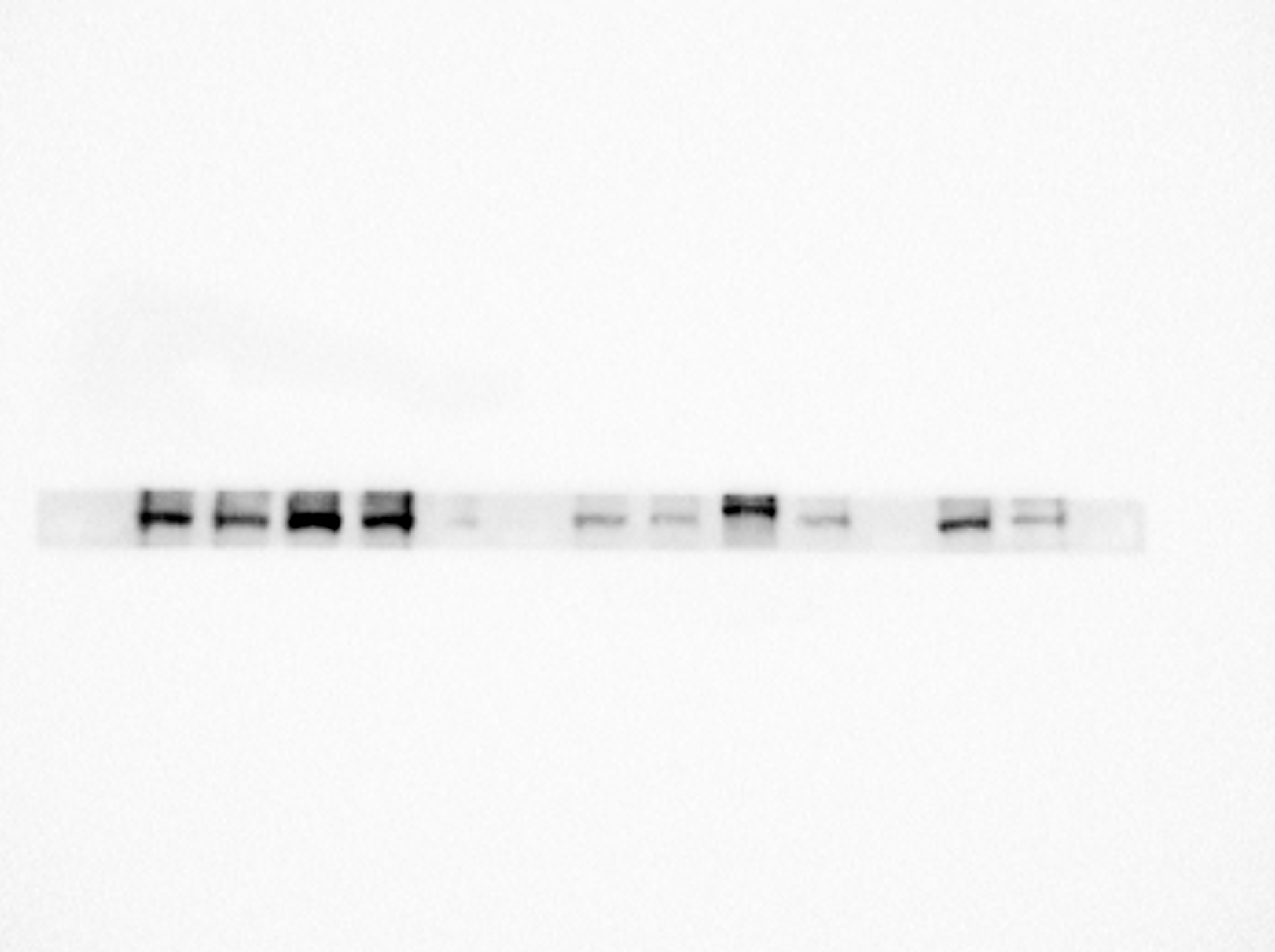

Supplement: Supplementary file 7 — Source data Fig. 5 [file 44321_2026_452_MOESM7_ESM.zip › Figure 5/5D-G/WB_ Uncropped blots_ p-eNOS.tif]

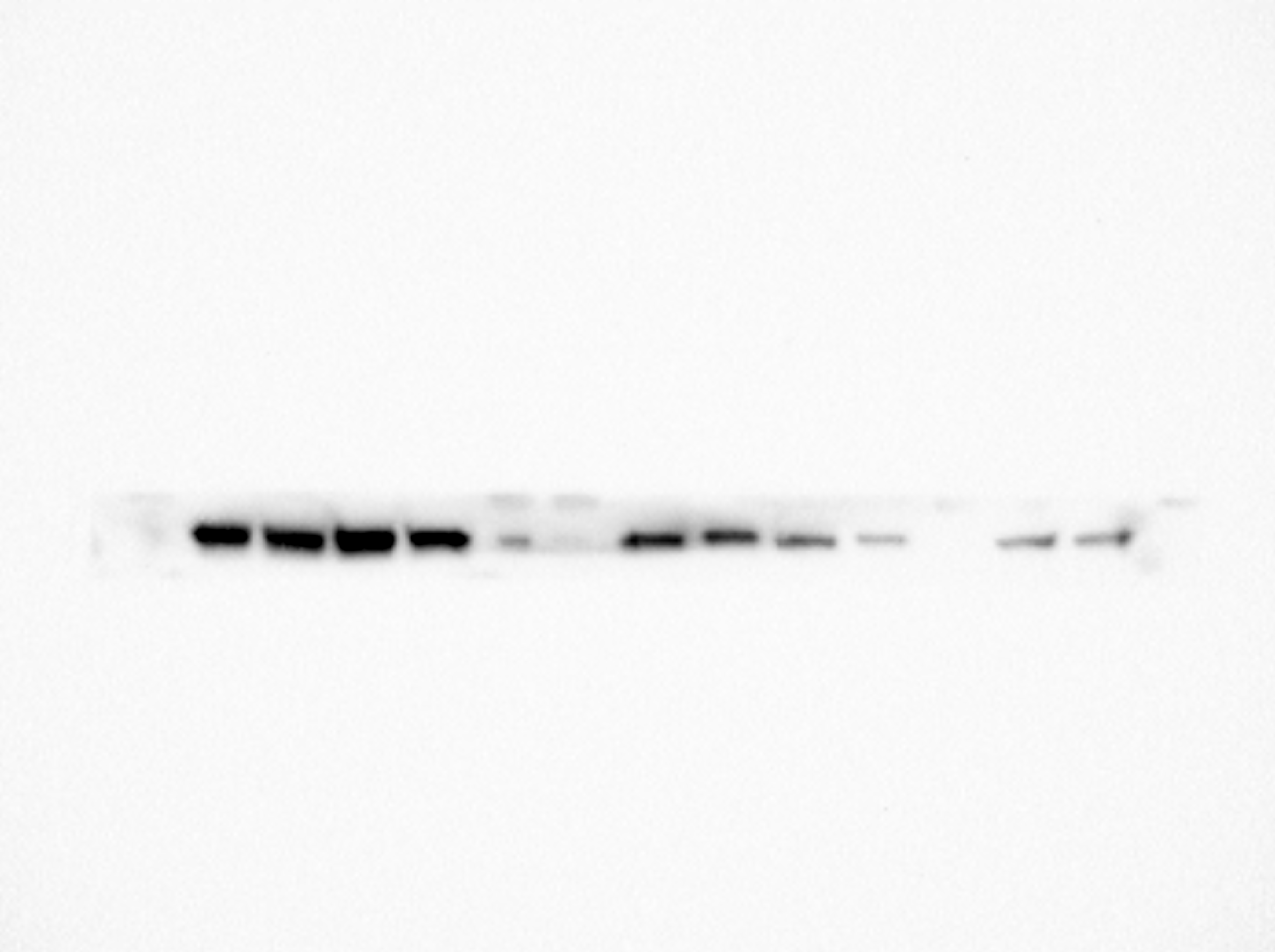

Supplement: Supplementary file 7 — Source data Fig. 5 [file 44321_2026_452_MOESM7_ESM.zip › Figure 5/5D-G/WB_ Uncropped blots_AKT.tif]

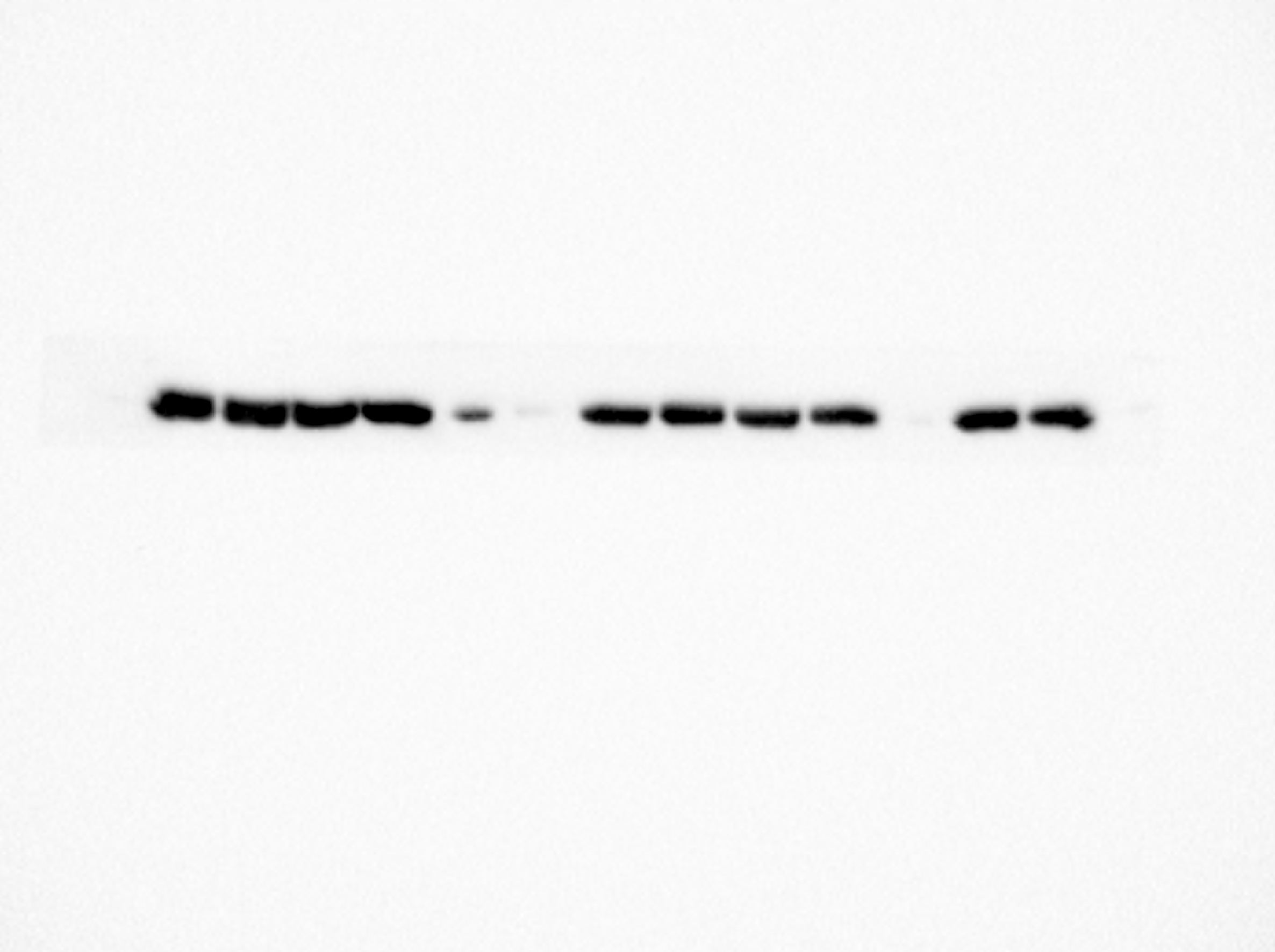

Supplement: Supplementary file 7 — Source data Fig. 5 [file 44321_2026_452_MOESM7_ESM.zip › Figure 5/5D-G/WB_ Uncropped blots_β-actin.tif]

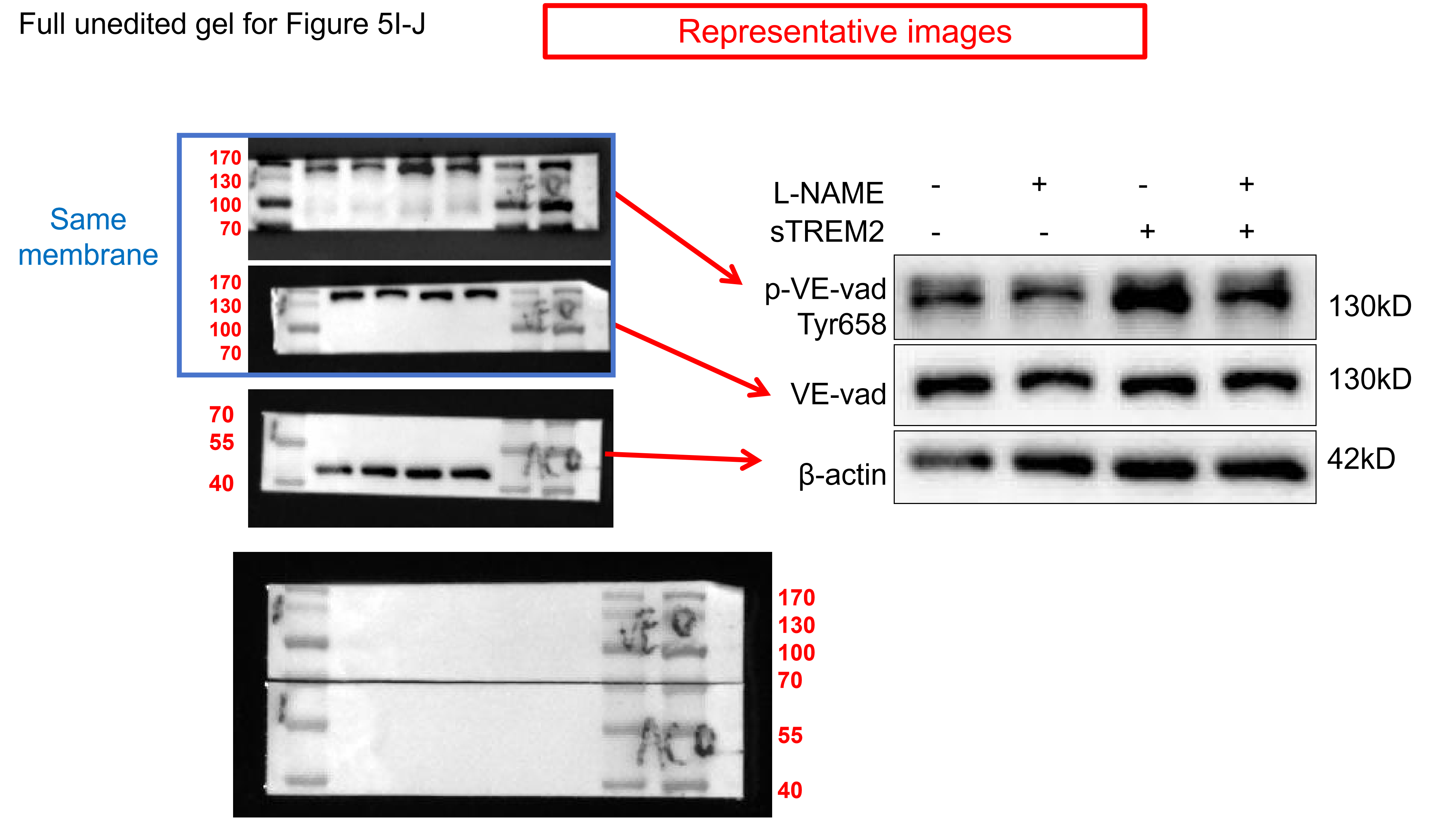

Supplement: Supplementary file 7 — Source data Fig. 5 [file 44321_2026_452_MOESM7_ESM.zip › Figure 5/5I-J/Instructions for cropping Western blot images 1.tif]

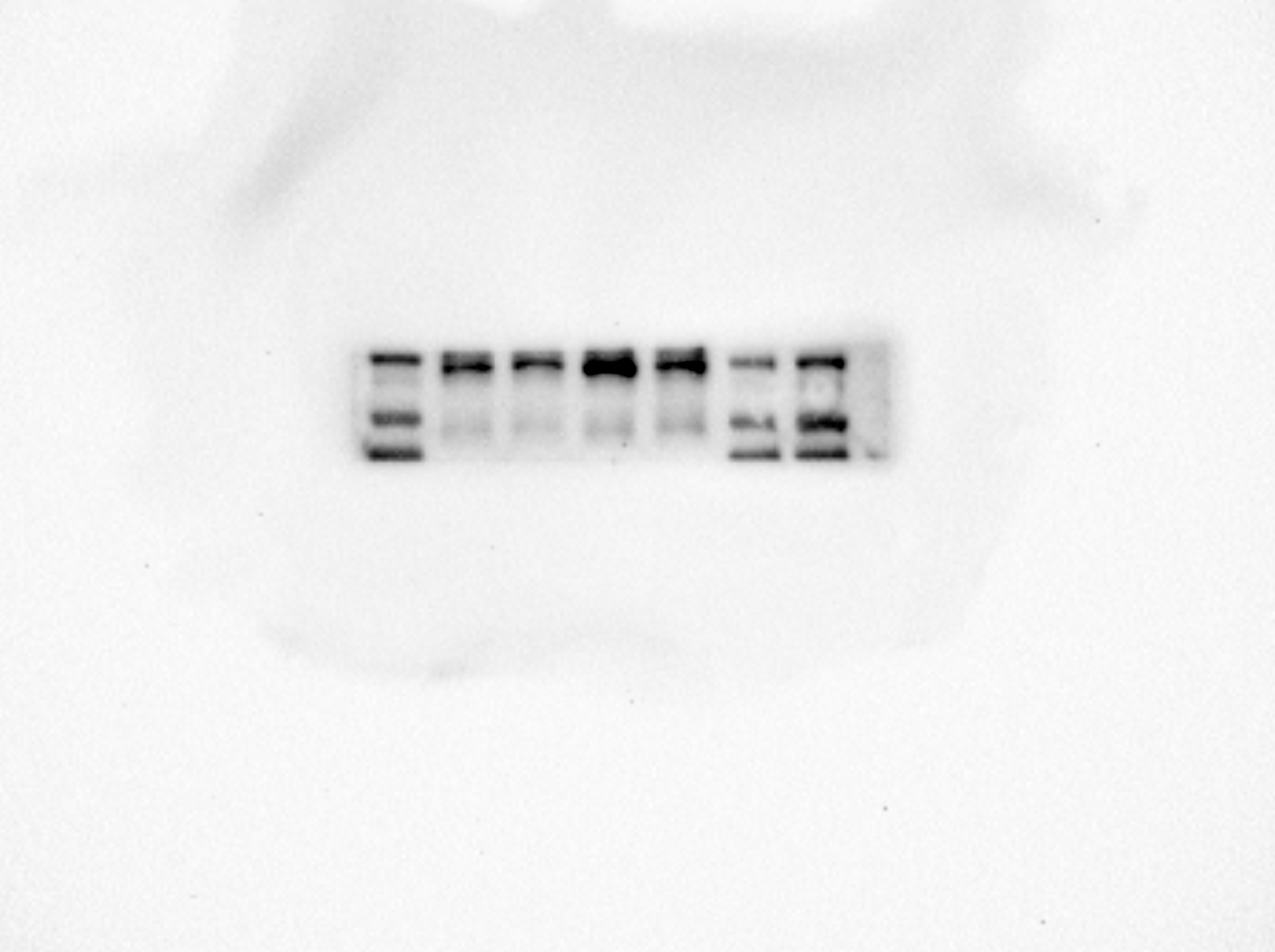

Supplement: Supplementary file 7 — Source data Fig. 5 [file 44321_2026_452_MOESM7_ESM.zip › Figure 5/5I-J/WB_ Uncropped blots_ p-VEcad.tif]

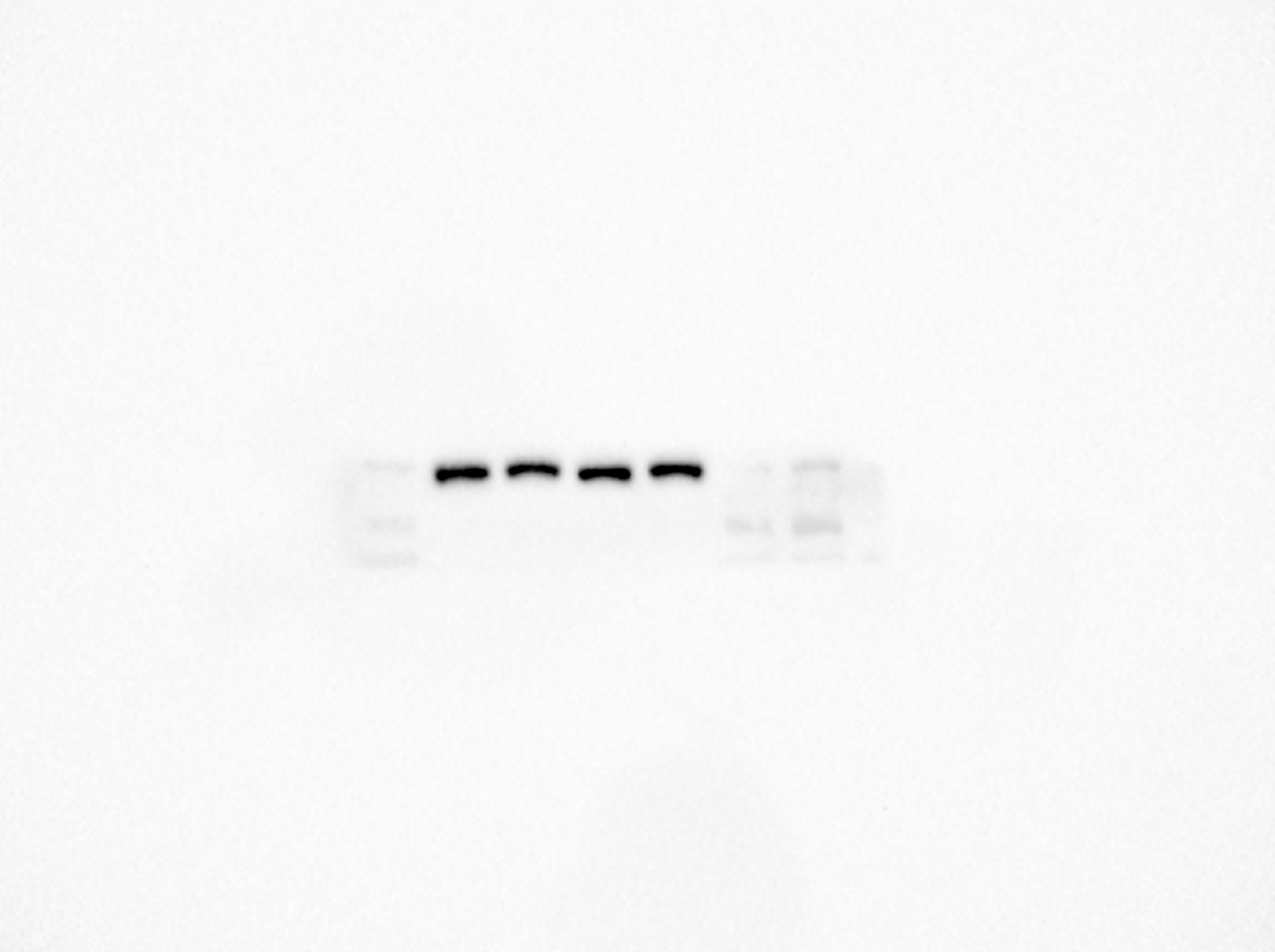

Supplement: Supplementary file 7 — Source data Fig. 5 [file 44321_2026_452_MOESM7_ESM.zip › Figure 5/5I-J/WB_ Uncropped blots_ VEcad.tif]

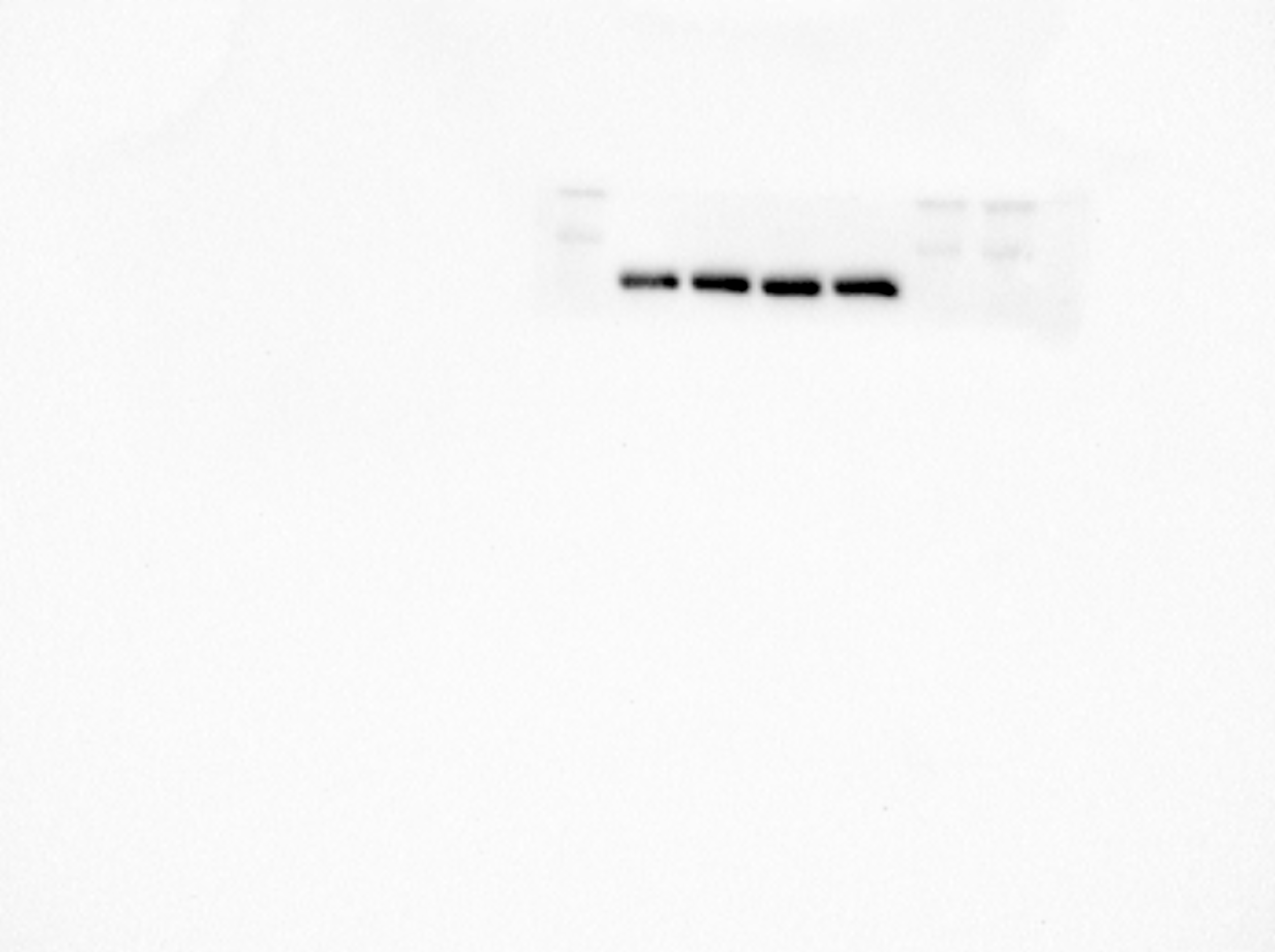

Supplement: Supplementary file 7 — Source data Fig. 5 [file 44321_2026_452_MOESM7_ESM.zip › Figure 5/5I-J/WB_ Uncropped blots_ β-actin.tif]

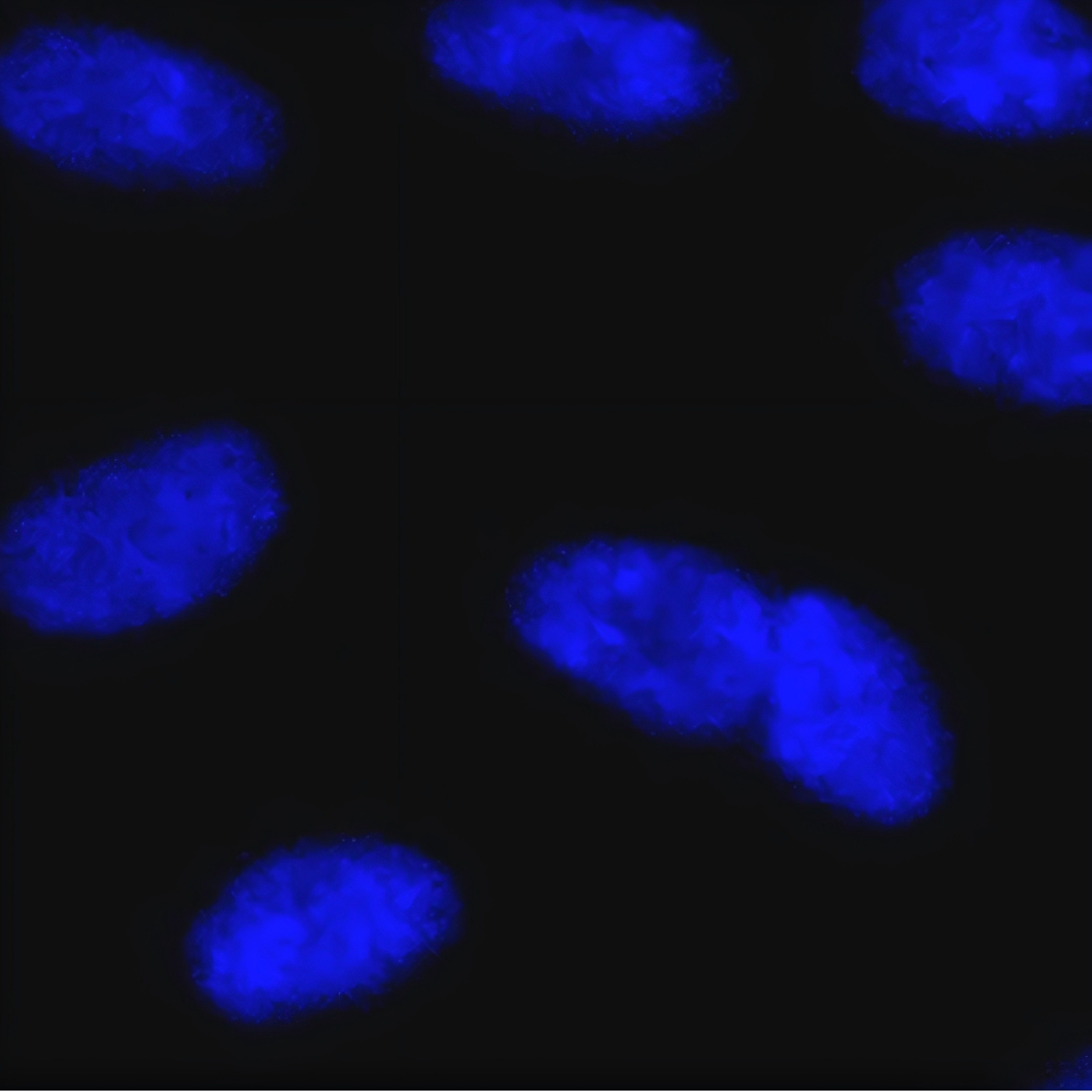

Supplement: Supplementary file 7 — Source data Fig. 5 [file 44321_2026_452_MOESM7_ESM.zip › Figure 5/5K-L/Figure 5K sTREM2 DAPI.tif]

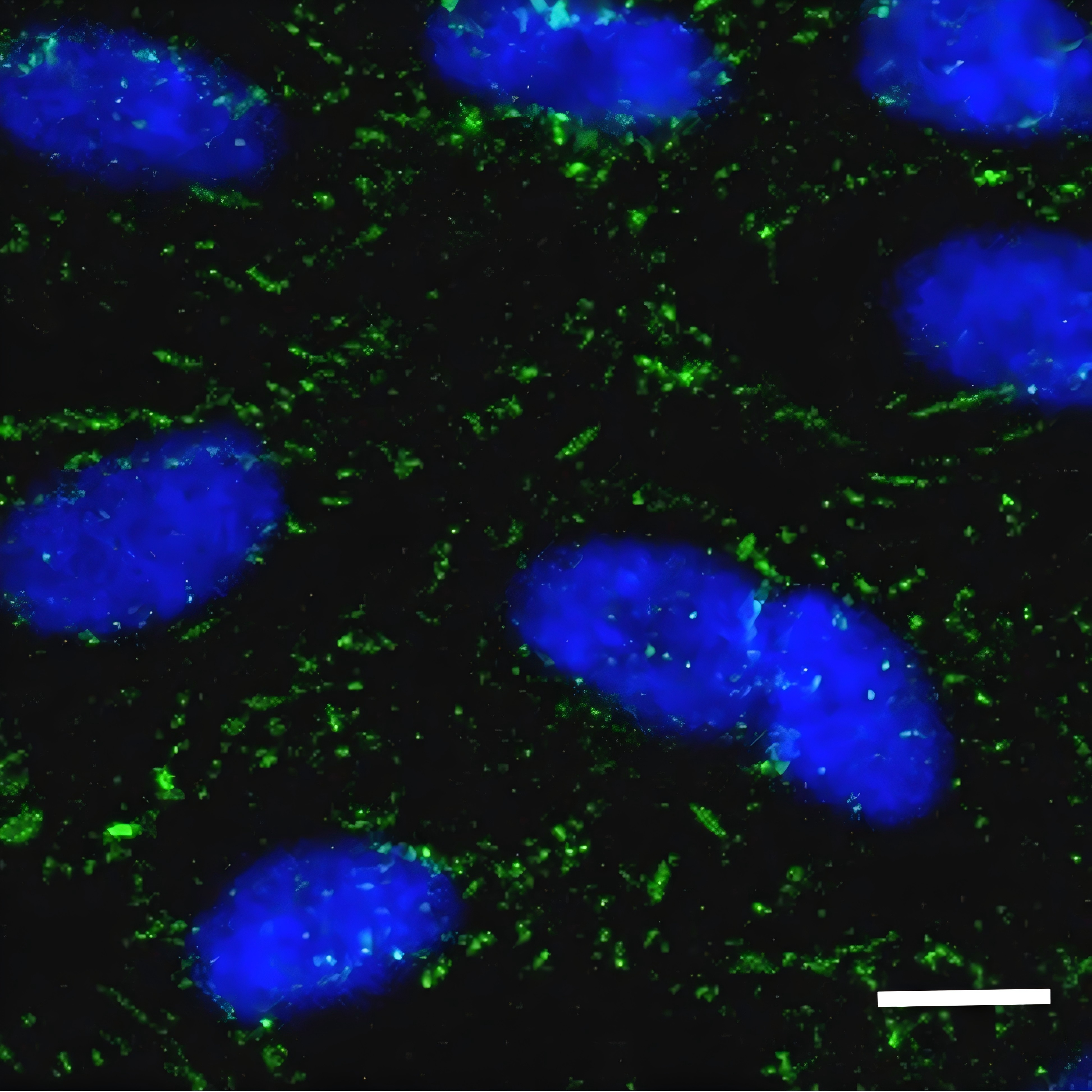

Supplement: Supplementary file 7 — Source data Fig. 5 [file 44321_2026_452_MOESM7_ESM.zip › Figure 5/5K-L/Figure 5K sTREM2 Merge.tif]

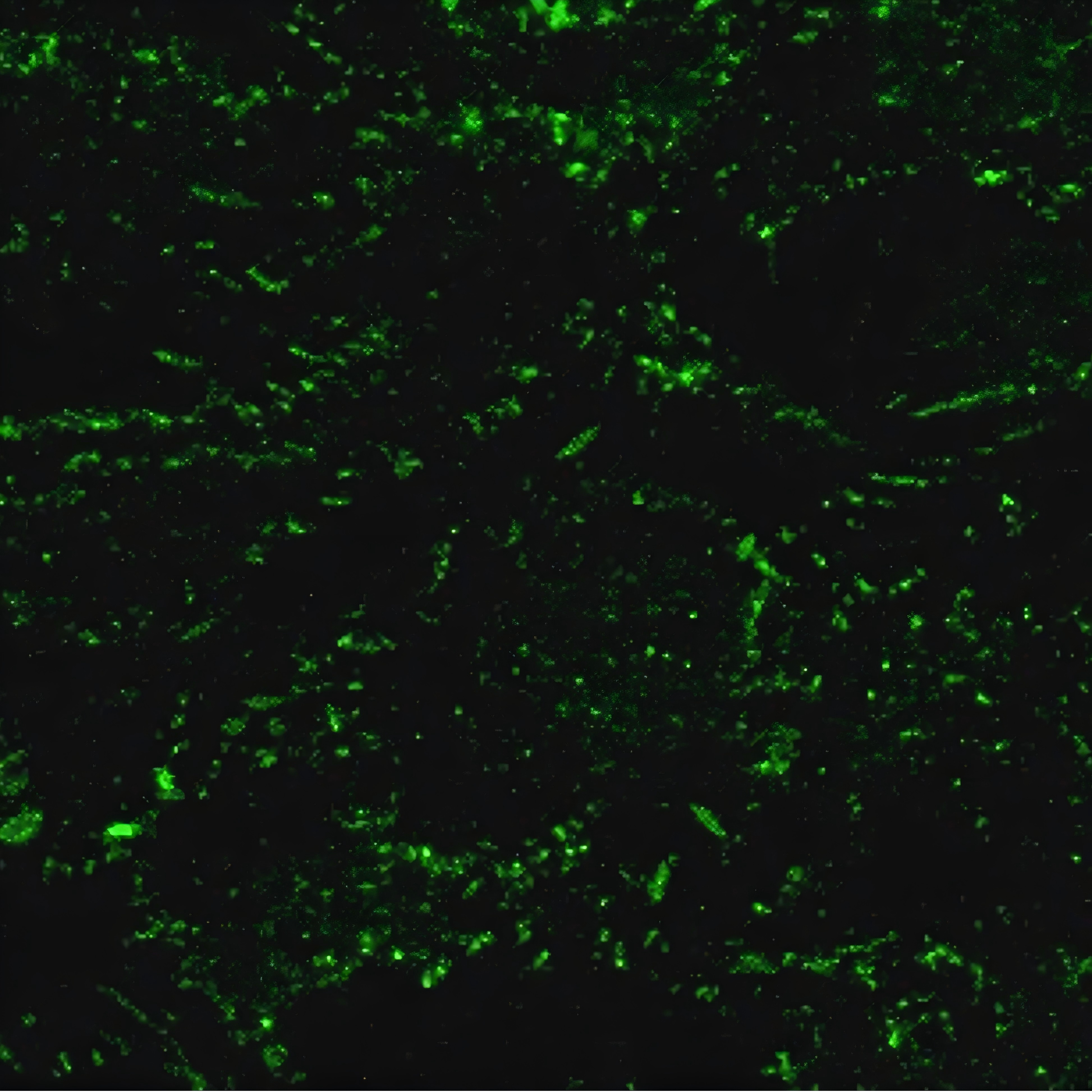

Supplement: Supplementary file 7 — Source data Fig. 5 [file 44321_2026_452_MOESM7_ESM.zip › Figure 5/5K-L/Figure 5K sTREM2 p-VE-cad.tif]

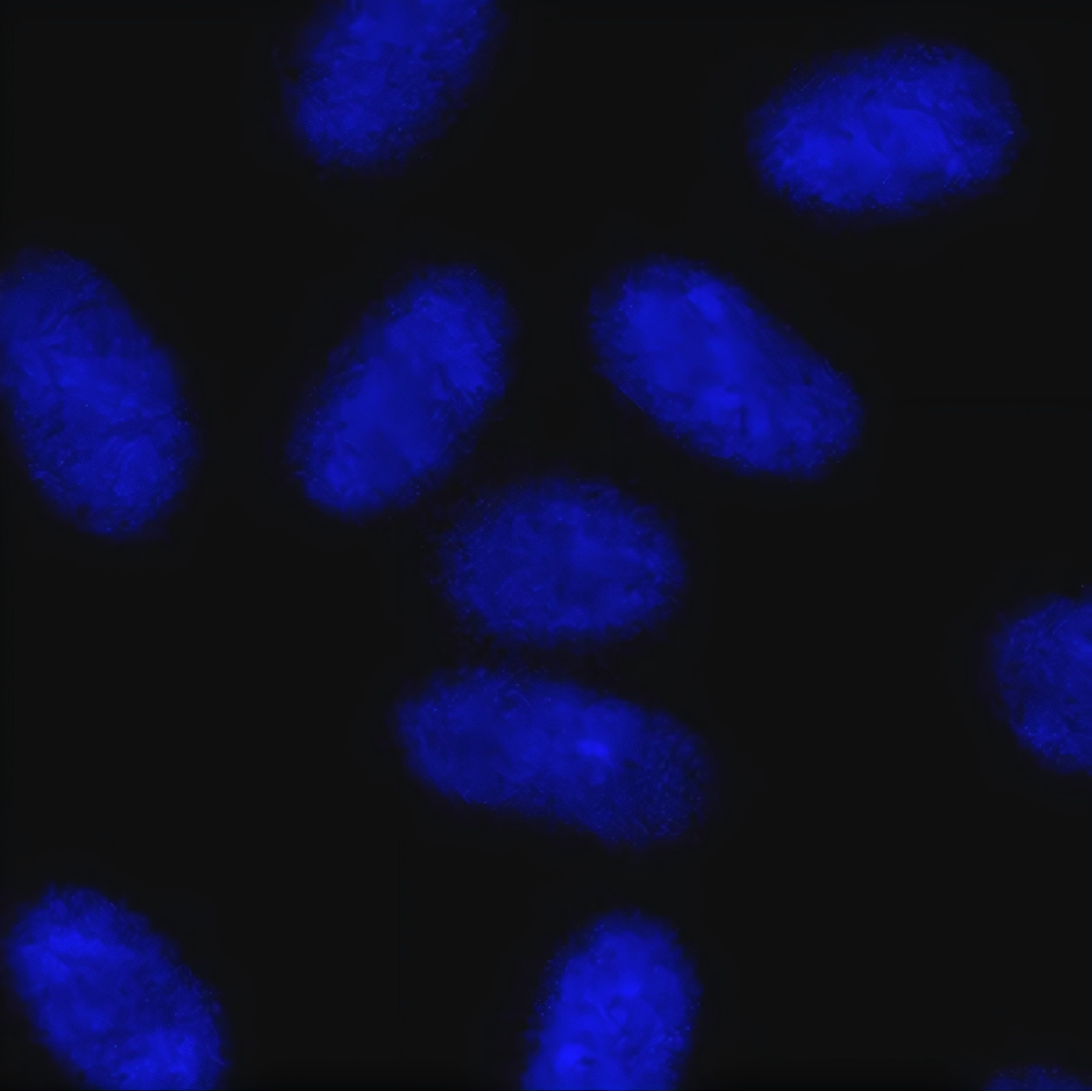

Supplement: Supplementary file 7 — Source data Fig. 5 [file 44321_2026_452_MOESM7_ESM.zip › Figure 5/5K-L/Figure 5K sTREM2+L-NAME DAPI.tif]

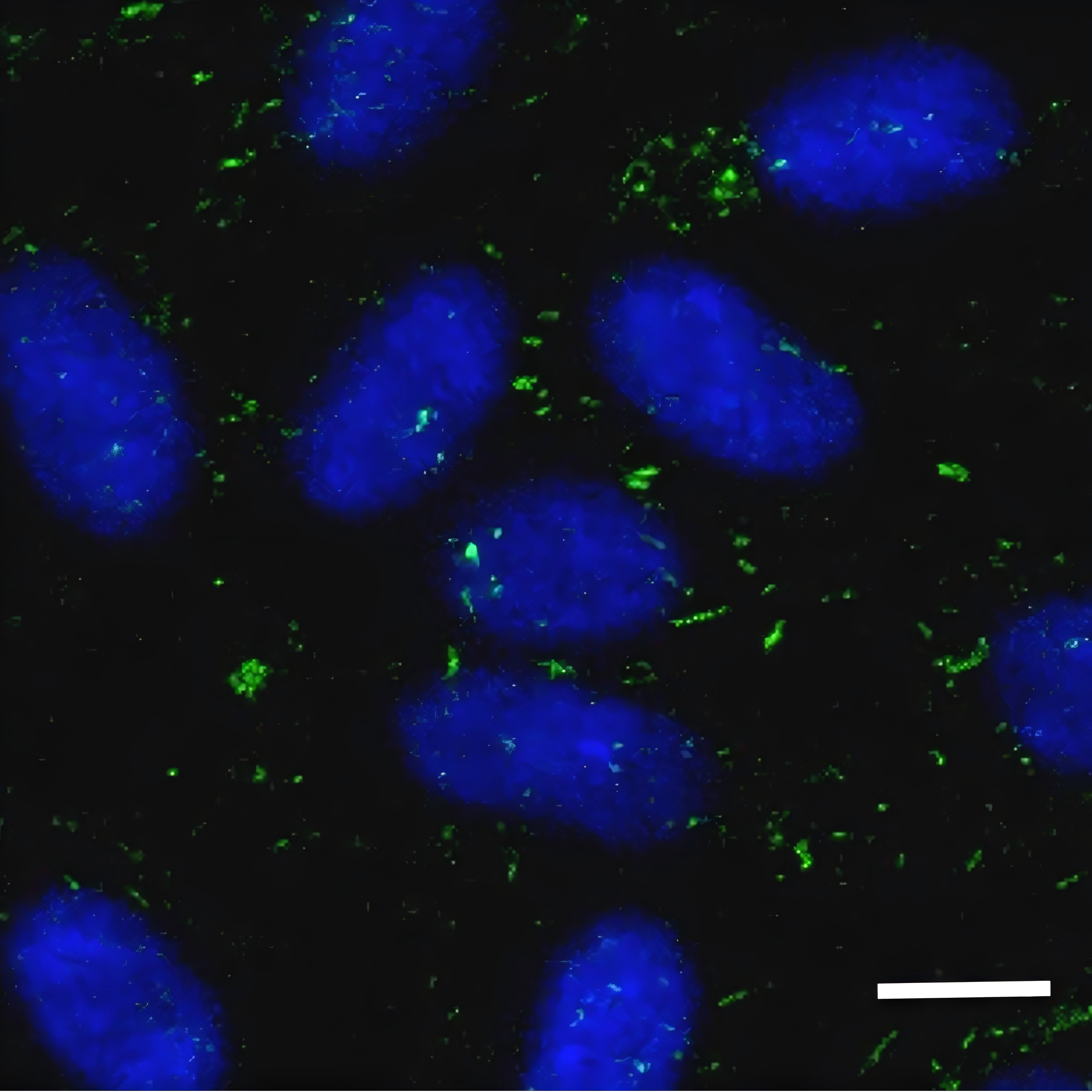

Supplement: Supplementary file 7 — Source data Fig. 5 [file 44321_2026_452_MOESM7_ESM.zip › Figure 5/5K-L/Figure 5K sTREM2+L-NAME Merge.tif]

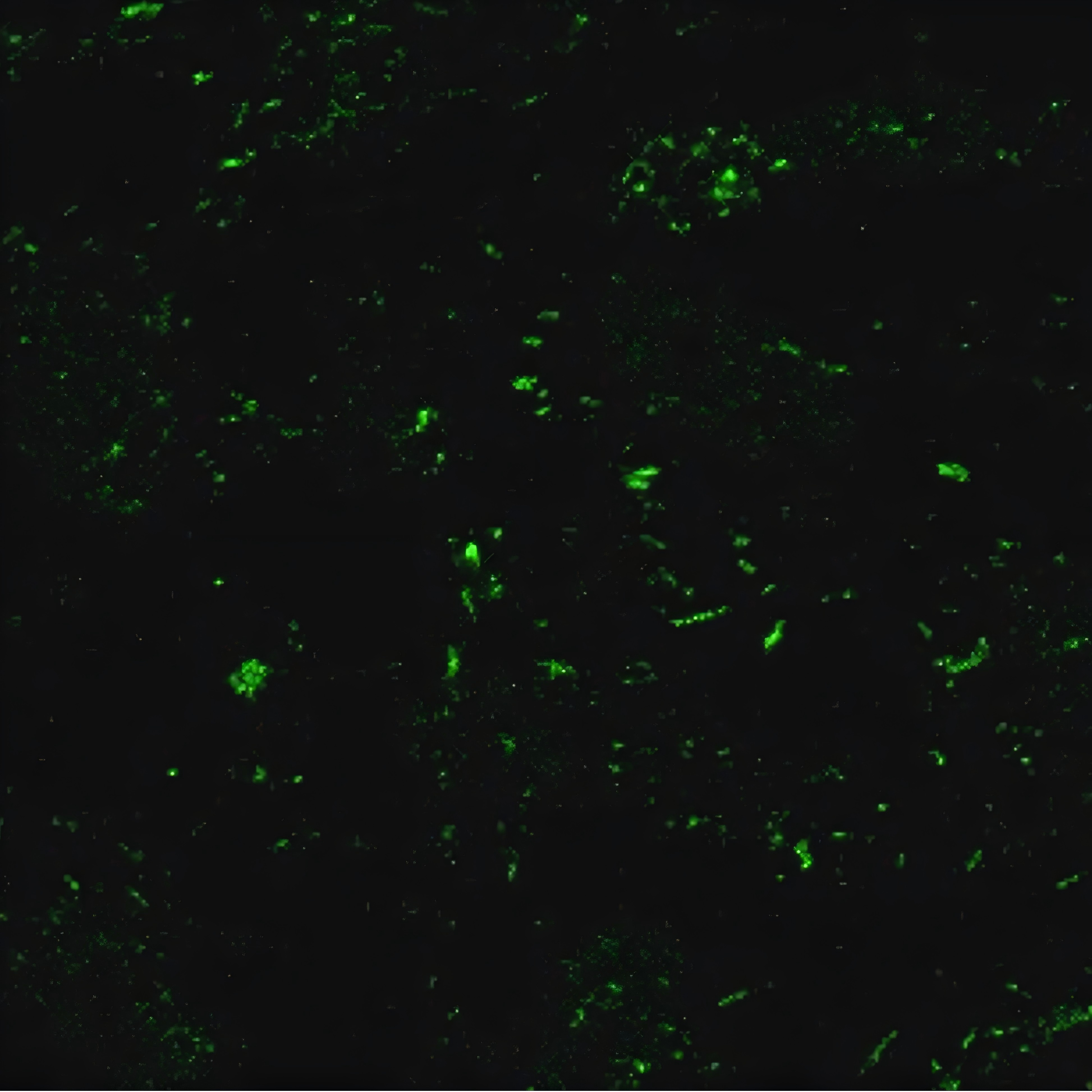

Supplement: Supplementary file 7 — Source data Fig. 5 [file 44321_2026_452_MOESM7_ESM.zip › Figure 5/5K-L/Figure 5K sTREM2+L-NAME p-VE-cad.tif]

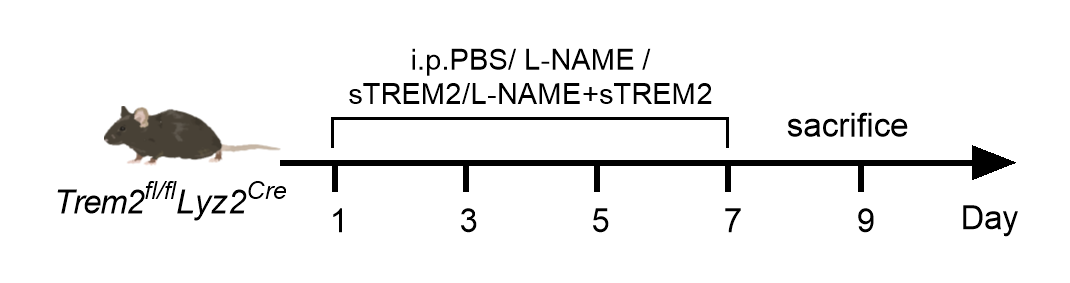

Supplement: Supplementary file 7 — Source data Fig. 5 [file 44321_2026_452_MOESM7_ESM.zip › Figure 5/5M/Fig5M.tif]

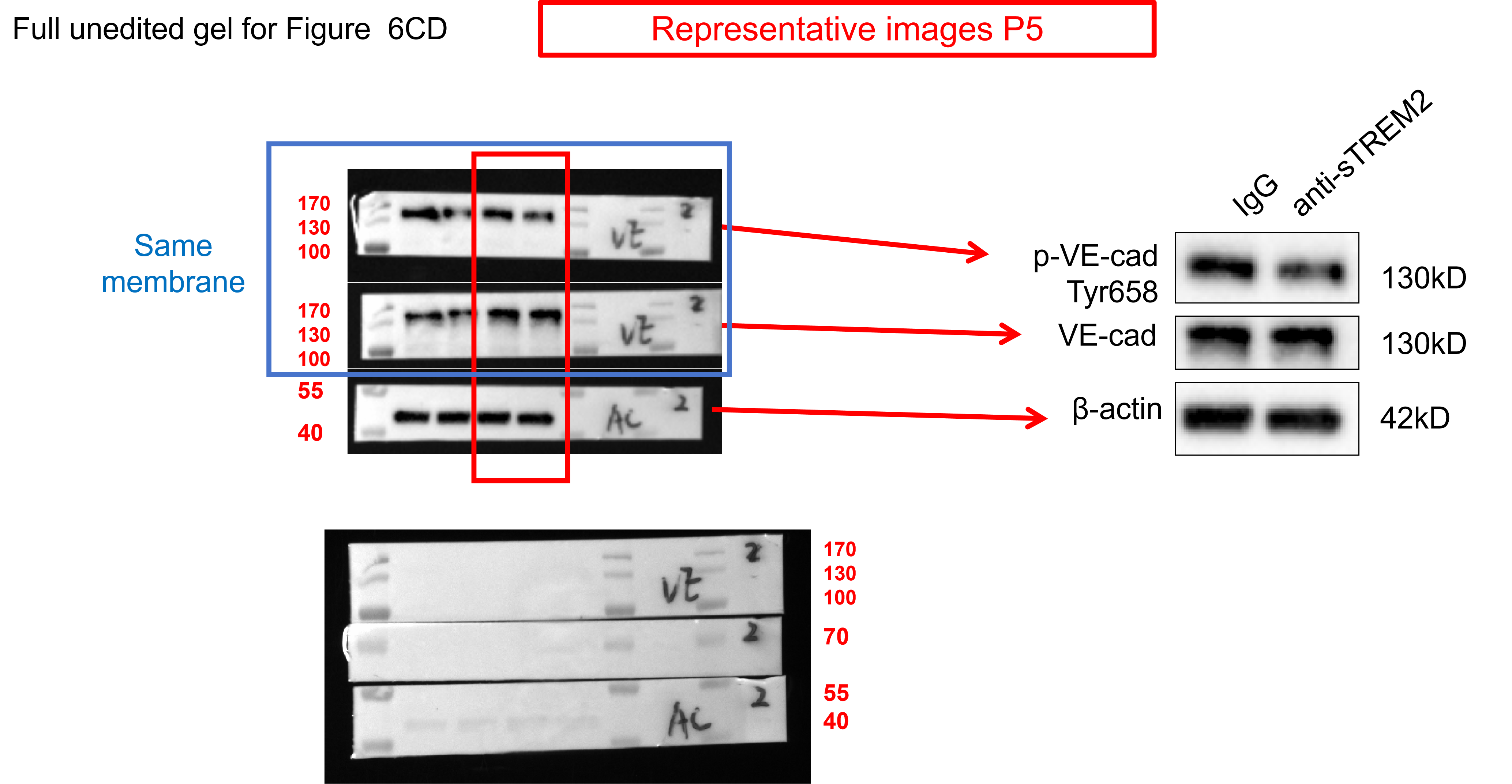

Supplement: Supplementary file 8 — Source data Fig. 6 [file 44321_2026_452_MOESM8_ESM.zip › Figure 6/6C-D/Instructions for cropping Western blot images P5.tif]

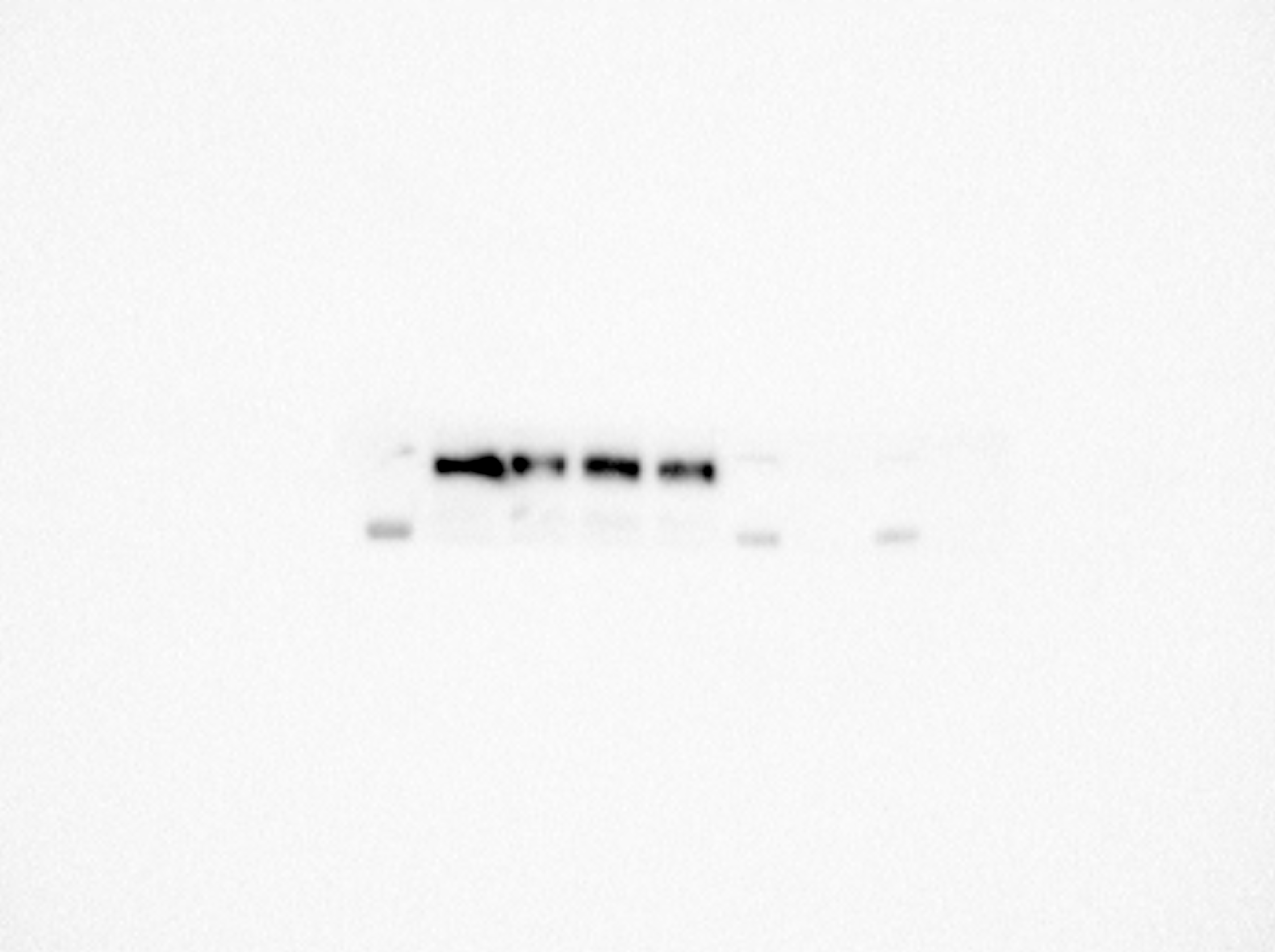

Supplement: Supplementary file 8 — Source data Fig. 6 [file 44321_2026_452_MOESM8_ESM.zip › Figure 6/6C-D/WB_ Uncropped blots_ p-VEcad.tif]
