# Supplementary figures and images for: Soluble TREM2 engages cell-surface nucleolin to drive vascular permeability and malignant ascites in ovarian cancer (part 2 of 2)
Source: EMBO Mol Med. 2026 May 26;18(7):2667–90. doi: 10.1038/s44321-026-00452-2 (PMC13365401; doi:10.1038/s44321-026-00452-2)

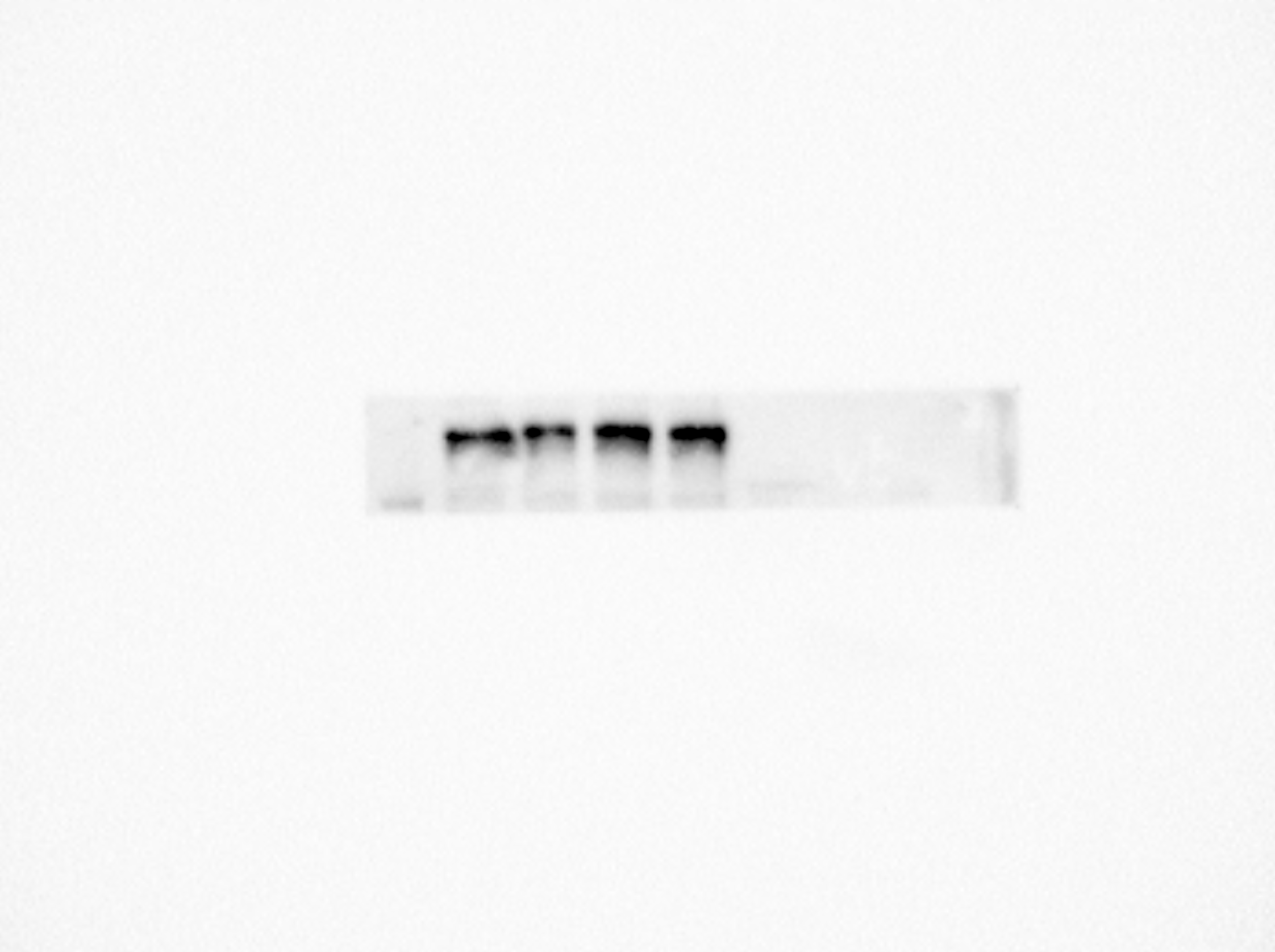

Supplement: Supplementary file 8 — Source data Fig. 6 [file 44321_2026_452_MOESM8_ESM.zip › Figure 6/6C-D/WB_ Uncropped blots_ VEcad.tif]

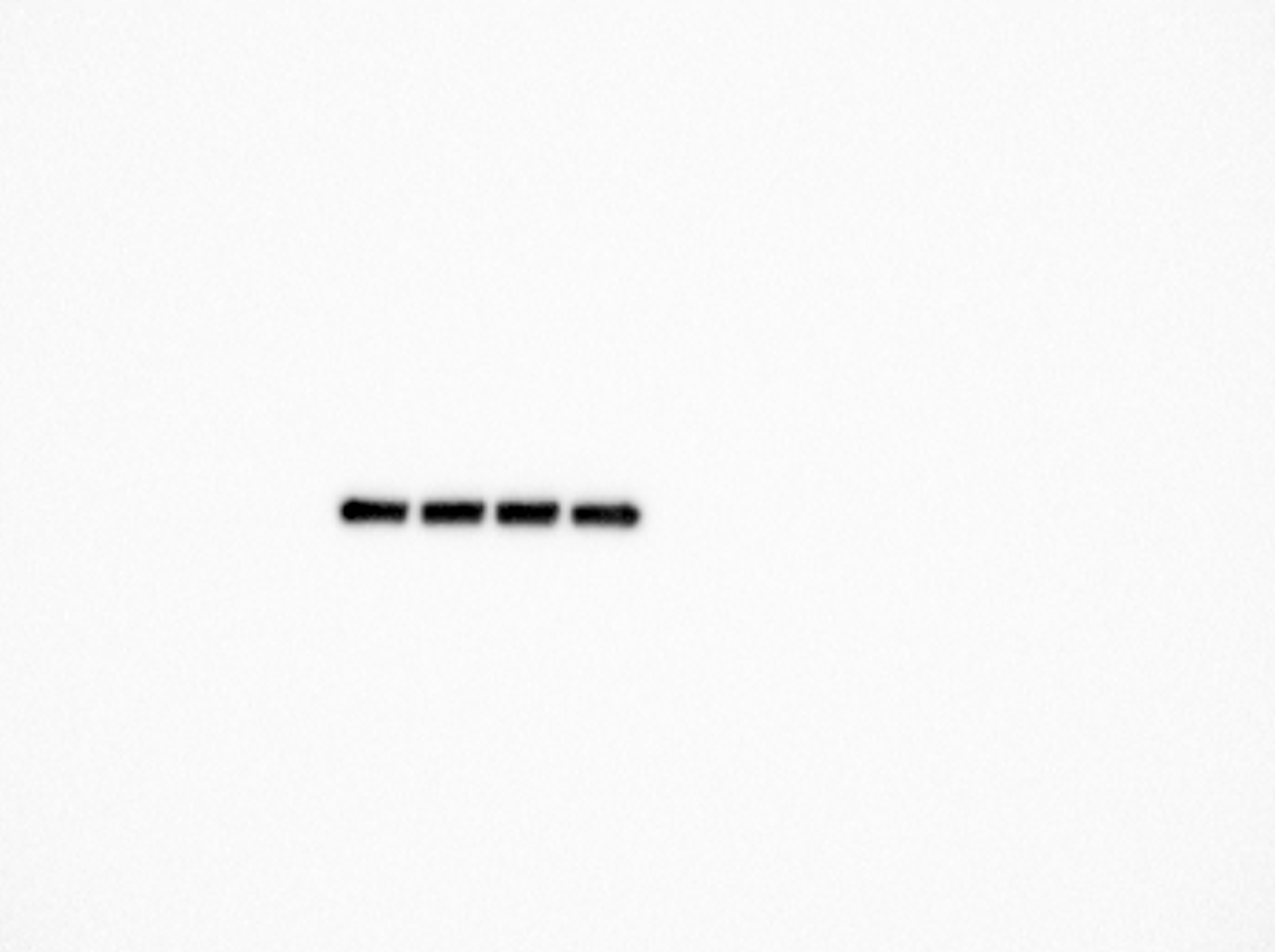

Supplement: Supplementary file 8 — Source data Fig. 6 [file 44321_2026_452_MOESM8_ESM.zip › Figure 6/6C-D/WB_ Uncropped blots_ β-actin.tif]

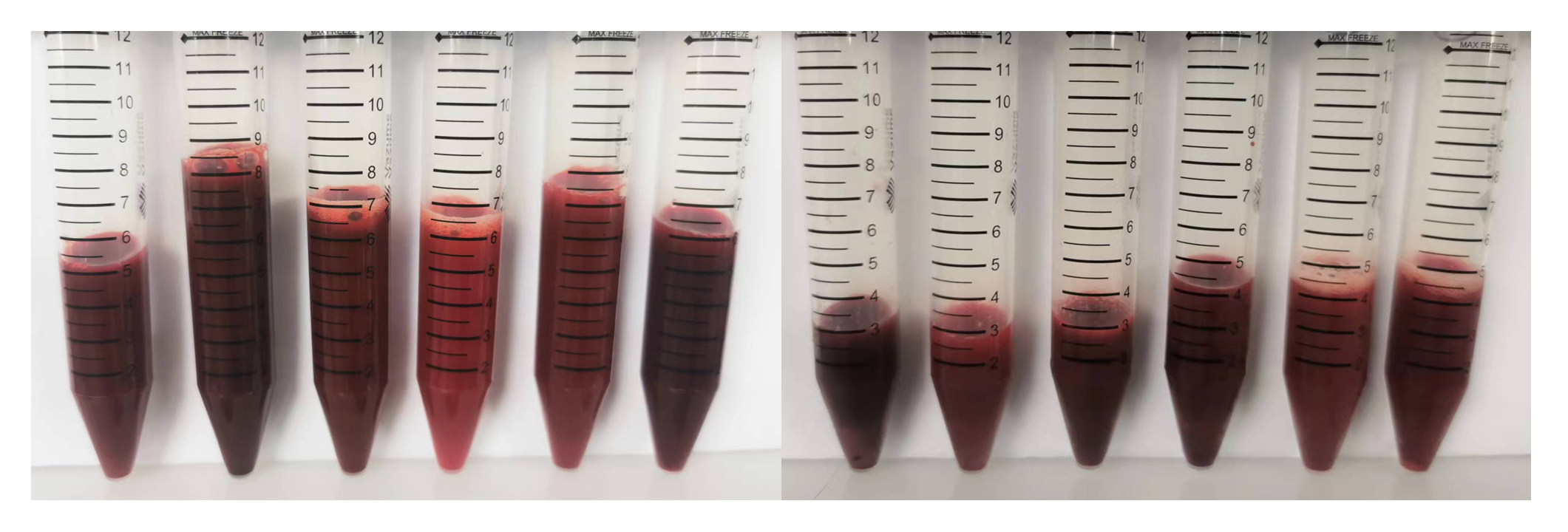

Supplement: Supplementary file 8 — Source data Fig. 6 [file 44321_2026_452_MOESM8_ESM.zip › Figure 6/6E-H/Fig 6E.tif]

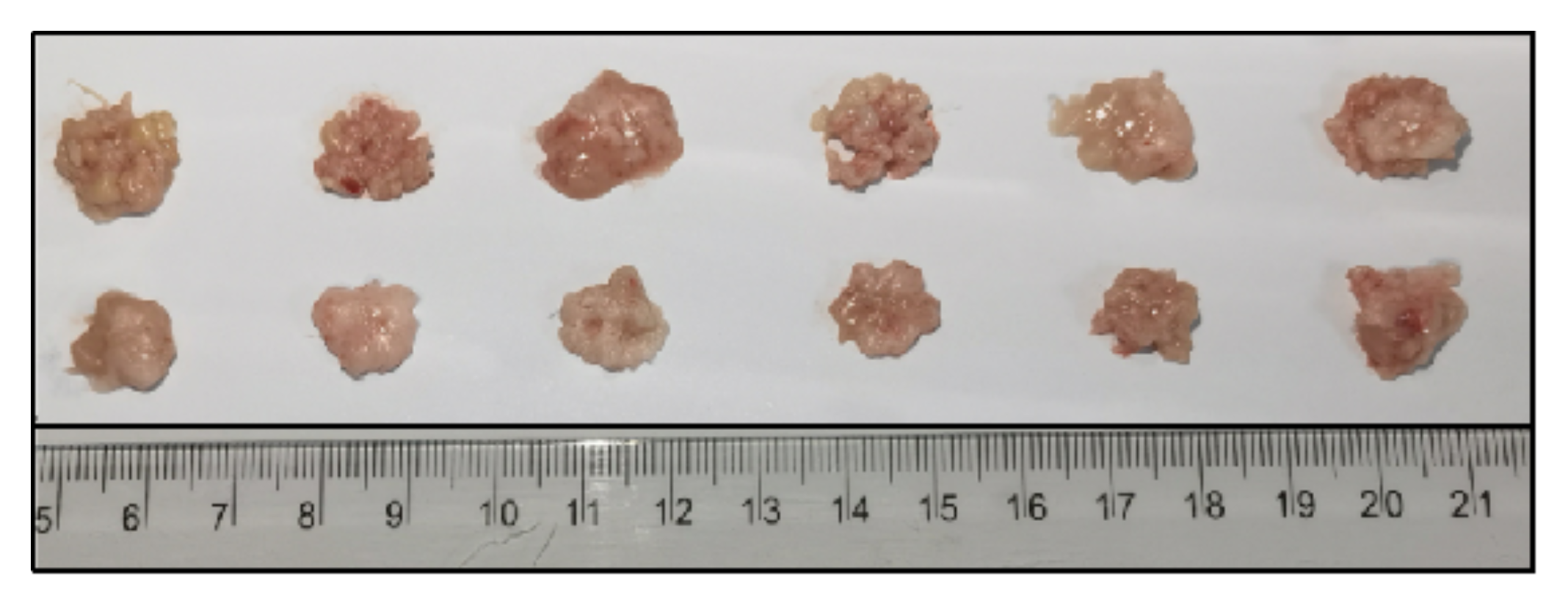

Supplement: Supplementary file 8 — Source data Fig. 6 [file 44321_2026_452_MOESM8_ESM.zip › Figure 6/6E-H/Fig 6F.tif]

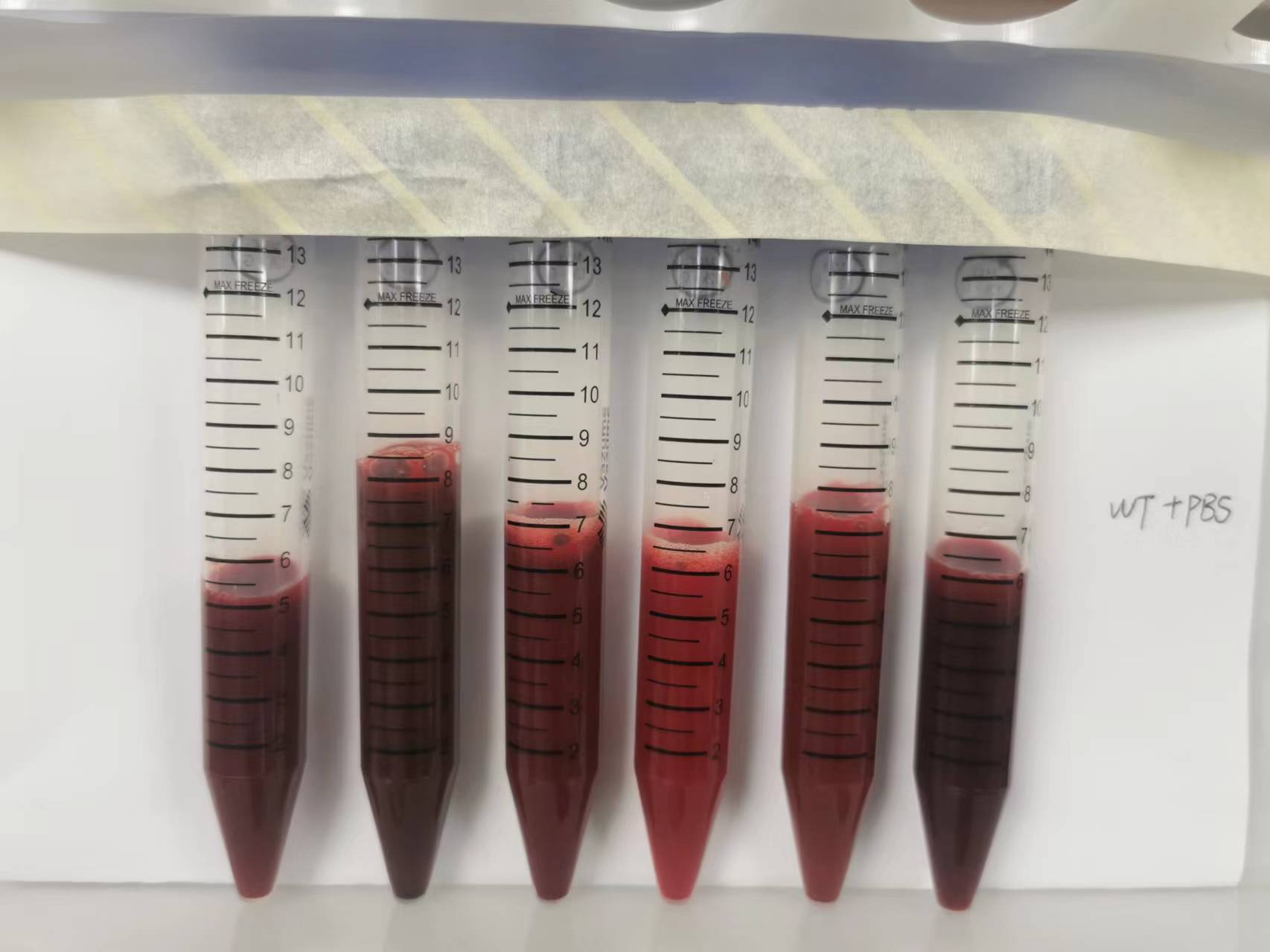

Supplement: Supplementary file 8 — Source data Fig. 6 [file 44321_2026_452_MOESM8_ESM.zip › Figure 6/6E-H/Figure 6E (left) WT PBS.jpg]

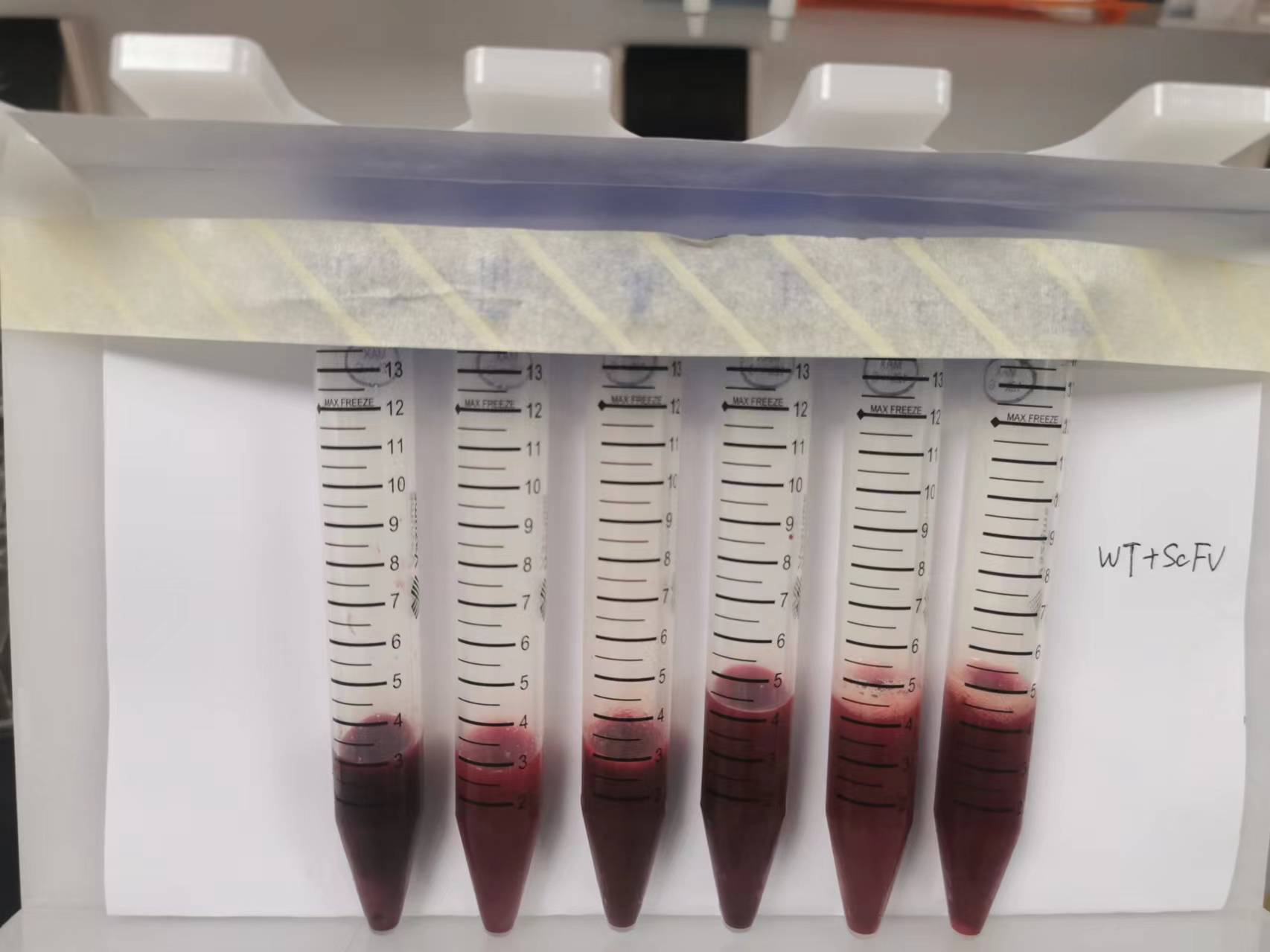

Supplement: Supplementary file 8 — Source data Fig. 6 [file 44321_2026_452_MOESM8_ESM.zip › Figure 6/6E-H/Figure 6E (Right) WT scFv.jpg]

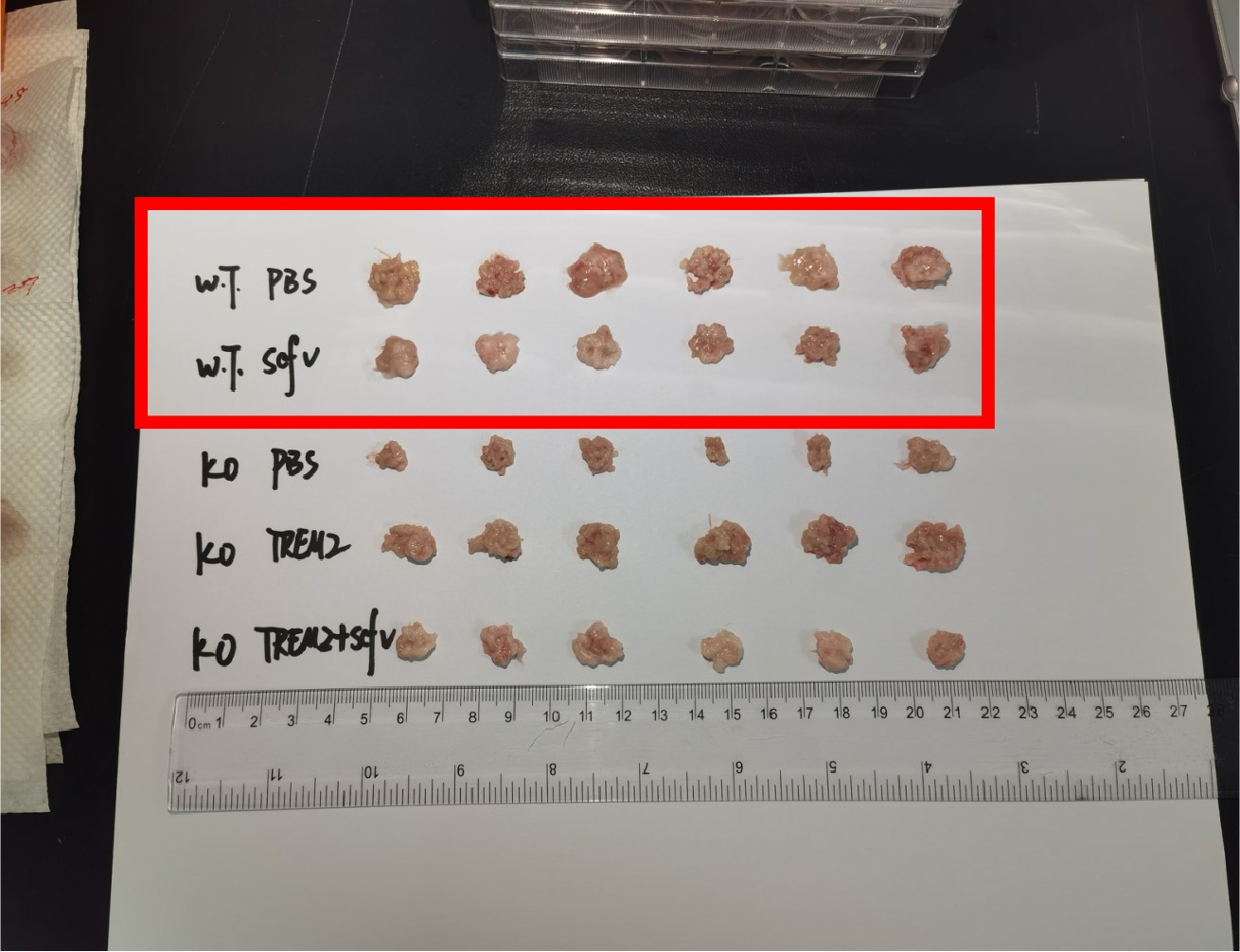

Supplement: Supplementary file 8 — Source data Fig. 6 [file 44321_2026_452_MOESM8_ESM.zip › Figure 6/6E-H/Figure 6E tumor burden.jpg]

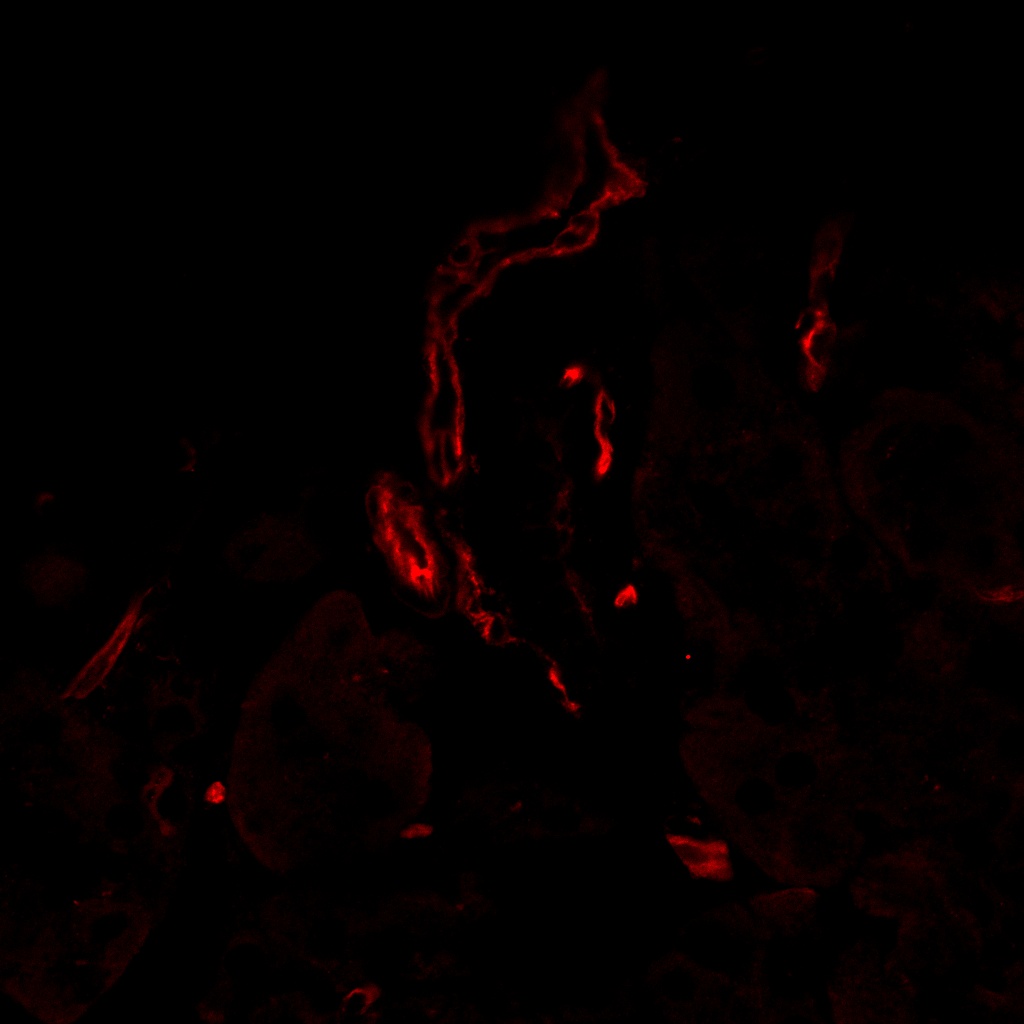

Supplement: Supplementary file 8 — Source data Fig. 6 [file 44321_2026_452_MOESM8_ESM.zip › Figure 6/6K-L/WT PBS CD31.jpg]

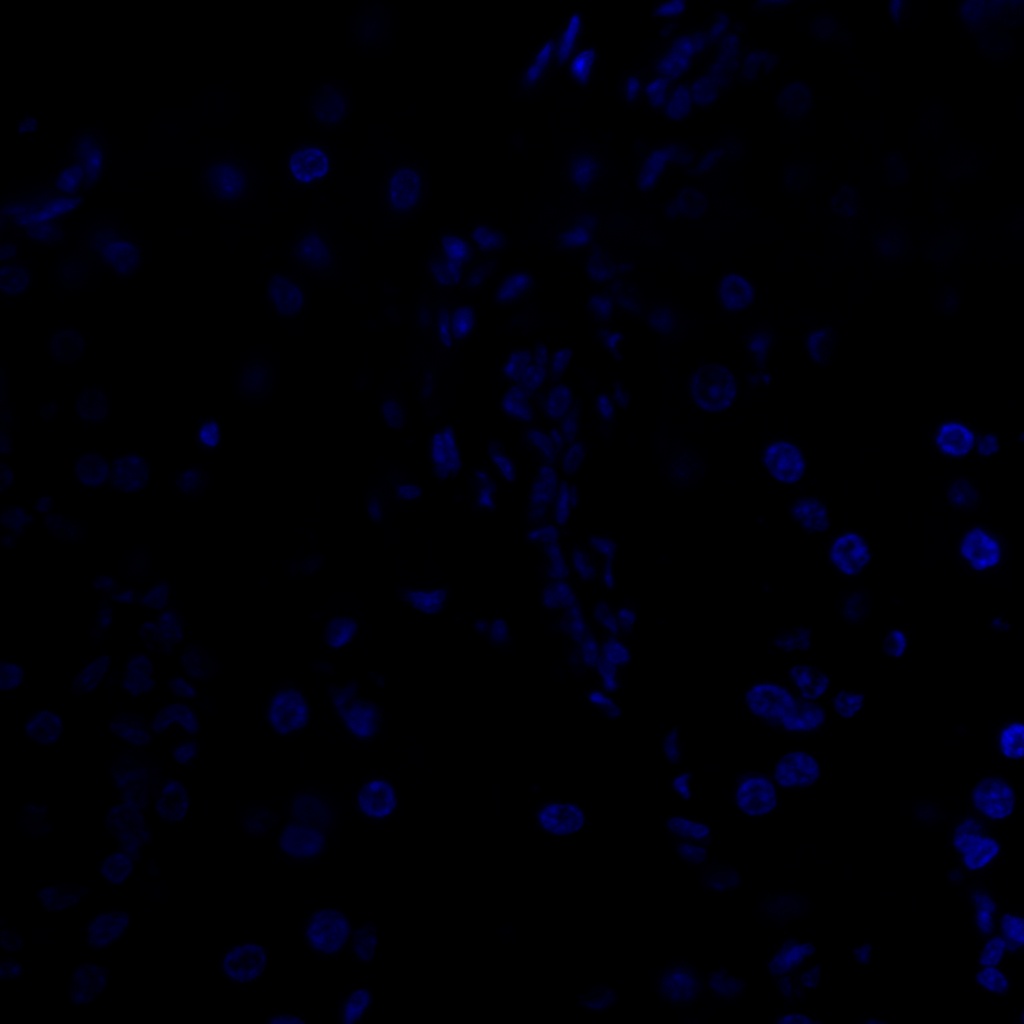

Supplement: Supplementary file 8 — Source data Fig. 6 [file 44321_2026_452_MOESM8_ESM.zip › Figure 6/6K-L/WT PBS dapi.jpg]

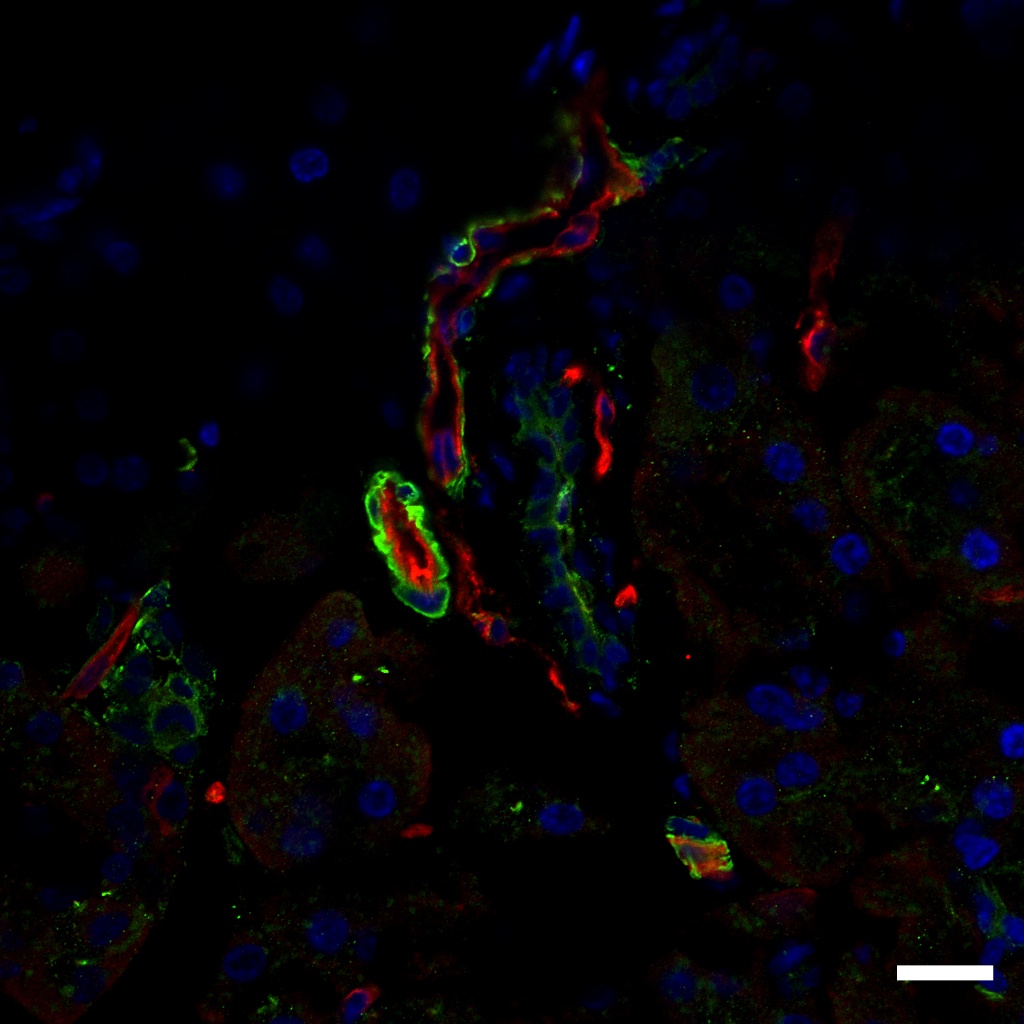

Supplement: Supplementary file 8 — Source data Fig. 6 [file 44321_2026_452_MOESM8_ESM.zip › Figure 6/6K-L/WT PBS Merge.jpg]

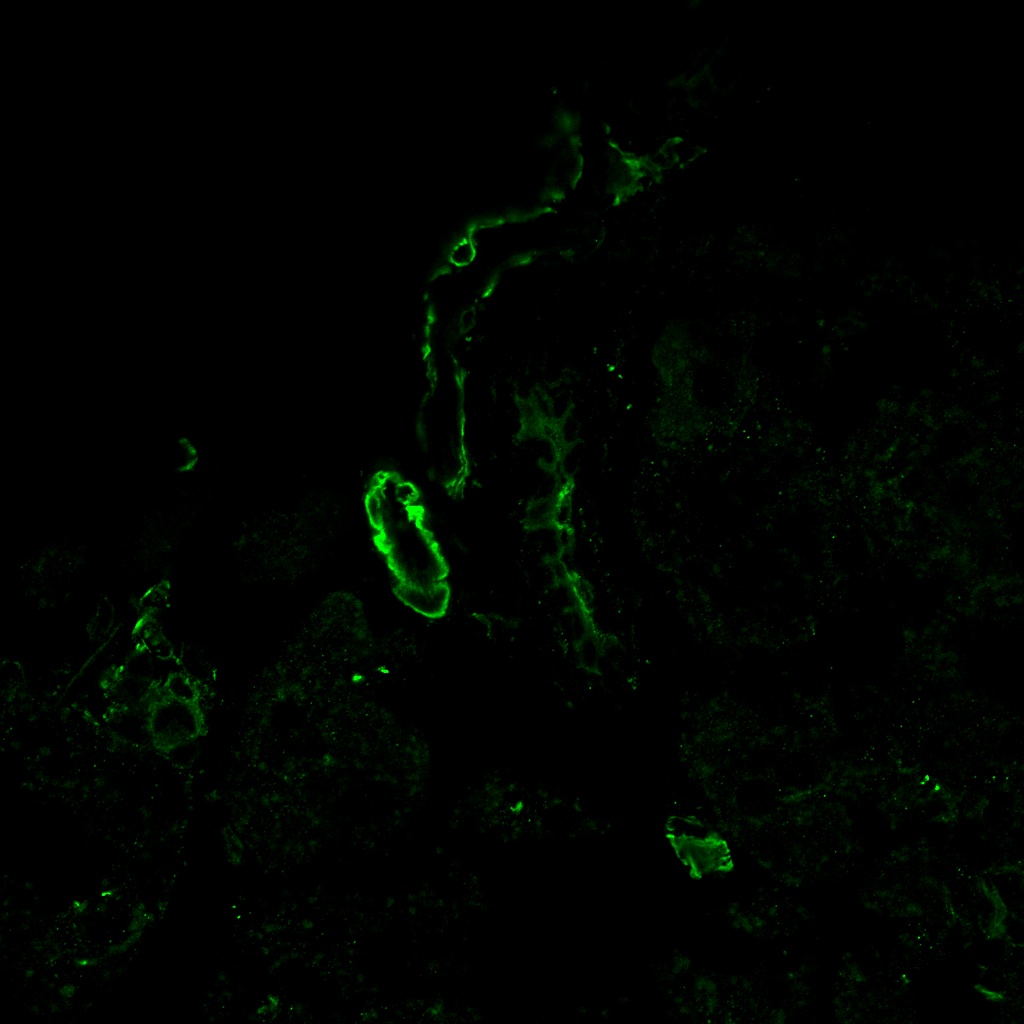

Supplement: Supplementary file 8 — Source data Fig. 6 [file 44321_2026_452_MOESM8_ESM.zip › Figure 6/6K-L/WT PBS p-VEcad.jpg]

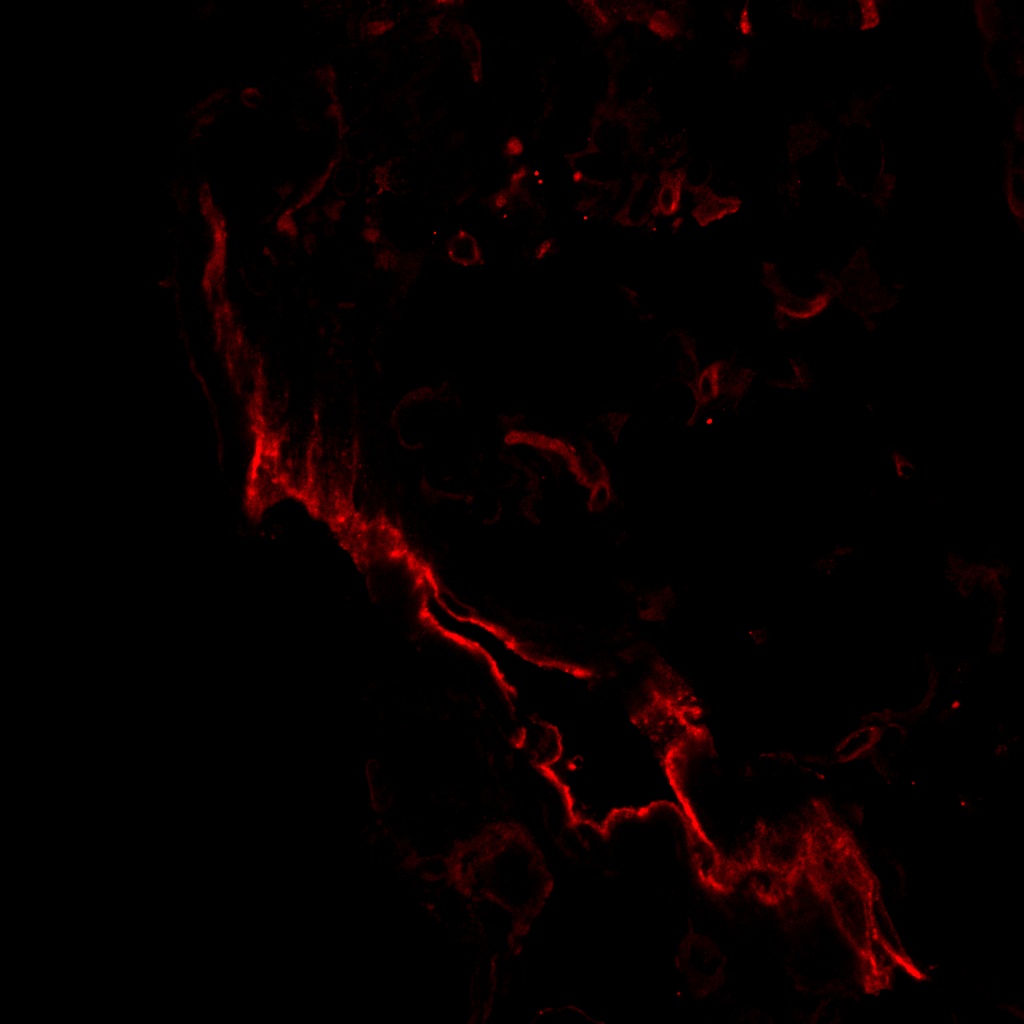

Supplement: Supplementary file 8 — Source data Fig. 6 [file 44321_2026_452_MOESM8_ESM.zip › Figure 6/6K-L/WT scFV CD31.jpg]

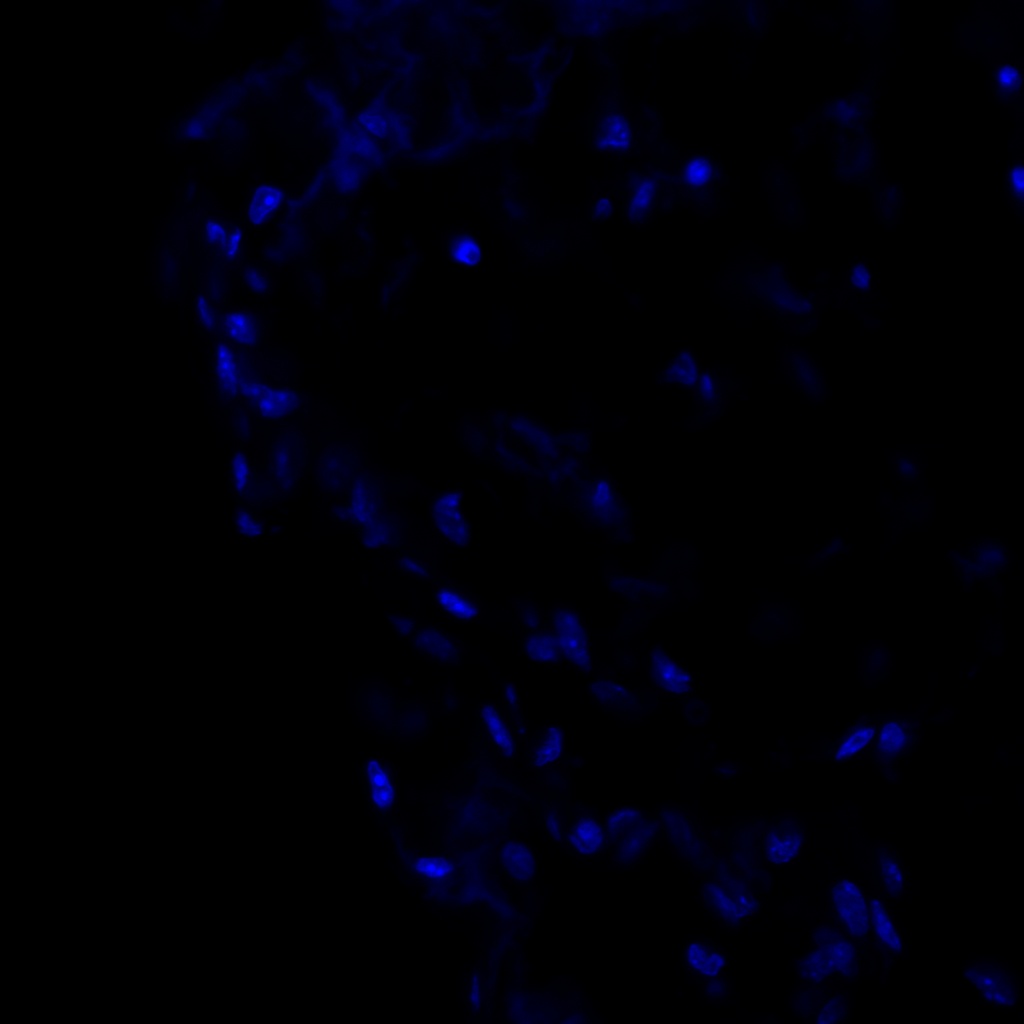

Supplement: Supplementary file 8 — Source data Fig. 6 [file 44321_2026_452_MOESM8_ESM.zip › Figure 6/6K-L/WT scFV dapi.jpg]

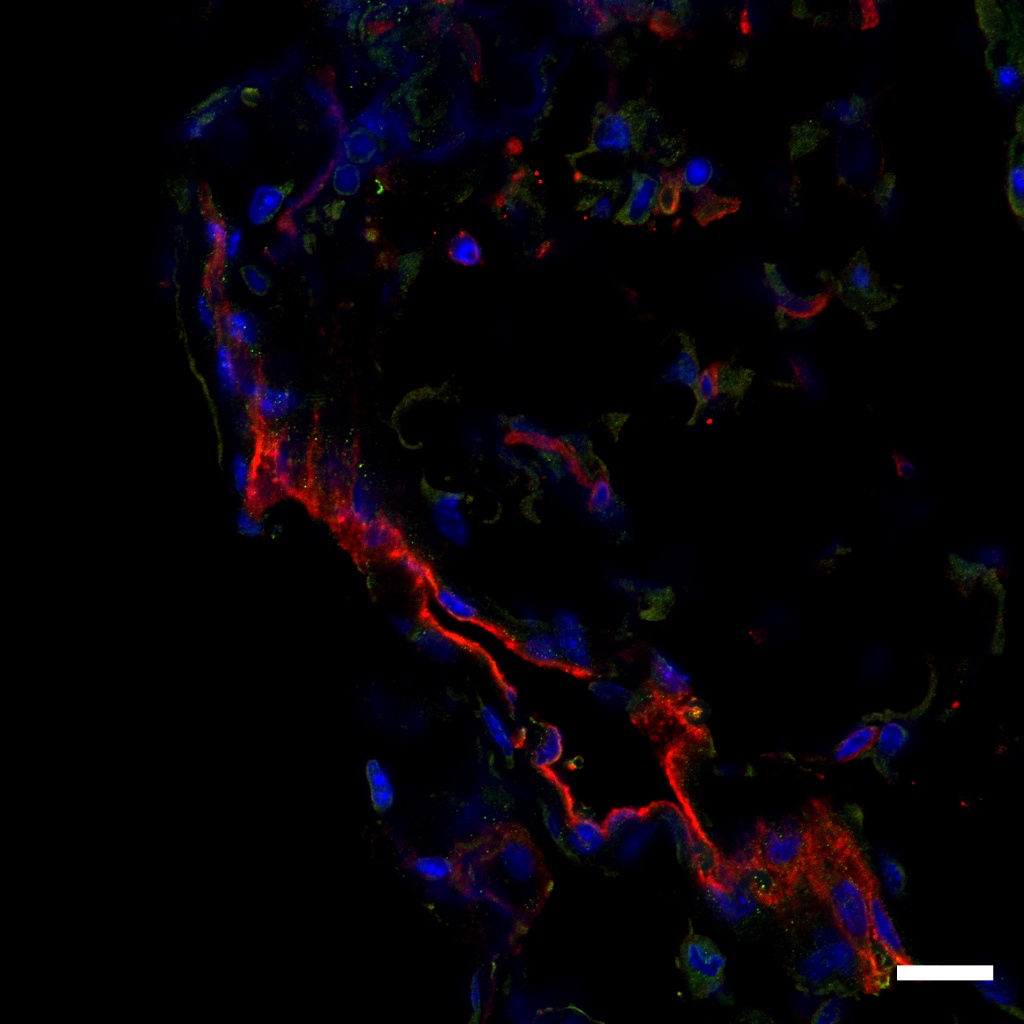

Supplement: Supplementary file 8 — Source data Fig. 6 [file 44321_2026_452_MOESM8_ESM.zip › Figure 6/6K-L/WT scFV Merge.jpg]

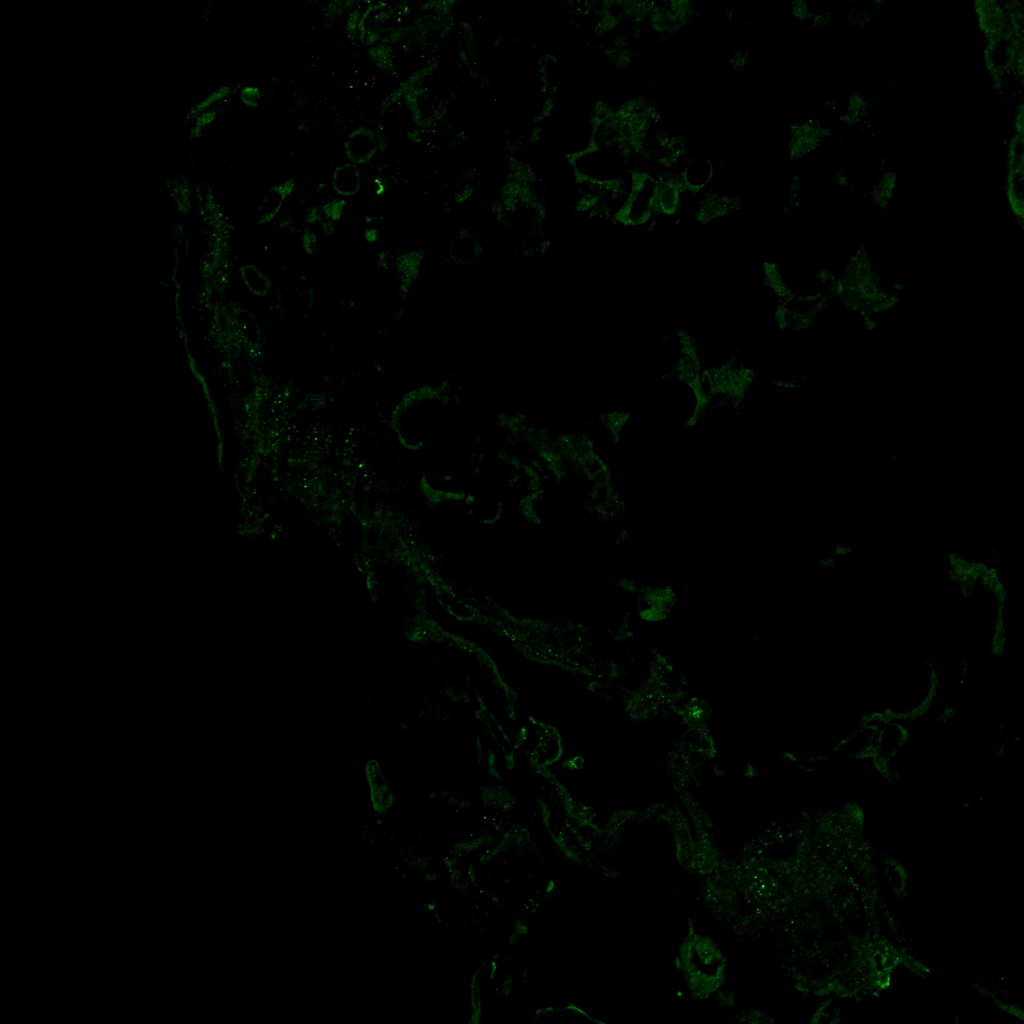

Supplement: Supplementary file 8 — Source data Fig. 6 [file 44321_2026_452_MOESM8_ESM.zip › Figure 6/6K-L/WT scFV p-VEcad.jpg]

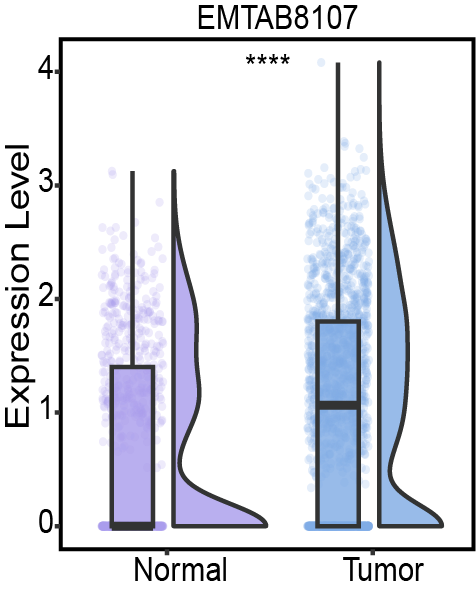

Supplement: Supplementary file 9 — Figure EV1 Source Data [file 44321_2026_452_MOESM9_ESM.zip › Figure EV1/EV1B/EMTAB8107_TREM2inMacro_violin_DiseaseV2 3.png]

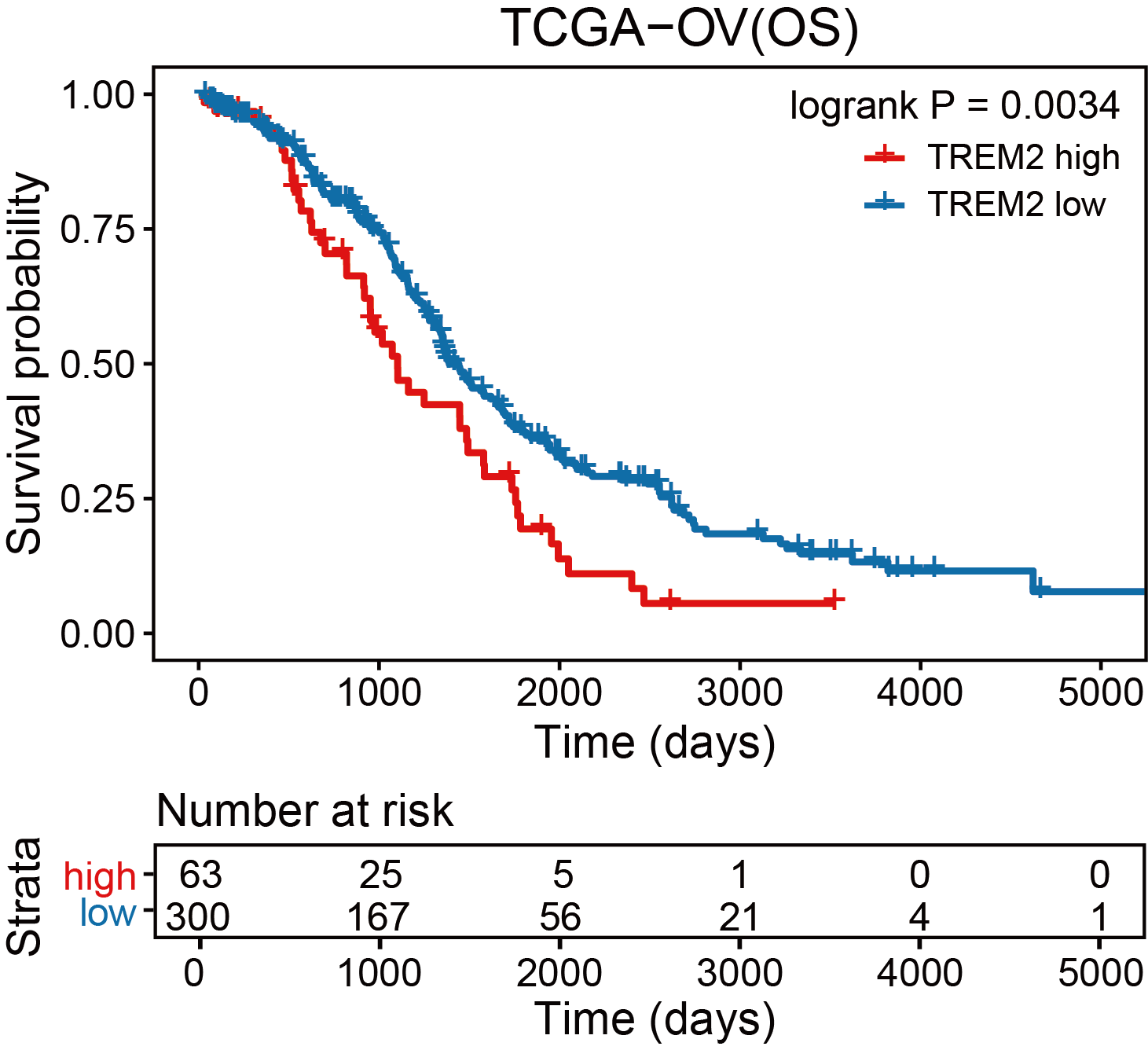

Supplement: Supplementary file 9 — Figure EV1 Source Data [file 44321_2026_452_MOESM9_ESM.zip › Figure EV1/EV1C-D/Figure EV1C_TCGA OS.png]

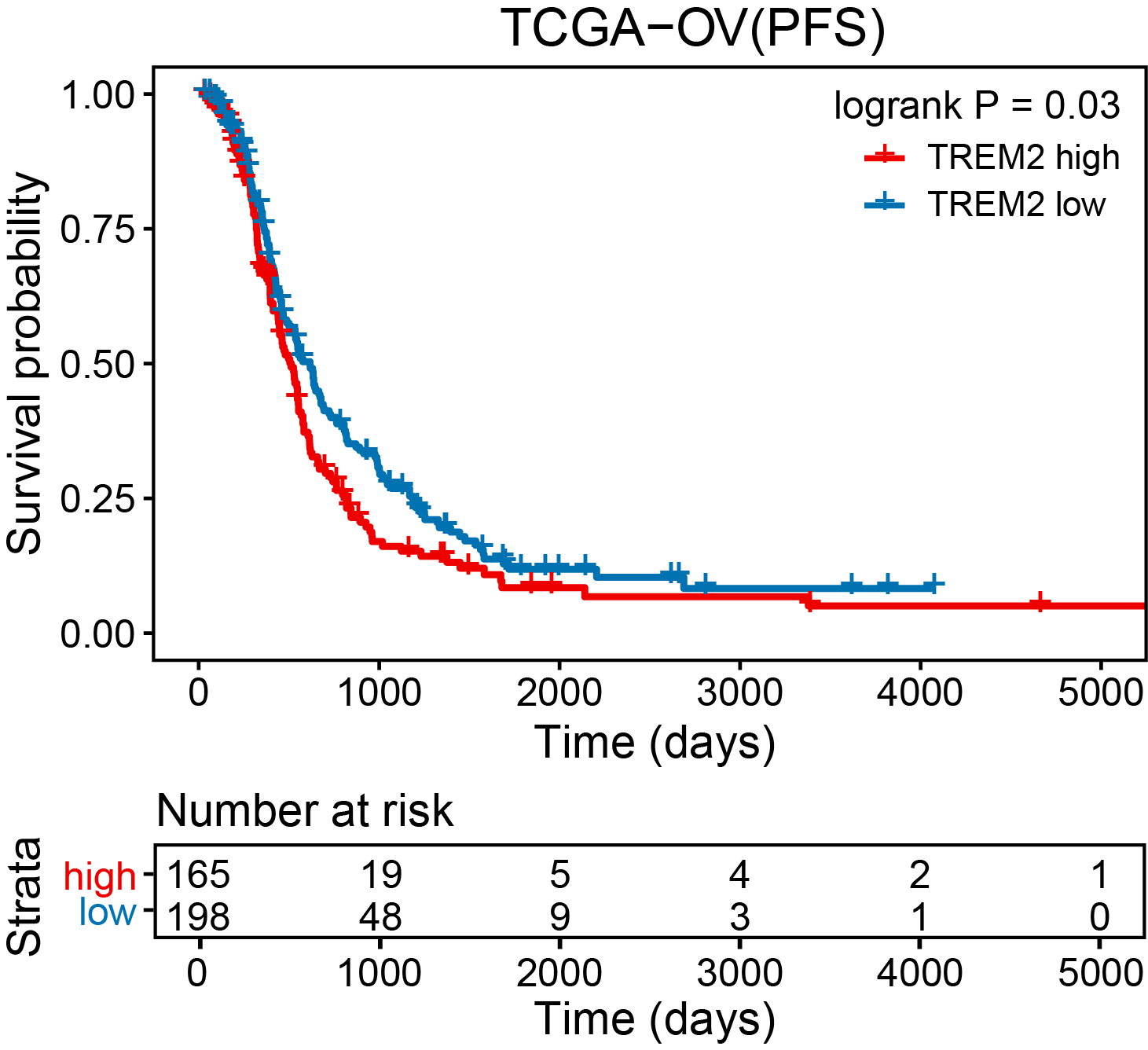

Supplement: Supplementary file 9 — Figure EV1 Source Data [file 44321_2026_452_MOESM9_ESM.zip › Figure EV1/EV1C-D/Figure EV1D_TCGA PFS.png]

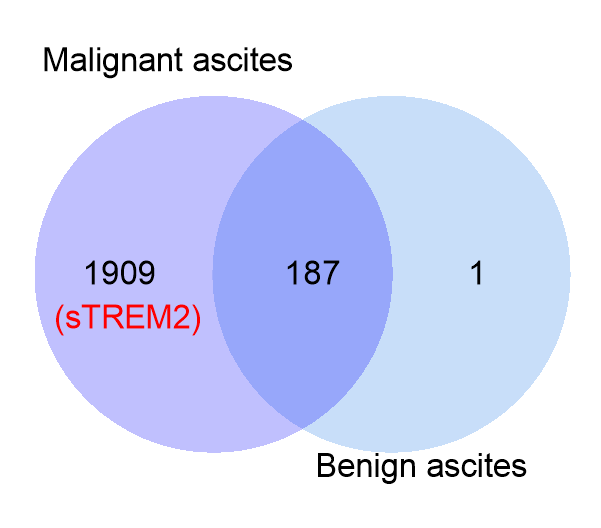

Supplement: Supplementary file 9 — Figure EV1 Source Data [file 44321_2026_452_MOESM9_ESM.zip › Figure EV1/EV1E/Figure EV1E.tif]

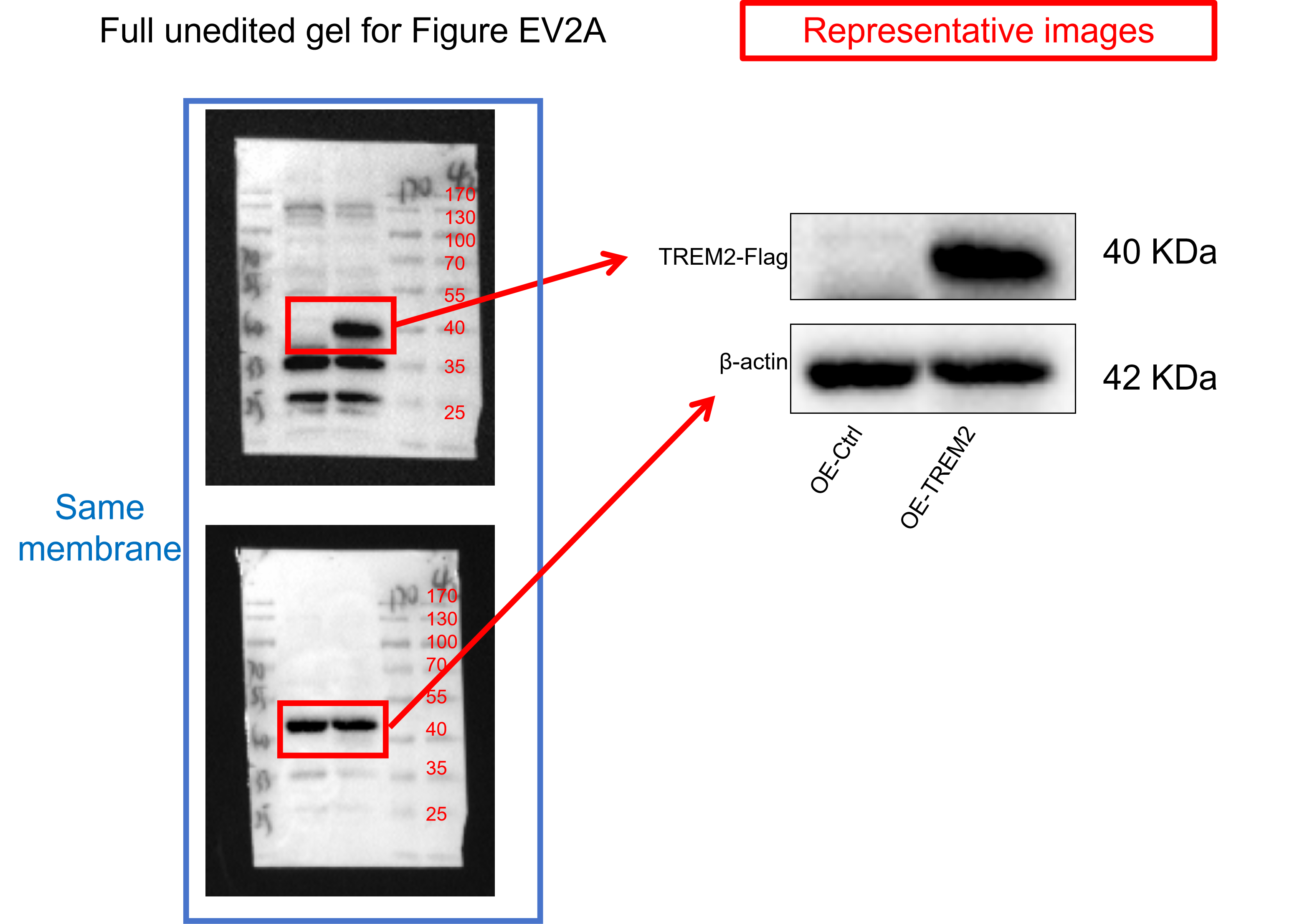

Supplement: Supplementary file 10 — Figure EV2 Source Data [file 44321_2026_452_MOESM10_ESM.zip › Figure EV2/EV2AB/Instructions for cropping Western blot images1.tif]

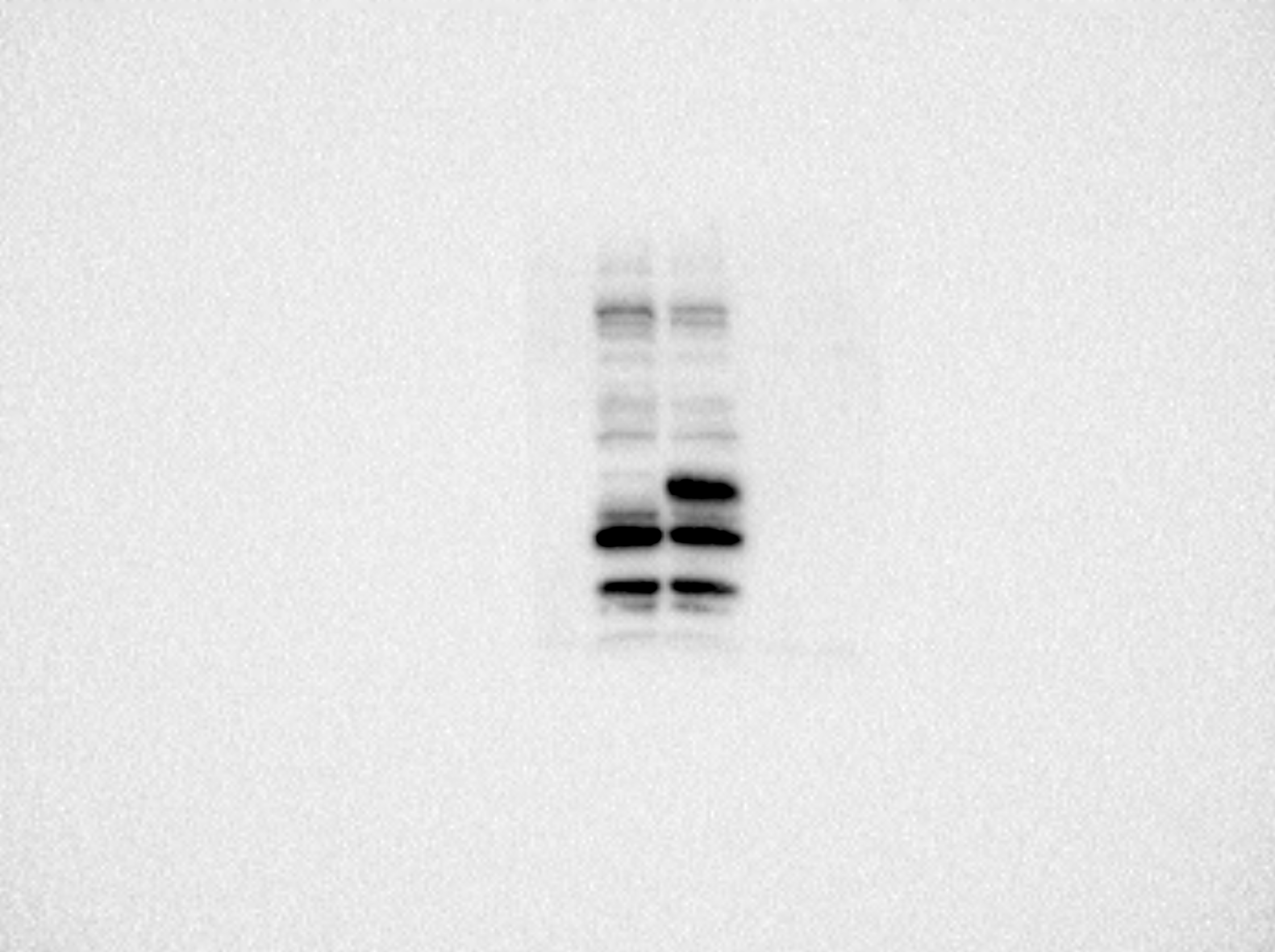

Supplement: Supplementary file 10 — Figure EV2 Source Data [file 44321_2026_452_MOESM10_ESM.zip › Figure EV2/EV2AB/WB_ Uncropped blots_ TREM2.tif]

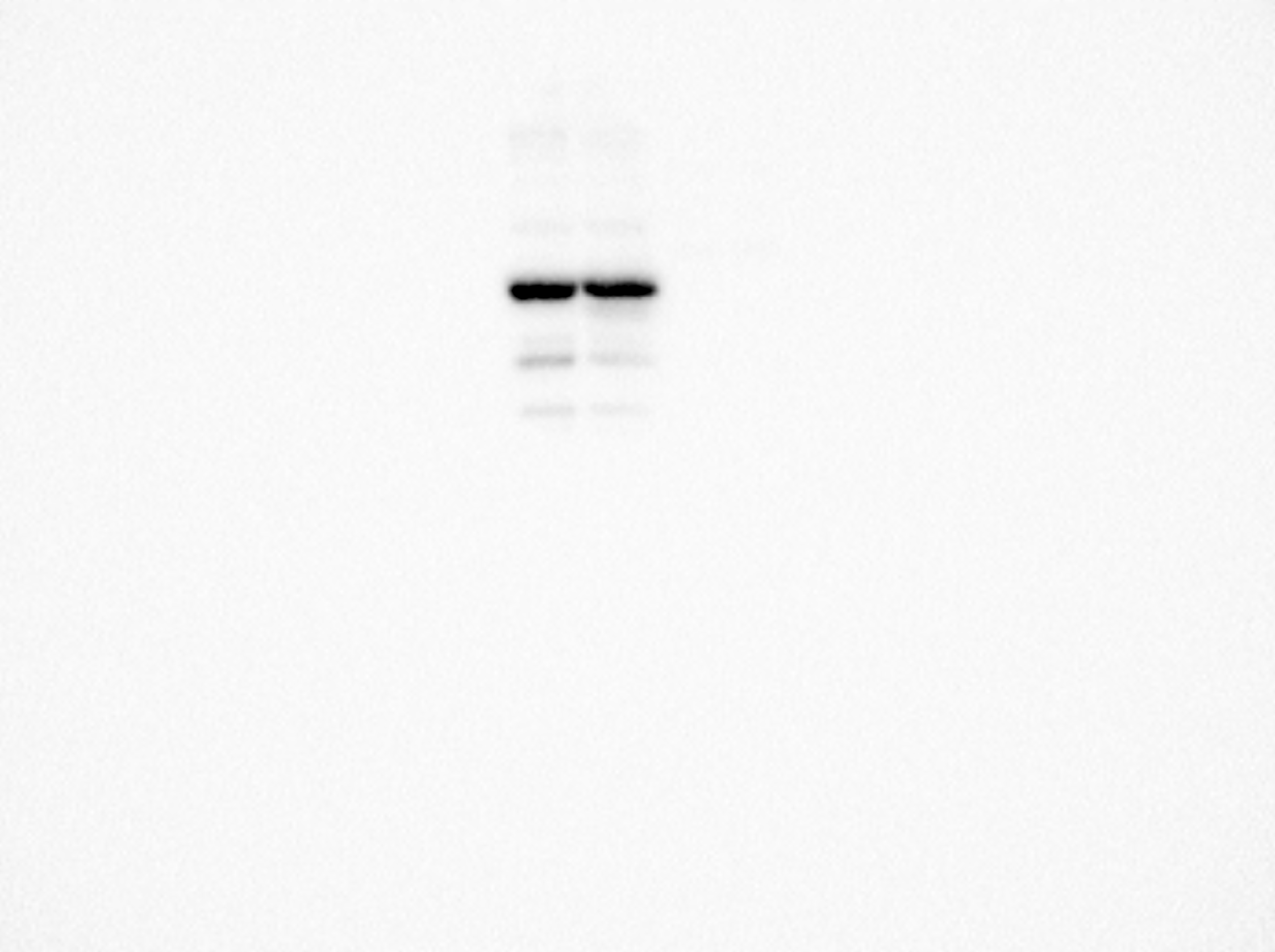

Supplement: Supplementary file 10 — Figure EV2 Source Data [file 44321_2026_452_MOESM10_ESM.zip › Figure EV2/EV2AB/WB_ Uncropped blots_ β-actin.tif]

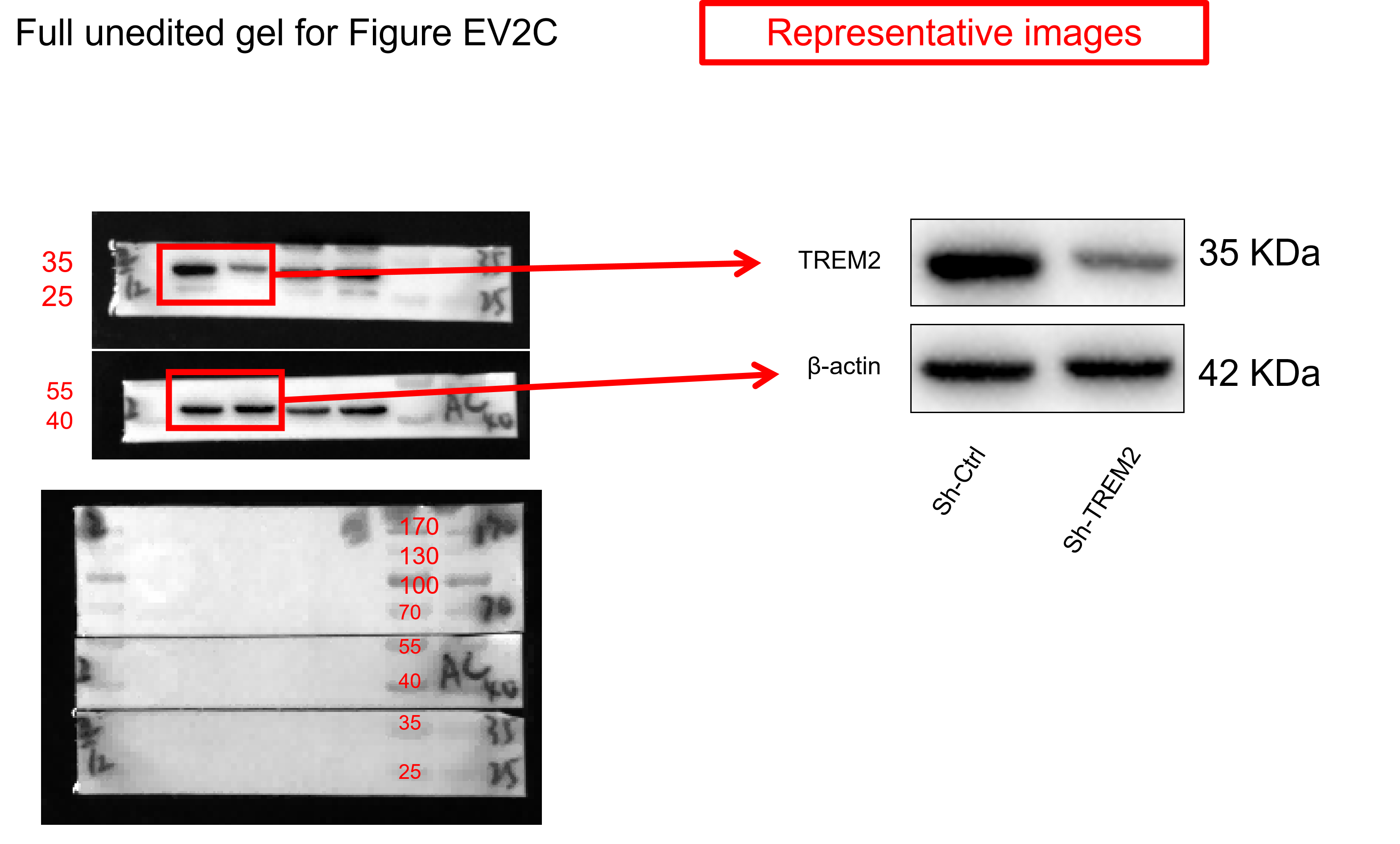

Supplement: Supplementary file 10 — Figure EV2 Source Data [file 44321_2026_452_MOESM10_ESM.zip › Figure EV2/EV2CD/Instructions for cropping Western blot images1.tif]

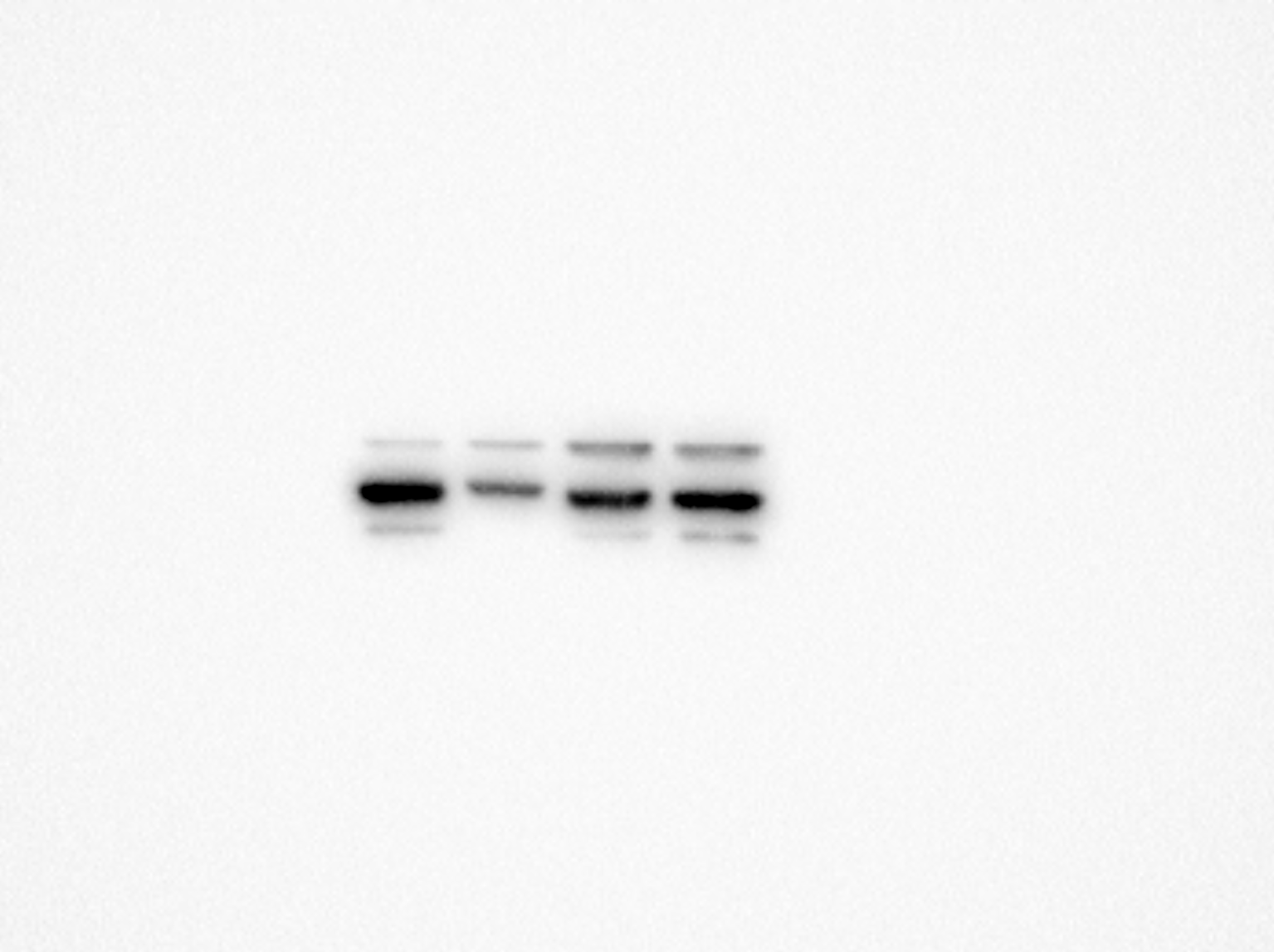

Supplement: Supplementary file 10 — Figure EV2 Source Data [file 44321_2026_452_MOESM10_ESM.zip › Figure EV2/EV2CD/WB_ Uncropped blots_ TREM2.tif]

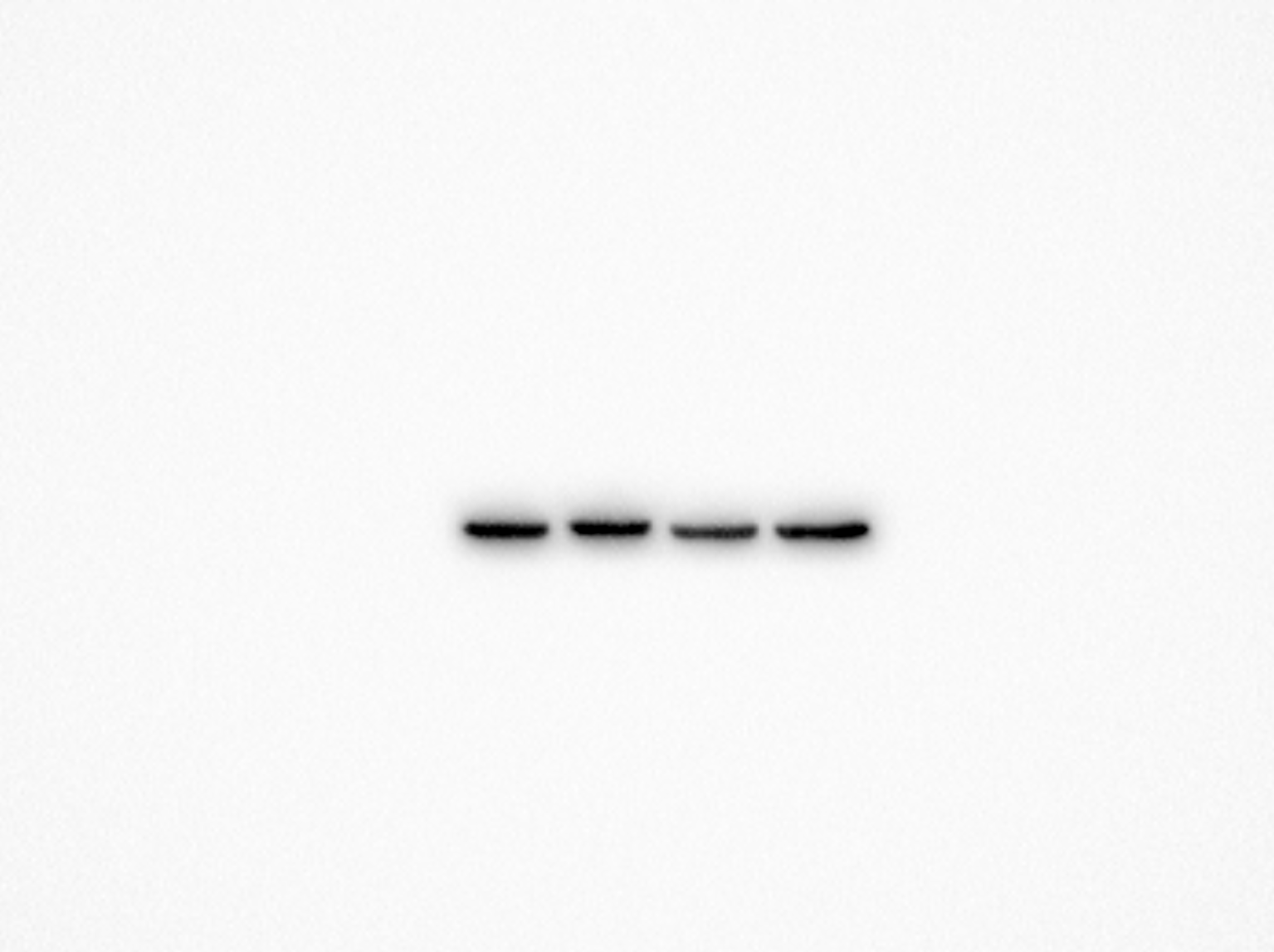

Supplement: Supplementary file 10 — Figure EV2 Source Data [file 44321_2026_452_MOESM10_ESM.zip › Figure EV2/EV2CD/WB_ Uncropped blots_ β-actin.tif]

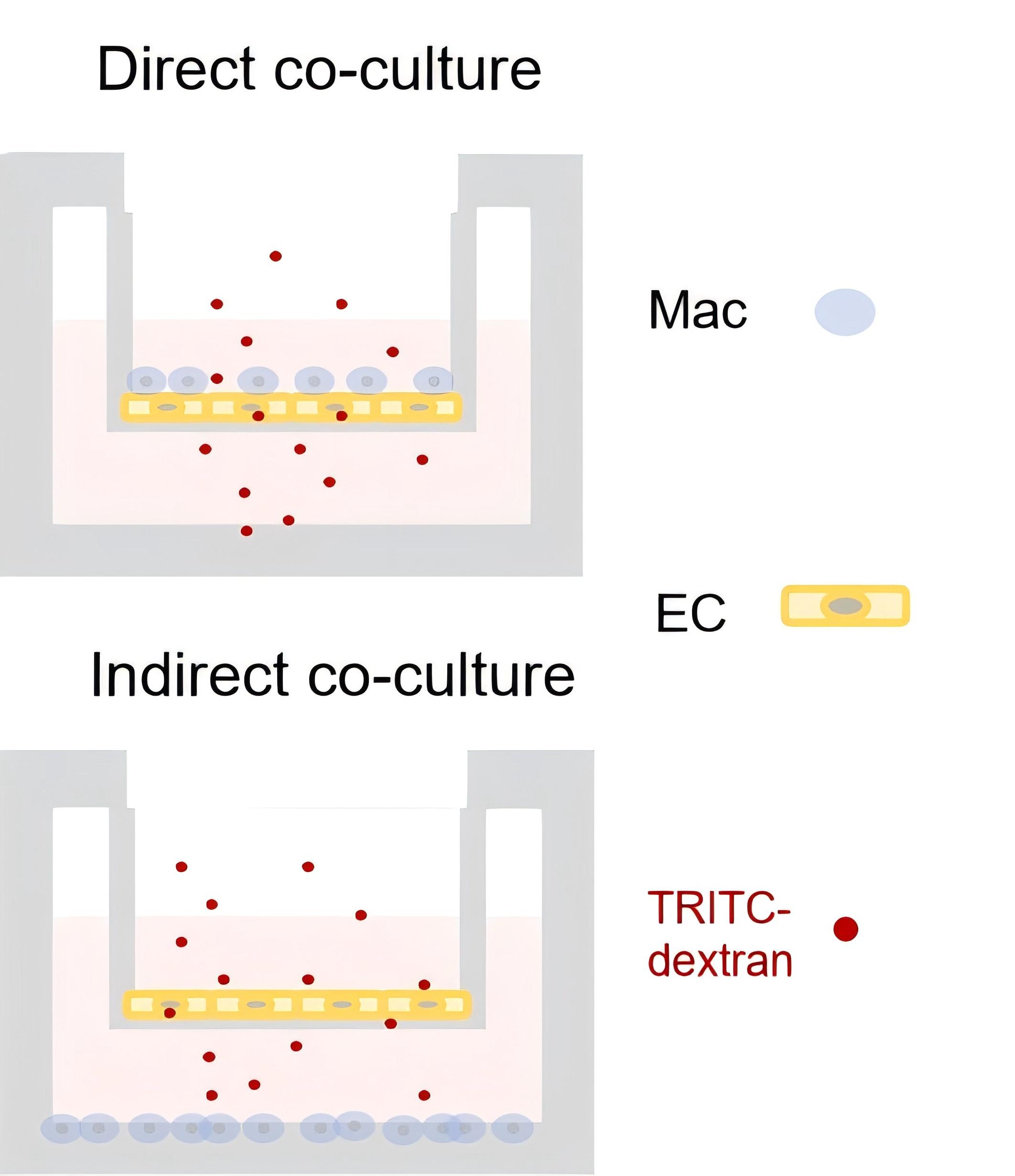

Supplement: Supplementary file 10 — Figure EV2 Source Data [file 44321_2026_452_MOESM10_ESM.zip › Figure EV2/EV2E/Figure EV2E.jpg]

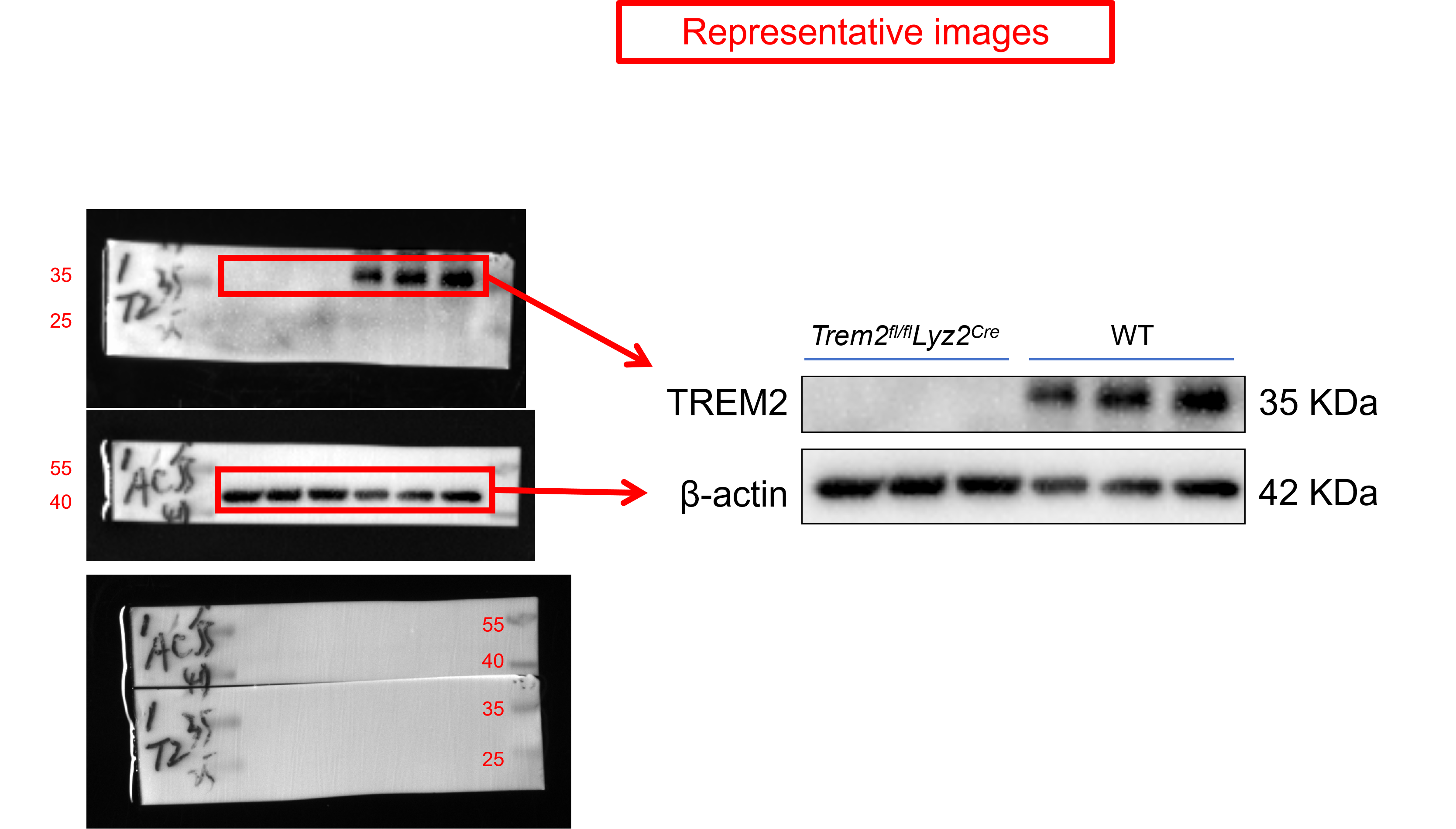

Supplement: Supplementary file 10 — Figure EV2 Source Data [file 44321_2026_452_MOESM10_ESM.zip › Figure EV2/EV2J/Instructions for cropping Western blot images 1.tif]

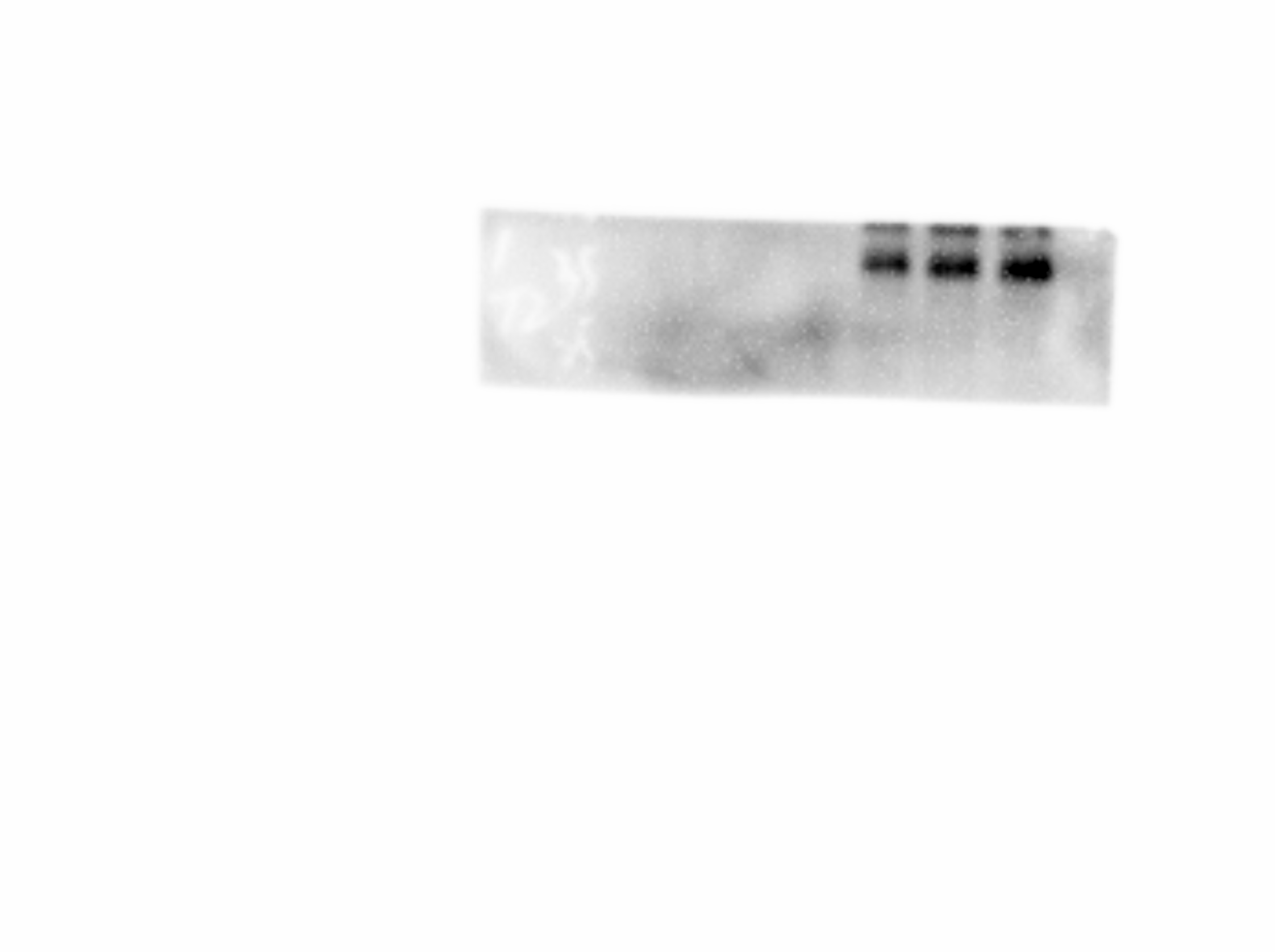

Supplement: Supplementary file 10 — Figure EV2 Source Data [file 44321_2026_452_MOESM10_ESM.zip › Figure EV2/EV2J/WB_ Uncropped blots_ TREM2.tif]

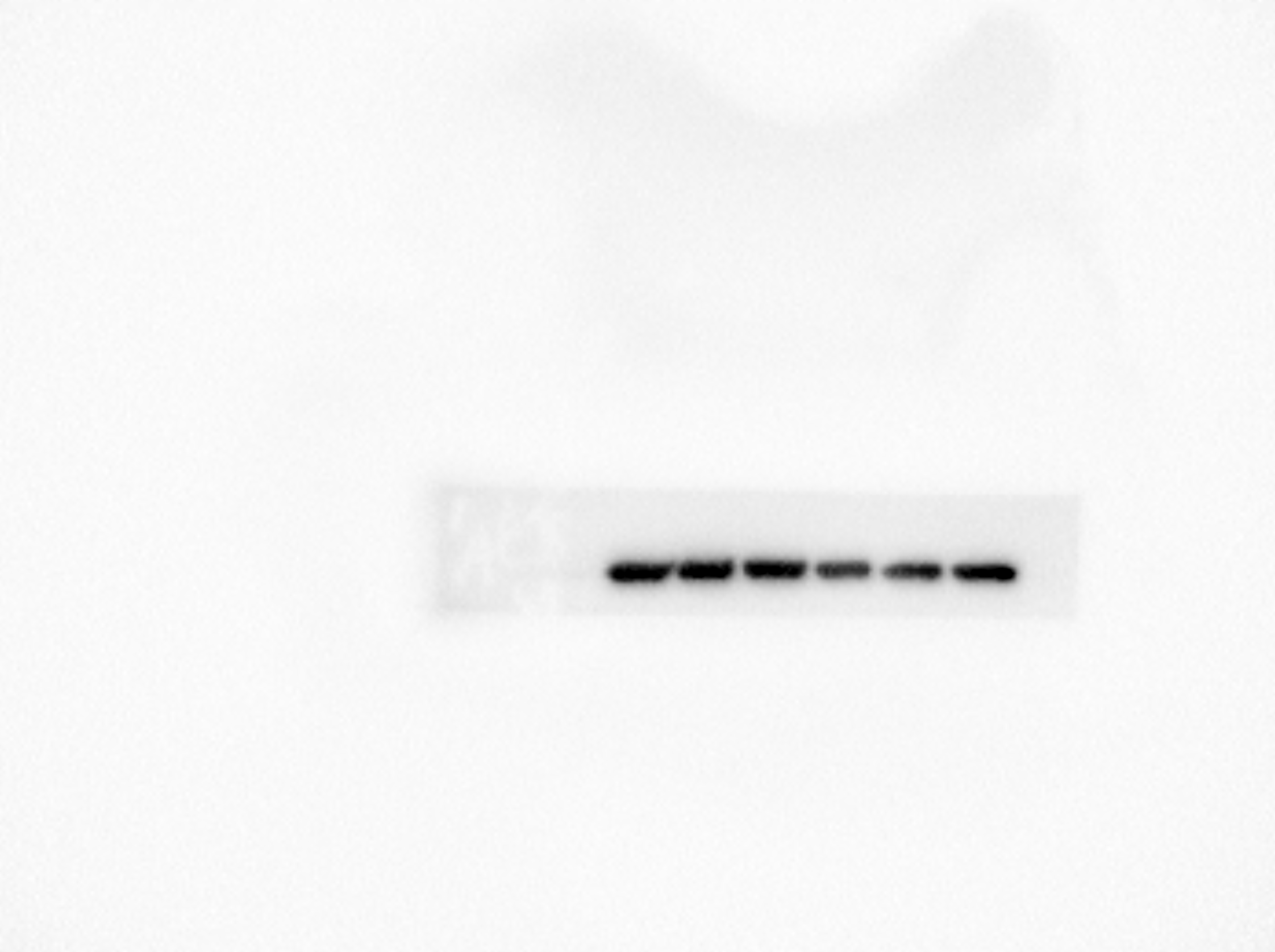

Supplement: Supplementary file 10 — Figure EV2 Source Data [file 44321_2026_452_MOESM10_ESM.zip › Figure EV2/EV2J/WB_ Uncropped blots_ β-actin.tif]

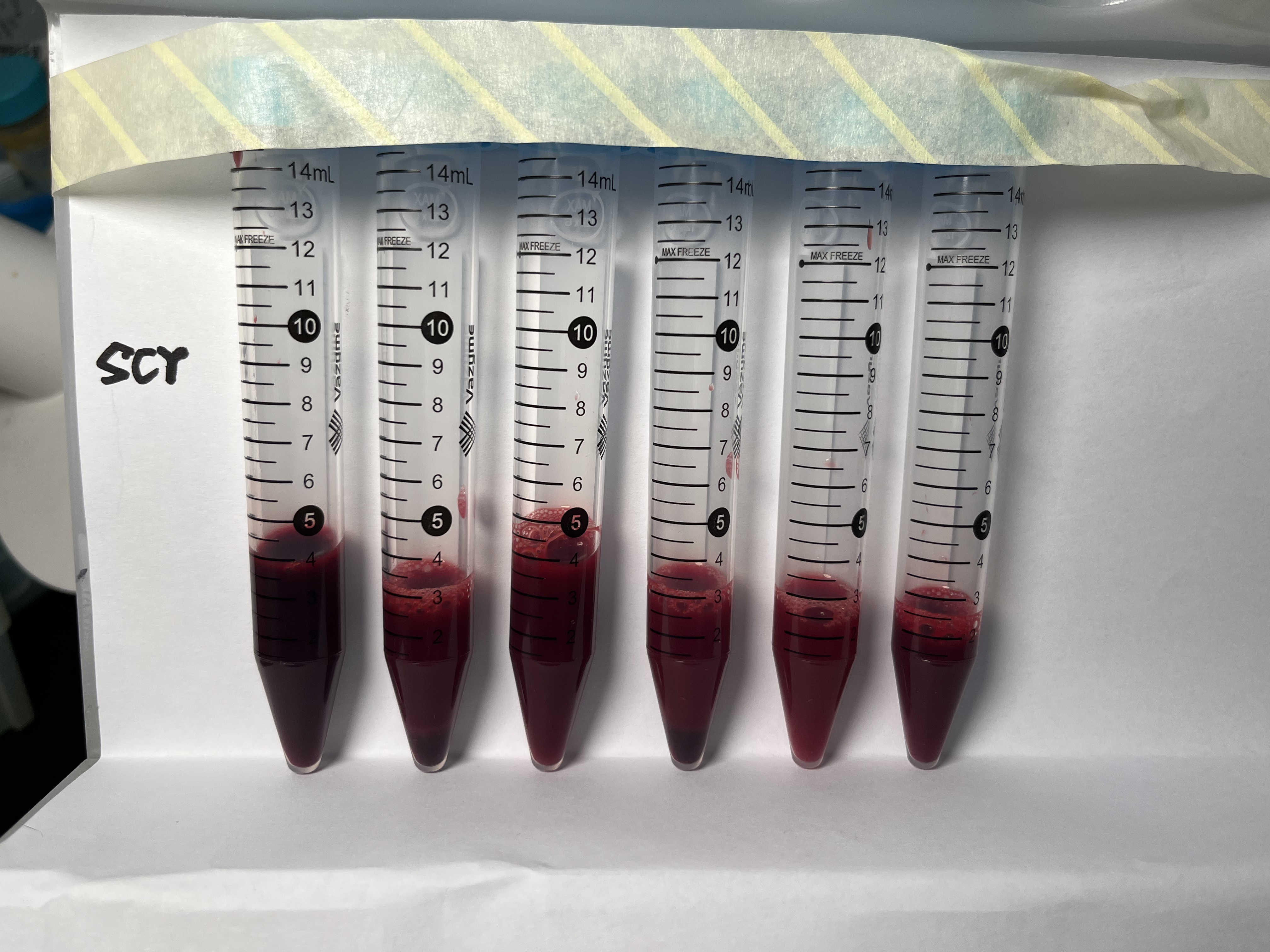

Supplement: Supplementary file 11 — Figure EV3 Source Data [file 44321_2026_452_MOESM11_ESM.zip › Figure EV3/EV3A-D/Figure EV3A(left) Sh-Ctrl.jpg]

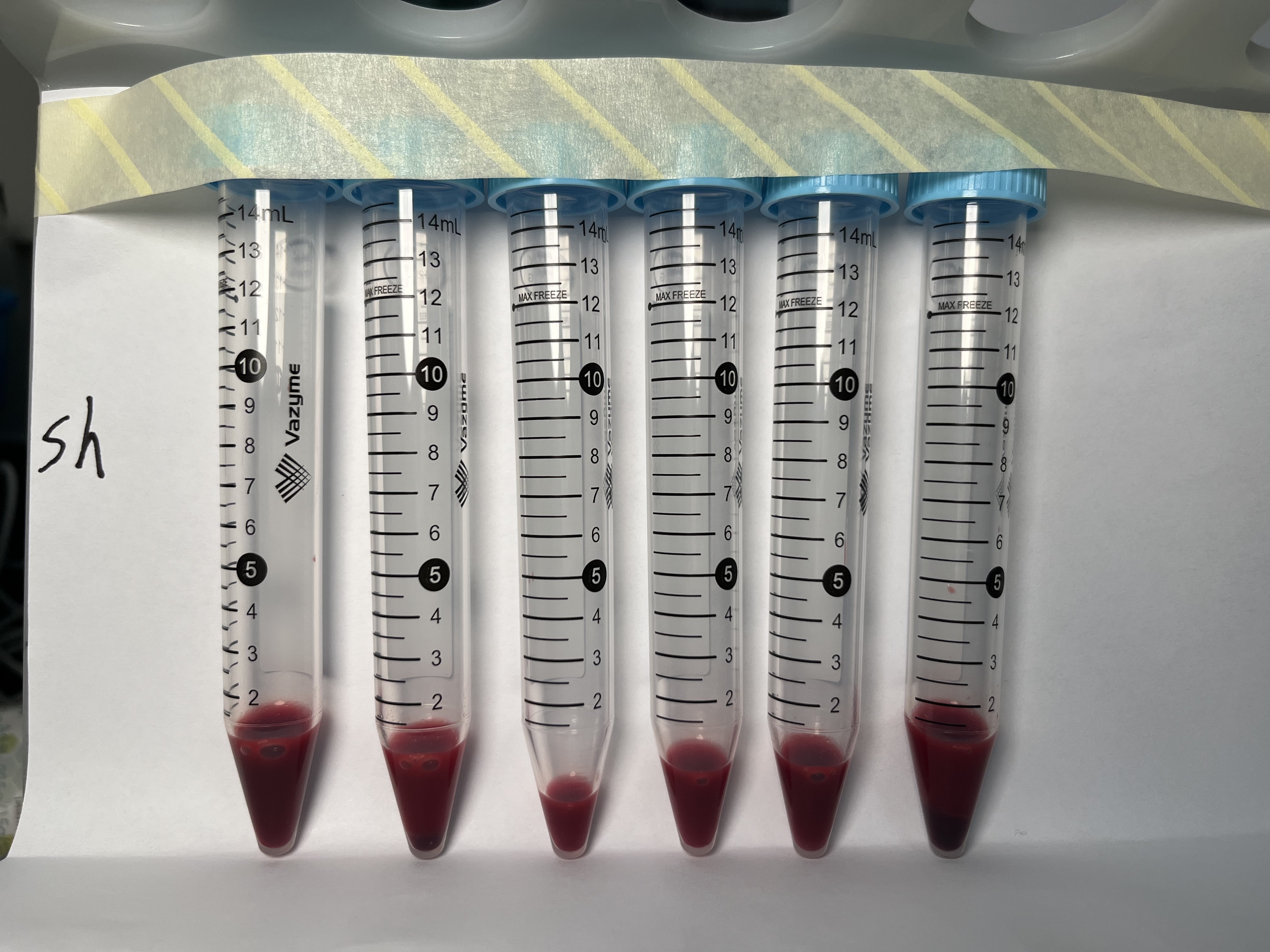

Supplement: Supplementary file 11 — Figure EV3 Source Data [file 44321_2026_452_MOESM11_ESM.zip › Figure EV3/EV3A-D/Figure EV3A(right) Sh-TREM2.jpg]

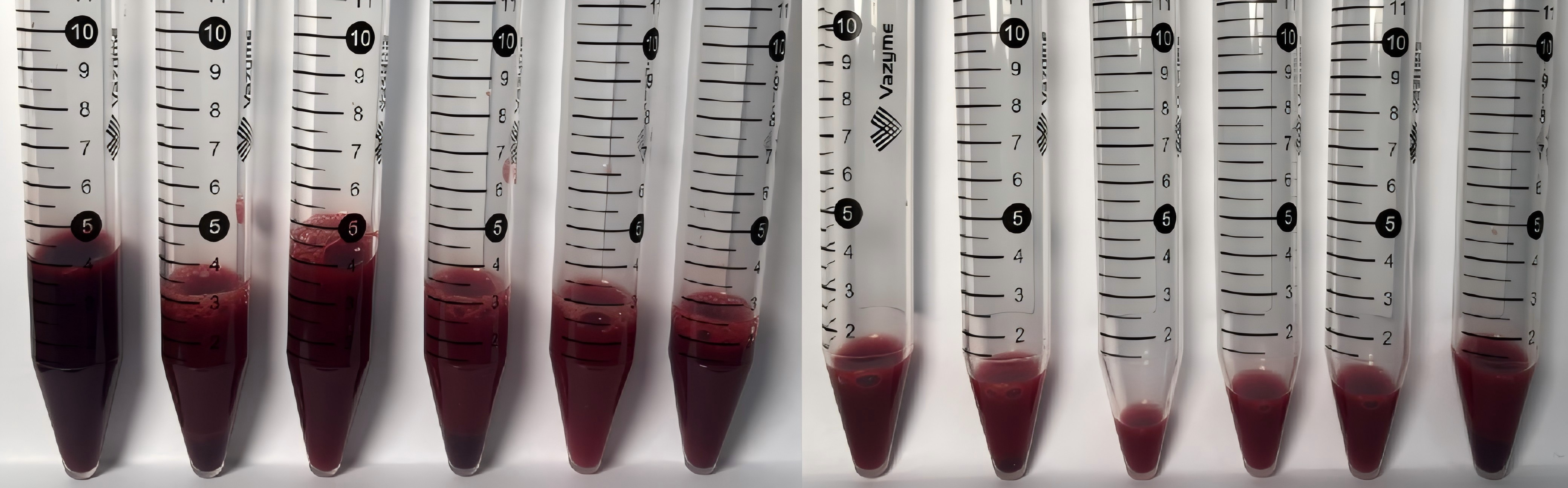

Supplement: Supplementary file 11 — Figure EV3 Source Data [file 44321_2026_452_MOESM11_ESM.zip › Figure EV3/EV3A-D/Figure EV3A.tif]

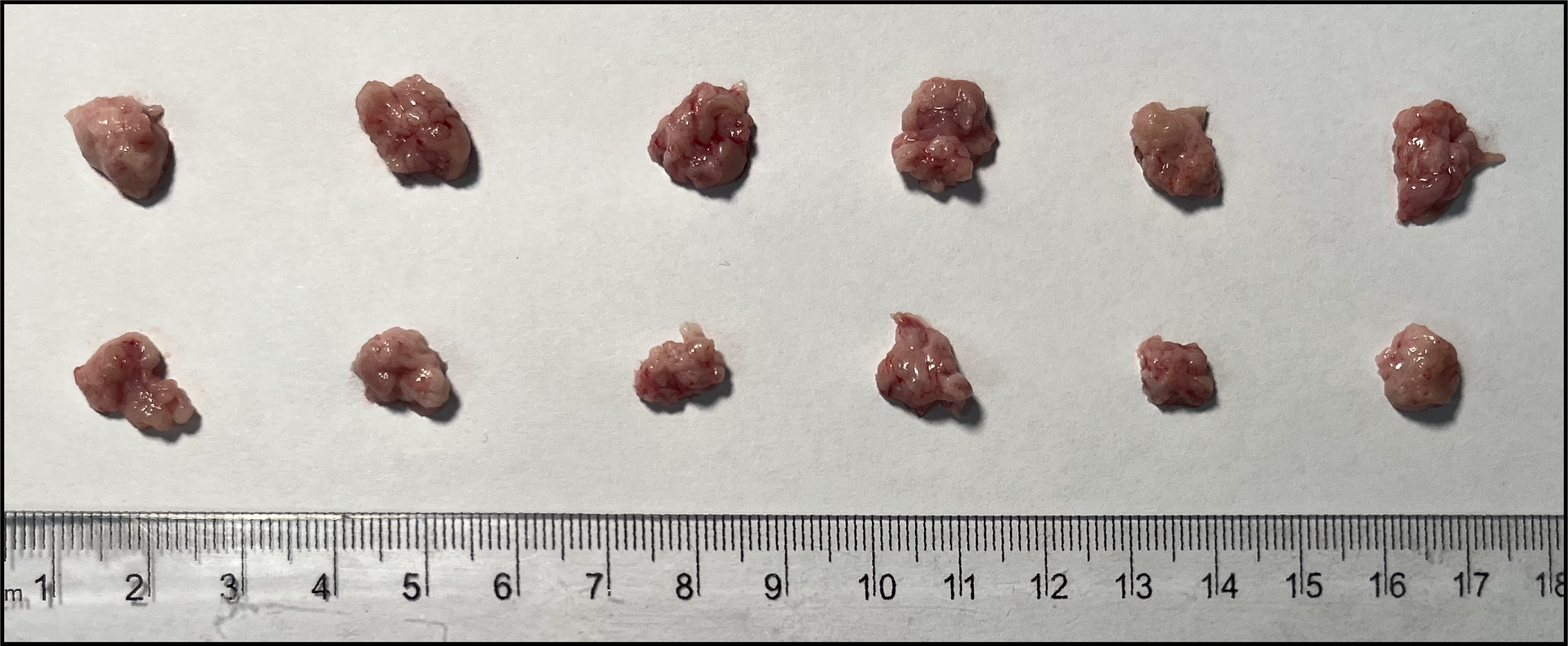

Supplement: Supplementary file 11 — Figure EV3 Source Data [file 44321_2026_452_MOESM11_ESM.zip › Figure EV3/EV3A-D/Figure EV3B tumor burden 2.tif]

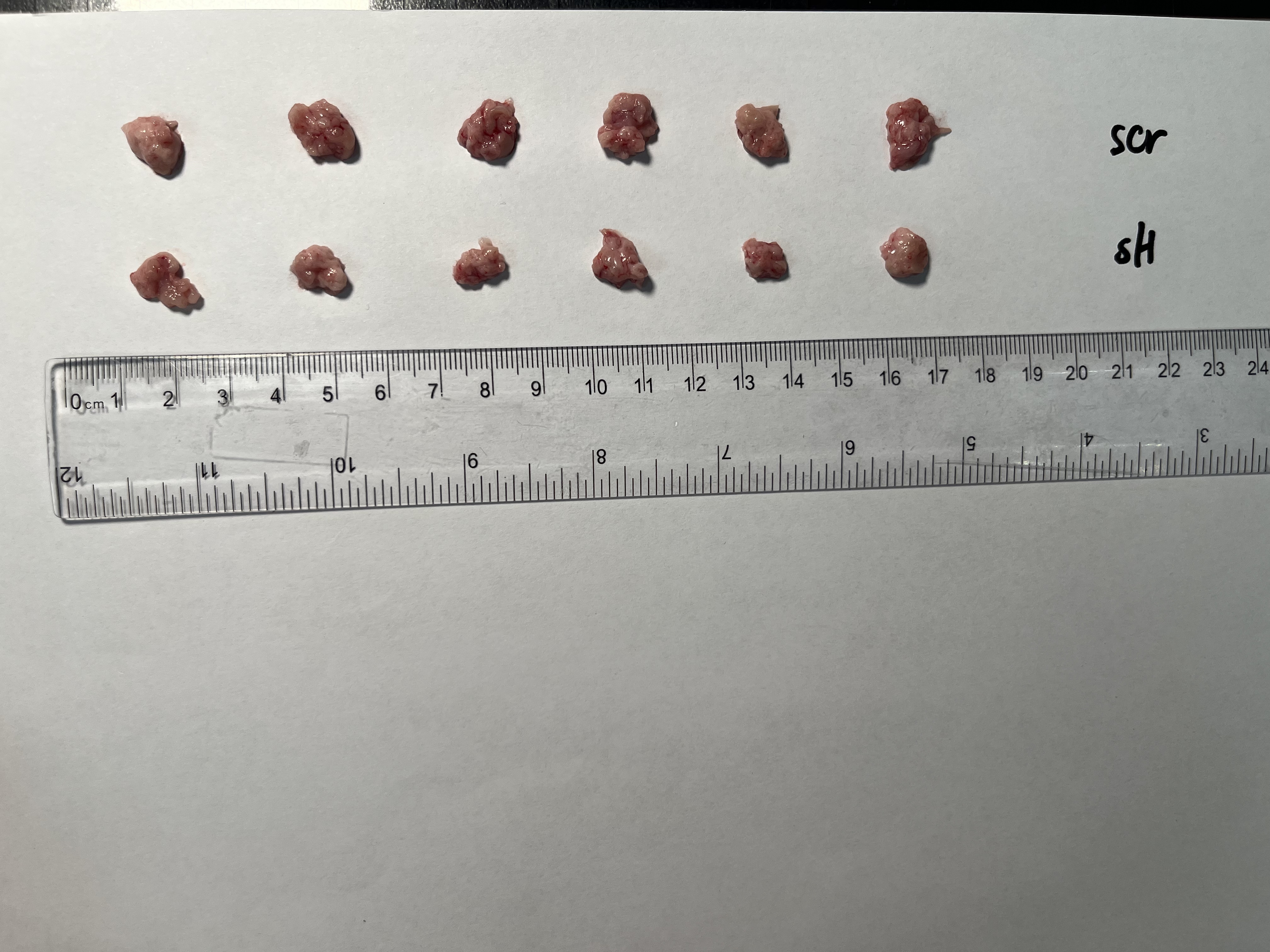

Supplement: Supplementary file 11 — Figure EV3 Source Data [file 44321_2026_452_MOESM11_ESM.zip › Figure EV3/EV3A-D/Figure EV3B tumor burden.jpg]

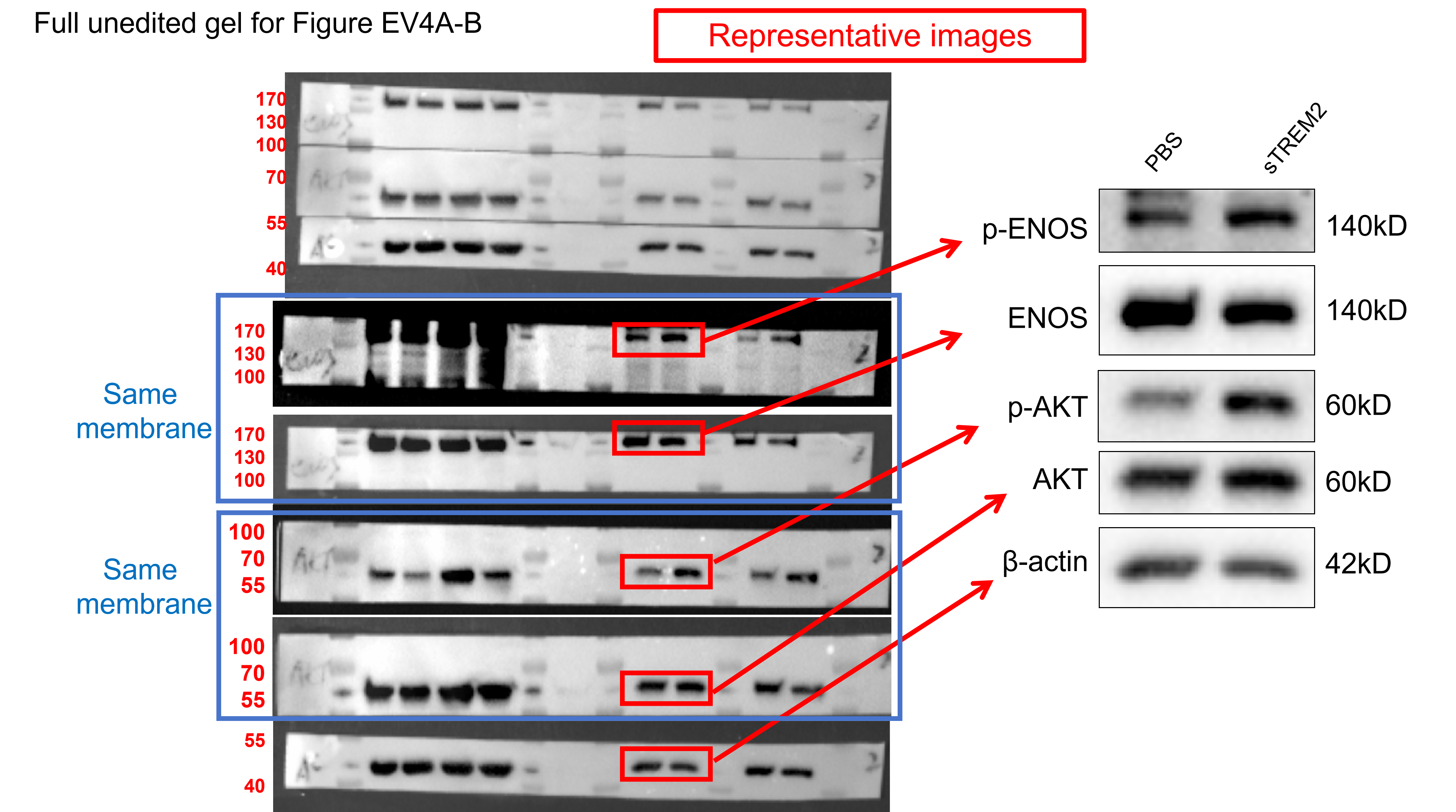

Supplement: Supplementary file 12 — Figure EV4 Source Data [file 44321_2026_452_MOESM12_ESM.zip › Figure EV4/EV4A-B/Instructions for cropping Western blot images.tif]

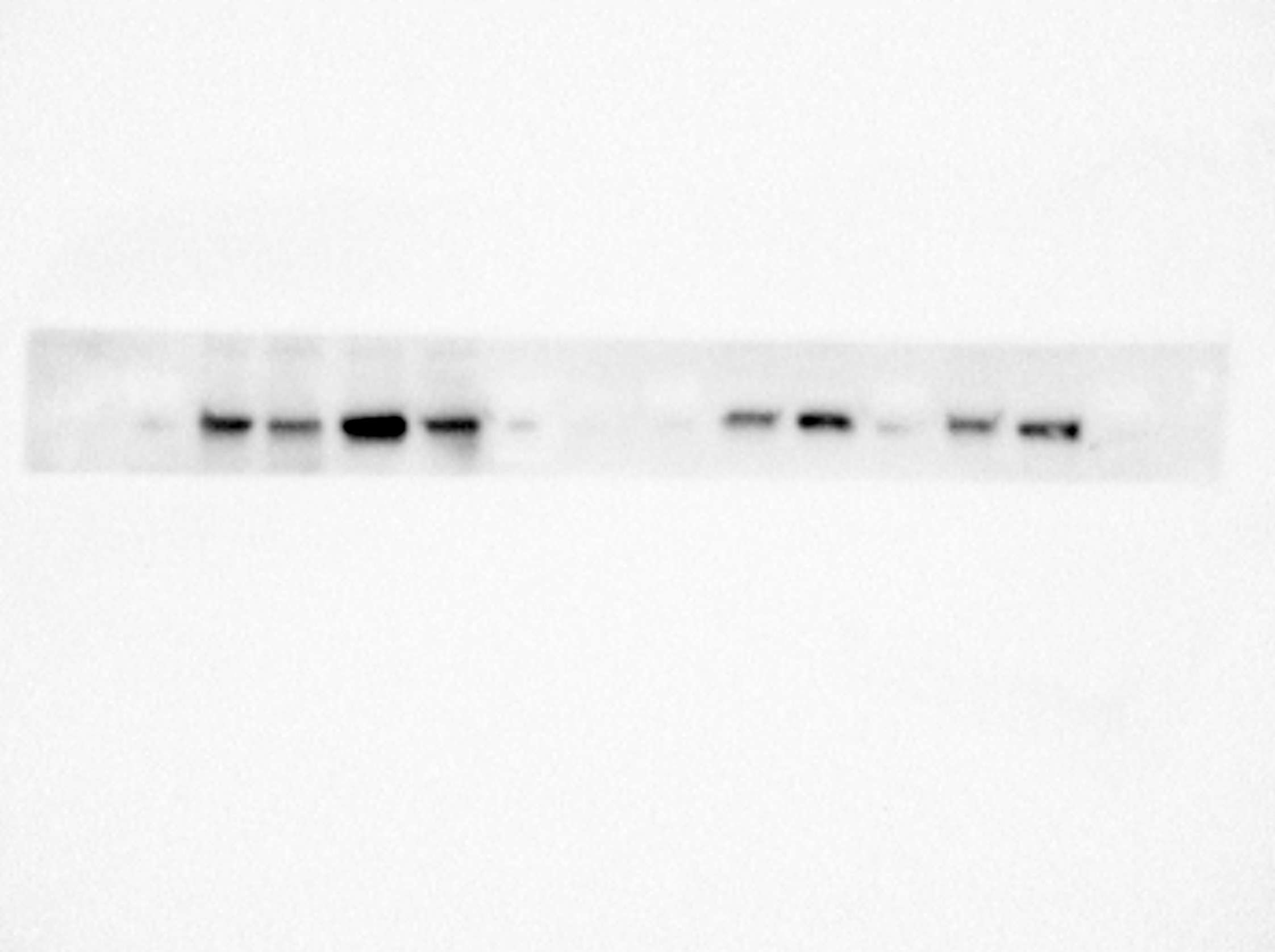

Supplement: Supplementary file 12 — Figure EV4 Source Data [file 44321_2026_452_MOESM12_ESM.zip › Figure EV4/EV4A-B/WB_ Uncropped blots_ p-AKT.tif]

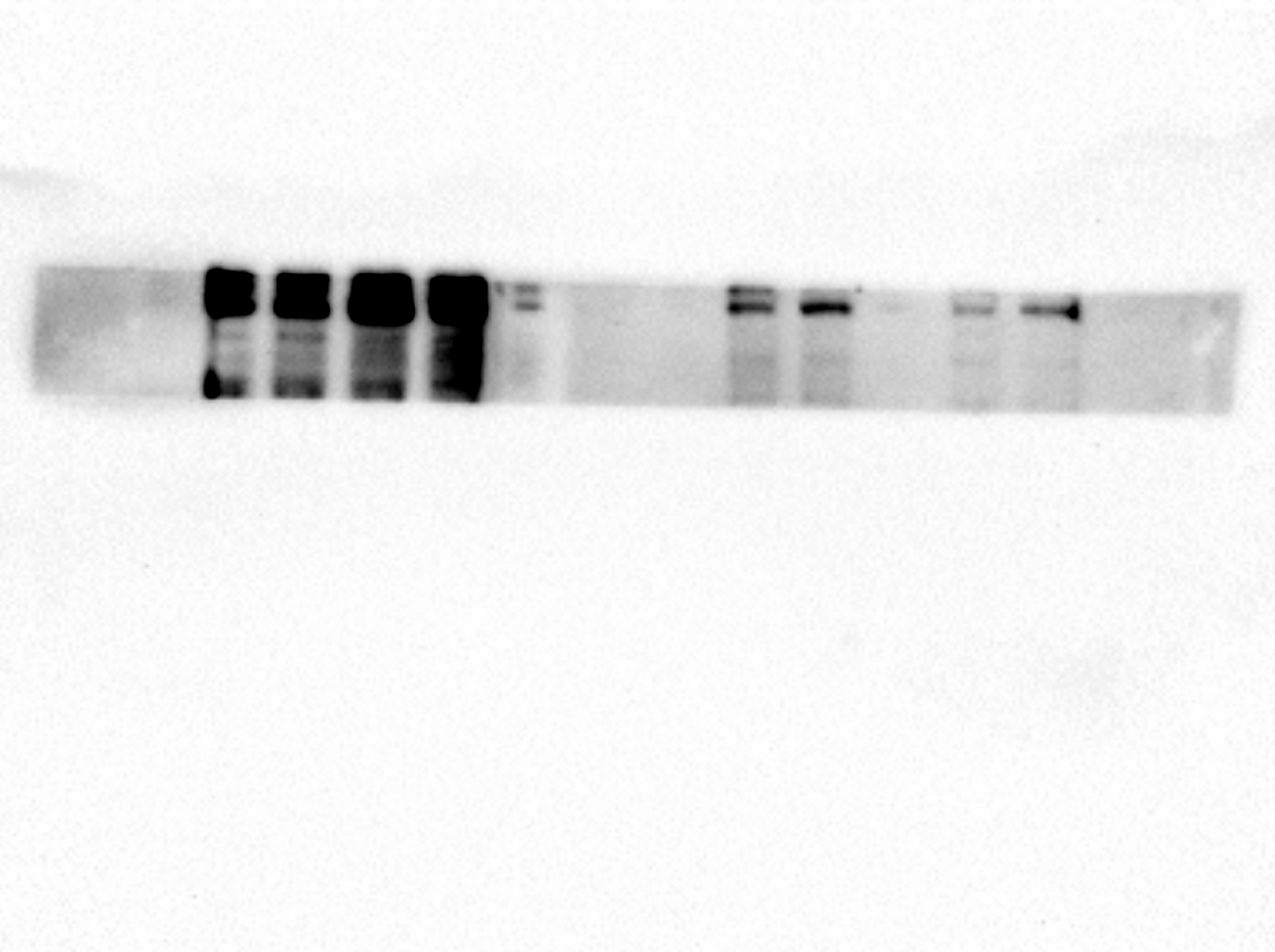

Supplement: Supplementary file 12 — Figure EV4 Source Data [file 44321_2026_452_MOESM12_ESM.zip › Figure EV4/EV4A-B/WB_ Uncropped blots_ p-eNOS.tif]

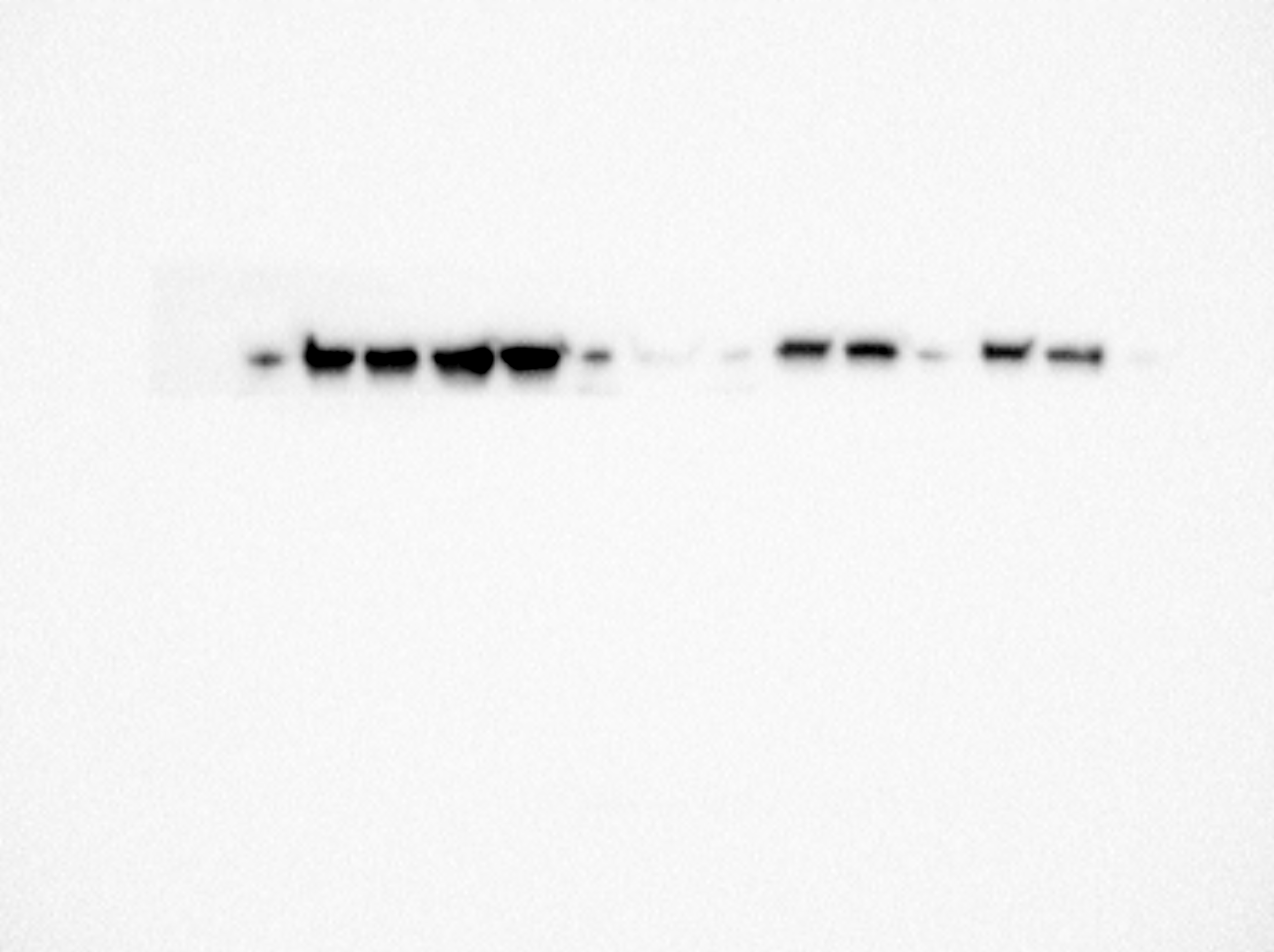

Supplement: Supplementary file 12 — Figure EV4 Source Data [file 44321_2026_452_MOESM12_ESM.zip › Figure EV4/EV4A-B/WB_ Uncropped blots_ AKT.tif]

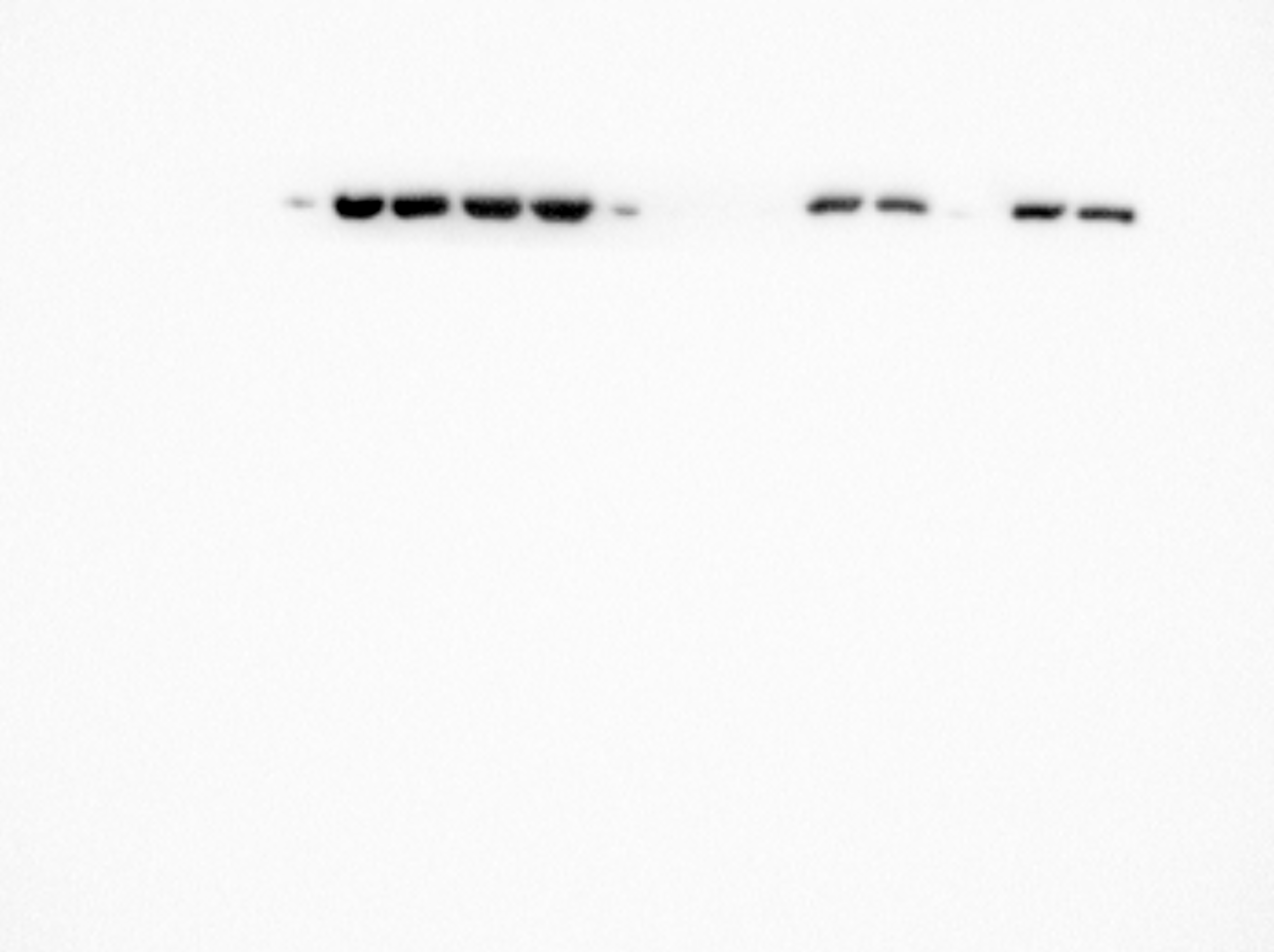

Supplement: Supplementary file 12 — Figure EV4 Source Data [file 44321_2026_452_MOESM12_ESM.zip › Figure EV4/EV4A-B/WB_ Uncropped blots_ β-actin.tif]

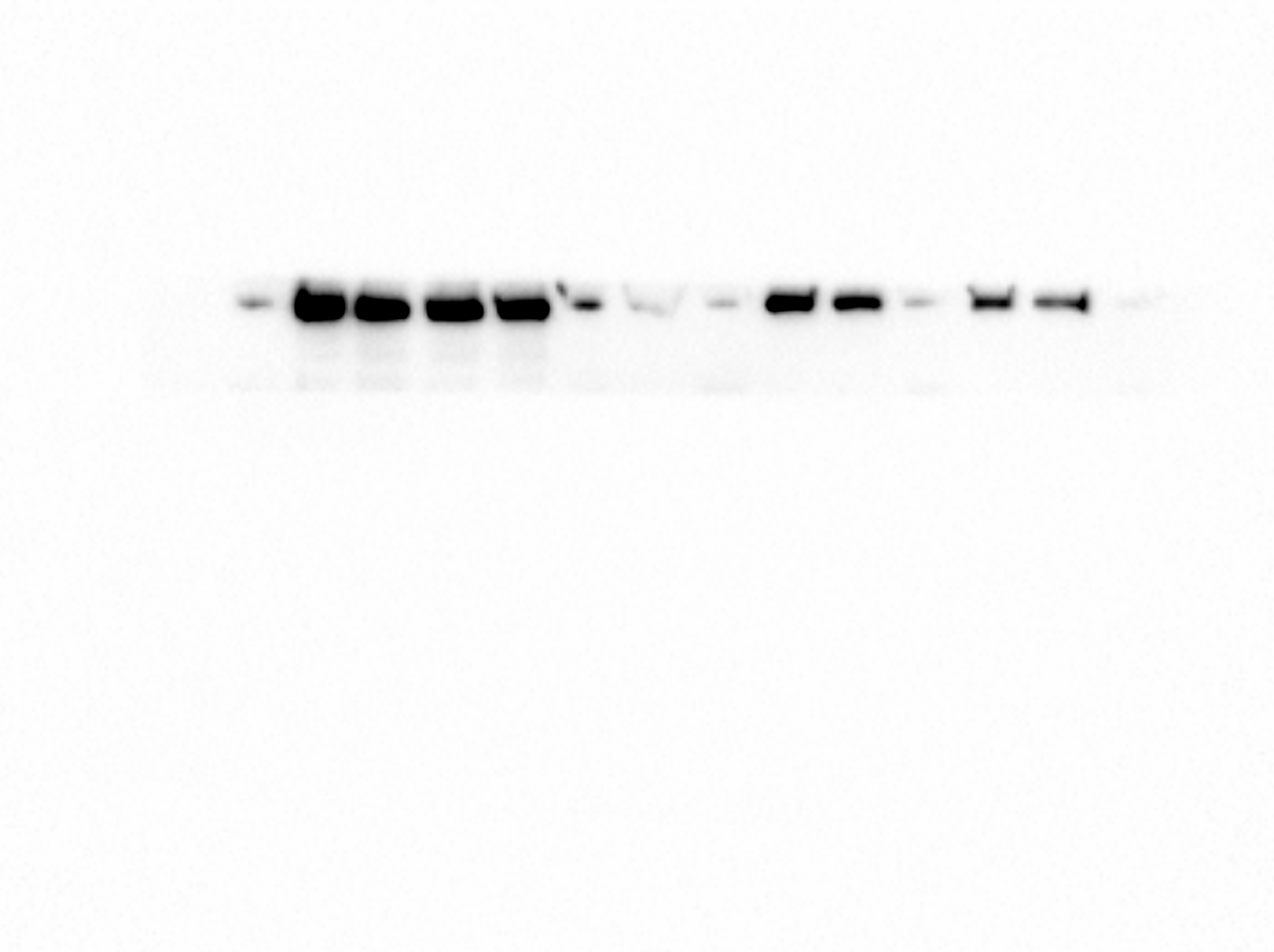

Supplement: Supplementary file 12 — Figure EV4 Source Data [file 44321_2026_452_MOESM12_ESM.zip › Figure EV4/EV4A-B/WB_ Uncropped blots_eNOS.tif]

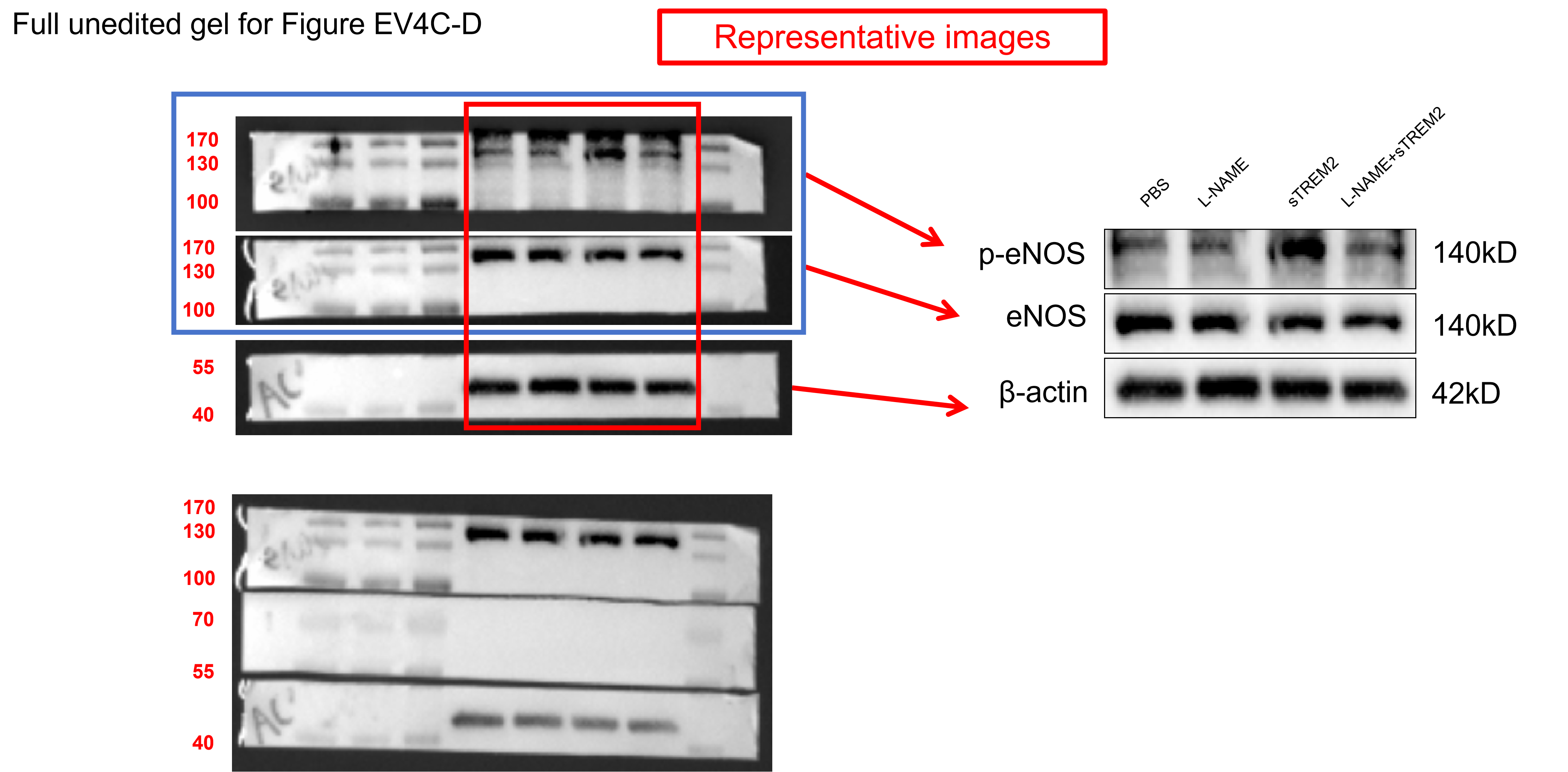

Supplement: Supplementary file 12 — Figure EV4 Source Data [file 44321_2026_452_MOESM12_ESM.zip › Figure EV4/EV4C-D/Instructions for cropping Western blot images.tif]

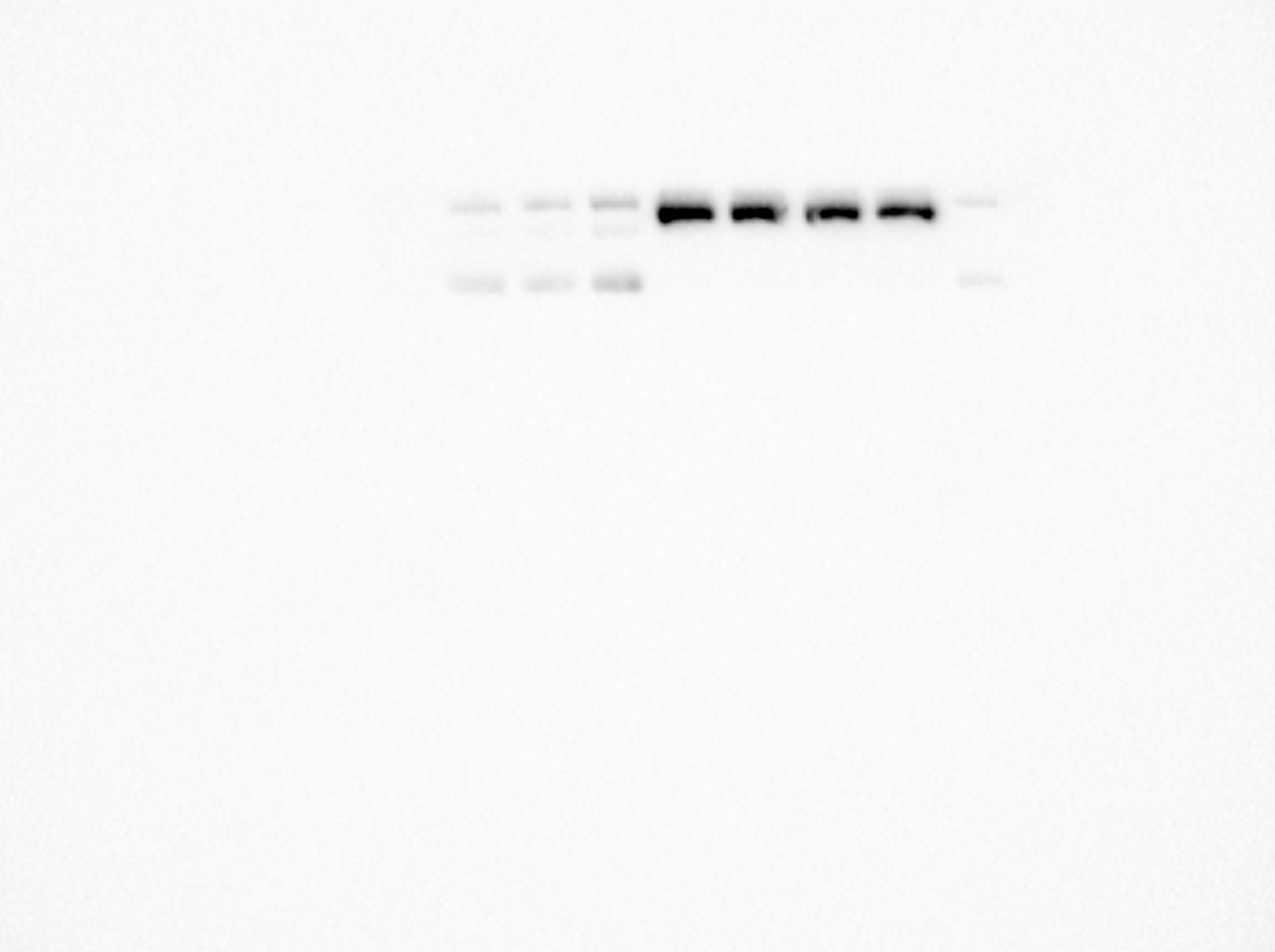

Supplement: Supplementary file 12 — Figure EV4 Source Data [file 44321_2026_452_MOESM12_ESM.zip › Figure EV4/EV4C-D/WB_ Uncropped blots_ eNOS.tif]

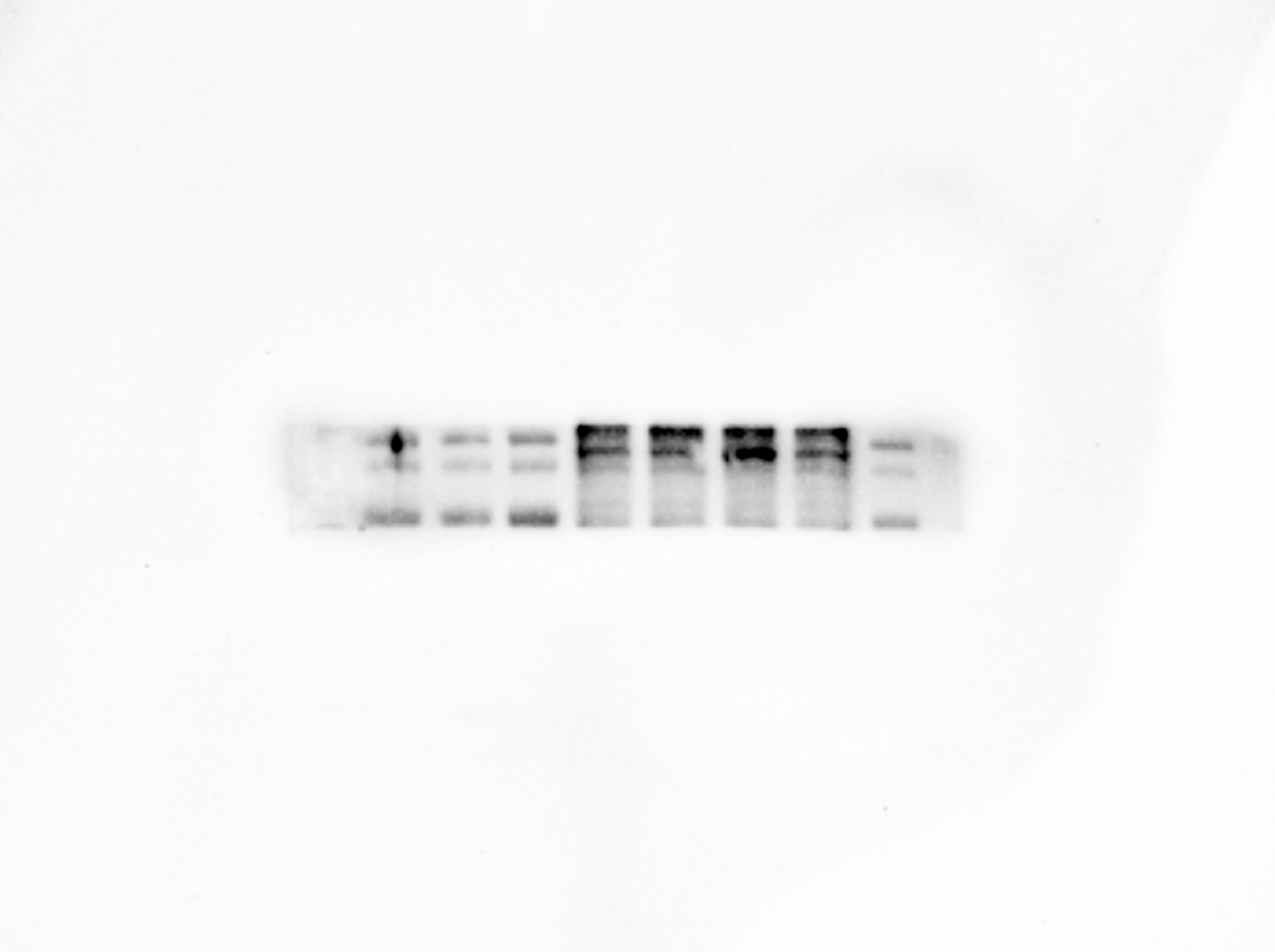

Supplement: Supplementary file 12 — Figure EV4 Source Data [file 44321_2026_452_MOESM12_ESM.zip › Figure EV4/EV4C-D/WB_ Uncropped blots_ p-eNOS.tif]

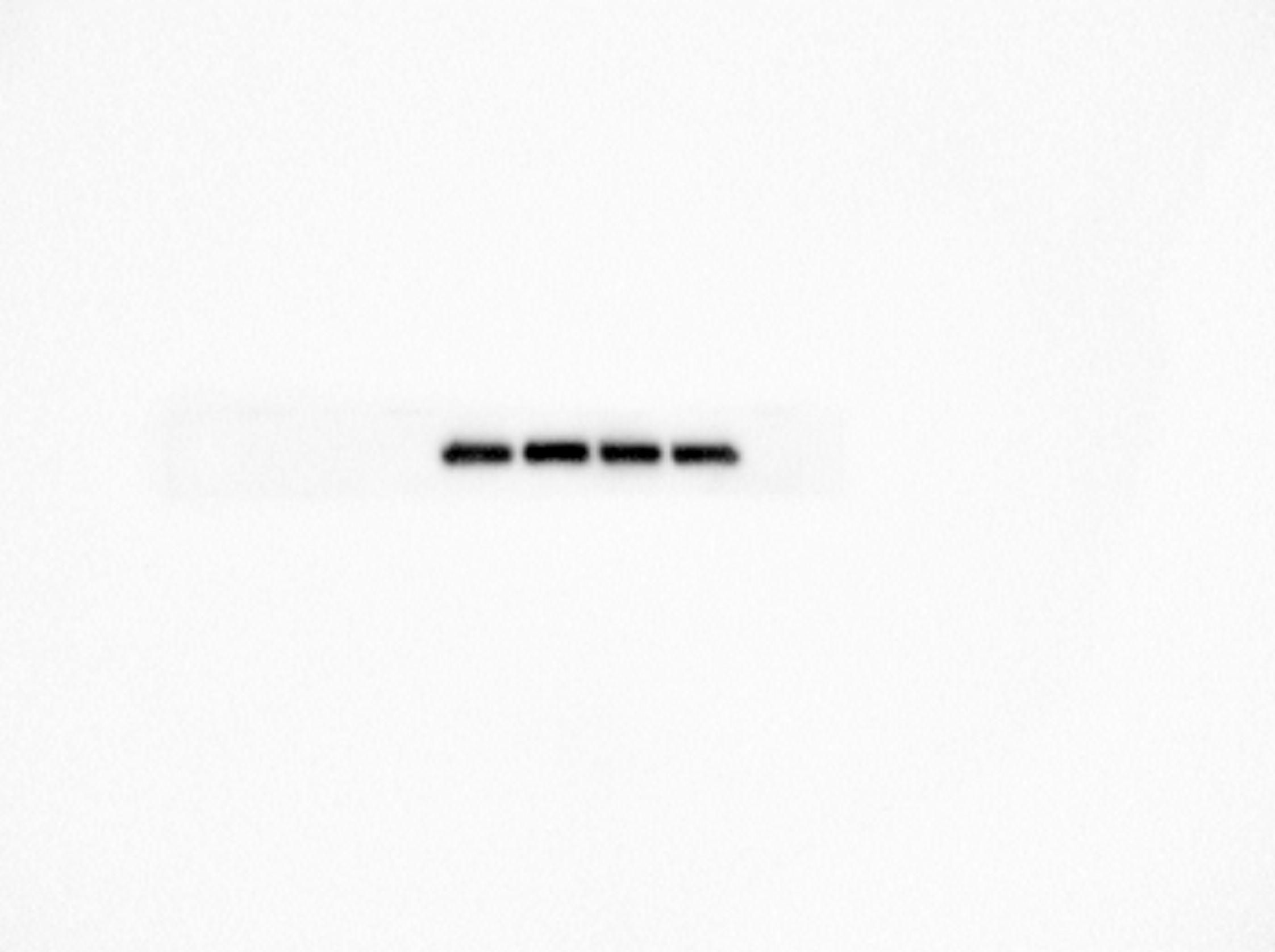

Supplement: Supplementary file 12 — Figure EV4 Source Data [file 44321_2026_452_MOESM12_ESM.zip › Figure EV4/EV4C-D/WB_ Uncropped blots_ β-actin.tif]

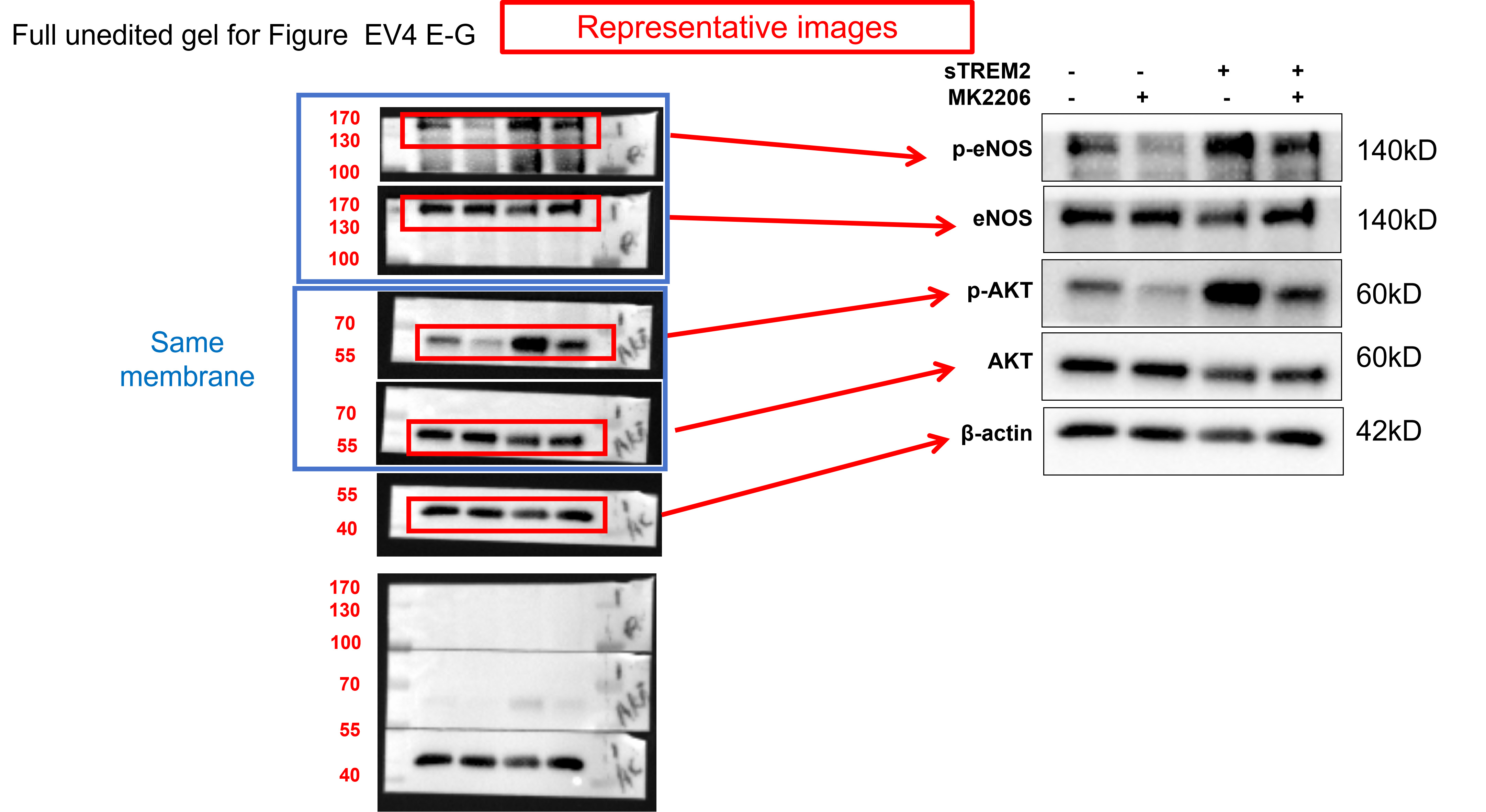

Supplement: Supplementary file 12 — Figure EV4 Source Data [file 44321_2026_452_MOESM12_ESM.zip › Figure EV4/EV4E-G/Instructions for cropping Western blot images 1.tif]

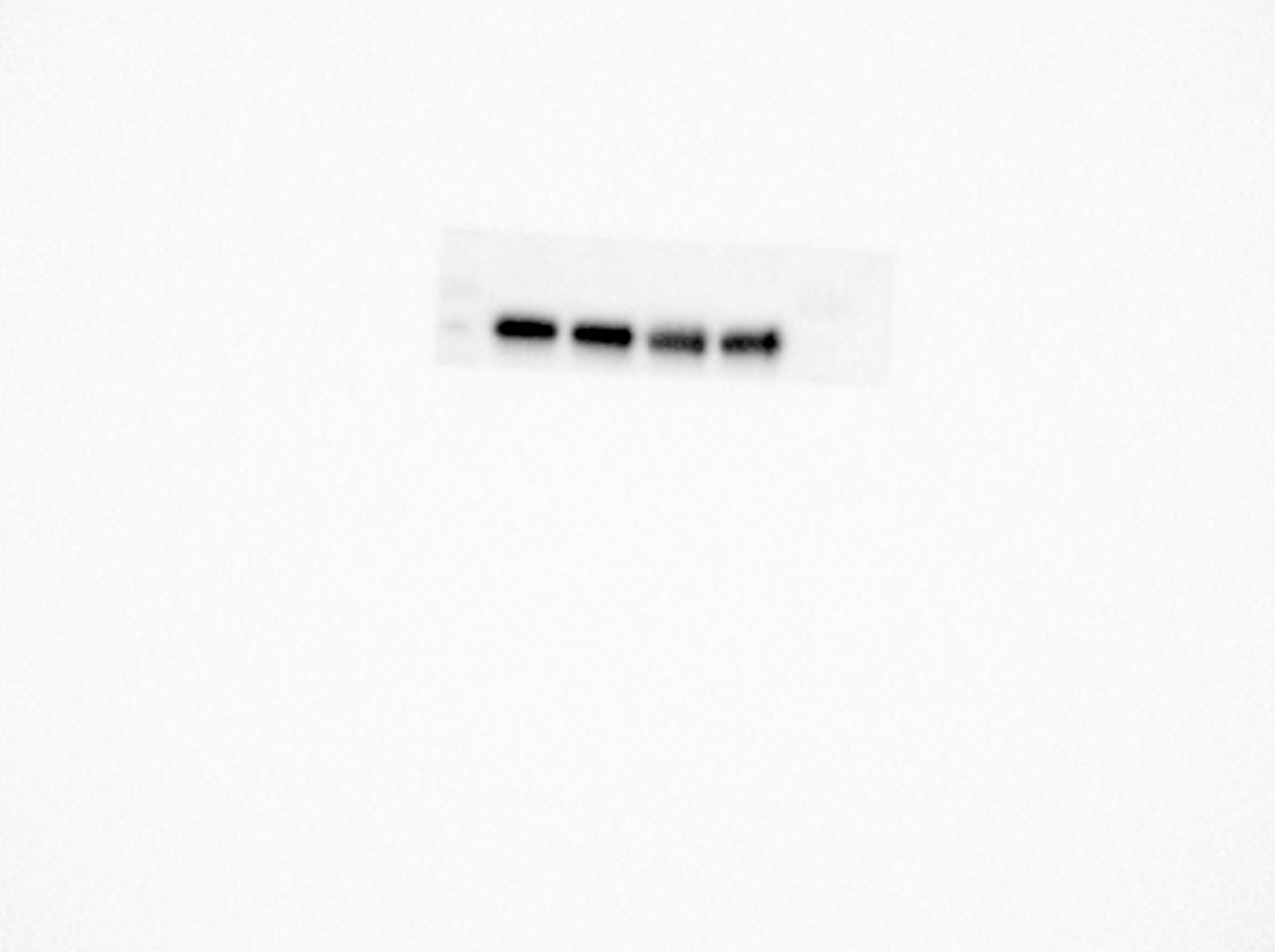

Supplement: Supplementary file 12 — Figure EV4 Source Data [file 44321_2026_452_MOESM12_ESM.zip › Figure EV4/EV4E-G/WB_ Uncropped blots_ AKT.tif]

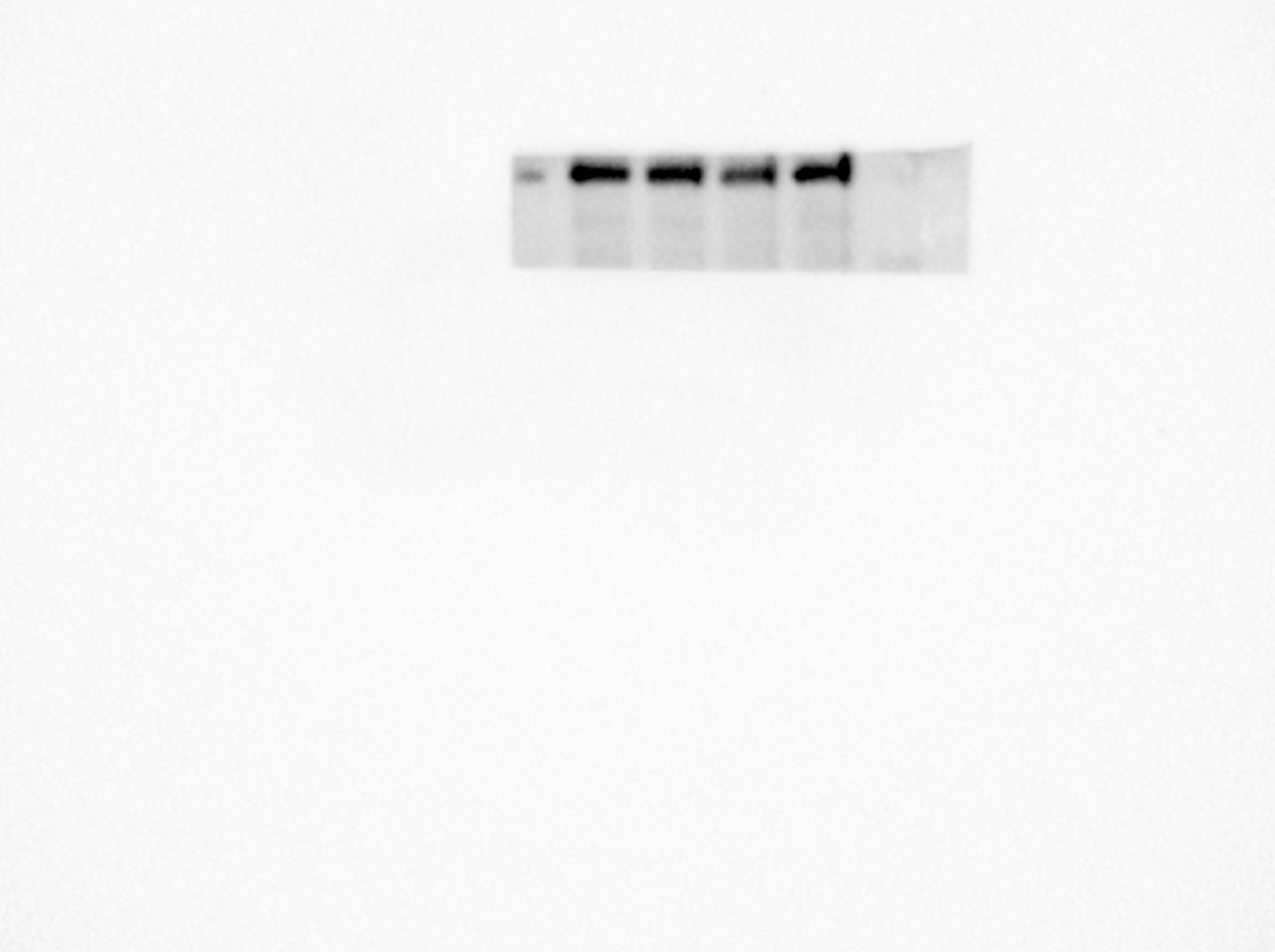

Supplement: Supplementary file 12 — Figure EV4 Source Data [file 44321_2026_452_MOESM12_ESM.zip › Figure EV4/EV4E-G/WB_ Uncropped blots_ eNOS.tif]

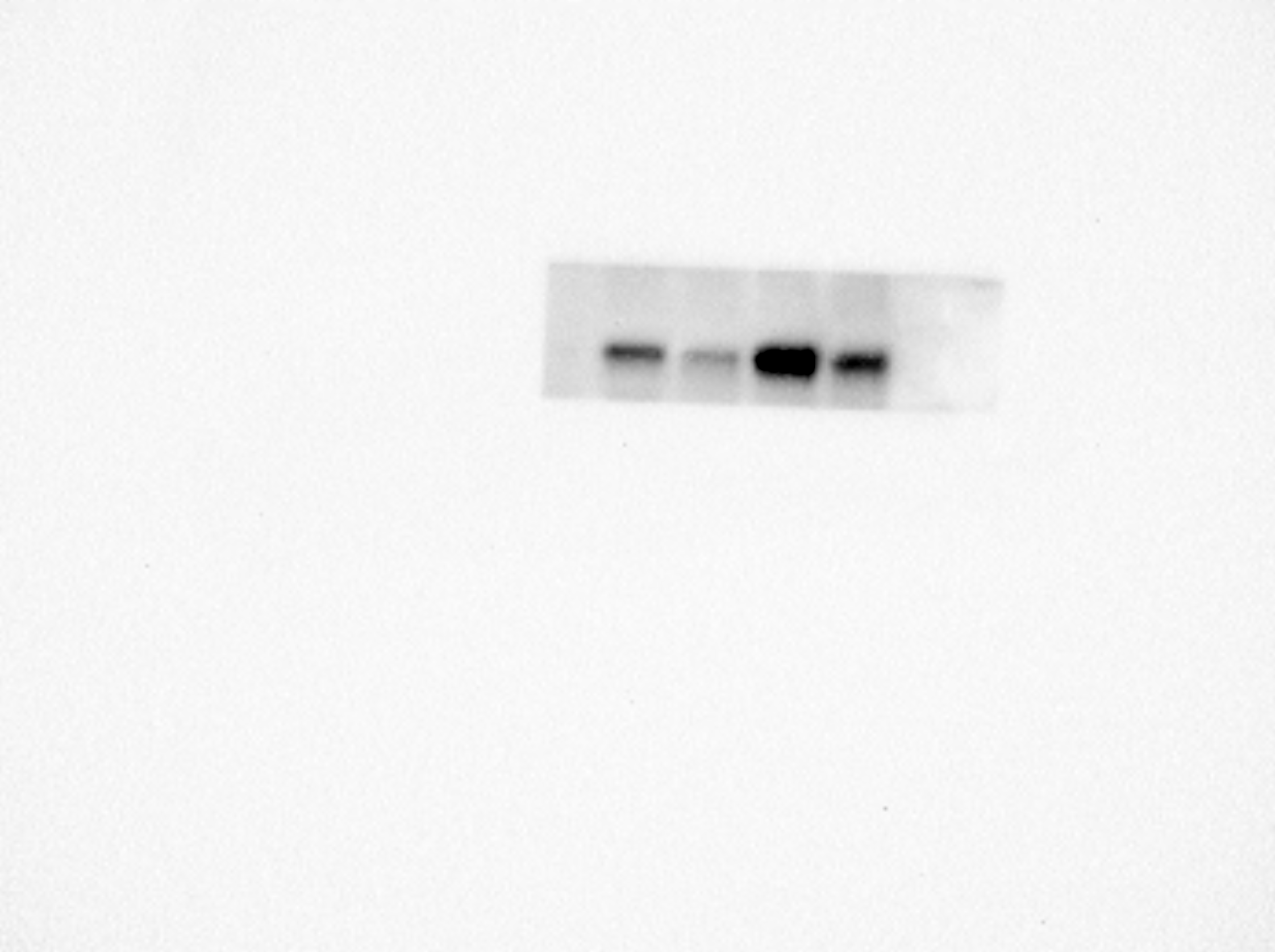

Supplement: Supplementary file 12 — Figure EV4 Source Data [file 44321_2026_452_MOESM12_ESM.zip › Figure EV4/EV4E-G/WB_ Uncropped blots_ p-AKT.tif]

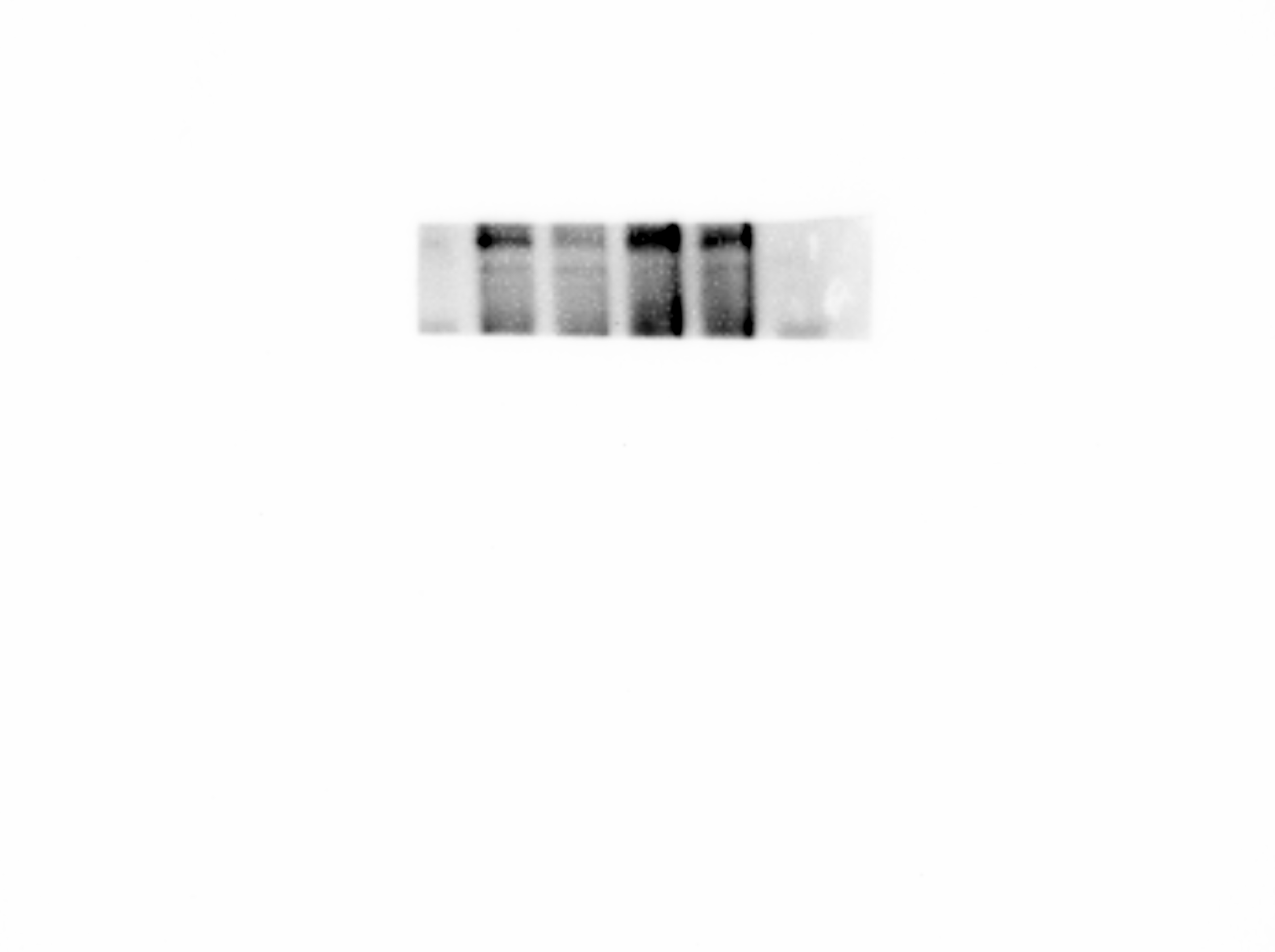

Supplement: Supplementary file 12 — Figure EV4 Source Data [file 44321_2026_452_MOESM12_ESM.zip › Figure EV4/EV4E-G/WB_ Uncropped blots_ p-eNOS.tif]

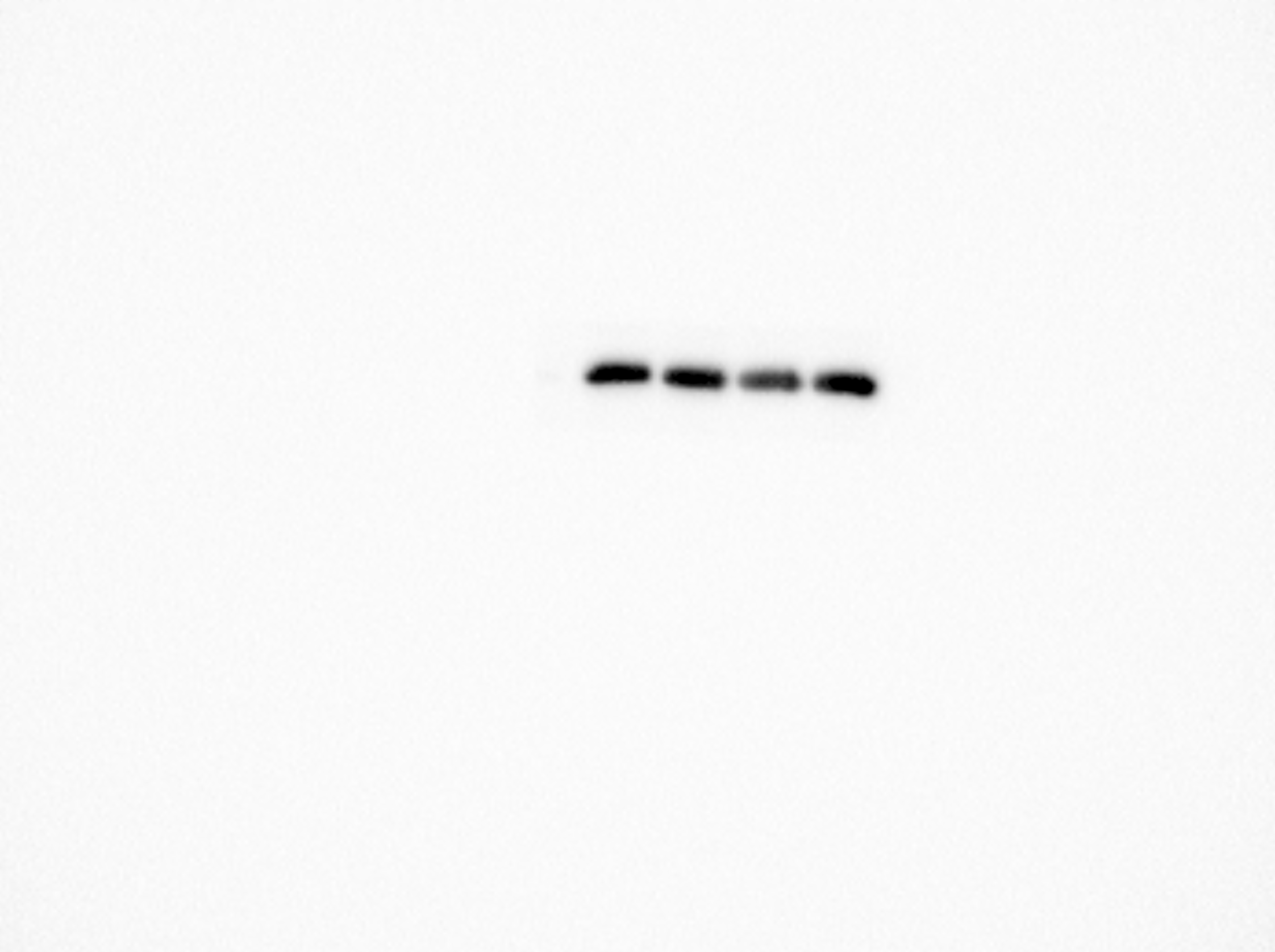

Supplement: Supplementary file 12 — Figure EV4 Source Data [file 44321_2026_452_MOESM12_ESM.zip › Figure EV4/EV4E-G/WB_ Uncropped blots_ β-actin.tif]

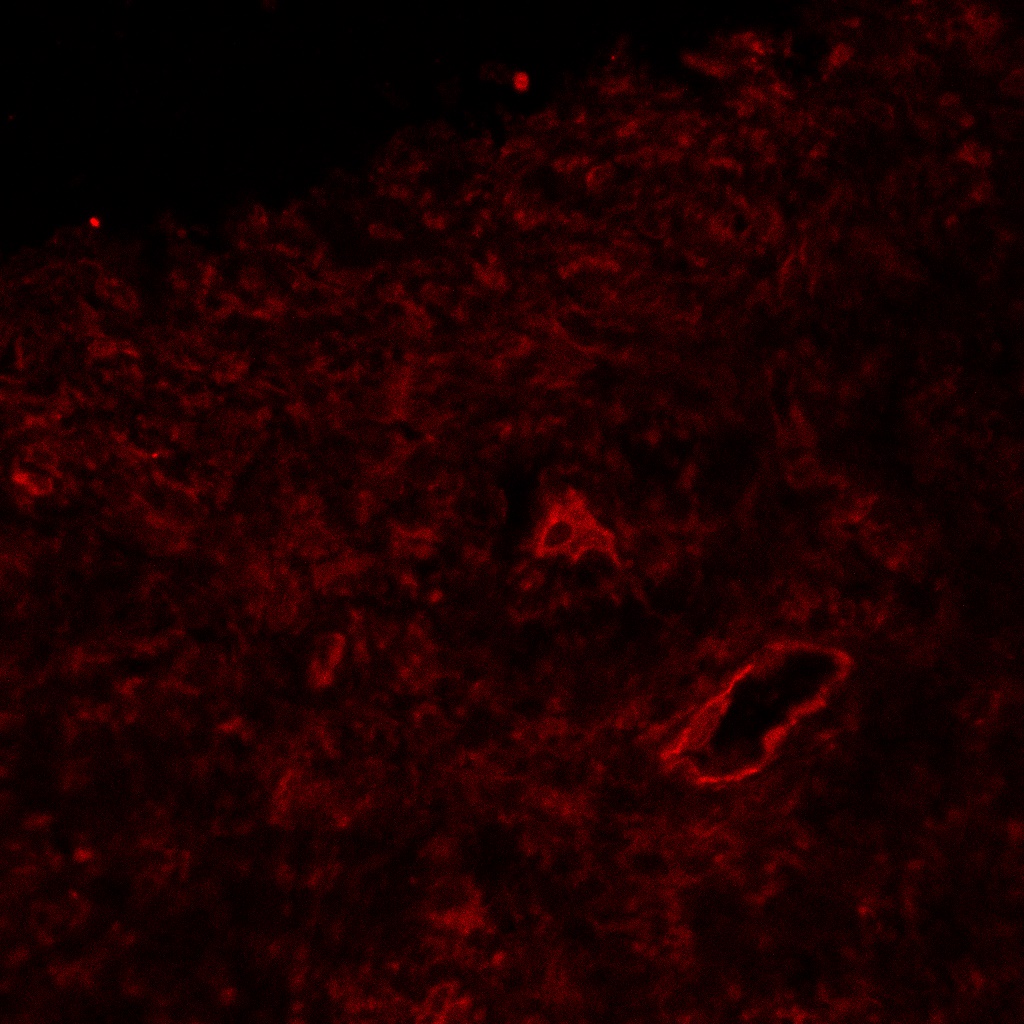

Supplement: Supplementary file 12 — Figure EV4 Source Data [file 44321_2026_452_MOESM12_ESM.zip › Figure EV4/EV4I-J/Ctrl CD31.jpg]

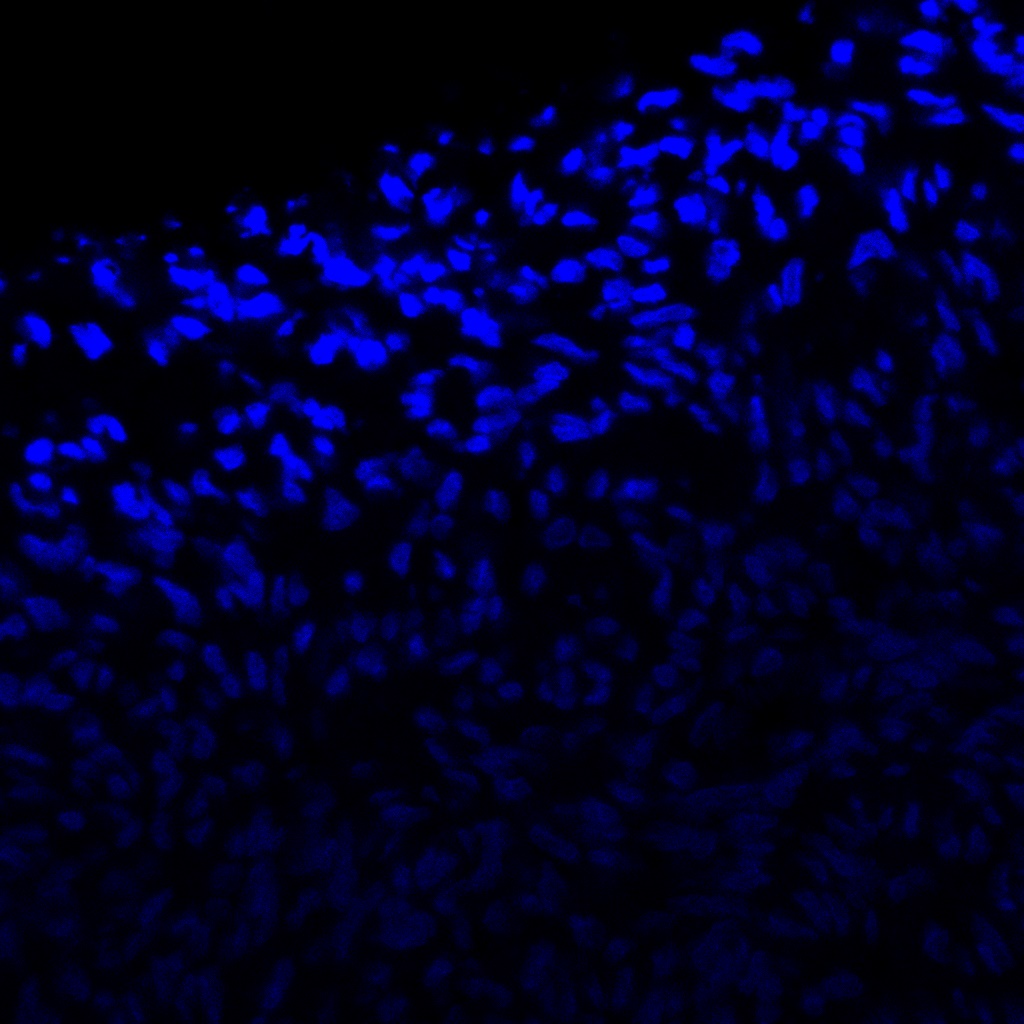

Supplement: Supplementary file 12 — Figure EV4 Source Data [file 44321_2026_452_MOESM12_ESM.zip › Figure EV4/EV4I-J/Ctrl DAPI.jpg]

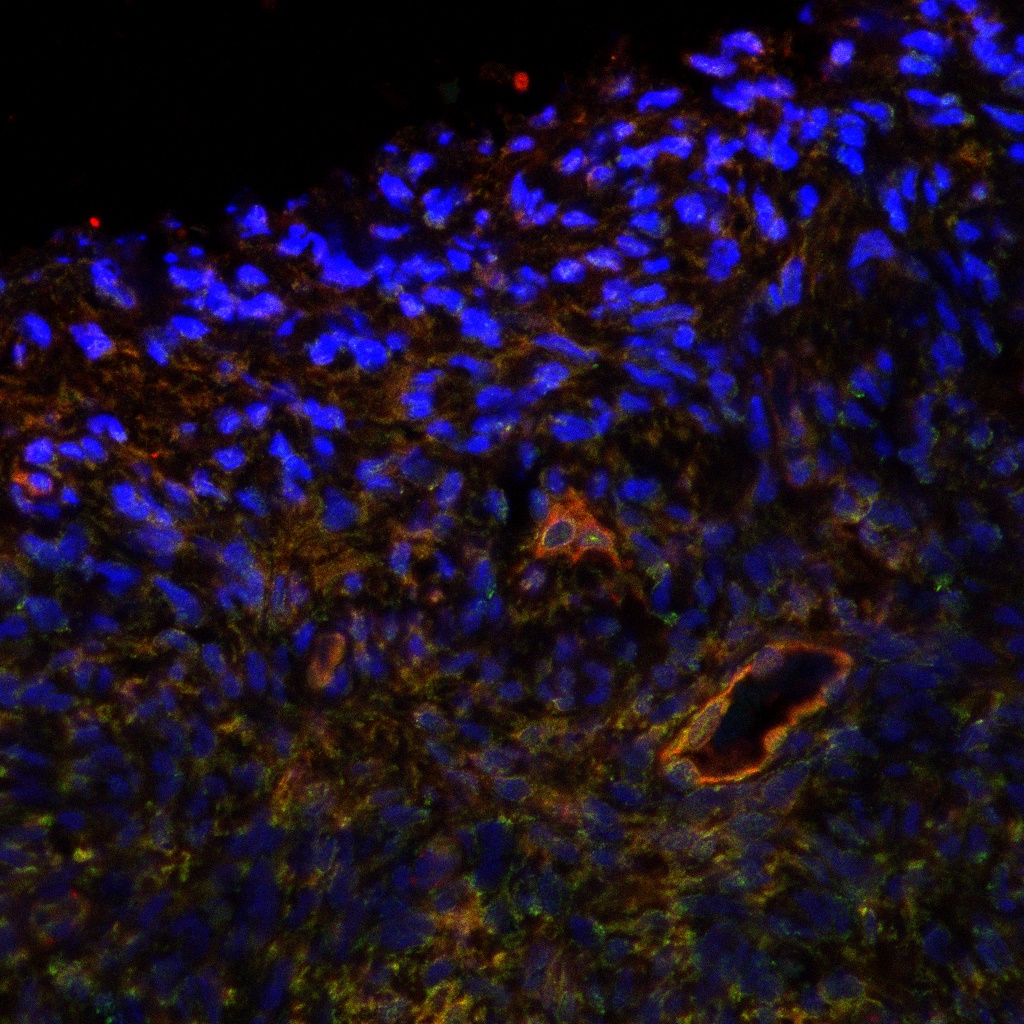

Supplement: Supplementary file 12 — Figure EV4 Source Data [file 44321_2026_452_MOESM12_ESM.zip › Figure EV4/EV4I-J/Ctrl merge.jpg]

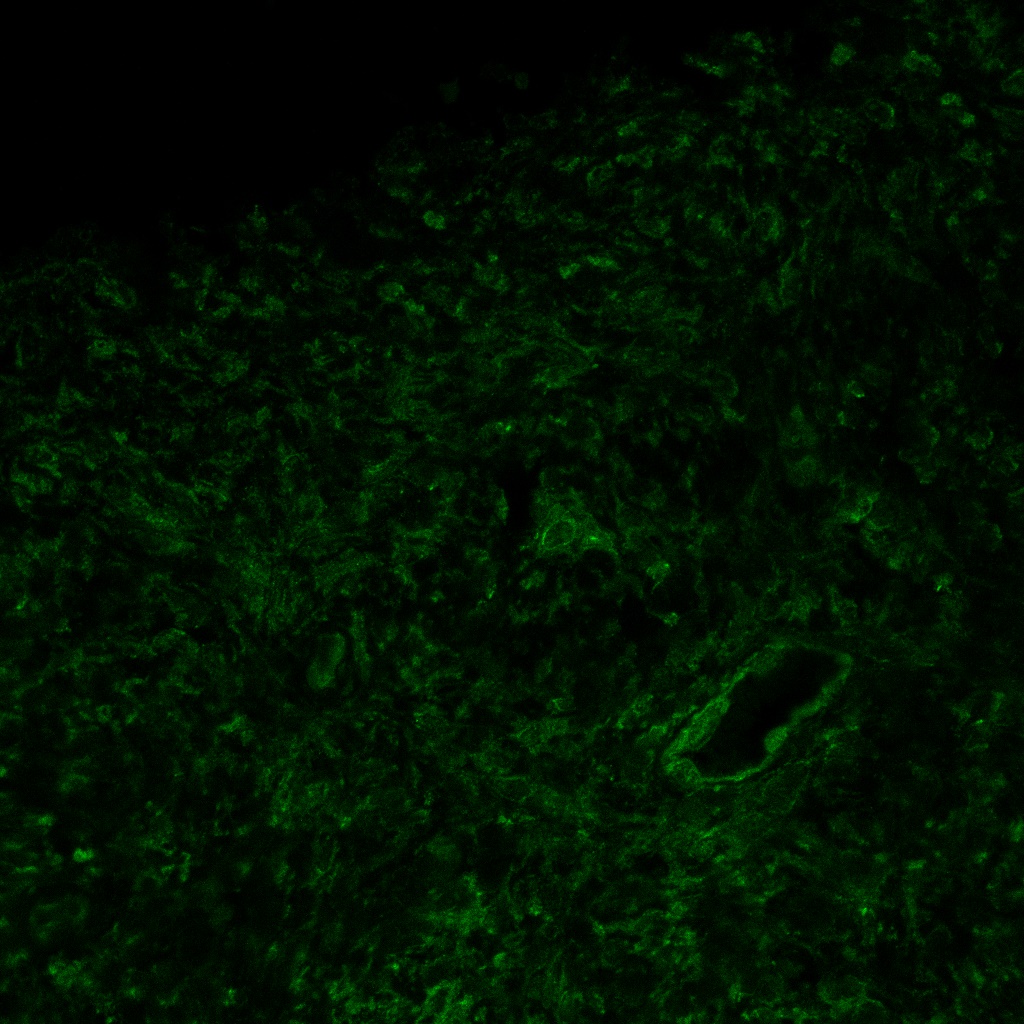

Supplement: Supplementary file 12 — Figure EV4 Source Data [file 44321_2026_452_MOESM12_ESM.zip › Figure EV4/EV4I-J/Ctrl p-eNOS.jpg]

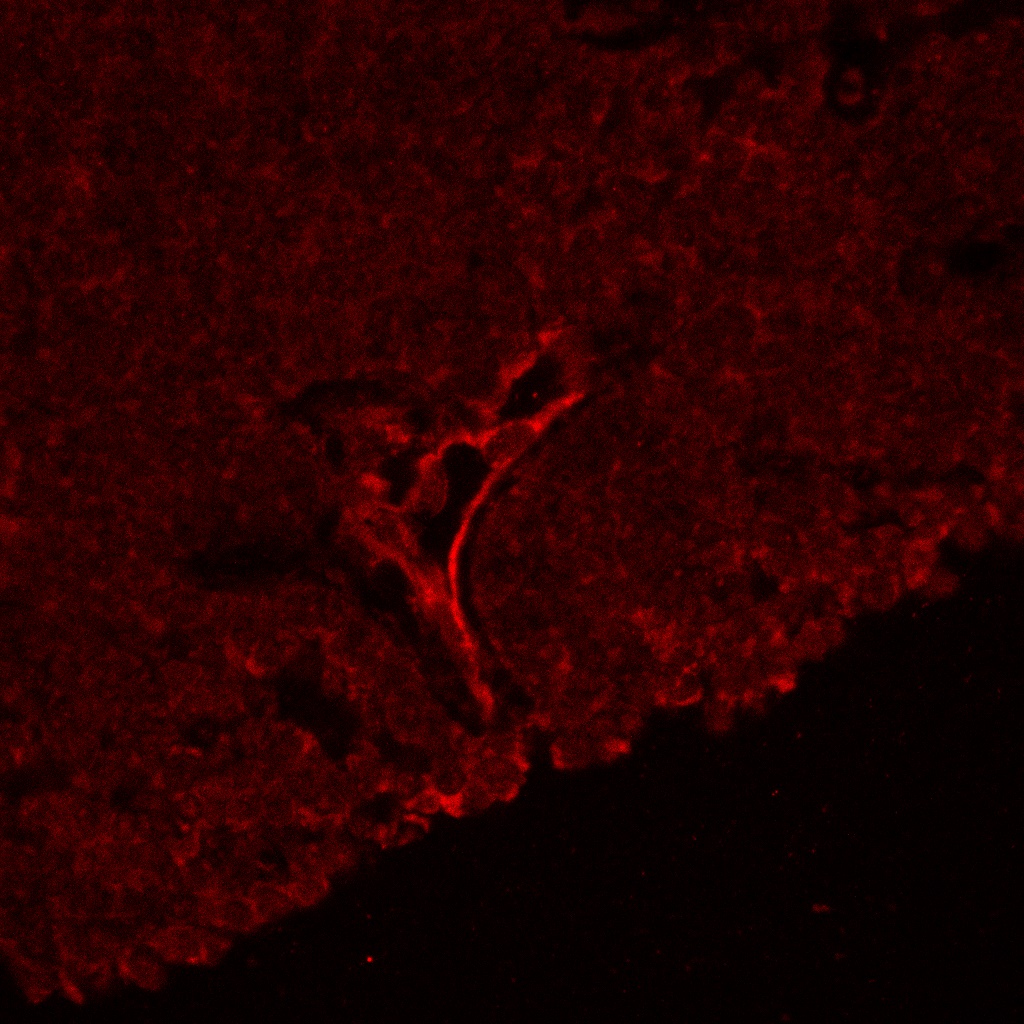

Supplement: Supplementary file 12 — Figure EV4 Source Data [file 44321_2026_452_MOESM12_ESM.zip › Figure EV4/EV4I-J/OC CD31.jpg]

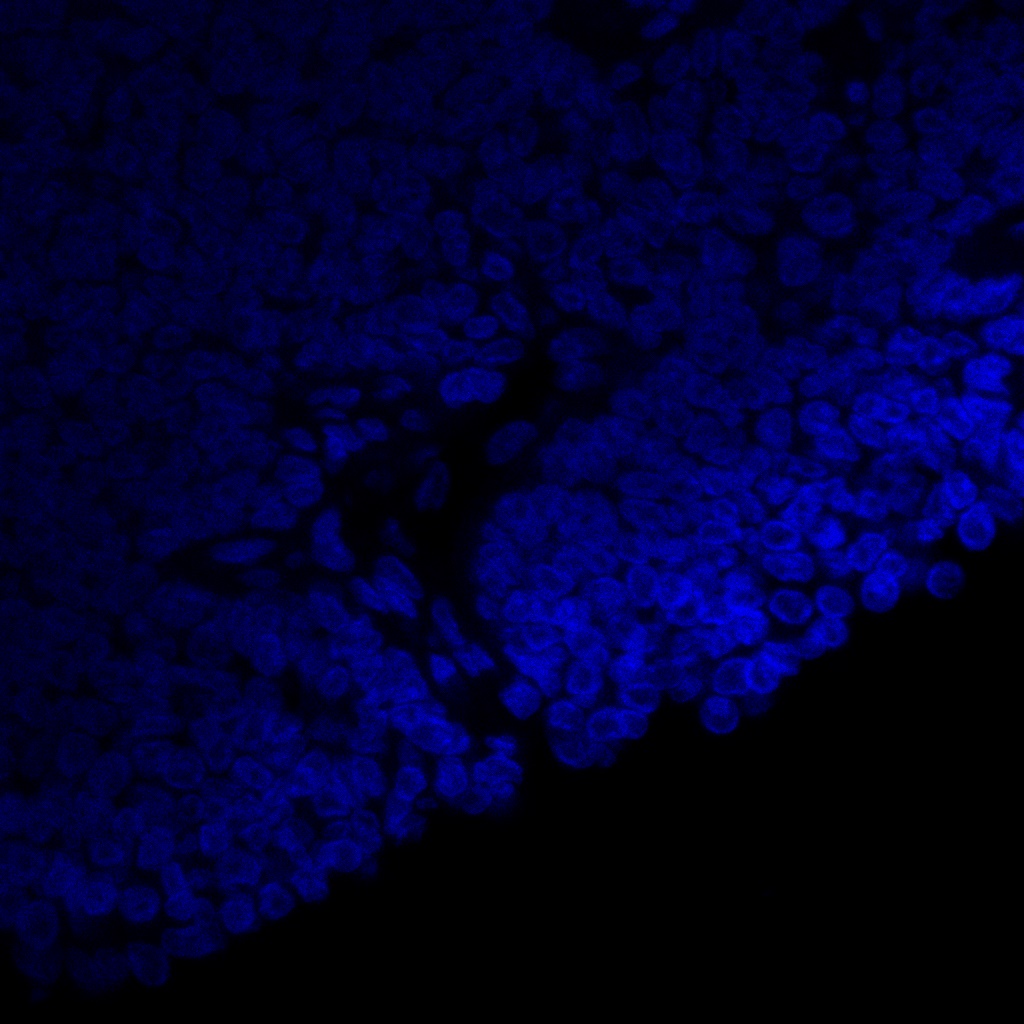

Supplement: Supplementary file 12 — Figure EV4 Source Data [file 44321_2026_452_MOESM12_ESM.zip › Figure EV4/EV4I-J/OC DAPI.jpg]

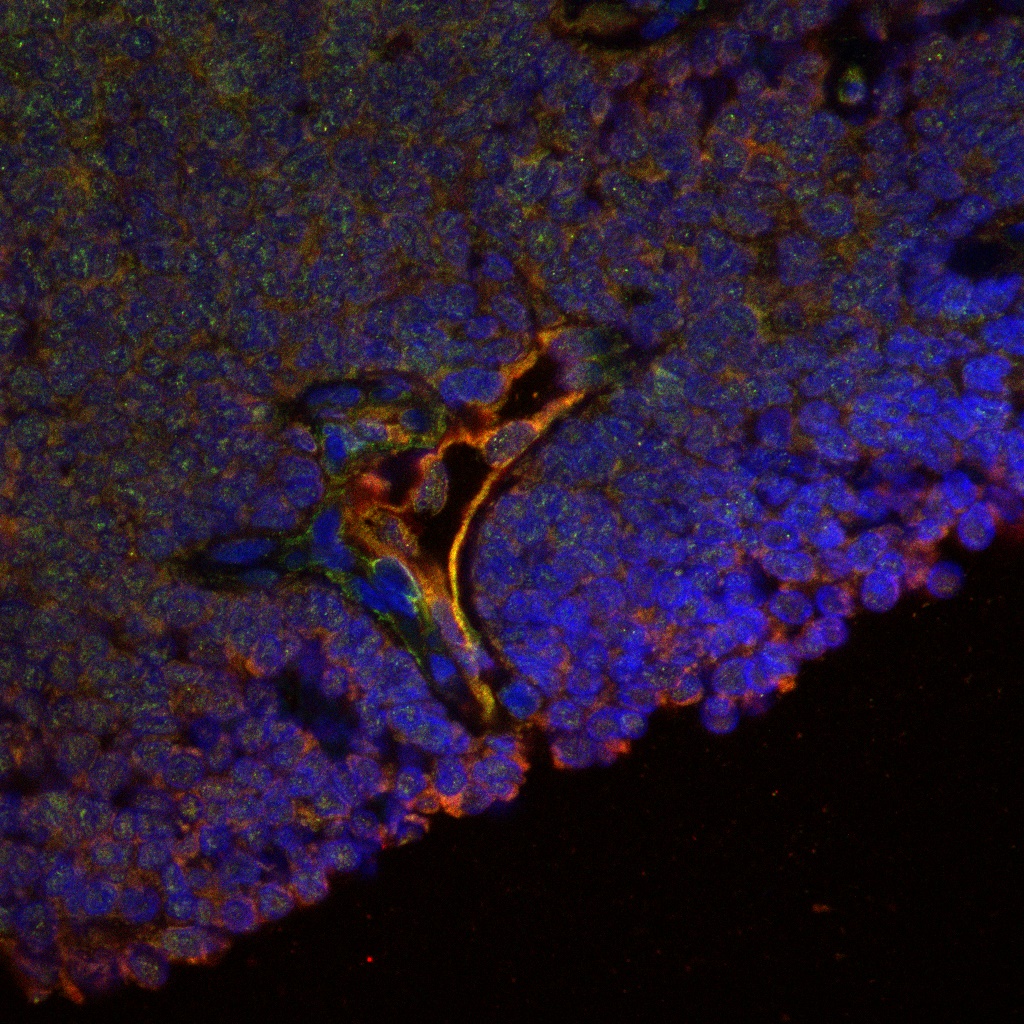

Supplement: Supplementary file 12 — Figure EV4 Source Data [file 44321_2026_452_MOESM12_ESM.zip › Figure EV4/EV4I-J/OC Merge.jpg]

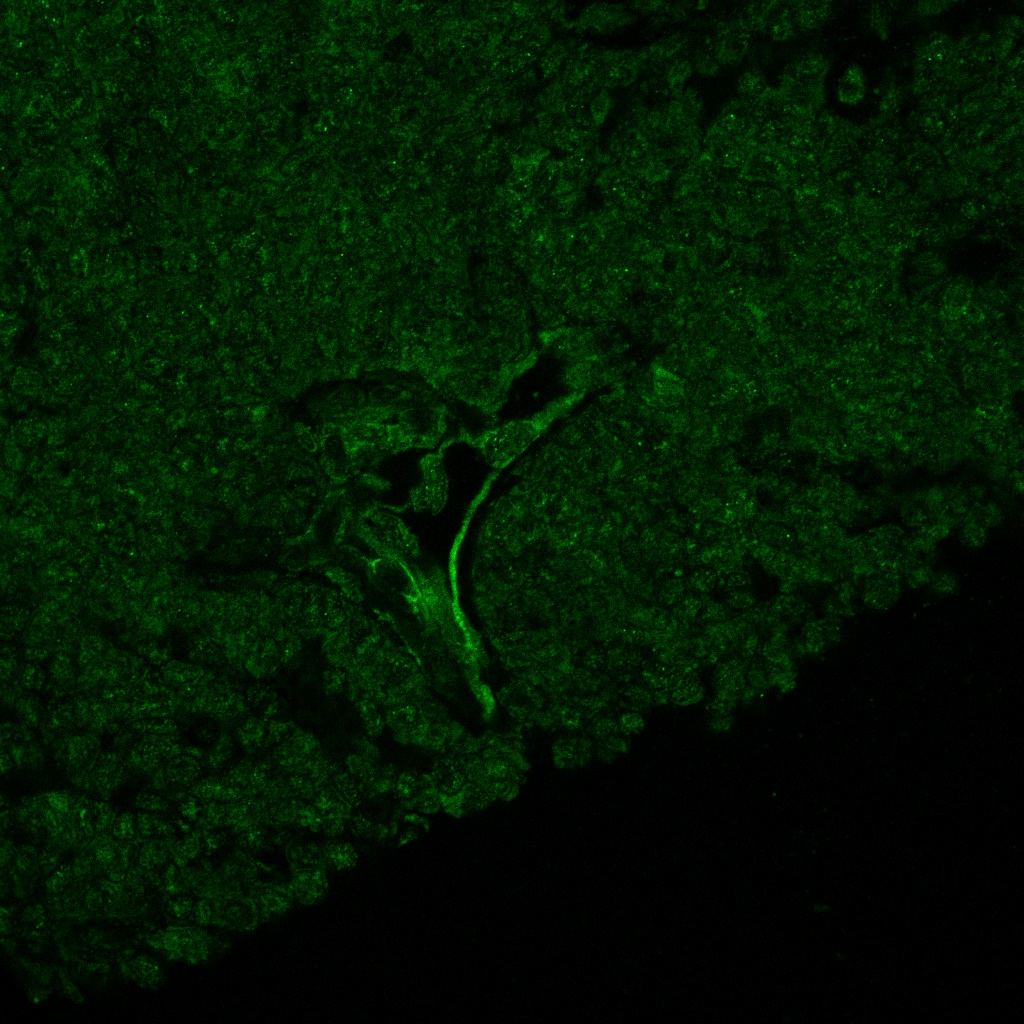

Supplement: Supplementary file 12 — Figure EV4 Source Data [file 44321_2026_452_MOESM12_ESM.zip › Figure EV4/EV4I-J/OC p-eNOS.jpg]

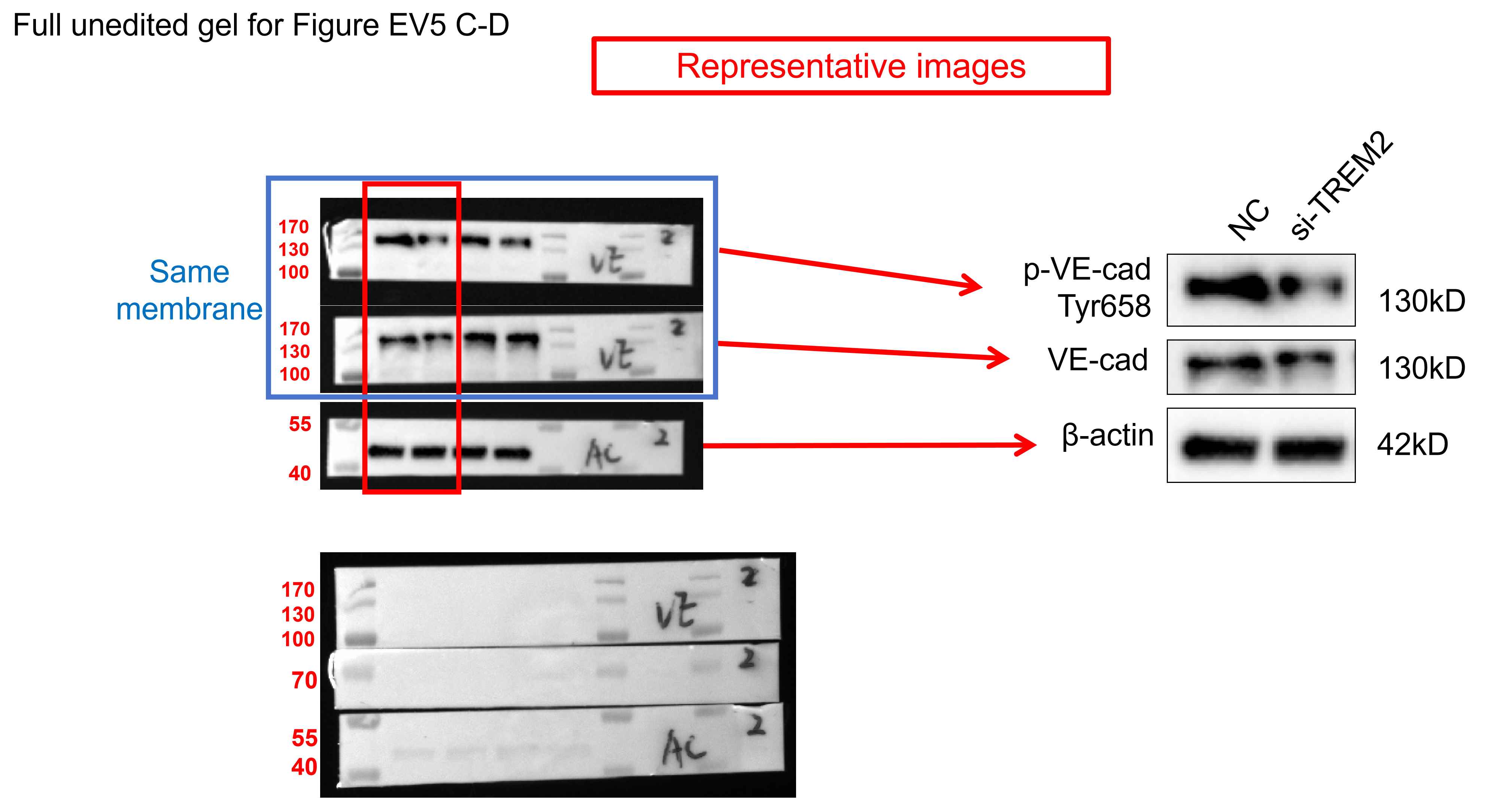

Supplement: Supplementary file 13 — Figure EV5 Source Data [file 44321_2026_452_MOESM13_ESM.zip › Figure EV5/EV5C-D/Instructions for cropping Western blot images 1.tif]

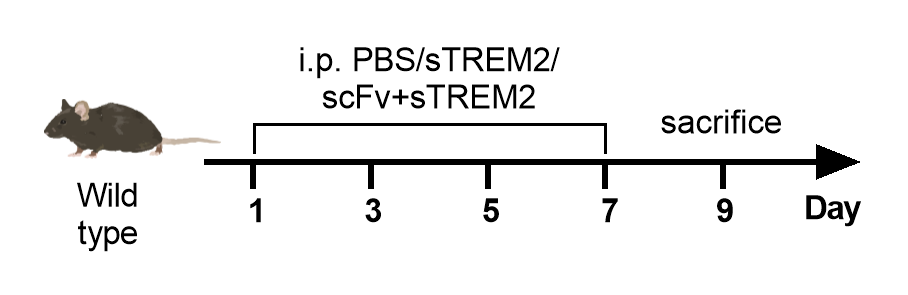

Supplement: Supplementary file 13 — Figure EV5 Source Data [file 44321_2026_452_MOESM13_ESM.zip › Figure EV5/EV5F/Figure EV5F.tif]
