# Supplementary material for: Soluble TREM2 engages cell-surface nucleolin to drive vascular permeability and malignant ascites in ovarian cancer
Source: EMBO Mol Med. 2026 May 26;18(7):2667–90. doi: 10.1038/s44321-026-00452-2 (PMC13365401; doi:10.1038/s44321-026-00452-2)
Supplement: Supplementary file 14 — Expanded View Figures [file 44321_2026_452_MOESM14_ESM.pdf]

Expanded View Figures

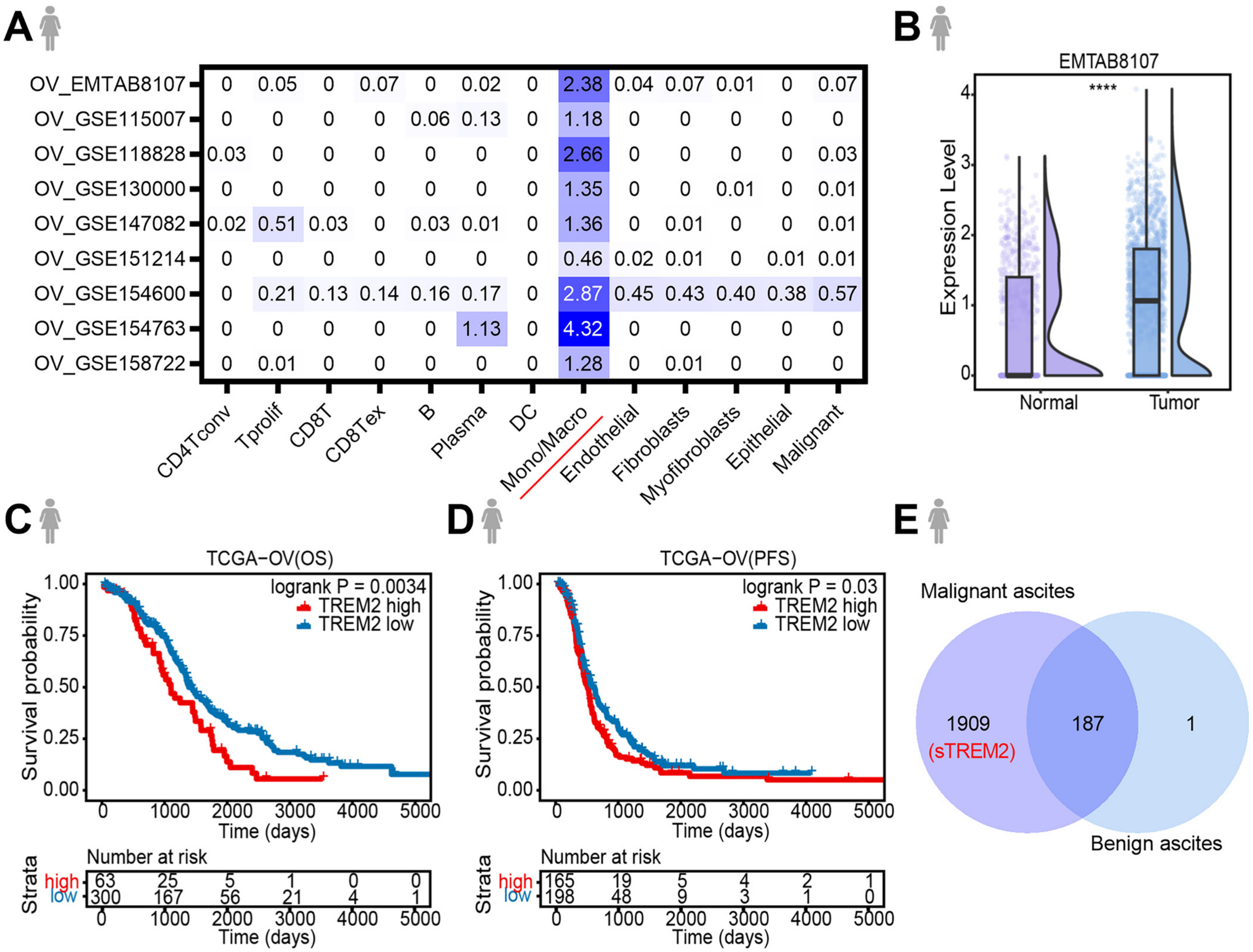

	CD4Tconv	Tprolif	CD8T	CD8Tex	B	Plasma	DC	Mono/Macro	Endothelial	Fibroblasts	Myofibroblasts	Epithelial	Malignant
OV_EMTAB8107	0	0.05	0	0.07	0	0.02	0	2.38	0.04	0.07	0.01	0	0.07
OV_GSE115007	0	0	0	0	0.06	0.13	0	1.18	0	0	0	0	0
OV_GSE118828	0.03	0	0	0	0	0	0	2.66	0	0	0	0	0.03
OV_GSE130000	0	0	0	0	0	0	0	1.35	0	0	0.01	0	0.01
OV_GSE147082	0.02	0.51	0.03	0	0.03	0.01	0	1.36	0	0.01	0	0	0.01
OV_GSE151214	0	0	0	0	0	0	0	0.46	0.02	0.01	0	0.01	0.01
OV_GSE154600	0	0.21	0.13	0.14	0.16	0.17	0	2.87	0.45	0.43	0.40	0.38	0.57
OV_GSE154763	0	0	0	0	0	1.13	0	4.32	0	0	0	0	0
OV_GSE158722	0	0.01	0	0	0	0	0	1.28	0	0.01	0	0	0

Number at risk (C, TCGA-OV(OS)):

Strata	0	1000	2000	3000	4000	5000
high	63	25	5	1	0	0
low	300	167	56	21	4	1

Number at risk (D, TCGA-OV(PFS)):

Strata	0	1000	2000	3000	4000	5000
high	165	19	5	4	2	1
low	198	48	9	3	1	0

Figure EV1. Single-cell and clinical characterization of TREM2 in ovarian cancer.

(**A**) Heatmap displaying TREM2 expression patterns across immune cell populations from multiple single-cell RNA sequencing datasets (tumor immune single-cell hub). (**B**) Raincloud plot validating TREM2 expression in macrophages from the EMTAB-8107 dataset. Boxplots show the median (central line), the upper and lower quartiles (box limits, representing the 25th and 75th percentiles), and the whiskers indicating the minimum and maximum values (full range). (**C**, **D**) Kaplan–Meier survival analysis of (**C**) overall survival (OS) and (**D**) progression-free survival (PFS) in TCGA ovarian cancer patients stratified by TREM2 expression levels. (**E**) Venn diagram showing differentially expressed proteins between malignant and benign ascites. Data were shown as mean ± SEM. ***$P < 0.001$. Exact p values are provided in Appendix Table S2. Two-tailed Student's t-test for (**B**); log-rank test for (**C**, **D**). Source data are available online for this figure.

 © The Author(s)

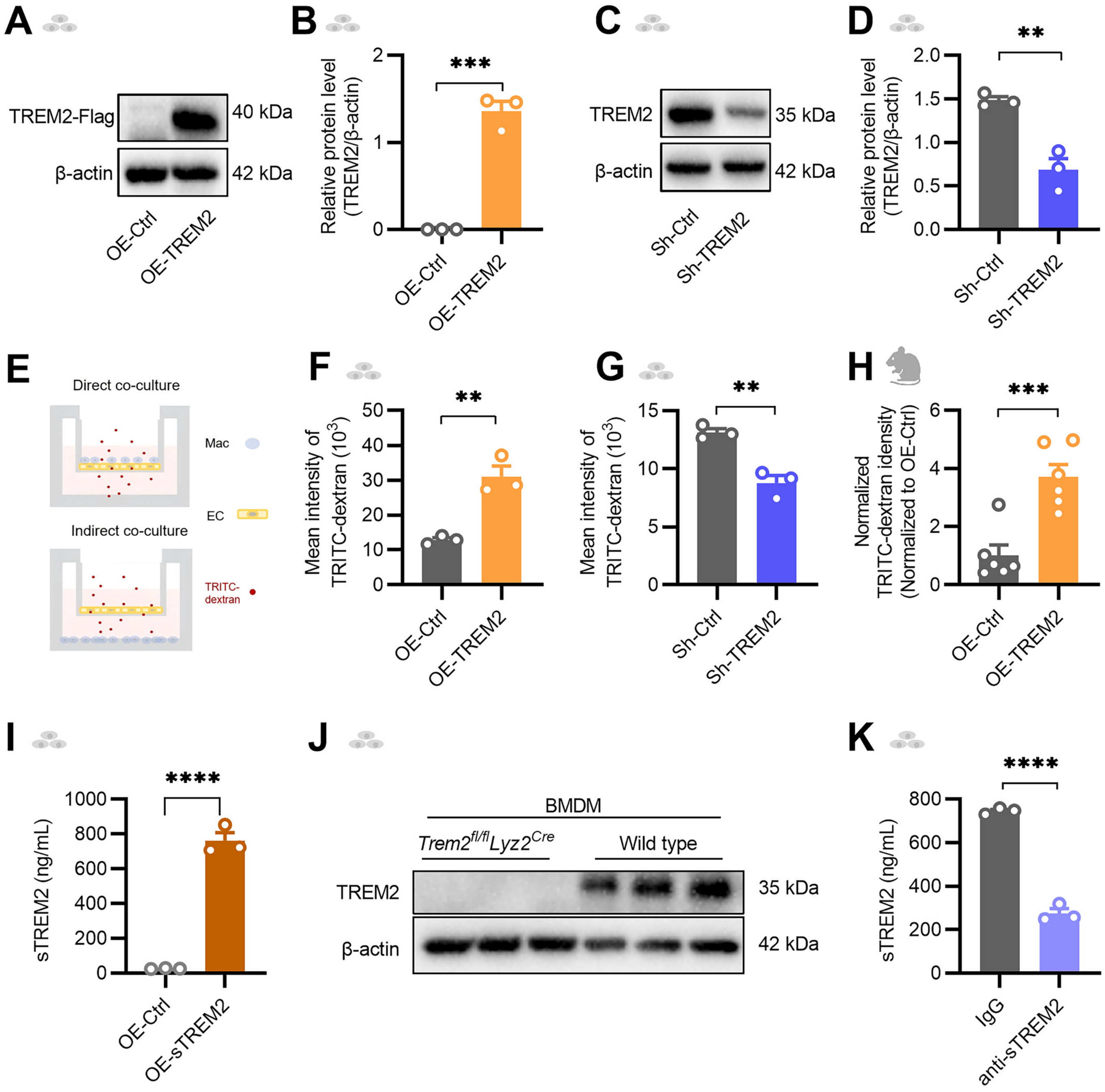

Figure EV2. sTREM2 modulates vascular permeability through macrophage-dependent and independent mechanisms.

(**A**, **B**) Western blot analysis (**A**) and quantification (**B**) of TREM2 expression in iBMDM cells overexpressing TREM2 (OE-TREM2) versus empty vector control (OE-Ctrl, $n = 3$ biological replicates). β-actin was used as a loading control. (**C**, **D**) Western blot analysis (**C**) and quantification (**D**) of TREM2 expression in iBMDM cells with TREM2 knockdown (Sh-TREM2) versus scramble control (Sh-Ctrl, $n = 3$ biological replicates). β-actin was used as a loading control. (**E**) Schematic diagram of direct and indirect coculture systems. (**F**) TRITC-dextran flux in C166 endothelial cells directly cocultured with OE-sTREM2 or OE-Ctrl iBMDM cells ($n = 3$ biological replicates). (**G**) TRITC-dextran flux in C166 cells cocultured with Sh-TREM2 or Sh-Ctrl iBMDM cells ($n = 3$ biological replicates). (**H**) Peritoneal vascular permeability in mice injected intraperitoneally with 1×10^6 TREM2-overexpressing iBMDM versus control iBMDM cells ($n = 6$ mice). (**I**) sTREM2 levels in the culture supernatants of THP-1 cells, detected by ELISA ($n = 3$ biological replicates). (**J**) Western blot validation of TREM2 knockout in bone marrow-derived macrophages from *Trem2*$^{fl/fl}$*Lyz2*^{Cre} mice. (**K**) sTREM2 levels in conditioned medium from sTREM2-overexpressing THP-1 cells before and after immunoprecipitation-mediated depletion with an anti-sTREM2 antibody, detected by ELISA ($n = 3$ biological replicates). Data were mean ± SEM. $^{**}P < 0.01$, $^{***}P < 0.001$, $^{****}P < 0.0001$. Exact p values are provided in Appendix Table S2. Two-tailed Student's t-test for (**B**, **D**, **F**–**I**, **K**). Source data are available online for this figure.

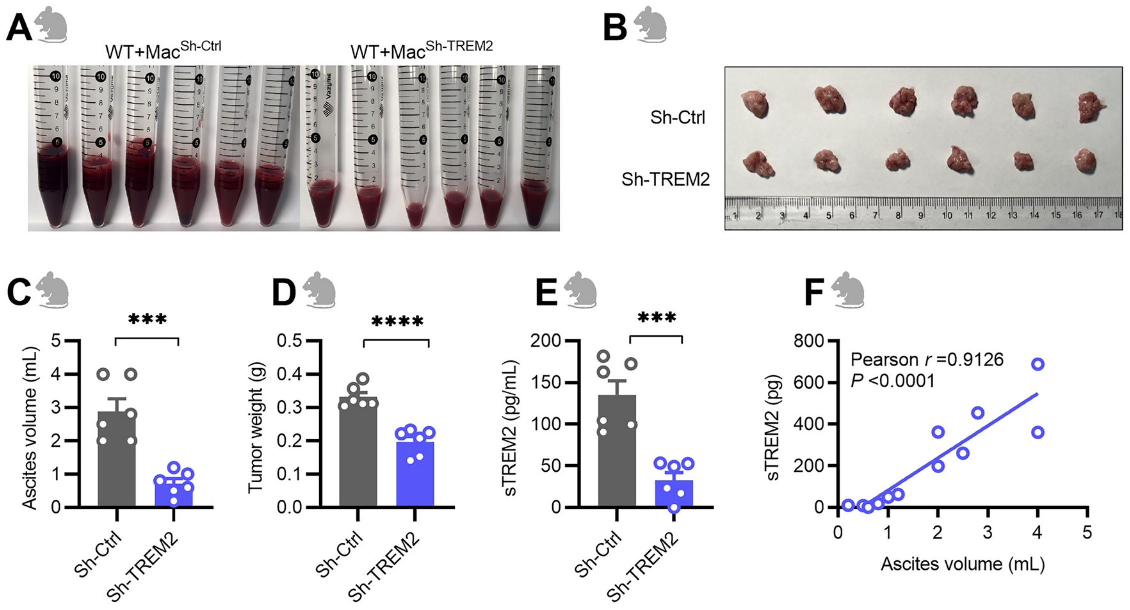

Figure EV3. TREM2 knockdown inhibits malignant ascites formation in ovarian cancer.

(**A–D**) Representative images (**A**, **B**) and quantification (**C**, **D**) of ascites (**A**, **C**) and tumor burden (**B**, **D**) in mice injected intraperitoneally with ID8 cells and Sh-TREM2 iBMDM cells versus Sh-Ctrl iBMDM ($n = 6$ mice per group). (**E**) sTREM2 levels in ascitic supernatants from (**A**), detected by ELISA ($n = 6$ mice per group). (**F**) Correlation analysis between ascites volume and sTREM2 concentration from (**E**, $n = 12$ mice). Data were shown as mean ± SEM. ***$P < 0.001$; ****$P < 0.0001$. Exact p values are provided in Appendix Table S2. Two-tailed Student's t-test for (**C–E**); Pearson correlation for (**F**). Source data are available online for this figure.

 © The Author(s)

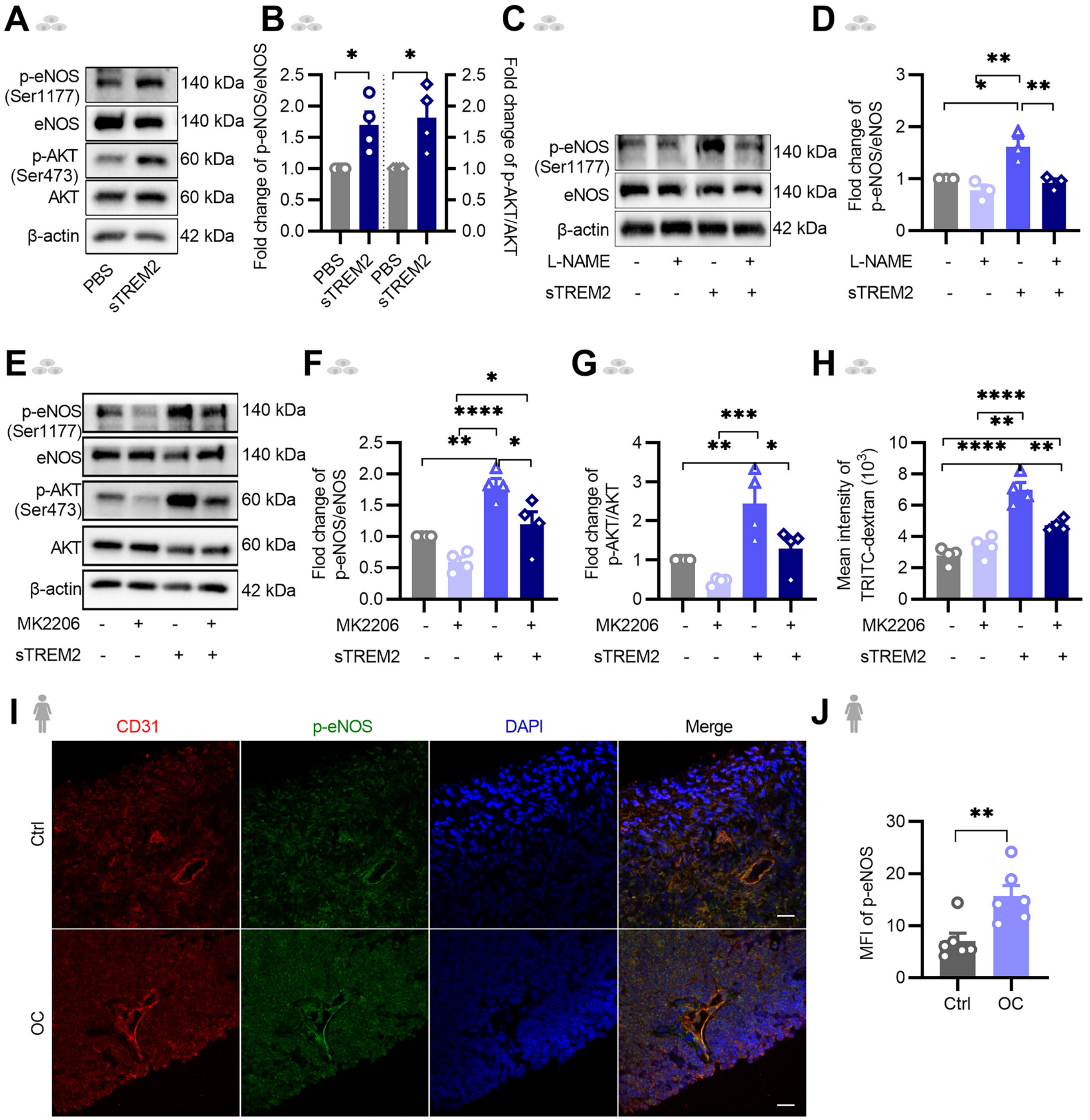

Figure EV4. Pharmacological inhibition of AKT/eNOS signaling blocks sTREM2-mediated effects.

Pharmacological inhibition of AKT/eNOS signaling blocks sTREM2-mediated effects (**A**, **B**) Western blot analysis (**A**) and quantification (**B**) of p-AKT and p-eNOS in 500 ng/mL sTREM2-treated C166 cells ($n = 4$ biological replicates). β-actin was used as a loading control. (**C**, **D**) Western blot analysis (**C**) and quantification (**D**) of eNOS phosphorylation inhibition by 1 mM L-NAME for 30 min ($n = 3$ biological replicates). β-actin was used as a loading control. (**E–G**) Western blot analysis (**E**) and quantification (**F**, **G**) of AKT and eNOS phosphorylation in HUVECs incubated with 1uM MK2206 for 1 h prior to treatment with 500 ng/mL sTREM2 ($n = 4$ biological replicates). β-actin was used as a loading control. (**H**) TRITC-dextran permeability assay in HUVECs treated with sTREM2 ± AKT inhibitor MK2206 ($n = 4$ biological replicates). (**I**, **J**) Immunofluorescence analysis (**I**) and quantification (**J**) of p-eNOS (green) in the vasculature of human OC tissues ($n = 6$ patient samples per group). CD31 (red) marks endothelium; nuclei are counterstained with DAPI (blue). MFI stands for mean fluorescence intensity. Scale bar: 20 μm. Data were shown as mean ± SEM. *$P < 0.05$; **$P < 0.01$; ***$P < 0.001$; ****$P < 0.0001$. Exact *p* values are provided in Appendix Table S2. Two-tailed Student's *t*-test for (**B**, **J**); one-way ANOVA for (**D**, **F–H**). Source data are available online for this figure.

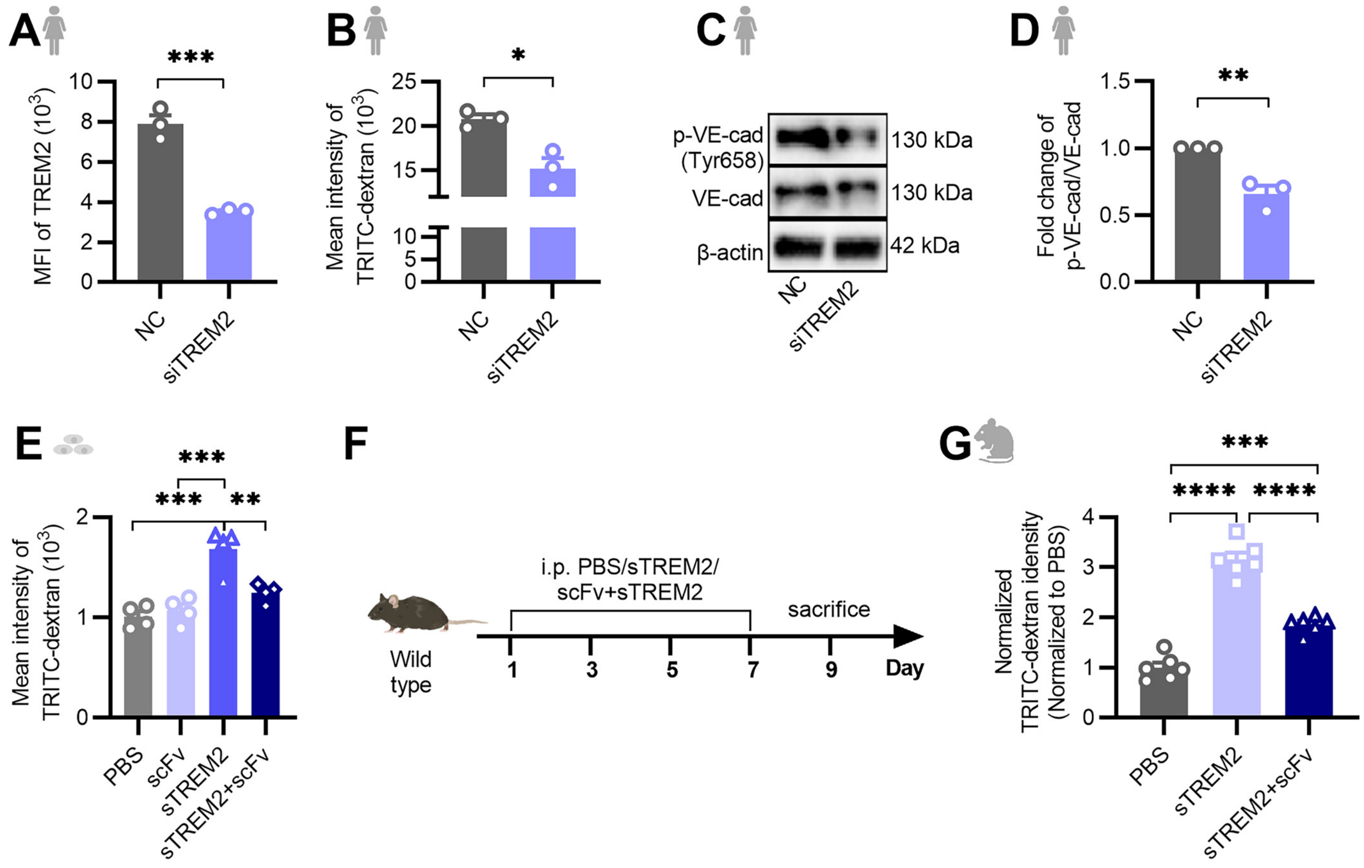

Figure EV5. Genetic and pharmacological targeting of TREM2 reduces vascular permeability.

Genetic and pharmacological targeting of TREM2 reduces vascular permeability (**A**) Flow cytometry analysis of TREM2 knockdown efficiency in ascites-derived macrophages (siTREM2 vs. negative control [NC]; $n = 3$ biological replicates). (**B**) TRITC-dextran permeability assay in HUVECs cocultured with ascites macrophages treated with siTREM2 or NC ($n = 3$ biological replicates). (**C**, **D**) Western blot analysis (**C**) and quantification (**D**) of p-VE-cadherin (Tyr658) in HUVECs cocultured with ascites macrophages with siTREM2 or NC ($n = 3$ biological replicates). β-actin was used as a loading control. (**E**) TRITC-dextran flux in C166 endothelial cells treated with sTREM2 ± anti-sTREM2 scFv ($n = 4$ biological replicates). (**F**) Schematic diagram of in vivo anti-sTREM2 scFv treatment protocol. (**G**) Peritoneal vascular permeability in mice intraperitoneally treated with sTREM2 (200 μg/dose) ± anti-sTREM2 scFv (200 μg/dose; $n = 6$ mice per group). Data were shown as mean ± SEM. $^*P < 0.05$; $^{**}P < 0.01$; $^{***}P < 0.001$; $^{****}P < 0.0001$. Exact *p* values are provided in Appendix Table S2. Two-tailed Student's *t*-test for (**A**, **B**, **D**); one-way ANOVA for (**E**, **G**). Source data are available online for this figure.

 © The Author(s)
