# Supplementary material for: Soluble TREM2 engages cell-surface nucleolin to drive vascular permeability and malignant ascites in ovarian cancer
Source: EMBO Mol Med. 2026 May 26;18(7):2667–90. doi: 10.1038/s44321-026-00452-2 (PMC13365401; doi:10.1038/s44321-026-00452-2)
Supplement: Supplementary file 2 — Peer Review File [file 44321_2026_452_MOESM2_ESM.pdf]

EMBO Molecular Medicine

Soluble TREM2 Engages Cell-surface Nucleolin to Drive Vascular Permeability and Malignant Ascites in Ovarian Cancer

Qianqian Li, Yubo Zhang, Hui Luo, Yongjian Wu, Jingbo Qin, Lijie Wang, Qianqian Zhang, Ting Yu, Xi Huang, Shuang Guo, Bingfan Xie, and Huanhuan He

Corresponding author(s): Huanhuan He (hehh23@mail.sysu.edu.cn) , Xi Huang (huangxi6@mail.sysu.edu.cn), Bingfan Xie (xiebf3@mail.sysu.edu.cn), Shuang Guo (guoshuang0317@hrbmu.edu.cn)

Review Timeline:

Submission Date:	10th Nov 25
Editorial Decision:	9th Dec 25
Revision Received:	23rd Mar 26
Editorial Decision:	18th Apr 26
Revision Received:	26th Apr 26
Accepted:	28th Apr 26

Editor: Jingyi Hou

Transaction Report:

(Note: With the exception of the correction of typographical or spelling errors that could be a source of ambiguity, letters and reports are not edited. Depending on transfer agreements, referee reports obtained elsewhere may or may not be included in this compilation. Referee reports are anonymous unless the Referee chooses to sign their reports.)

9th Dec 2025

Dear Prof. He,

Thank you again for submitting your work to EMBO Molecular Medicine. We have now received the reports from the three reviewers and as you will see below, the reviewers think that the study is potentially interesting. They raise however a series of concerns, which we would ask you to convincingly address in a revision.

I think the reviewers' recommendations are clear and need not be repeated here. All issues raised by the reviewers need to be satisfactorily addressed. As you may already know, our editorial policy allows in principle a single round of major revision so it is essential to provide responses to the referees' comments that are as complete as possible. Please feel free to contact me in case you would like to discuss in further detail any of the issues raised by the referees.

EMBO Molecular Medicine has a "scooping protection" policy, whereby similar findings that are published by others during review or revision are not a criterion for rejection. Should you decide to submit a revised version, I do ask that you get in touch after three months if you have not completed it, to update us on the status.

Please also contact us as soon as possible if similar work is published elsewhere. If other work is published, we may not be able to extend the revision period beyond three months.

Please read below for important editorial formatting and consult our author's guidelines for proper formatting of your revised article for EMBO Molecular Medicine.

I look forward to receiving your revised manuscript.

Yours sincerely,
Jingyi Hou

Jingyi Hou
Senior Editor
EMBO Molecular Medicine

*****
When preparing your revised manuscript, please refer to our guidelines: https://link.springer.com/journal/44321/submission-guidelines#cms-Revised-submissions. We perform an initial quality control of all revised manuscripts before re-review; failure to include requested items will delay the evaluation of your revision.

We require:

1) A .docx formatted version of the manuscript text (including legends for main figures, EV figures and tables). Please make sure that the changes are highlighted to be clearly visible.

2) Individual production quality figure files as .eps, .tif, .jpg (one file per figure). For guidance, download the 'Figure Guide PDF': https://media.springernature.com/original/springer-cms/rest/v1/content/27825798/data/v1.

3) A .docx formatted letter INCLUDING the reviewers' reports and your detailed point-by-point responses to their comments. As part of the EMBO Press transparent editorial process, the point-by-point response is part of the Review Process File (RPF), which will be published alongside your paper.

4) A complete author checklist, which you can download from our author guidelines. Please insert information in the checklist that is also reflected in the manuscript. The completed author checklist will also be part of the RPF.

5) Please note that all corresponding authors are required to supply an ORCID ID for their name upon submission of a revised manuscript.

6) It is mandatory to include a 'Data Availability' section after the Materials and Methods. Before submitting your revision, primary datasets produced in this study need to be deposited in an appropriate public database, and the accession numbers and

database listed under 'Data Availability'. Please remember to provide a reviewer password if the datasets are not yet public.

In case you have no data that requires deposition in a public database, please state so in this section. Note that the Data Availability Section is restricted to new primary data that are part of this study.

7) For data quantification: please specify the name of the statistical test used to generate error bars and P values, the number (n) of independent experiments (specify technical or biological replicates) underlying each data point and the test used to calculate p-values in each figure legend. The figure legends should contain a basic description of n, P and the test applied. Graphs must include a description of the bars and the error bars (s.d., s.e.m.).

8) At EMBO Press we ask authors to provide source data for the main manuscript figures. You will receive a separate email with instructions for providing source data with your revised manuscript, including how to upload and organize the files.

9) Our journal encourages inclusion of *data citations in the reference list* to directly cite datasets that were re-used and obtained from public databases. Data citations in the article text are distinct from normal bibliographical citations and should directly link to the database records from which the data can be accessed. In the main text, data citations are formatted as follows: "Data ref: Smith et al, 2001" or "Data ref: NCBI Sequence Read Archive PRJNA342805, 2017". In the Reference list, data citations must be labeled with "[DATASET]". A data reference must provide the database name, accession number/identifiers and a resolvable link to the landing page from which the data can be accessed at the end of the reference.

10) We replaced Supplementary Information with Expanded View (EV) Figures and Tables that are collapsible/expandable online. A maximum of 5 EV Figures can be typeset. EV Figures should be cited as 'Figure EV1, Figure EV2" etc... in the text and their respective legends should be included in the main text after the legends of regular figures.

- For the figures that you do NOT wish to display as Expanded View figures, they should be bundled together with their legends in a single PDF file called *Appendix*, which should start with a short Table of Content. Appendix figures should be referred to in the main text as: "Appendix Figure S1, Appendix Figure S2" etc.

- Additional Tables/Datasets should be labeled and referred to as Table EV1, Dataset EV1, etc. Legends have to be provided in a separate tab in case of .xls files. Alternatively, the legend can be supplied as a separate text file (README) and zipped together with the Table/Dataset file.

11) The paper explained: EMBO Molecular Medicine articles are accompanied by a summary of the articles to emphasize the major findings in the paper and their medical implications for the non-specialist reader. Please provide a draft summary of your article highlighting

- the medical issue you are addressing,

- the results obtained and

- their clinical impact.

This may be edited to ensure that readers understand the significance and context of the research. Please refer to any of our published articles for an example.

12) Author contributions: You will be asked to provide CRediT (Contributor Role Taxonomy) terms in the submission system. These replace a narrative author contribution section in the manuscript.

13) A Conflict of Interest statement should be provided in the main text.

14) Please provide a 'Synopsis' to further enhance discoverability. Synopses are displayed on the journal webpage and are freely accessible to all readers. They include a short stand first (maximum of 300 characters, including space) as well as 2-5 one-sentences bullet points that summarizes the paper. Please write the bullet points to summarize the key NEW findings. They should be designed to be complementary to the abstract - i.e. not repeat the same text. We encourage inclusion of key acronyms and quantitative information (maximum of 30 words / bullet point). Please use the passive voice. Please attach these in a separate file or send them by email, we will incorporate them accordingly.

Please also provide a visual abstract to illustrate your article as a PNG file 550 px wide x 300-600 px high.

15)All Materials and Methods need to be described in the main text using our 'Structured Methods' format. According to this format, the Methods section includes a Reagents and Tools Table (listing key reagents, experimental models, software and relevant equipment and including their sources and relevant identifiers) followed by a Methods and Protocols section describing the methods, ideally using a step-by-step protocol format. The aim is to facilitate adoption of the methodologies across labs.

Please download and fill our Reagents and Tools Table template (.docx), which you can find in our author guidelines. When submitting your revised manuscript, please do not include the Reagents and Tools Table in the Methods section of the manuscript but upload it as a separate file choosing the file type "Reagent Table".

EMBO Molecular Medicine has a "scooping protection" policy, whereby similar findings that are published by others during review or revision are not a criterion for rejection. Should you decide to submit a revised version, I do ask that you get in touch after three months if you have not completed it, to update us on the status.

***** Reviewer's comments *****

Referee #1 (Comments on Novelty/Model System for Author):

This study is advancing the understanding of TREM2 macrophage and sTREM in ovarian cancer, specifically pertaining to vascular leakage and malignant ascites. Cellular and molecular experiments are well done and of high quality. The discovery of sTREM2 acting through NCL to cause vascular leakage is a novel and interesting finding.

Referee #1 (Remarks for Author):

This study by Li et al is advancing the understanding of TREM2 macrophage and sTREM in ovarian cancer, specifically pertaining to vascular leakage and malignant ascites. Cellular and molecular experiments are well done and of high quality. The discovery that sTREM2 acting through NCL to cause vascular leakage is a novel and interesting finding.
There are a several minor concerns.
1. The scatter plot (Figure 1B) does not seem to support higher TREM2 expression in malignant ascites.
2. What is the control group in Figure 1C?
3. Given that the sTREM2-NCL axis is a novel discovery, would it be possible to substantiate this sTREM2-dependent NCL activation in patient tissue?
4. The scFv therapeutic effect of decreasing ascites is compelling (Figure 6I). What is the scFv's therapeutic action? Just depleting sTREM2? Any impact on TREM2+ macrophages? How does TREM2 signaling impact OC tumor cells?

Referee #2 (Comments on Novelty/Model System for Author):

Various model systems and experimental settings have been used without providing sufficient information on their generation, features, and criteria that supported their selection. This concern is detailed in the remarks to authors.

Referee #2 (Remarks for Author):

The manuscript reports the role of macrophage-derived soluble TREM2 (sTREM2) in ovarian cancer-associated ascites formation via the induction of vascular permeability. Mechanistically, sTREM2 acts via binding to nucleolin on endothelial surface and stimulating the Akt signaling pathway that, in turn, induces the phosphorylation of VE-cadherin which accounts for vessel permeability. The authors also show that a sTREM2-neutralizing antibody counteracts ascites and tumor development in a mouse model of ovarian cancer.
The manuscript is well written and easy to follow, with an experimental strategy that is suitable to address the scientific questions and the objectives of the study. In addition, the findings are interesting and add novel insights into the biology of ovarian cancer with regard in particular to ascites formation.
However, there are quite a few relevant issues that should be considered and addressed, and quite a body of additional information that should be included, in order to improve the quality of the manuscript and to support some of the authors' conclusions, thus making the study suitable for publication on EMBO Mol Med.

Major points:

1. The activation of Akt pathway upon binding of extracellular sTREM2 to endothelial cell-surface nucleolin is a key element of novelty in this study. Yet, how nucleolin engagement leads to Akt activation in endothelial cells remains unexplained. Is there any hint in this regard?

2. In all in vitro and in vivo experiments, the criteria for using certain numbers of cells and/or certain concentrations of sTREM2 are not specified. In particular, it is unclear whether these numbers and concentrations are consistent with the physiological levels found in real tumors. This is exemplified by the following experiments:
- Fig. S2I,J (number of macrophages?).
- Fig. 2A,B (number of THP1 and ratio with HUVEC?).
- Fig. 3A,D (where equal numbers of ID8 and RAW264.7 cells were co-injected, a ratio not really representative of the one

expected in a tumor).
- Fig. S2K,L (sTREM2 concentration?).
- Fig. S2E (is 500 ng/ml consistent with the concentration of sTREM2 in patients' ascites?).

3. Fig. 1C. Both TREM2 and CD68 show nuclear localization in many cells, while they are both supposed to be membrane-associated.

4. Fig. 2A-D. What is the level of ectopic sTREM2 released by THP1 cells? Along the same line, what evidence supports the actual depletion of released sTREM2 in Fig. 2M,N?

5. Fig. 2I. The role of VE-cadherin in enhancing endothelial barrier require tight cell-cell adhesion. However, the experiment here was performed on non-confluent HUVEC, which somehow questions the link of altered phospho-VE-cadherin with endothelial permeability , at least under these experimental conditions.

6. Fig. S2A and C. GFP is around 27kDa and TREM2 is around 28kDa (panel C). The TREM2-GFP shown in panel A is most likely a fusion protein between the two ("most likely" is mandatory here, as no information is provided on the construct, which raises another issue); how comes, then, that this fusion protein migrates as a 40-kDa protein (panel A) instead of ~55kDa?

7. RAW264.7 cells are used as a model for both overexpression and knockdown of TREM2, and it is unclear what is the endogenous level (Fig. S2A-D does not help in this regard).

8. Line 129 introduces "macrophages overexpressing sTREM2". However, these are not described anywhere in the manuscript: neither information on their generation nor evidence of the ectopic expression and release are provided.

9. Line 134 introduces "macrophage-specific TREM2 knockout mice". Again, no information on such an important model is provided. How was this conditional ko model generated? What is the evidence of macrophage specificity?

10. Fig. 4D is redundant with Fig. S4B, unless the authors give more details on the difference between the two assays.

11. Lines 183-184 and Fig. 4I. The RBD region is proposed as the binding site for sTREM2. Is this region exposed to the extracellular space in cell-surface nucleolin?

12. Fig. 6A,B. Among the three samples subjected to sTREM2 depletion, at least one shows residual sTREM2 even higher than the non-depleted samples. Yet, the reduction in the biological effect is similar among all three depleted samples (panel B). This would imply that the induction of permeability does not correlate with sTREM2 levels. How can this be explained?

Minor points:

1. Fig. 1B: Do the dots shown refer to statistically significant differential expression? For example, TREM2 in "AC" does not appear to be above 1.5 FC in spite of what is claimed in Methods (lines 353-354).

2. Line 106: Data in Fig. 1F cannot be used to infer a "positive correlation" but rather a general association of sTREM2 levels with ascites volume. Along the same line, why the authors did not perform a correlation analysis as done in Fig. 1G?

3. Lines 112-114. "This clinical finding was further corroborated by published proteomic data (19), which demonstrated a significant enrichment of sTREM2 in malignant ascites (Fig. S1E)." Actually, Fig. S1E only shows a Venn diagram with numbers, nothing to do with enrichment of anything. Either the authors provide details on how the differentially expressed proteins have been detected and quantified (together with the list of the protein themselves) or they should just mention that Ref. 19 reports the upregulation of sTREM2 in malignant ascites.

4. Line 421. Cell index is introduced here and shown in a few figures as a parameter. Yet, no explanation is given on what it is and how it relates to endothelial permeability?

5. Figs. 4D,E. What is "anti-IgG"? The authors probably mean simply "IgG".

6. Fig. S5G. The difference between the first bars on the left (without sTREM2) seems statistically significant, is that the case?

Referee #3 (Comments on Novelty/Model System for Author):

Please refer to the comments to author below.

Referee #3 (Remarks for Author):

This is a well performed study supporting the macrophage-produced soluble TREM2 (sTREM2) as a key factor to engage cell-surface Nucleolin (NCL) to drive vascular permeability and malignant ascites in ovarian cancer by using the sTREM2-NCL-AKT-eNOS signaling axis in endothelial cells. I only have several minor points:

Fig. 1B is supposed to show TREM2 was significantly upregulated in primary tumors, metastatic lesions, and malignant ascites compared to normal ovarian tissues. But it is unclear what are really shown in this figure panel and there is also no statistics provided to support the claim of "significantly upregulated".

Fig. 1F shows TREM2 expression levels have a significant positive correlation with ascites volume and Fig. 1H further shows the same correlation for sTREM2. What is the level of macrophages, particularly TREM2+ macrophages in malignant ascites? Is the positive correlation of sTREM2 level with ascites volume simply due to higher numbers of TREM2+ macrophages in larger volumes of ascites?

The authors described their results in a very succinct way, but sometimes it appears to lack sufficient details for readers to fully understand their experiments: for instance, the in vitro coculture system consisting of endothelial cells and macrophages with differentially expressed TREM2: it seems the authors used two cocultures, human HUVECs cocultured with THP-1-derived macrophage in Fig. 2, as well as murine C166 endothelial cells cocultured with RAW264.7 macrophages in Fig. S2. In Methods section, under "Transfection and establishment of stable cell lines", the authors described how they modulated TREM2 levels in RAW264.7 cell lines, but not THP-1 cells.

The authors need to provide more detail for Trem2MacKO mice. Assuming this is a macrophage-specific conditional knockout model for Trem2, is Trem2 allele here a floxed conditional KO allele? If so, is there a reference for it? What is the macrophage-specific Cre line to knock it out? If it's not a published model, the authors should provide more detail about the mouse line and also some data to demonstrate the macrophage-specific loss of Trem2.

In Fig. 3, the authors studied macrophage-specific TREM2 function in vivo by co-injecting ID8 cells and RAW264.7 macrophages (either TREM2-overexpressing, knockdown or control) to C57BL/6 female mice. The ID8 cell line is under the same C57BL/6 background, but the RAW264.7 macrophage cell line is under the BALB/c background (based on ATCC). How could injection of RAW264.7 cells into C57BL/6 mice not induce any immune rejection?

POINT-BY-POINT RESPONSE TO REVIEWERS

We would like to express our gratitude to the reviewers and editors for their constructive comments and criticisms, which have contributed to the enhancement of the study. In response, the author team has meticulously conducted a series of experiments to address the points raised. This process has resulted in the incorporation of 15 new or revised panels in main figures and 21 new or revised panels in the supplementary figures, indicated below in red. The detailed point-by-point responses are outlined below in blue. In addition, to comply with journal formatting requirements, supplementary materials have been reorganized and renamed.

Reviewer #1:

This study by Li et al is advancing the understanding of TREM2 macrophage and sTREM in ovarian cancer, specifically pertaining to vascular leakage and malignant ascites. Cellular and molecular experiments are well done and of high quality. The discovery that sTREM2 acting through NCL to cause vascular leakage is a novel and interesting finding.

There are a several minor concerns.

1.The scatter plot (Figure 1B) does not seem to support higher TREM2 expression in malignant ascites.

Response: Thank you for your valuable feedback. We fully agree that the original scatter plot (Fig. 1B) did not adequately visualize the elevated expression of TREM2 in malignant ascites. In response, we have replaced it with a violin plot (revised Figure 1B), which more clearly illustrates the data distribution and facilitates group comparisons. Statistical significance of the differences in TREM2 expression between groups is now explicitly indicated with asterisks (*).

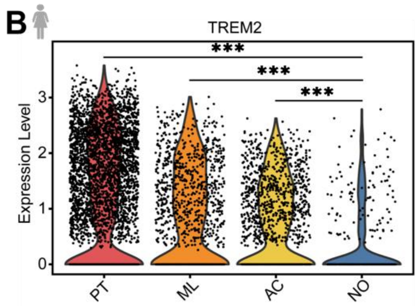

Figure 1B. Differential expression of TREM2 in tumor-associated macrophages from primary tumors (PT), omental metastatic lesions (ML), and malignant ascites (AC) compared with normal ovarian tissue (violin plot; dataset: HRA002362). Wilcoxon Rank-Sum test for B, ****P* < 0.001.

2.What is the control group in Figure 1C?

Response: Thank you for raising this important point. In Fig. 1C, the control group

included female patients aged ⩾18 years who underwent gynecologic surgery for non-ovarian malignant indications, such as cervical cancer, endometrial cancer, severe endometriosis, benign ovarian cysts, uterine prolapse, refractory dysfunctional uterine bleeding, or prophylactic surgery in high-risk individuals. Control ovarian tissues were obtained during surgery and were confirmed to be free of malignant or premalignant lesions by postoperative histopathological examination when available, or were considered macroscopically normal by the operating surgeons. These details are now incorporated into the revised **Methods section** (Page 17 Line 403-411).

3.Given that the sTREM2-NCL axis is a novel discovery, would it be possible to substantiate this sTREM2-dependent NCL activation in patient tissue?

Response: This is indeed an insightful point. In response, we have conducted some preliminary exploration. By immunofluorescence staining of paraffin sections from patient tissues, we assessed the level of p-eNOS—a key downstream signaling molecule of the NCL pathway—in endothelium (marked by CD31). The results showed a significant increase in p-eNOS signal in ovarian cancer samples compared to controls (revised Figure EV4I, J), consistent with our in vitro and in vivo observations. These data provide further evidence that sTREM2 may activate the Akt/eNOS pathway via NCL, thereby contributing to malignant ascites formation.

At the same time, we acknowledge that this experiment does not provide direct evidence of physical binding or functional dependence between sTREM2 and NCL in patient tissues. Directly validating this interaction is challenging due to limited patient tissue availability and technical limitations (e.g., capturing transient interactions in fixed tissues). We have now addressed this limitation in the revised **Discussion section** (Page 13 Line 317-322).

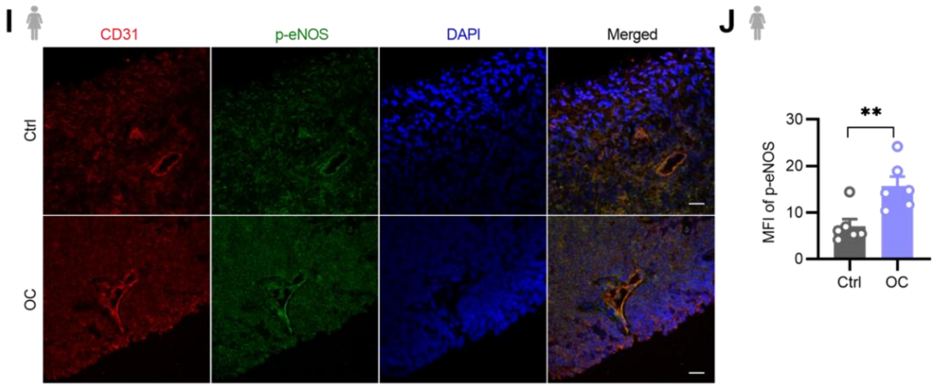

Figure EV4I, J. Immunofluorescence analysis (I) and quantification (J) of p-eNOS (green) expression in vasculature (CD31, red) within human OC tissues (n = 6 patients). Nuclei were counterstained with DAPI (blue). MFI stands for mean fluorescence intensity. Scale bar: 20 μm. Data are shown as mean ± SEM. $^{**}P < 0.01$, two-tailed Student's t-test for J.

4. The scFv therapeutic effect of decreasing ascites is compelling (Figure 6I). What is the scFv's therapeutic action? Just depleting sTREM2? Any impact on TREM2+ macrophages? How does TREM2 signaling impact OC tumor cells?

Response: Thank you for these critical questions, which prompted us to explore the therapeutic mechanism and potential impacts of the scFv in greater depth.

Our scFv targets the extracellular domain of TREM2 and its primary therapeutic mechanism involves neutralizing sTREM2 to block the sTREM2–NCL signaling axis. At the same time, we assessed its potential effects on TREM2+ macrophages through the following experiments: 1) treatment of macrophages (iBMDMs) with the scFv did not significantly affect cell proliferation, as determined by CCK-8 assay (RR Figure 1A); 2) qPCR analysis showed no notable changes in the expression of M1 (CD80, CD86) and M2 (ARG1, CD206) polarization markers, or in VEGF expression, following scFv treatment (RR Figure 1B). These preliminary data suggest that, under the tested conditions, scFv may not directly alter the fundamental state of macrophages.

Regarding how TREM2 signaling affects ovarian cancer (OC) cells, our preliminary data show that recombinant sTREM2 directly stimulates ID8 cell invasion and migration (RR Figure 1C, D), consistent with its reported effect on breast cancer cells (Ref. 1). Together, these data suggest that the tumor-promoting effect of sTREM2 may exist across multiple tumor types.

Combined with our other findings, sTREM2 may promote OC progression via two mechanisms: activating the endothelial NCL/Akt/eNOS pathway to increase vascular permeability and ascites formation, and enhancing tumor cell malignancy. Future studies will dissect the relative contributions of these mechanisms.

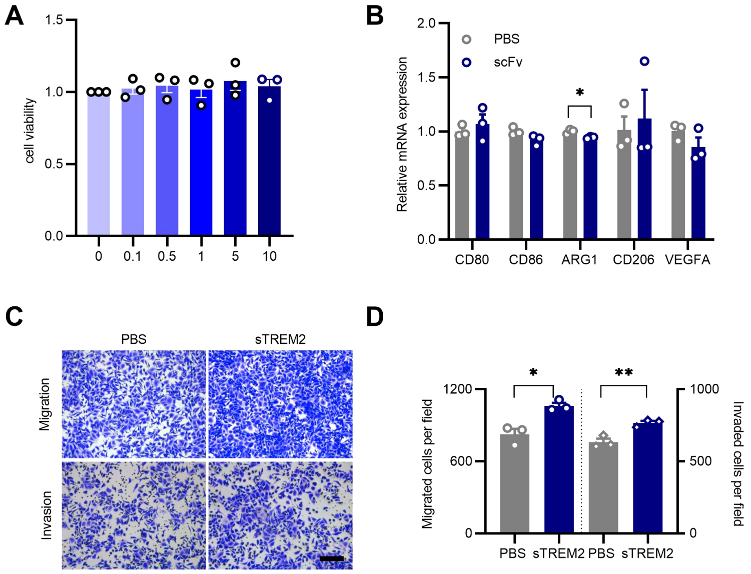

RR Figure 1. Effects of scFv and sTREM2 on macrophages and ovarian cancer cells respectively. (A) CCK-8 assay assessing the effect of scFv treatment on iBMDM cell viability (n = 3 biological replicates). (B) qPCR analysis of the effect of scFv treatment on iBMDM polarization (n = 3 biological replicates). (C, D) Representative images (C) and quantification (D) of migrated and invaded ID8 cells stimulated with sTREM2 (n = 3 biological replicates). Data are shown as mean ± SEM. *$P < 0.05$, **$P < 0.01$; one-way ANOVA analysis for A; two-tailed Student's t-test for B and D.

Referee #2:

The manuscript reports the role of macrophage-derived soluble TREM2 (sTREM2) in ovarian cancer-associated ascites formation via the induction of vascular permeability. Mechanistically, sTREM2 acts via binding to nucleolin on endothelial surface and stimulating the Akt signaling pathway that, in turn, induces the phosphorylation of VE-cadherin which accounts for vessel permeability. The authors also show that a sTREM2-neutralizing antibody counteracts ascites and tumor development in a mouse model of ovarian cancer.

The manuscript is well written and easy to follow, with an experimental strategy that is suitable to address the scientific questions and the objectives of the study. In addition, the findings are interesting and add novel insights into the biology of ovarian cancer with regard in particular to ascites formation.

However, there are quite a few relevant issues that should be considered and addressed, and quite a body of additional information that should be included, in order to improve the quality of the manuscript and to support some of the authors' conclusions, thus making the study suitable for publication on EMBO Mol Med.

Major points:

1. The activation of Akt pathway upon binding of extracellular sTREM2 to endothelial cell-surface nucleolin is a key element of novelty in this study. Yet, how nucleolin engagement leads to Akt activation in endothelial cells remains unexplained. Is there any hint in this regard?

Response: We thank the reviewer for raising this important mechanistic question. Through literature review and preliminary explorations, we have developed several hypotheses, though definitive conclusions remain to be established.

According to existing literature, multiple potential mechanisms may underlie NCL-mediated Akt activation. For instance, in nasopharyngeal carcinoma, NCL has been shown to directly interact with Akt and promote its phosphorylation (Ref. 2). In prostate cancer, modified NCL can enhance binding to Akt mRNA, thereby promoting Akt protein synthesis (Ref. 3). Additionally, in lung cancer, NCL may indirectly influence the PI3K/Akt pathway by regulating upstream receptors such as ITGB1(Ref. 4).

Building on these reports, we first examined whether sTREM2 enhances the physical interaction between NCL and Akt in HUVECs. As shown in RR Figure 2A, B, while we confirmed a direct protein-protein interaction between NCL and Akt, stimulation with sTREM2 did not significantly strengthen this binding. This result suggests that Akt activation via the sTREM2–NCL axis is unlikely to occur through simple recruitment of additional Akt molecules but rather involves a more sophisticated mechanism. We speculate that sTREM2 binding may induce conformational changes, post-translational modifications, or altered membrane microdomain localization of NCL. Such changes could modulate the functional state of NCL complexes with Akt signaling components—for example, by enhancing the phosphorylation efficiency of Akt by proximal kinases such as PDK1 or mTORC2—or facilitate interactions between NCL and other upstream signaling molecules (e.g.,

PI3K). These possibilities warrant further investigation.

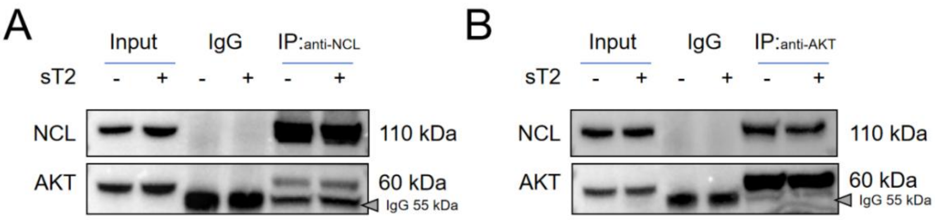

RR Figure 2. Co-immunoprecipitation (co-IP) analysis of the NCL-Akt interaction. (A) Forward co-IP: IP with anti-NCL antibody followed by immunoblotting for Akt. (B) Reverse co-IP: IP with anti-Akt antibody followed by immunoblotting for NCL.

2. In all in vitro and in vivo experiments, the criteria for using certain numbers of cells and/or certain concentrations of sTREM2 are not specified. In particular, it is unclear whether these numbers and concentrations are consistent with the physiological levels found in real tumors. This is exemplified by the following experiments:

- Fig. S2I,J (number of macrophages?).

- Fig. 2A,B (number of THP1 and ratio with HUVEC?).

- Fig. 3A,D (where equal numbers of ID8 and RAW264.7 cells were co-injected, a ratio not really representative of the one expected in a tumor).

- Fig. S2K,L (sTREM2 concentration?).

- Fig. S2E (is 500 ng/ml consistent with the concentration of sTREM2 in patients' ascites?).

Response: Thank you for raising these detailed and important questions. We have incorporated the relevant details into the revised **Methods and Figure Legends sections**, and have provided a consolidated explanation below.

1） The number of macrophages (1×10^6 per mouse) was selected based on previously published studies (Ref. 5, 6), in which this cell dosage was used to evaluate macrophage-mediated regulation of vascular permeability. This number has been shown to induce measurable functional effects without causing overt toxicity in mice. We therefore adopted this established experimental parameter to ensure reproducibility and comparability with prior reports.

2） In Fig. 2A, HUVECs (1×10^5 cells/insert) were seeded in the upper chamber of a 24-well Transwell plate and allowed to form a confluent monolayer. THP-1-derived macrophages (5×10^4 cells/well) were then added to the lower chamber, resulting in a 2:1 endothelial-to-macrophage ratio. In Fig. 2B, HUVECs were seeded into E-plates at 2×10^4 cells per well (adjusted for the smaller effective growth area, comparable to a 96-well format) and cultured to form a confluent monolayer. Subsequently, THP-1-derived macrophages (1×10^4 cells/well) were added in E-plate inserts, maintaining the same 2:1 endothelial-to-macrophage ratio. The selected cell numbers ensured stable endothelial monolayer formation while allowing sufficient exposure of HUVECs to macrophage-derived sTREM2.

3） Regarding the use of a 1:1 ratio of ID8 tumor cells to macrophages in the in vivo experiments (Fig. 3A, D), we acknowledge that this represents a simplified model of the complex tumor microenvironment. Published single-cell sequencing data indicate that the macrophage-to-tumor cell ratio in ovarian cancer ascites varies widely among patients, ranging from approximately 0.07 in patient P14 to 4.87 in patient P15 (Ref. 7; RR Figure 3A, B), making it difficult to define a "representative" ratio. Our 1:1 design thus provides a balanced and reproducible starting point to establish the functional importance of macrophages and (s)TREM2 specifically in this setting. Future studies would benefit from validating these findings in models that more closely reflect the physiological cellular composition.

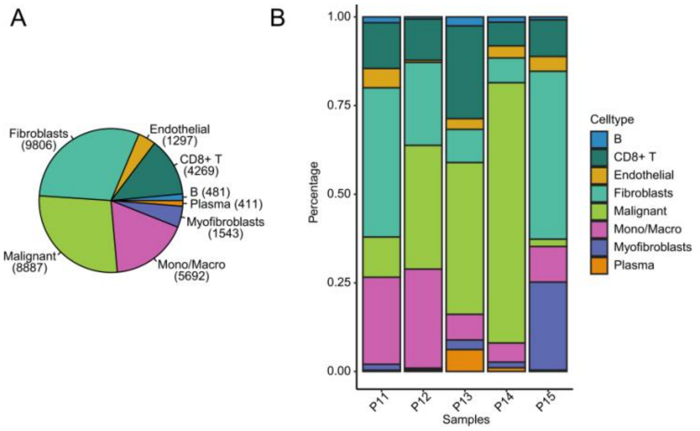

RR Figure 3. Cellular composition of ovarian tumor samples from the EMTAB8107 dataset. (A) Overall distribution of major cell populations identified by single-cell RNA sequencing. Numbers in parentheses indicate total cell counts for each cell type. (B) Relative proportions of each cell population across individual patient samples (P11–P15), presented as stacked bar plots. Percentages represent the fraction of each cell type within the corresponding sample.

4） The sTREM2 concentration used in Fig. S2K, L is 500 ng/mL. This working concentration was selected based on three considerations. First, dose–response experiments showed that 500 ng/mL induces a robust and reproducible endothelial phenotype, providing a reliable window for mechanistic investigation (RR Figure 4A, B). Second, while the average sTREM2 level in patient ascites is lower, concentrations can approach 500 ng/mL (median: 136.6 ng/mL; maximum: 441.4 ng/mL; n=30; RR Figure 4C). Third, this concentration aligns with the concentration used in previous sTREM2 functional studies (500 ng/mL ≈ 26 nM, comparable to the ~20–25 nM commonly reported; Ref. 1, 8, 9).

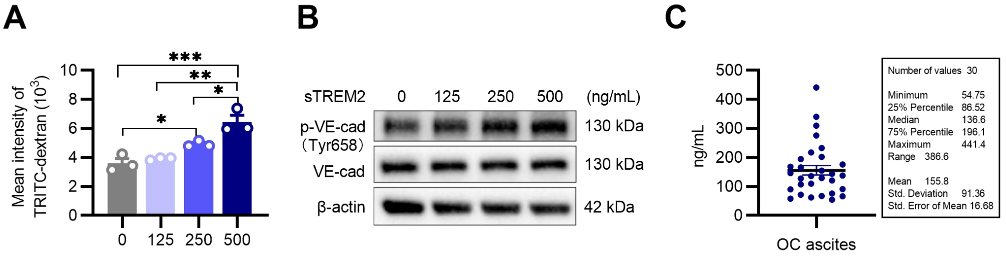

RR Figure 4. (A) TRITC-dextran permeability assay showing endothelial permeability in HUVEC monolayers treated with various concentrations of sTREM2 (n = 3 biological

replicates). (B) Western blot analysis of p-VE-cadherin (Tyr658) and total VE-cadherin in HUVECs incubated with sTREM2. (C) sTREM2 concentrations detected in ascites of ovarian cancer patients (n=30 patients).Data are mean ± SEM.**P* < 0.05, ***P* < 0.01, ****P* < 0.001, one-way ANOVA for A.

3. Fig. 1C. Both TREM2 and CD68 show nuclear localization in many cells, while they are both supposed to be membrane-associated.

Response: We thank the reviewer for this helpful observation. We have re-examined the high-resolution original images from Fig. 1C and reassessed the localization patterns. Upon detailed inspection, the apparent "nuclear" signals likely arise from the partial overlap—in two-dimensional projections—of cytoplasmic or membrane signals located above or below the nuclear plane with DAPI-stained nuclei, a common artifact in fluorescence imaging of thicker tissue sections. To clarify, we now provide magnified views of the representative regions in the original image (RR Figure 5). These higher-magnification views more clearly demonstrate that TREM2 and CD68 signals are predominantly localized to membrane and cytoplasmic regions surrounding the nuclei, consistent with their known roles as membrane-associated proteins.

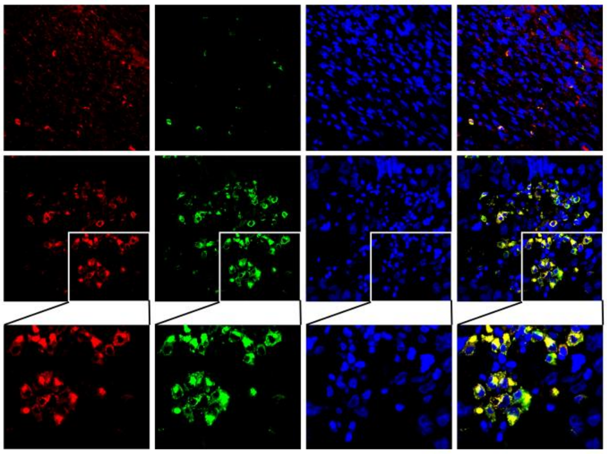

RR Figure 5. Magnified views of representative regions from the original Fig. 1C showing immunofluorescence staining of TREM2 (red) and CD68 (green) in human ovarian cancer tissues. Nuclei were counterstained with DAPI (blue). The enlarged images highlight the cytoplasmic and membrane localization of TREM2 and CD68 surrounding the nuclei.

4. Fig. 2A-D. What is the level of ectopic sTREM2 released by THP1 cells? Along the same line, what evidence supports the actual depletion of released sTREM2 in Fig. 2M,N?

Response: We thank the reviewer for raising these important questions. In response, we have now included additional validation data as summarized below.

First, regarding the generation and validation of sTREM2-overexpressing macrophages, a detailed description is now provided in the **Methods section** (Page 19 Line 455-461). Successful establishment of this cell line was confirmed by two approaches: 1) confocal microscopy verified the red fluorescence in the vast majority of cells; and 2) ELISA of conditioned medium (collected from 1×10^5 cells seeded in a

24-well plate with 500 μL medium for 24 hours) confirmed high levels of sTREM2 release (revised Figure EV2I).

Second, for the depletion experiment in Fig. 2M, N, we have supplemented with ELISA data showing that immunoprecipitation-mediated depletion using an anti-sTREM2 antibody effectively removed sTREM2 from the conditioned medium (revised Figure EV2K). All details regarding cell line establishment and depletion validation have been integrated into the **Methods and Results sections**.

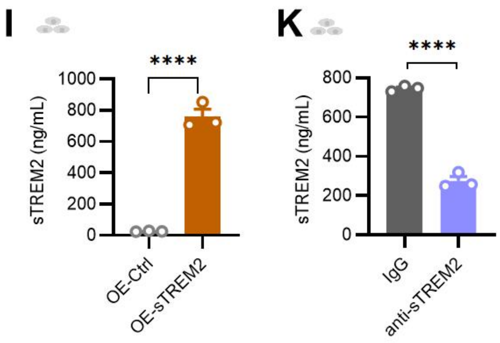

Figure EV2. (I) sTREM2 levels in the culture supernatants of THP-1 cells, detected by ELISA (n = 3 biological replicates). (K) sTREM2 levels in conditioned medium from sTREM2-overexpressing THP-1 cells before and after immunoprecipitation-mediated depletion with an anti-sTREM2 antibody, detected by ELISA (n = 3 biological replicates). Data are mean ± SEM. ****$P < 0.0001$, two-tailed Student's t-test for I and K.

5. Fig. 2I. The role of VE-cadherin in enhancing endothelial barrier require tight cell-cell adhesion. However, the experiment here was performed on non-confluent HUVEC, which somehow questions the link of altered phospho-VE-cadherin with endothelial permeability, at least under these experimental conditions.

Response: We thank the reviewer for this important comment. In response, we have confirmed that all permeability assays were performed under confluent conditions and have repeated key immunofluorescence experiments to further validate our findings.

First, we confirm that all functional permeability assays were performed under confluent conditions. For the TRITC-dextran permeability assay, HUVECs (1×10^5 cells) were seeded in 24-well Transwell inserts and cultured for 24 h to allow formation of a continuous monolayer prior to sTREM2 stimulation. Similarly, for real-time impedance (RTCA) measurements, experiments were initiated only after the Cell Index reached a stable plateau, indicating the establishment of an intact endothelial layer.

Second, to further address the concern, we have repeated the immunofluorescence experiments under fully confluent conditions. The new data confirm that sTREM2 increases VE-cadherin phosphorylation at endothelial cell-cell junctions in confluent monolayers (revised Fig. 2I, J). Moreover, pharmacological inhibition of eNOS with L-NAME significantly attenuated this effect, further supporting the involvement of the AKT-eNOS signaling pathway (revised Fig. 5K, L).

Together, these results support the conclusion that sTREM2-induced signaling promotes VE-cadherin phosphorylation at endothelial junctions and contributes to

endothelial barrier dysfunction.

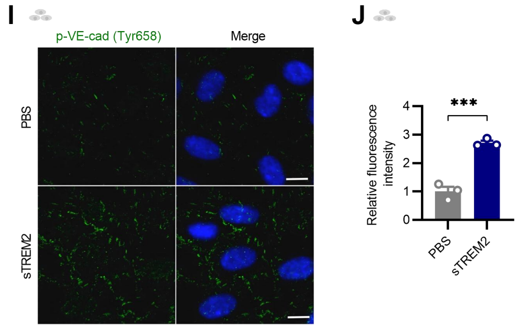

Figure 2I, J. Immunofluorescence analysis (I) and quantification (J) of p-VE-cadherin (Tyr658; green) in HUVECs treated with sTREM2 (500 ng/mL); nuclei were stained with DAPI (blue). Scale bar: 10 μm (n = 3 biological replicates). Data are shown as mean ± SEM. ****P* < 0.001, two-tailed Student's t-test for J.

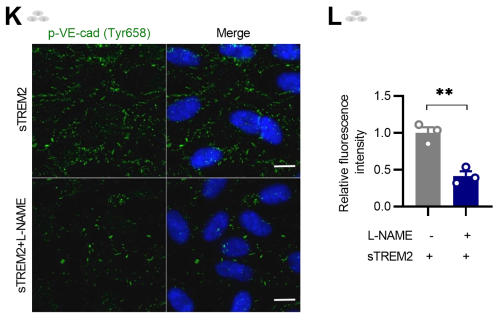

Figure 5K, L. Immunofluorescence analysis (K) and quantification (L) of p-VE-cadherin (Tyr658) (green) in HUVECs incubated with 1 mM eNOS inhibitor L-NAME prior to treatment with 500 ng/mL sTREM2; nuclei were counterstained with DAPI (blue). Scale bar: 10 μm (n = 3 biological replicates). Data are shown as mean ± SEM. ***P* < 0.01, two-tailed Student's t-test for L.

6. Fig. S2A and C. GFP is around 27kDa and TREM2 is around 28kDa (panel C). The TREM2-GFP shown in panel A is most likely a fusion protein between the two ("most likely" is mandatory here, as no information is provided on the construct, which raises another issue); how comes, then, that this fusion protein migrates as a 40-kDa protein (panel A) instead of ~55kDa?

Response: We thank the reviewer for raising this important concern. In response, we have corrected the figure labeling, clarified the construct details, and included additional reference data to support our interpretation.

We acknowledge that the labeling in Fig. S2A was incorrect in the original manuscript. The construct used in is not a TREM2–GFP fusion protein, but a TREM2–3×FLAG construct, as indicated in the plasmid design (Ubi-MCS-3FLAG-CBh-gcGFP-IRES-puromycin), which is now clearly described in Appendix Table S3. The figure labeling has been corrected accordingly.

The predicted molecular weight of the unmodified TREM2–3×FLAG fusion protein is approximately 30–31 kDa (TREM2 core: ~27.5 kDa; 3×FLAG tag: ~2.6 kDa). The observed migration at ~35–40 kDa under reducing SDS–PAGE conditions is

consistent with the known biochemical properties of TREM2. TREM2 is a type I transmembrane glycoprotein that undergoes extensive N-linked glycosylation, resulting in an increased apparent molecular weight during electrophoresis. Consistently, previous studies have reported that glycosylated TREM2 migrates between ~30–40 kDa in Western blot analyses (Ref. 10, 11).

For transparency, we provide the uncropped Western blot image corresponding to Fig. S2A (RR Figure 6A). In addition, to illustrate the characteristic migration pattern of endogenous TREM2 and the size shift following deglycosylation, we include representative antibody validation data from a manufacturer (*Cell Signaling Technology*), showing Western blot analyses of endogenous TREM2 before and after treatment with PNGase F (RR Figure 6B). These reference data demonstrate the typical electrophoretic migration pattern of glycosylated TREM2 and its downward shift upon removal of N-linked glycans, supporting the interpretation of the band observed in Fig. S2A.

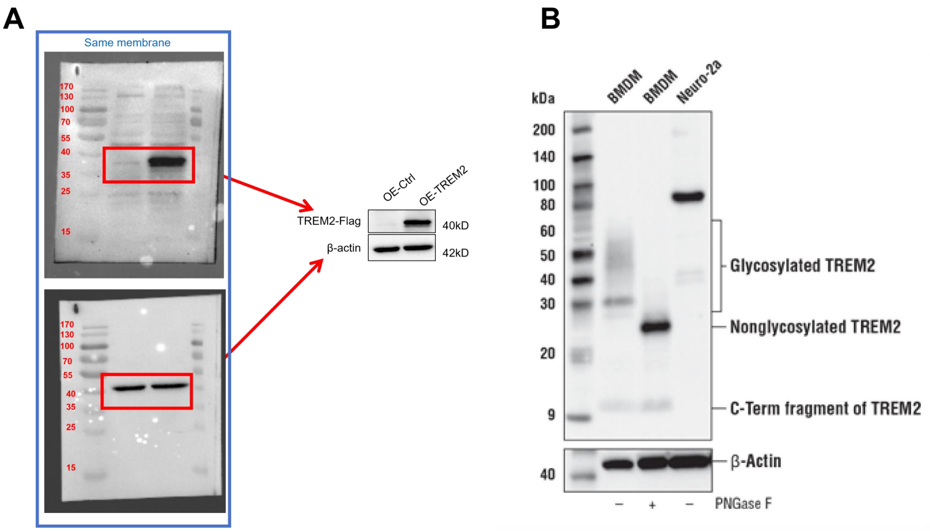

RR Figure 6. Validation of the molecular weight of TREM2 in Western blot analysis. (A) Uncropped Western blot image corresponding to Fig. S2A, showing expression of the TREM2-3×FLAG construct in transfected cells. (B) Representative Western blot validation data obtained with the anti-TREM2 antibody E7P8J from *Cell Signaling Technology*. Cell lysates from mouse bone marrow–derived macrophages (BMDMs), untreated (−) or treated with the deglycosylation enzyme PNGase F (+), and Neuro-2a cells were probed with anti-TREM2 antibody (CST, cat# 76765).

7. RAW264.7 cells are used as a model for both overexpression and knockdown of TREM2, and it is unclear what is the endogenous level (Fig. S2A-D does not help in this regard).

Response: Thank you for raising this important point. To address this, we performed additional Western blot analyses to assess endogenous TREM2 protein levels across the murine cell lines used in this study (C166, ID8, RAW264.7, and iBMDM).

The results show that TREM2 is nearly undetectable in ID8 and C166 cells. RAW264.7 macrophages exhibit low but detectable endogenous TREM2 expression, supporting the feasibility of both knockdown and overexpression approaches. As

expected, BMDM display markedly higher endogenous TREM2 levels (RR Figure 7A). These data clarify the baseline expression levels and support the validity of the genetic manipulation strategies used in RAW264.7 cells.

Importantly, key experiments were repeated in iBMDM and yielded results consistent with those obtained in RAW264.7 cells (Figure EV2A-D, H, Figure 3A-F, and Figure EV3A-F), thereby further supporting the robustness and generalizability of our findings.

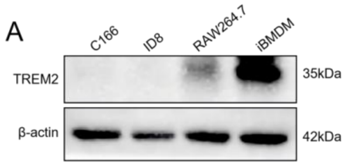

RR Figure 7A. Endogenous TREM2 protein expression in murine cell lines. Western blot analysis of TREM2 expression in whole-cell lysates from C166, ID8, RAW264.7, and iBMDM, β-actin was used as a loading control.

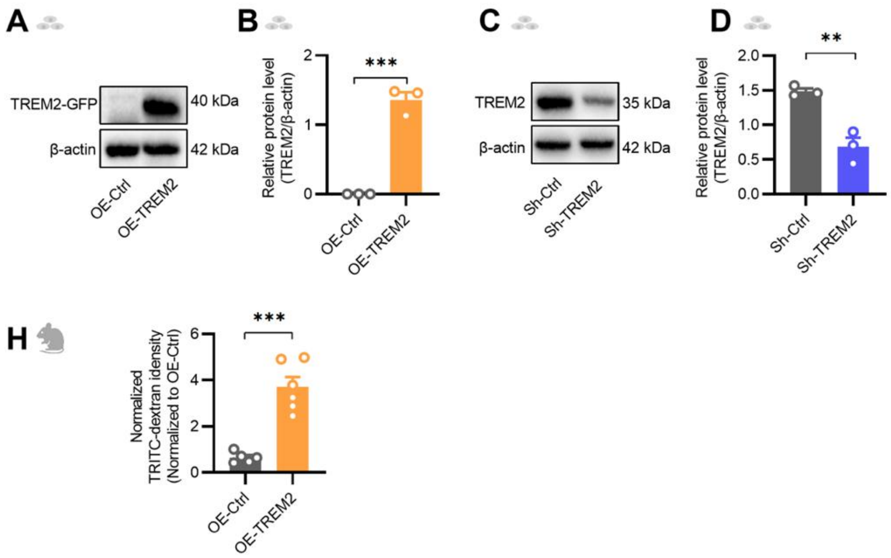

Figure EV2. (A, B) Western blot analysis (A) and quantification (B) of TREM2 expression in iBMDM macrophages overexpressing TREM2 (OE-TREM2) versus empty vector control (OE-Ctrl); β-actin as loading control (n = 3 biological replicates). (C, D) Western blot analysis (C) and quantification (D) of TREM2 expression in iBMDM macrophages with TREM2 knockdown (Sh-TREM2) versus scramble control (Sh-Ctrl); β-actin as loading control (n = 3 biological replicates). (H) Peritoneal vascular permeability in mice injected intraperitoneally with 1 × 10^6 TREM2-overexpressing iBMDM (n = 5 mice) versus control iBMDM cells (n = 6 mice). Data are mean ± SEM. $^{**}P < 0.01$, $^{***}P < 0.001$, two-tailed Student's t-test for B, D and H.

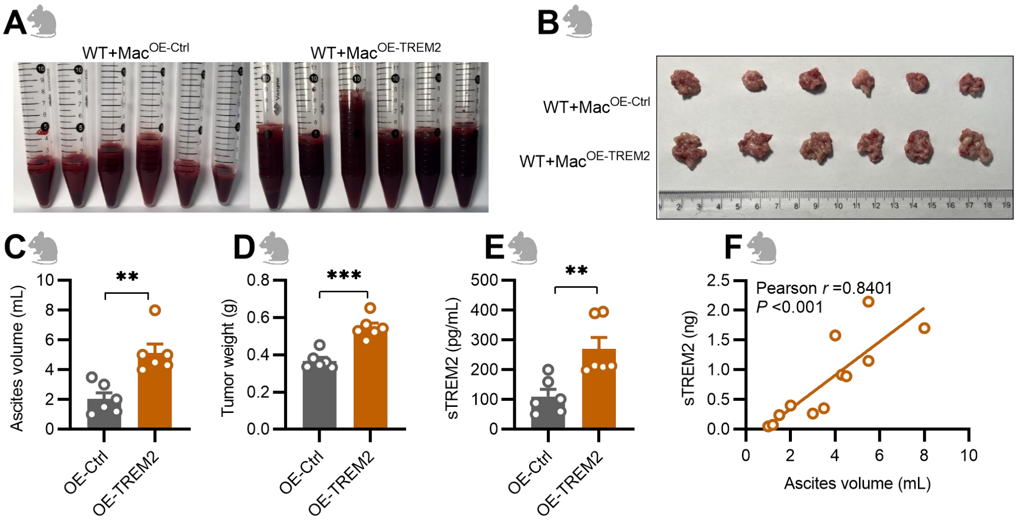

Figure 3. (A-D) Representative images and quantification of ascites volume (A, C) and tumor burden (B, D) in mice following intraperitoneal injection of OE-TREM2 or OE-Ctrl iBMDM in combination with ID8 ovarian cancer cells (n = 6 mice). (E) ELISA measurement of sTREM2 levels in ascites supernatant from (A). (F) Correlation analysis between ascites volume and sTREM2 levels from (E). Data are shown as mean ± SEM. $^{**}P < 0.01$; $^{***}P < 0.001$; two-tailed Student's t-test for C-E, Pearson correlation analysis for F.

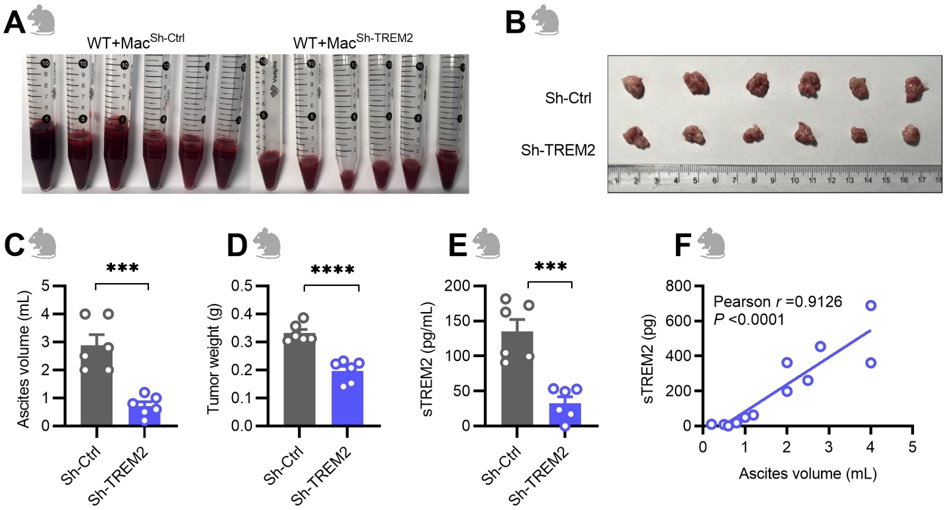

Figure EV3. TREM2 knockdown inhibits malignant ascites formation in ovarian cancer. (A, C) Representative images (A) and quantification (C) of ascites in mice injected intraperitoneally with Sh-TREM2 iBMDM cells and ID8 ovarian cancer cells versus Sh-Ctrl iBMDM and ID8 cells (n = 6 mice). (B, D) Intraperitoneal tumor formation (B) and quantification (D) in the same experimental groups as (A) (n = 6 mice). (E) ELISA measurement of sTREM2 levels in ascites supernatant from (A). (F) Correlation analysis between ascites volume and sTREM2 concentration from (E). Data are shown as mean ± SEM. $^{**}P < 0.01$, $^{***}P < 0.001$; $^{****}P < 0.0001$, two-tailed Student's t-test for C-E, Pearson correlation analysis for F.

8. Line 129 introduces "macrophages overexpressing sTREM2". However, these are not described anywhere in the manuscript: neither information on their generation nor evidence of the ectopic expression and release are provided.

Response: We thank the reviewer for pointing out this omission. As described in our response to Comment #4, we have now provided a detailed description of the generation and validation of the sTREM2-overexpressing THP-1 macrophages in the **Methods section** (Page 19 Line 455-461). Briefly, THP-1 cells were transduced with a lentiviral vector encoding human sTREM2 fused with an HA tag and mScarlet reporter, followed by blasticidin selection to establish a stable cell line. Successful overexpression and secretion of sTREM2 were confirmed by confocal imaging of mScarlet fluorescence and ELISA quantification of sTREM2 in conditioned media.

9. Line 134 introduces "macrophage-specific TREM2 knockout mice". Again, no information on such an important model is provided. How was this conditional ko model generated? What is the evidence of macrophage specificity?

Response: Thank you for raising this important point regarding the animal model. This information has now been comprehensively addressed in the revised version.

The mice used are more accurately described as myeloid cell-specific *Trem2* conditional knockout mice. The construction and validation of this model have been described in detail previously (Ref. 12). Briefly, the model was generated by crossing mice carrying a floxed *Trem2* allele ($Trem2^{fl/fl}$) with $Lyz2^{Cre}$ transgenic mice, in which Cre recombinase expression is driven by the lysozyme M promoter to achieve specific deletion in myeloid cells, including macrophages.

To directly confirm the model's validity in our study, we have supplemented the manuscript with the following evidence: 1) genotyping results confirming that the mice used were of the $Trem2^{fl/fl}Lyz2^{Cre}$ genotype (RR Figure 8); and 2) Western blot analysis demonstrating effective *Trem2* knockout in bone marrow-derived macrophages (revised Figure EV2J). All related information has been integrated into the **Methods** (Page 26 Line 628-633) **and Results sections.**

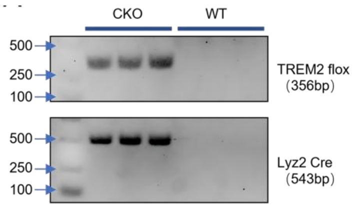

RR Figure 8. Identification of gene knockout mice. The gene phenotypes of $Trem2^{fl/fl}Lyz2^{Cre}$ mice were respectively identified by PCR.

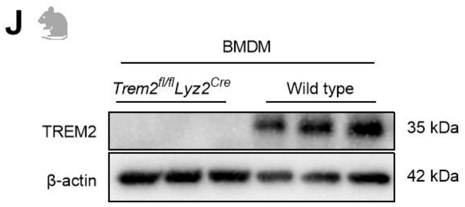

Figure EV2J. Western blot validation of TREM2 knockout in bone marrow-derived macrophages from *Trem2*$^{fl/fl}$*Lyz2*^{Cre} mice.

10. Fig. 4D is redundant with Fig. S4B, unless the authors give more details on the difference between the two assays.

Response: Thank you for raising this detailed question. While both Fig. 4D and Fig. S4B validate the sTREM2–NCL interaction, they are complementary in experimental design and perspective.

The core distinction lies in the strategy used to capture the target protein:

Fig. 4D (co-immunoprecipitation): A specific antibody against sTREM2 was used to capture sTREM2 and associated proteins from solution. This approach mimics a scenario in which an antibody recognizes and pulls down the target protein along with its binding partners, demonstrating that NCL is co-precipitated as an interaction partner.

Fig. S4B (pull-down): Biotin-labeled recombinant sTREM2 was captured via its biotin tag using streptavidin-coated beads—a high-affinity interaction independent of antibody recognition. This approach shows that, even in the absence of any antibody (and thus eliminating potential antibody-related artifacts), sTREM2 itself is sufficient to bind directly to NCL.

Together, these two experiments provide rigorous methodological cross-validation: Fig. 4D confirms the sTREM2–NCL interaction within an antibody-mediated recognition system, while Fig. S4B establishes that the interaction is direct and intrinsic to sTREM2, independent of antibody involvement. This dual approach significantly strengthens the robustness of our conclusion by ruling out the possibility that the observed interaction is an artifact of the detection method.

11. Lines 183-184 and Fig. 4I. The RBD region is proposed as the binding site for sTREM2. Is this region exposed to the extracellular space in cell-surface nucleolin?

Response: Thank you for raising this important question. Based on current knowledge and our functional data, the RBD region of NCL is indeed accessible to the extracellular environment when NCL localizes to the plasma membrane.

Although NCL lacks a classical signal peptide, a fraction is transported to the cell surface via a non-canonical pathway and functions as a peripheral membrane receptor (Ref. 13, 14). Extracellular ligands—including viruses, growth factors, and bacterial toxins—interact specifically with the RBDs of surface NCL, indicating that these domains are accessible from the extracellular side (Ref. 15, 16). In addition, surface NCL undergoes N-linked glycosylation at residues within the RBD region (e.g., Asn-317 and Asn-492) (Ref. 17), further supporting extracellular exposure of these domains.

Importantly, our functional evidence further supports this topology. As shown in Fig. 4I, RBD-specific competitive peptides significantly attenuate the interaction between sTREM2 and surface NCL, and structural disruption of the RBD domains

markedly reduces binding. These findings functionally indicate that sTREM2 engages the extracellularly accessible RBD region of cell-surface NCL.

12. Fig. 6A,B. Among the three samples subjected to sTREM2 depletion, at least one shows residual sTREM2 even higher than the non-depleted samples. Yet, the reduction in the biological effect is similar among all three depleted samples (panel B). This would imply that the induction of permeability does not correlate with sTREM2 levels. How can this be explained?

Response: We thank the reviewer for this key observation and apologize for the confusion in the original figure presentation.

Upon careful re-examination, we found that the description of “n = 3 patients” in the original figure legend was inaccurate. Original Fig. 6A depicted sTREM2 depletion efficiency in three independent ascites samples, illustrating variability in baseline sTREM2 levels and depletion efficiency across specimens. In contrast, the permeability assays in Fig. 6B–D were initially performed using a single representative ascites sample treated with different antibody batches to ensure assay reproducibility.

To directly address this concern, we performed additional validation experiments using four independent ovarian cancer ascites samples, bringing the total to five patient-derived samples analyzed in parallel. For each sample, sTREM2 was depleted using an anti-sTREM2 antibody, followed by measurement of both sTREM2 concentrations by ELISA, endothelial permeability using the TRITC-dextran assay in HUVEC, and VE-cadherin phosphorylation levels by Western blotting (Figure 6A-D). Across all five ascites samples, antibody-mediated depletion consistently reduced endothelial permeability. Importantly, we observed a positive correlation between sTREM2 concentrations and permeability (Pearson r = 0.6464; RR Figure 9).

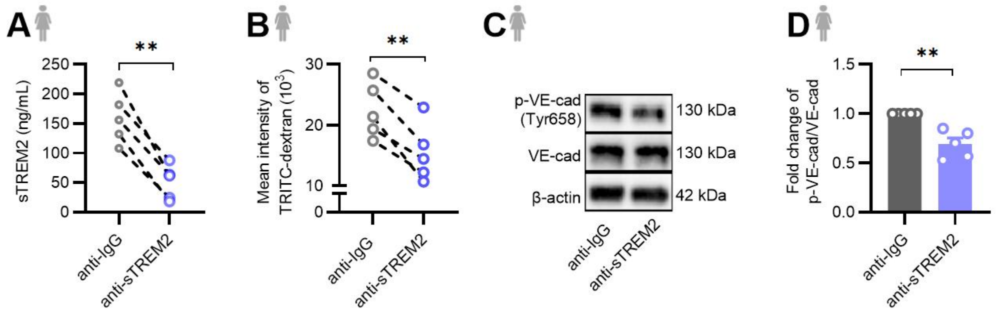

Figure 6. (A) ELISA quantification of sTREM2 depletion efficiency in ovarian cancer ascites (n = 5 patients). (B) TRITC-dextran permeability assay in HUVECs treated with sTREM2-depleted versus control ascites (n = 5 patients). (C, D) Western blot analysis (C) and quantification (D) of p-VE-cadherin (Tyr658) in HUVECs exposed to sTREM2-depleted or non-depleted ascites. β-actin was used as a loading control (n = 5 patients). Data are shown as mean ± SEM. ** $P < 0.01$, two-tailed Student’s t-test for A, B and D.

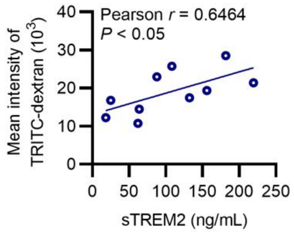

RR Figure 9. Pearson correlation analysis between sTREM2 concentrations and TRITC-dextran permeability in HUVEC treated with patient-derived ascites samples.

Minor points:

1. Fig. 1B: Do the dots shown refer to statistically significant differential expression? For example, TREM2 in "AC" does not appear to be above 1.5 FC in spite of what is claimed in Methods (lines 353-354).

Response: Thank you for the question. In Fig. 1B, the y-axis represents $\log_2$FC, not linear fold change. With a linear FC threshold of 1.5, the corresponding $\log_2$FC is approximately 0.585. To better illustrate, we have replaced the original scatter plot with a violin plot and added statistical annotations for group comparisons (revised Fig. 1B).

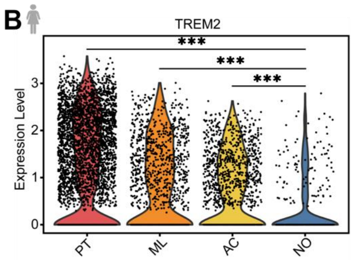

Figure 1B. Differential expression of TREM2 in macrophages from primary tumors (PT), omental metastatic lesions (ML), and malignant ascites (AC) compared with normal ovarian tissue (NO; dataset: HRA002362). Wilcoxon Rank Sum test for B, ***$P < 0.001$.

2. Line 106: Data in Fig. 1F cannot be used to infer a "positive correlation" but rather a general association of sTREM2 levels with ascites volume. Along the same line, why the authors did not perform a correlation analysis as done in Fig. 1G?

Response: Thank you for this important critique. We have revised the description throughout the manuscript to more accurately state that "patients with higher sTREM2 levels tended to present with larger ascites volumes" (Page 5, Line 114-115).

Regarding the absence of a formal correlation analysis for Fig. 1F: ascites volume in clinical records is typically documented as a rough estimate (e.g., categorized as ">500 mL" or based on imaging assessment) rather than a precise continuous variable obtained through standardized measurement. Therefore, unlike the data in Fig. 1G (which are continuous and suitable for correlation analysis), the ascites volume data do not meet the assumptions required for rigorous quantitative correlation analysis.

3. Lines 112-114. "This clinical finding was further corroborated by published proteomic data (19), which demonstrated a significant enrichment of sTREM2 in malignant ascites (Fig. S1E)." Actually, Fig. S1E only shows a Venn diagram with numbers, nothing to do with enrichment of anything. Either the authors provide details on how the differentially expressed proteins have been detected and quantified (together with the list of the protein themselves) or they should just mention that Ref. 19 reports the upregulation of sTREM2 in malignant ascites.

Response: Thank you for pointing this out. We have revised the text to simply state: "This clinical finding is consistent with published proteomic data reporting the accumulation of sTREM2 in malignant ascites" (Page 5, Line 123-124).

4. Line 421. Cell index is introduced here and shown in a few figures as a parameter. Yet, no explanation is given on what it is and how it relates to endothelial permeability?

Response: Thank you for raising this technical point. The Cell Index (CI) is a parameter measured by a real-time cell analyzer (RTCA system) that continuously reflects impedance changes in an adherent cell layer. As endothelial cells form a confluent monolayer, tight junctions restrict electrical current flow, increasing impedance and progressively elevating the Cell Index until it reaches a stable plateau, which indicates an intact endothelial barrier. A decrease in Cell Index later on, reflects disruption of cell–cell junctions and increased permeability. In our experiments, after CI reached plateau, various conditions were applied and the observed drop in CI served as a quantitative, real-time readout of barrier impairment. A detailed description has been included in the **Methods section** (Page20, Line 479-482).

5. Figs. 4D,E. What is "anti-IgG"? The authors probably mean simply "IgG".

Response: You are correct. We have now revised the labels to "IgG".

6. Fig. S5G. The difference between the first bars on the left (without sTREM2) seems statistically significant, is that the case?

Response: Thank you for the careful observation. In Fig. S5G, the two leftmost bars represent the PBS and MK2206 treatment groups (both in the absence of sTREM2). While a direct t-test between these two groups yielded a nominal p-value < 0.05 (RR Figure 10A), this comparison must be interpreted within the appropriate statistical framework. Since this experiment includes four treatment groups, one-way ANOVA was performed as the primary statistical test for multi-group comparisons. The overall ANOVA did not reach statistical significance ($p > 0.05$) (RR Figure 10B). According to standard statistical practice, when the overall test is not significant, post-hoc pairwise comparisons are not warranted, as they would increase the risk of Type I error. Therefore, the observed visual difference between the two control groups is not considered statistically significant in the context of this experiment.

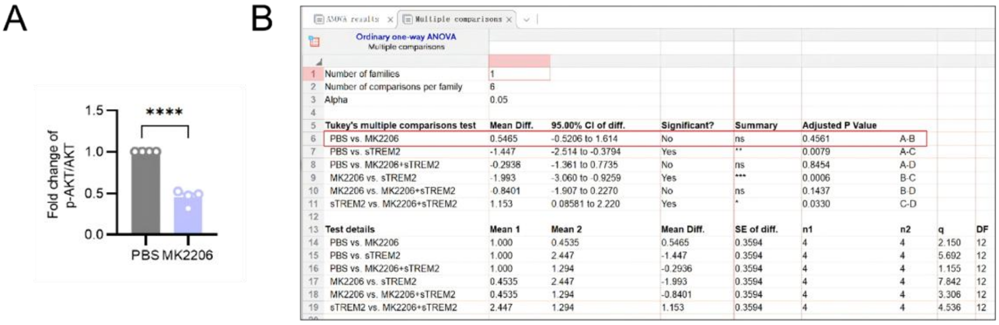

1	Number of families	1							
2	Number of comparisons per family	6							
3	Alpha	0.05							
4									
5	**Tukey's multiple comparisons test**	**Mean Diff.**	**95.00% CI of diff.**	**Significant?**	**Summary**	**Adjusted P Value**			
6	PBS vs. MK2206	0.5465	-0.5206 to 1.614	No	ns	0.4561	A-B		
7	PBS vs. sTREM2	-1.447	-2.514 to -0.3794	Yes	**	0.0079	A-C		
8	PBS vs. MK2206+sTREM2	-0.2938	-1.361 to 0.7735	No	ns	0.8454	A-D		
9	MK2206 vs. sTREM2	-1.993	-3.060 to -0.9259	Yes	***	0.0006	B-C		
10	MK2206 vs. MK2206+sTREM2	-0.8401	-1.907 to 0.2270	No	ns	0.1437	B-D		
11	sTREM2 vs. MK2206+sTREM2	1.153	0.08581 to 2.220	Yes	*	0.0330	C-D		
12									
13	**Test details**	**Mean 1**	**Mean 2**	**Mean Diff.**	**SE of diff.**	**n1**	**n2**	**q**	**DF**
14	PBS vs. MK2206	1.000	0.4535	0.5465	0.3594	4	4	2.150	12
15	PBS vs. sTREM2	1.000	2.447	-1.447	0.3594	4	4	5.692	12
16	PBS vs. MK2206+sTREM2	1.000	1.294	-0.2936	0.3594	4	4	1.155	12
17	MK2206 vs. sTREM2	0.4535	2.447	-1.993	0.3594	4	4	7.842	12
18	MK2206 vs. MK2206+sTREM2	0.4535	1.294	-0.8401	0.3594	4	4	3.306	12
19	sTREM2 vs. MK2206+sTREM2	2.447	1.294	1.153	0.3594	4	4	4.536	12

RR Figure 10. (A) Western blot quantification of AKT phosphorylation in HUVEC treated with PBS or MK2206 (data extracted from Fig. S5G). Data are shown as mean ± SEM. A two-tailed Student's t-test yielded ****$P < 0.0001$. (B) One-way ANOVA of all experimental groups in Fig. S5G (including PBS, MK2206, sTREM2, and sTREM2+MK2206). The overall ANOVA was not significant ($p > 0.05$), indicating no statistically significant differences among groups after correction for multiple comparisons.

Referee #3:

This is a well performed study supporting the macrophage-produced soluble TREM2 (sTREM2) as a key factor to engage cell-surface Nucleolin (NCL) to drive vascular permeability and malignant ascites in ovarian cancer by using the sTREM2-NCL-AKT-eNOS signaling axis in endothelial cells. I only have several minor points:

1. Fig. 1B is supposed to show TREM2 was significantly upregulated in primary tumors, metastatic lesions, and malignant ascites compared to normal ovarian tissues. But it is unclear what are really shown in this figure panel and there is also no statistics provided to support the claim of "significantly upregulated".

Response: We thank the reviewer for this important comment. In accordance with your suggestion, we have made substantial updates to Figure 1B.

In the revised manuscript, we have replaced the scatter plot with a violin plot to better illustrate data distribution and facilitate group comparisons (revised Figure 1B). Importantly, we performed pairwise statistical comparisons between normal ovarian tissues and each of the three tumor-associated groups: primary tumors, metastatic lesions, and malignant ascites. Significance levels are now indicated with asterisks. This revision provides direct visual and statistical support for the conclusion.

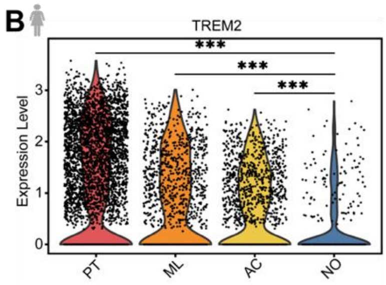

Figure 1B. Differential expression of TREM2 in tumor-associated macrophages from primary tumors (PT), omental metastatic lesions (ML), and malignant ascites (AC) compared with normal ovarian tissue (violin plot; dataset: HRA002362). Wilcoxon Rank Sum test for B, ***$P < 0.001$.

2. Fig. 1F shows TREM2 expression levels have a significant positive correlation with ascites volume and Fig. 1H further shows the same correlation for sTREM2. What is the level of macrophages, particularly TREM2+ macrophages in malignant ascites? Is the positive correlation of sTREM2 level with ascites volume simply due to higher numbers of TREM2+ macrophages in larger volumes of ascites?

Response: Thank you for raising this profound and critical question. To address it, we performed additional flow cytometry analysis to examine the relationship between macrophage abundance and ascites volume, as summarized below.

Based on our flow cytometry data, in patients with larger ascites volume (≥ 500 mL), the proportion of macrophages among nucleated cells is significantly higher (~17.8%) compared to patients with smaller volume ascites (< 500 mL, ~6.2%) (RR Figure 11), suggesting a greater absolute number of macrophages in larger ascites.

However, we believe this does not fully explain the correlation between sTREM2 levels and ascites volume. In Fig. 1F, the y-axis represents the MFI of TREM2—the mean fluorescence intensity of TREM2 protein on ascites-derived macrophages—which is a per-cell metric that inherently normalizes for absolute cell numbers. The positive correlation of this per-cell expression level with ascites volume indicates that in patients with larger ascites, macrophages not only are more abundant but also exist in a more activated state characterized by higher TREM2 expression per cell, a phenotypic shift that cannot be attributed solely to increased cell numbers.

Furthermore, sTREM2 shedding, primarily mediated by ADAM10/17 proteases, closely reflects macrophage activation status. Thus, the correlation between sTREM2 concentration and ascites volume more likely reflects a sustained activation of macrophages driven by the ascites microenvironment, leading to enhanced TREM2 expression and subsequent ADAM-mediated cleavage.

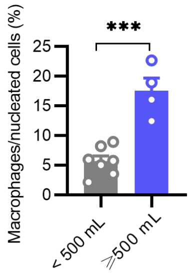

RR Figure 11. Macrophage proportion in the Fig. 1F patient cohort. Flow cytometric analysis of CD45⁺ CD68⁺ macrophages among nucleated cells in ascites from patients with small (< 500 mL, n = 7 patients) or large (≥ 500 mL, n = 4 patients) ascites volume. Data are shown as mean ± SEM. ****P* < 0.001, two-tailed Student's t-test for A.

3. The authors described their results in a very succinct way, but sometimes it appears to lack sufficient details for readers to fully understand their experiments: for instance, the in vitro coculture system consisting of endothelial cells and macrophages with differentially expressed TREM2: it seems the authors used two cocultures, human HUVECs cocultured with THP-1-derived macrophage in Fig. 2, as well as murine C166 endothelial cells cocultured with RAW264.7 macrophages in Fig. S2. In Methods section, under "Transfection and establishment of stable cell lines", the authors described how they modulated TREM2 levels in RAW264.7 cell lines, but not THP-1 cells.

Response: We thank the reviewer for pointing out this lack of methodological detail and have now clarified the procedures for generating THP-1–derived macrophages with altered sTREM2 expression.

We have added detailed descriptions of THP-1 genetic modification in the **Methods section** (Page 19 Line 455-461). Specifically, THP-1 cells were transduced with a lentiviral vector encoding human sTREM2 fused with an HA tag and mScarlet reporter to establish a stable overexpression cell line. Transduction efficiency was verified by mScarlet fluorescence, and secretion of sTREM2 into the culture medium was quantified by ELISA (revised Fig. EV2I). For the depletion experiments (Fig.

2M,N), we have added ELISA data demonstrating efficient immunoprecipitation-mediated depletion of sTREM2 from conditioned media (revised Fig. EV2K).

To further clarify the experimental design, we have also included a schematic diagram illustrating the two complementary coculture systems used in this study—HUVECs indirectly cocultured with THP-1 derived macrophages and murine C166 directly cocultured with RAW264.7 macrophages (revised Fig. EV2E).

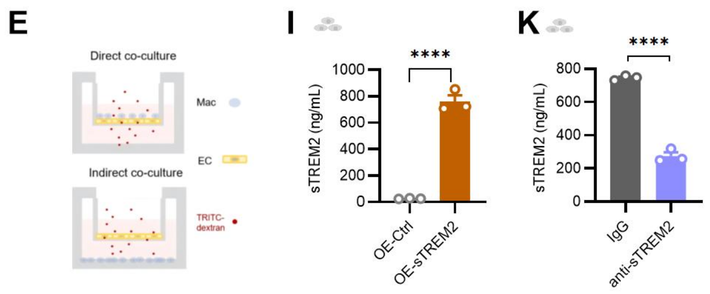

Figure EV2. (E) Schematic diagram of direct and indirect co-culture. (I) sTREM2 levels in the culture supernatants of THP-1 cells, detected by ELISA (n = 3 biological replicates). (K) sTREM2 levels in conditioned medium from sTREM2-overexpressing THP-1 cells before and after immunoprecipitation-mediated depletion with an anti-sTREM2 antibody, detected by ELISA (n = 3 biological replicates). Data are mean ± SEM. ****$P < 0.0001$, two-tailed Student's t-test for I and K.

4. The authors need to provide more detail for Trem2MacKO mice. Assuming this is a macrophage-specific conditional knockout model for Trem2, is Trem2 allele here a floxed conditional KO allele? If so, is there a reference for it? What is the macrophage-specific Cre line to knock it out? If it's not a published model, the authors should provide more detail about the mouse line and also some data to demonstrate the macrophage-specific loss of Trem2.

Response: Thank you for raising this important point regarding the animal model. We have now provided detailed information on its genetic background, generation, and validation.

The mice used are more accurately described as myeloid cell-specific *Trem2* conditional knockout mice. The construction and validation of this model have been described in detail previously (Ref. 12). Briefly, the model was generated by crossing mice carrying a floxed *Trem2* allele (*Trem2*$^{fl/fl}$) with *Lyz2*^{Cre} transgenic mice, in which Cre recombinase expression is driven by the lysozyme M promoter to achieve specific deletion in myeloid cells, including macrophages.

To directly confirm the model's validity in our study, we have supplemented the manuscript with the following evidence: 1) genotyping results confirming that the mice used were of the *Trem2*$^{fl/fl}$*Lyz2*^{Cre} genotype (RR Figure 8); and 2) Western blot analysis demonstrating effective Trem2 knockout in bone marrow-derived macrophages (revised Figure EV2J). All related information has been integrated into the **Methods and Results sections**.

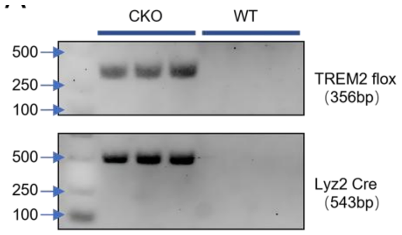

RR Figure 8. Identification of gene knockout mice. The gene phenotypes of *Trem2*$^{fl/fl}$*Lyz2*^{Cre} mice were respectively identified by PCR.

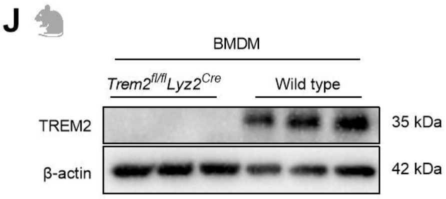

Figure EV2J. Western blot validation of TREM2 knockout in bone marrow-derived macrophages from *Trem2*$^{fl/fl}$*Lyz2*^{Cre} mice.

5. In Fig. 3, the authors studied macrophage-specific TREM2 function in vivo by co-injecting ID8 cells and RAW264.7 macrophages (either TREM2-overexpressing, knockdown or control) to C57BL/6 female mice. The ID8 cell line is under the same C57BL/6 background, but the RAW264.7 macrophage cell line is under the BALB/c background (based on ATCC). How could injection of RAW264.7 cells into C57BL/6 mice not induce any immune rejection?

Response: Thank you for raising this important concern. To rigorously address this issue, we performed additional *in vivo* experiments using syngeneic iBMDM cells derived from the C57BL/6 background, as summarized below.

iBMDM cells were purchased from Shanghai Fuheng Biotechnology Co., Ltd. (Cat. No. FH-Y032). All key experiments originally conducted with RAW264.7 cells were independently repeated using iBMDM with corresponding TREM2 overexpression or knockdown modifications (revised Figure EV2A–D).

Importantly, the results obtained using syngeneic iBMDM were consistent with those observed using RAW264.7 cells: 1) TREM2 overexpression in iBMDM significantly increased ascites volume, tumor burden, and sTREM2 levels compared to controls (revised Figure 3A–F); 2) TREM2 knockdown in iBMDM significantly reduced ascites formation, tumor burden, and sTREM2 levels (revised Figure EV3A–F); 3) TREM2-overexpressing iBMDM enhanced peritoneal vascular permeability in vivo, consistent with the pro-permeability role of sTREM2 (revised Figure EV2H). These complementary experiments using fully syngeneic macrophages further demonstrate that the observed phenotypes reflect TREM2-dependent mechanisms in macrophages.

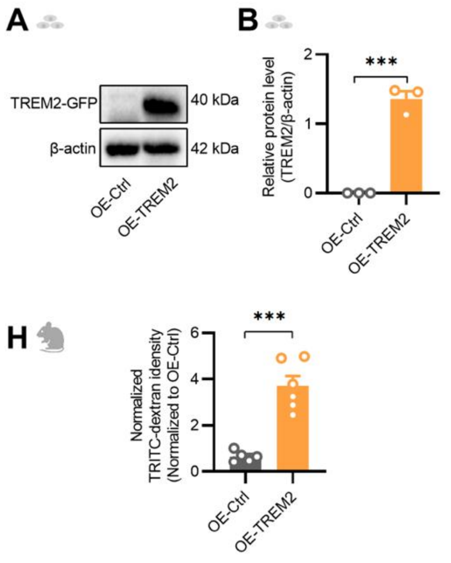

Figure EV2. (A, B) Western blot analysis (A) and quantification (B) of TREM2 expression in iBMDM macrophages overexpressing TREM2 (OE-TREM2) versus empty vector control (OE-Ctrl); β-actin as loading control (n = 3 biological replicates). (C, D) Western blot analysis (C) and quantification (D) of TREM2 expression in iBMDM macrophages with TREM2 knockdown (Sh-TREM2) versus scramble control (Sh-Ctrl); β-actin as loading control (n = 3 biological replicates). (H) Peritoneal vascular permeability in mice injected intraperitoneally with 1 × 10^6 TREM2-overexpressing iBMDM (n = 5 mice) versus control iBMDM cells (n = 6 mice). Data are mean ± SEM. ***P* < 0.01, ****P* < 0.001, two-tailed Student's t-test for B, D and H.

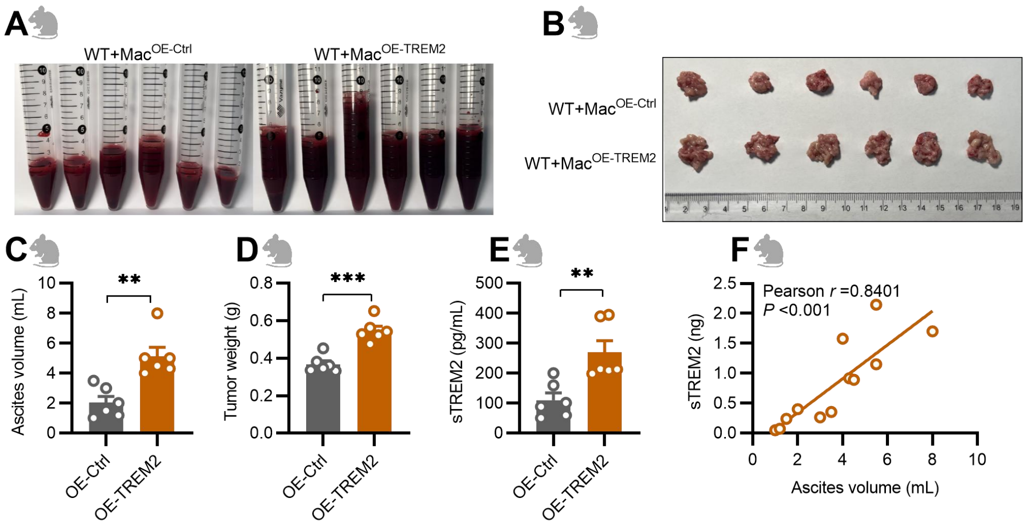

Figure 3. (A-D) Representative images and quantification of ascites volume (A, C) and tumor burden (B, D) in mice following intraperitoneal injection of OE-TREM2 or OE-Ctrl iBMDM in combination with ID8 ovarian cancer cells (n = 6 mice). (E) ELISA measurement of sTREM2 levels in ascites supernatant from (A). (F) Correlation analysis between ascites volume and sTREM2 levels from (E). Data are shown as mean ± SEM. ***P* < 0.01; ****P* < 0.001; two-tailed Student's t-test for C-E, Pearson correlation analysis for F.

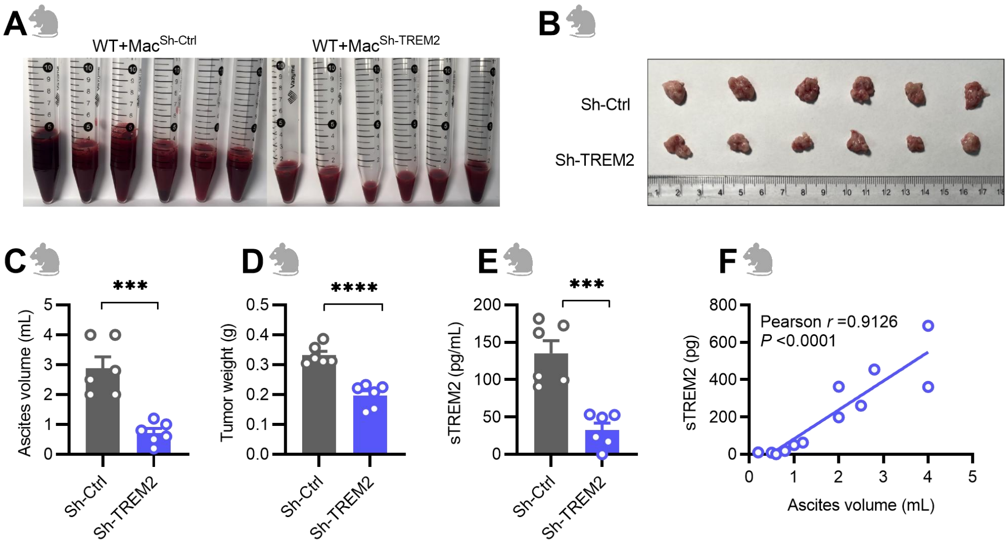

Figure EV3. TREM2 knockdown inhibits malignant ascites formation in ovarian cancer. (A, C) Representative images (A) and quantification (C) of ascites in mice injected intraperitoneally with Sh-TREM2 iBMDM cells and ID8 ovarian cancer cells versus Sh-Ctrl iBMDM and ID8 cells (n = 6 mice). (B, D) Intraperitoneal tumor formation (B) and quantification (D) in the same experimental groups as (A) (n = 6 mice). (E) ELISA measurement of sTREM2 levels in ascites supernatant from (A). (F) Correlation analysis between ascites volume and sTREM2 concentration from (E). Data are shown as mean ± SEM. $^{**}P < 0.01$, $^{***}P < 0.001$; $^{****}P < 0.0001$, two-tailed Student's t-test for C-E, Pearson correlation analysis for F.

Reference

1. Yin P, Jiang H, Ji X, Xia L, Su Z, Tian Y. Soluble TREM2 drives triple-negative breast cancer progression via activation of the AKT pathway. Int Immunopharmacol. 2025;145:113750.
2. Liu G, Wang J, Han M, et al. RNA-binding domain 2 of nucleolin is important for the autophagy induction of curcumol in nasopharyngeal carcinoma cells. Phytomedicine. 2023;115:154833.
3. Shin SH, Lee GY, Lee M, et al. Aberrant expression of CITED2 promotes prostate cancer metastasis by activating the nucleolin-AKT pathway. Nat Commun. 2018;9(1):4113.
4. Xu L, Ma W, Huo X, et al. New insights into the function and mechanisms of piRNA PMLCPIR in promoting PM2.5-induced lung cancer. J Adv Res. 2025;73:659-670.
5. Zhang S, Xie B, Wang L, et al. Macrophage-mediated vascular permeability via VLA4/VCAM1 pathway dictates ascites development in ovarian cancer. J Clin Invest. 2021;131(3):e140315.
6. He H, Mack JJ, Güç E, et al. Perivascular macrophages limit permeability. Arterioscler Thromb Vasc Biol. 2016;36(11):2203-2212.
7. Zheng X, Wang X, Cheng X, et al. Single-cell analyses implicate ascites in remodeling the ecosystems of primary and metastatic tumors in ovarian cancer. Nat Cancer. 2023;4(8):1138-1156.
8. Zhong L, Chen XF, Wang T, et al. Soluble TREM2 induces inflammatory responses and enhances microglial survival. J Exp Med. 2017;214(3):597-607.
9. Zhang X, Tang L, Yang J, et al. Soluble TREM2 ameliorates tau phosphorylation and cognitive deficits through activating transgelin-2 in Alzheimer's disease. Nat Commun. 2023;14(1):6670.
10. Kleinberger G, Yamanishi Y, Suárez-Calvet M, et al. TREM2 mutations implicated in neurodegeneration impair cell surface transport and phagocytosis. Sci Transl Med. 2014;6(243):243ra86.
11. Wunderlich P, Glebov K, Kemmerling N, Tien NT, Neumann H, Walter J. Sequential proteolytic processing of the triggering receptor expressed on myeloid cells-2 (TREM2) protein by ectodomain shedding and γ-secretase-dependent intramembranous cleavage. J Biol Chem. 2013;288(46):33027-33036.
12. Ming S, Li X, Xiao Q, et al. TREM2 aggravates sepsis by inhibiting fatty acid oxidation via the SHP1/BTK axis. J Clin Invest. 2024;135(1):e159400.
13. Prakash K, Satishkartik S, Ramalingam S, Gangadaran P, Gnanavel S, Aruljothi KN. Investigating the multifaceted role of nucleolin in cellular function and cancer: structure, regulation, and therapeutic implications. Gene. 2025;957:149479.
14. Jia W, Yao Z, Zhao J, Guan Q, Gao L. New perspectives of physiological and pathological functions of nucleolin (NCL). Life Sci. 2017;186:1-10.
15. Tonello F, Massimino ML, Peggion C. Nucleolin: a cell portal for viruses, bacteria, and toxins. Cell Mol Life Sci. 2022;79(5):271.
16. Kirman DC, Renganathan B, Chui WK, et al. Cell surface nucleolin is a novel ADAMTS5 receptor mediating endothelial cell apoptosis. Cell Death Dis. 2022;13(2):172.
17. Carpentier M, Morelle W, Coddeville B, et al. Nucleolin undergoes partial N- and O-glycosylations in the extranuclear cell compartment. Biochemistry. 2005;44(15):5804-5815.

18th Apr 2026

Dear Prof. He,

Thank you for the submission of your revised manuscript to EMBO Molecular Medicine. We have now received the enclosed report from the three referees who were asked to re-assess it. As you will see, the referees are now supportive, and I am pleased to inform you that we will be able to accept your manuscript pending the following amendments:

1. the remaining minor issues mentioned by the referees.

On a more editorial level:

1. Please provide an institutional email address for Shuang Guo.

2. Please remove the "Authorship Note" from the manuscript text and the entire "Author Contributions" section.

3. Please provide a "standfirst text" summarizing the study in one or two sentences (approximately 250 characters, including space), three to four "bullet points" highlighting the main findings and a " visual abstract" (exactly 550px width and 300 px height, PNG format) to highlight the paper on our homepage.

4. Provide a "The paper explained" section in the manuscript file: EMBO Molecular Medicine articles are accompanied by a summary of the articles to emphasize the major findings in the paper and their medical implications for the non-specialist reader. Please provide a draft summary of your article highlighting

- the medical issue you are addressing,

- the results obtained and

- their clinical impact.

This may be edited to ensure that readers understand the significance and context of the research. Please refer to any of our published articles for an example.

5. Author checklist: include the names of all corresponding authors.

6. Merge the funding information into the Acknowledgments section, and remove the separate "Funding Support" heading.

7. Data availability: The provided URL does not appear to be accessible. Please provide a resolvable link and ensure that the deposited data (PRJNA1332085) are publicly available.

8. Please note that Appendix Table S2 should not be called out before Appendix Table S1.

9. Please remove the numbers in the reference list.

10. Please address the following issue in the figure legends:
- Please note that the exact p values are not provided in the legends of figures 2F, L; 3I, J, K, L, N; 5E, F, G, N; 6H, L; EV1 B,EV2 I, EV3 D, F; EV4 F, H; EV5 G.
- Please indicate the statistical test used for data analysis in the legends of figures 5A, 6J.
- Please note that the box plots need to be defined in terms of minima, maxima, centre, bounds of box and whiskers, and percentile in the legend of figure EV1 B.
- Please note that information related to n is missing in the legends of figures 1A, B; EV2 I.

I look forward to reading a new revised version of your manuscript as soon as possible.

Kind regards,
Jingyi

Jingyi Hou
Senior Editor
EMBO Molecular Medicine

*** Instructions to submit your revised manuscript ***

*** PLEASE NOTE *** As part of the EMBO Publications transparent editorial process initiative (see our Editorial at https://www.embopress.org/doi/pdf/10.1002/emmm.201000094), EMBO Molecular Medicine will publish online a Review Process File to accompany accepted manuscripts.

In the event of acceptance, this file will be published in conjunction with your paper and will include the anonymous referee reports, your point-by-point response and all pertinent correspondence relating to the manuscript. If you do NOT want this file to be published, please inform the editorial office at contact@embomolmed.org.

***** Reviewer's comments *****

Referee #1 (Comments on Novelty/Model System for Author):

The authors addressed the previous concerns very well. The revised data lend credence to the conclusions reached. This manuscript is addressing a significant malignancy, OC, and the major complication of this disease. The findings are novel and can open up new therapeutic options to address malignant ascites.

Referee #1 (Remarks for Author):

The authors adequately address all prior concerns raised. This reviewer supports the publication of this manuscript.

Referee #2 (Comments on Novelty/Model System for Author):

The technical quality has improved significantly in the revised version. This also includes a more careful selection and analysis of the model systems.

Referee #2 (Remarks for Author):

The authors have properly addressed all my comments, and the revised version of the manuscript has markedly improved.

Referee #3 (Remarks for Author):

In this revised work, the authors have sufficiently addressed reviewers' concerns.

In the newly added data using the iBMDM cells (Fig. 3), the authors included the Cat. No. in the responses to reviewers file ["iBMDM cells were purchased from Shanghai Fuheng Biotechnology Co., Ltd (Cat. No. FH-Y032)"]. But this information should be included in the Methods section, page 18, lines 432-433; in addition, it should be clearly described here that these iBMDM cells were under the syngeneic C57BL/6 background.

Response to the Editor and Reviewers

We sincerely thank the editor and reviewers for your positive feedback and suggestions. We have carefully addressed all remaining comments and editorial requests. All changes have been incorporated into the revised manuscript and the detailed point-by-point responses are outlined below in blue.

Editorial Comments

1. Please provide an institutional email address for Shuang Guo.
Response: The institutional email address for Dr. Shuang Guo has been provided.

2. Please remove the "Authorship Note" and the entire "Author Contributions" section.
Response: These sections have been removed.

3. Please provide a "standfirst text" summarizing the study in one or two sentences (approximately 250 characters, including space), three to four "bullet points" highlighting the main findings and a " visual abstract" (exactly 550px width and 300 px height, PNG format) to highlight the paper on our homepage.
Response: Please refer to the file “Visual Abstract and Texts”.

4. Provide a "The paper explained" section in the manuscript file: EMBO Molecular Medicine articles are accompanied by a summary of the articles to emphasize the major findings in the paper and their medical implications for the non-specialist reader. Please provide a draft summary of your article highlighting the medical issue you are addressing, the results obtained and their clinical impact.
This may be edited to ensure that readers understand the significance and context of the research. Please refer to any of our published articles for an example.
Response: This section has been added to the manuscript.

5. Author checklist: include the names of all corresponding authors.
Response: The author checklist has been updated.

6. Merge the funding information into the Acknowledgments section, and remove the separate "Funding Support" heading.
Response: Funding information has been merged into the Acknowledgments section.

7. Data availability: The provided URL does not appear to be accessible. Please provide a resolvable link and ensure that the deposited data (PRJNA1332085) are publicly available.
Response: We have updated the link. The dataset is now publicly available at: https://www.ncbi.nlm.nih.gov/sra/?term=PRJNA1332085.

8. Please note that Appendix Table S2 should not be called out before Appendix Table S1.

Response: We have swapped the order of the two Appendix Tables. The in-text citations have been updated accordingly.

9. Please remove numbers in the reference list.
Response: The numbering in the reference list has been removed.

10. Please address the following issue in the figure legends:
- Please note that the exact p values are not provided in the legends of figures 2F, L; 3I, J, K, L, N; 5E, F, G, N; 6H, L; EV1 B,EV2 I, EV3 D, F; EV4 F, H; EV5 G.
Response: Exact p-values have now been provided in Appendix Table for all indicated figures.

- Please indicate the statistical test used for data analysis in the legends of figures 5A, 6J.
Response: Statistical methods have been added to the relevant figure legends.

- Please note that the box plots need to be defined in terms of minima, maxima, centre, bounds of box and whiskers, and percentile in the legend of figure EV1 B.
Response: We have added the following description to the legend: "Boxplots show the median (central line), the upper and lower quartiles (25th and 75th percentiles), and whiskers indicating the minimum and maximum values."

- Please note that information related to n is missing in the legends of figures 1A, B; EV2 I.
Response: Sample size (n) information has been added to the legends of Figures 1A, 1B, and EV2I.

Reviewer Comments

Referee #1 & Referee #2

We thank the reviewer for the positive assessment and support for publication. No further changes were requested.

Referee #3

In this revised work, the authors have sufficiently addressed reviewers' concerns.
In the newly added data using the iBMDM cells (Fig. 3), the authors included the Cat. No. in the responses to reviewers file ["iBMDM cells were purchased from Shanghai Fuheng Biotechnology Co., Ltd (Cat. No. FH-Y032)"]. But this information should be included in the Methods section, page 18, lines 432-433; in addition, it should be clearly described here that these iBMDM cells were under the syngeneic C57BL/6 background.
Response: We have now incorporated this information into the Methods section: "Immortalized bone marrow–derived macrophages (iBMDM) were purchased from Fuheng (China) and were on a syngeneic C57BL/6 background."

28th Apr 2026

Dear Prof. He,

We are pleased to inform you that your manuscript is accepted for publication and is now being sent to our publisher to be included in the next available issue of EMBO Molecular Medicine.

Your manuscript will be processed for publication by EMBO Press. It will be copy edited and you will receive page proofs prior to publication. Please note that you will be contacted by Springer Nature Author Services to complete licensing and payment information.

You may qualify for financial assistance for your publication charges - either via a Springer Nature fully open access agreement or an EMBO initiative. Check your eligibility: https://link.springer.com/journal/44321/how-to-publish-with-us

Should you be planning a Press Release on your article, please get in contact with embo_production@springernature.com as early as possible in order to coordinate publication and release dates.

If you have any questions, please do not hesitate to contact the Editorial Office. Thank you for your contribution to EMBO Molecular Medicine.

Sincerely,
Jingyi

Jingyi Hou
Senior Editor
EMBO Molecular Medicine

>>> Please note that it is EMBO Molecular Medicine policy for the transcript of the editorial process (containing referee reports and your response letter) to be published as an online supplement to each paper. If you do NOT want this, you will need to inform the Editorial Office via email immediately. More information is available here: https://link.springer.com/partners/embo-press/editorial-policies#Peer%20review

EMBO Press Author Checklist

Corresponding Author Name: Xi Huang, Shuang Guo, Bingfan Xie, and Huanhuan He	
Journal Submitted to: EMBO Molecular Medicine	
Manuscript Number: EMM-2025-22943	

USEFUL LINKS FOR COMPLETING THIS FORM
The EMBO Journal - Author Guidelines
EMBO Reports - Author Guidelines
Molecular Systems Biology - Author Guidelines
EMBO Molecular Medicine - Author Guidelines

Reporting Checklist for Life Science Articles (updated January
This checklist is adapted from Materials Design Analysis Reporting (MDAR) Checklist for Authors. MDAR establishes a minimum set of requirements in transparent reporting in the life sciences (see Statement of Task: 10.31222/osf.io/9sm4x). Please follow the journal's guidelines in preparing your
Please note that a copy of this checklist will be published alongside your article.

Abridged guidelines for figures
1. Data
The data shown in figures should satisfy the following conditions:
- the data were obtained and processed according to the field's best practice and are presented to reflect the results of the experiments in an accurate and unbiased manner.
- ideally, figure panels should include only measurements that are directly comparable to each other and obtained with the same assay.
- plots include clearly labeled error bars for independent experiments and sample sizes. Unless justified, error bars should not be shown for technical
- if n<5, the individual data points from each experiment should be plotted. Any statistical test employed should be justified.
- Source Data should be included to report the data underlying figures according to the guidelines set out in the authorship guidelines on Data

2. Captions
Each figure caption should contain the following information, for each panel where they are relevant:
- a specification of the experimental system investigated (eg cell line, species name).
- the assay(s) and method(s) used to carry out the reported observations and measurements.
- an explicit mention of the biological and chemical entity(ies) that are being measured.
- an explicit mention of the biological and chemical entity(ies) that are altered/varied/perturbed in a controlled manner.
- the exact sample size (n) for each experimental group/condition, given as a number, not a range;
- a description of the sample collection allowing the reader to understand whether the samples represent technical or biological replicates (including how many animals, litters, cultures, etc.).
- a statement of how many times the experiment shown was independently replicated in the laboratory.
- definitions of statistical methods and measures:
  - common tests, such as t-test (please specify whether paired vs. unpaired), simple χ2 tests, Wilcoxon and Mann-Whitney tests, can be unambiguously identified by name only, but more complex techniques should be described in the methods section;
  - are tests one-sided or two-sided?
  - are there adjustments for multiple comparisons?
  - exact statistical test results, e.g., P values = x but not P values < x;
  - definition of 'center values' as median or average;
  - definition of error bars as s.d. or s.e.m.

Please complete ALL of the questions below.
Select "Not Applicable" only when the requested information is not relevant for your study.

Materials

Newly Created Materials	Information included in the manuscript?	In which section is the information available? (Reagents and Tools Table, Materials and Methods, Figures, Data Availability Section)
New materials and reagents need to be available; do any restrictions apply?	Yes	Reagents and Tools Table, Materials and Methods

Antibodies	Information included in the manuscript?	In which section is the information available? (Reagents and Tools Table, Materials and Methods, Figures, Data Availability Section)
For **antibodies** provide the following information:<br>- Commercial antibodies: RRID (if possible) or supplier name, catalogue number and or/clone number<br>- Non-commercial: RRID or citation	Yes	Reagents and Tools Table, Materials and Methods

DNA and RNA sequences	Information included in the manuscript?	In which section is the information available? (Reagents and Tools Table, Materials and Methods, Figures, Data Availability Section)
**Short novel DNA or RNA including primers, probes**: provide the sequences.	Yes	Appendix Table

Cell materials	Information included in the manuscript?	In which section is the information available? (Reagents and Tools Table, Materials and Methods, Figures, Data Availability Section)
**Cell lines:** Provide species information, strain. Provide accession number in repository **OR** supplier name, catalog number, clone number, and/**OR** RRID.	Yes	Materials and Methods
**Primary cultures:** Provide species, strain, sex of origin, genetic modification status.	Yes	Materials and Methods
Report if the cell lines were recently **authenticated** (e.g., by STR profiling) and tested for mycoplasma contamination.	Yes	Materials and Methods

Experimental animals	Information included in the manuscript?	In which section is the information available? (Reagents and Tools Table, Materials and Methods, Figures, Data Availability Section)
**Laboratory animals or Model organisms:** Provide species, strain, sex, age, genetic modification status. Provide accession number in repository **OR** supplier name, catalog number, clone number, **OR** RRID.	Yes	Reagents and Tools Table, Materials and Methods
**Animal observed in or captured from the field:** Provide species, sex, and age where possible.	Not Applicable	
Please detail **housing and husbandry conditions**.	Yes	Materials and Methods

Plants and microbes	Information included in the manuscript?	In which section is the information available? (Reagents and Tools Table, Materials and Methods, Figures, Data Availability Section)
**Plants:** provide species and strain, ecotype and cultivar where relevant, unique accession number if available, and source (including location for collected wild specimens).	Not Applicable	
**Microbes:** provide species and strain, unique accession number if available, and source.	Not Applicable	

Human research participants	Information included in the manuscript?	In which section is the information available? (Reagents and Tools Table, Materials and Methods, Figures, Data Availability Section)
If collected and within the bounds of privacy constraints report on age, sex and gender or ethnicity for all study participants.	Yes	Materials and Methods

Core facilities	Information included in the manuscript?	In which section is the information available? (Reagents and Tools Table, Materials and Methods, Figures, Data Availability Section)
If your work benefited from core facilities, was their service mentioned in the acknowledgments section?	Not Applicable	

Design

Study protocol	Information included in the manuscript?	In which section is the information available? (Reagents and Tools Table, Materials and Methods, Figures, Data Availability Section)
If study protocol has been **pre-registered, provide DOI in the manuscript**. For clinical trials, provide the trial registration number **OR** cite DOI.	Not Applicable	
Report the **clinical trial registration number** (at ClinicalTrials.gov or equivalent), where applicable.	Not Applicable	

Laboratory protocol	Information included in the manuscript?	In which section is the information available? (Reagents and Tools Table, Materials and Methods, Figures, Data Availability Section)
Provide DOI OR other citation details if **external detailed step-by-step protocols** are available.	Not Applicable	

Experimental study design and statistics	Information included in the manuscript?	In which section is the information available? (Reagents and Tools Table, Materials and Methods, Figures, Data Availability Section)
Include a statement about **sample size** estimate even if no statistical methods were used.	Yes	Materials and Methods
Were any steps taken to minimize the effects of subjective bias when allocating animals/samples to treatment (e.g. **randomization procedure**)? If yes, have they been described?	Yes	Materials and Methods
Include a statement about **blinding** even if no blinding was done.	Yes	Materials and Methods
Describe **inclusion/exclusion criteria** if samples or animals were excluded from the analysis. Were the criteria pre-established?<br><br>If sample or data points were omitted from analysis, report if this was due to attrition or intentional exclusion and provide justification.	Yes	Materials and Methods
For every figure, are **statistical tests** justified as appropriate? Do the data meet the assumptions of the tests (e.g., normal distribution)? Describe any methods used to assess it. Is there an estimate of variation within each group of data? Is the variance similar between the groups that are being statistically compared?	Yes	Materials and Methods

Sample definition and in-laboratory replication	Information included in the manuscript?	In which section is the information available? (Reagents and Tools Table, Materials and Methods, Figures, Data Availability Section)
In the figure legends: state number of times the experiment was **replicated** in laboratory.	Yes	Figure Legends
In the figure legends: define whether data describe **technical or biological replicates**.	Yes	Figure Legends

Ethics

Ethics	Information included in the manuscript?	In which section is the information available? (Reagents and Tools Table, Materials and Methods, Figures, Data Availability Section)
Studies involving **human participants**: State details of **authority granting ethics approval** (IRB or equivalent committee(s), provide reference number for approval.	Yes	Materials and Methods
Studies involving **human participants**: Include a statement confirming that **informed consent** was obtained from all subjects and that the experiments conformed to the principles set out in the WMA Declaration of Helsinki and the Department of Health and Human Services Belmont Report.	Yes	Materials and Methods
Studies involving **human participants:** For publication of **patient photos**, include a statement confirming that consent to publish was obtained.	Not Applicable	
Studies involving experimental **animals**: State details of **authority granting ethics approval** (IRB or equivalent committee(s), provide reference number for approval. Include a statement of compliance with ethical regulations.	Yes	Materials and Methods
Studies involving **specimen and field samples:** State if relevant **permits** obtained, provide details of authority approving study; if none were required, explain why.	Yes	Materials and Methods

Dual Use Research of Concern (DURC)	Information included in the manuscript?	In which section is the information available? (Reagents and Tools Table, Materials and Methods, Figures, Data Availability Section)
Could your study fall under dual use research restrictions? Please check biosecurity documents and list of **select agents and toxins** (CDC): https://www.selectagents.gov/sat/list.htm	Not Applicable	
If you used a select agent, is the security level of the lab appropriate and reported in the manuscript?	Not Applicable	
If a study is subject to dual use research of concern regulations, is the name of the **authority granting approval and reference number** for the regulatory approval provided in the manuscript?	Not Applicable	

Reporting

The MDAR framework recommends adoption of discipline-specific guidelines, established and endorsed through community initiatives. Journals have their own policy about requiring specific guidelines and recommendations to complement MDAR.

Adherence to community standards	Information included in the manuscript?	In which section is the information available? (Reagents and Tools Table, Materials and Methods, Figures, Data Availability Section)
State if relevant guidelines or checklists (e.g., **ICMJE, MIBBI, ARRIVE, PRISMA**) have been followed or provided.	Not Applicable	
For **tumor marker prognostic studies,** we recommend that you follow the **REMARK** reporting guidelines (see link list at top right). See author guidelines, under 'Reporting Guidelines'. Please confirm you have followed these guidelines.	Not Applicable	
For **phase II and III randomized controlled trials**, please refer to the **CONSORT** flow diagram (see link list at top right) and submit the CONSORT checklist (see link list at top right) with your submission. See author guidelines, under 'Reporting Guidelines'. Please confirm you have submitted this list.	Not Applicable	

Data Availability

Data availability	Information included in the manuscript?	In which section is the information available? (Reagents and Tools Table, Materials and Methods, Figures, Data Availability Section)
Have **primary datasets** been deposited according to the journal's guidelines (see 'Data Deposition' section) and the respective accession numbers provided in the Data Availability Section?	Yes	Data Availability Section
Were **human clinical and genomic datasets** deposited in a public access-controlled repository in accordance to ethical obligations to the patients and to the applicable consent agreement?	Yes	Data Availability Section
Are **computational models** that are central and integral to a study available without restrictions in a machine-readable form? Were the relevant accession numbers or links provided?	Not Applicable	
If publicly available data were reused, provide the respective **data citations in the reference list**.	Yes	Reference List
